# Iron-Overload triggers ADAM-17 mediated inflammation in Severe Alcoholic Hepatitis

**DOI:** 10.1038/s41598-018-28483-x

**Published:** 2018-07-06

**Authors:** Jaswinder Singh Maras, Sukanta Das, Sachin Sharma, Sukriti Sukriti, Jitendra kumar, Ashish Kumar Vyas, Dhananjay Kumar, Adil Bhat, Gaurav Yadav, Manish Chandra Choudhary, Shvetank Sharma, Guresh kumar, Chhagan Bihari, Nirupma Trehanpati, Rakhi Maiwall, Shiv Kumar Sarin

**Affiliations:** 1 Department of Molecular and Cellular Medicine, Institute of Liver and Biliary Sciences, New Delhi, 110070 India; 2 Department of Pathology, Institute of Liver and Biliary Sciences, New Delhi, 110070 India; 3 Department of Hepatology, Institute of Liver and Biliary Sciences, New Delhi, 110070 India

## Abstract

Severe alcoholic hepatitis (SAH) is associated with iron accumulation in hepatocytes/macrophages. This possibly correlates with inflammation and stress but the exact mechanism still remains obscure. To understand the role of iron and the mechanisms of systemic iron-overload, a transcriptomic study of liver and Peripheral Blood -Mononuclear-Cells (PBMCs) was undertaken in SAH patients, with and without hepatic iron-overload. Our results show that iron-overload in hepatocytes/macrophages is due to an increased expression of iron-loading receptors and CD163 signaling cascade. Increase in labile iron pool induces expression of iron-loading, oxidative-stress and inflammatory genes along with expression of CD163 and ADAM17. Increased liver iron correlated with circulatory iron, TNF-α, macrophage activation (sCD163) and peroxide-stress in CD163^+^macrophages in patients who were iron-overloaded and died. Circulatory TNF-α and sCD163 levels were associated with poor outcome. Temporal iron/Fenton stress induced in healthy monocyte-derived-macrophage (MDM)/Tohoku-Hospital-Pediatrics-1(THP1) cells showed higher expression of iron-regulatory, inflammatory and oxidative-stress genes. These genes could be suppressed by iron-chelation. These results suggest that iron mediates inflammation through ADAM17 induction, resulting in macrophage activation and increased shedding of TNF-α and sCD163. These events could be inhibited with iron chelation or with ADAM17-blockade, postulating a therapeutic strategy for SAH patients with iron overload.

## Introduction

Severe alcoholic hepatitis (SAH) is a serious form of alcoholic liver disease, attended with high morbidity and short-term mortality^1^. Activation of monocytes and macrophages induces a systemic inflammatory response^2,3^ and mediates progression of alcoholic hepatitis^2,4^. Spontaneous activation of inflammatory cascade in macrophages is due to autocrine TNF-α signaling,^5,6^ which in turn is under the regulation of ADAM (A Disintegrins and Metalloproteinase) metallopeptidase domain 17 (ADAM17)^7–9^. ADAM17 is involved in the process of ‘shedding’ which involves cleavage and release of the soluble ectodomain fraction of many membrane-bound pro-proteins including pro-TNF-α and CD163 receptor^10,11^. CD163 is a scavenger receptor for hemoglobin-haptoglobin (He-Hp) complex^12^ and soluble CD163 (sCD163) is its shedded ectodomain fraction^12^. Increase in CD163 expression on macrophages has been seen in inflammatory conditions including SAH^13^. Patients with advanced liver diseases exhibit higher CD163 expression on hepatic macrophages^14^ and have higher sCD163 levels^15,16^. Heavy alcohol abuse results in a vicious cycle of progressive oxidative stress and inflammation in the liver and circulating blood in SAH patients^17^. In this inflammation rich environment, CD163 promotes an anti-inflammatory response^12^ and regulates the circulatory/ hepatic levels of heme and its metabolites; bilirubin and iron^12^. In an earlier study, we have shown increased expression of CD163 on the circulating monocytes and dysregulated iron homeostasis was found to be associated with high mortality in alcoholic acute-on-chronic liver failure (ACLF) patients^13^.

In SAH patients, iron accumulates not only in hepatocytes but also in macrophages^18,19^. In liver, the iron load produces cellular stress and accelerates the generation of reactive oxygen species (ROS) and lipid-peroxidation products leading to cellular injury and death^20^. In circulation, active iron (Fe^2+^) is converted into inert-iron (Fe^3+^) and is stored in hepatocytes, macrophages and ferritin as a result of Fenton reaction (Fe^2+^+H_2_O_2_→Fe^3+^+HO^•^+OH^−^), (Fe^3+^+H_2_O_2_→Fe^2+^+HO_2_^•^+H^+^)^21^. Excessive Fenton reaction may lead to oxidative stress, inflammation, and organ dysfunction^22^. It is known that systemic iron overload correlates with cellular expression of CD163 and inflammation^23^. However, the underlying regulatory mechanisms which link CD163, iron stress and inflammation in SAH are not well understood. We hypothesized that by using a transcriptomic approach on the liver tissue and PBMCs of SAH patients, we could identify target genes and mechanisms linked to systemic iron-overload, oxidative stress and inflammation. To specifically study the role of iron, we studied the gene expression profile of liver and PBMCs of SAH patients with or without iron overload and correlated the observations with severity of liver disease and patient outcomes. We also investigated the underlying mechanisms related to increase in TNF-α and sCD163 levels in such patients and relevance of reduction of iron load on the inflammatory signals and pathways.

## Results

### Baseline character

RNA Seq was performed initially in a derivative cohort of SAH patients with iron load (SAH-IO; Scheuer-grade ≥1+, Group A: n = 5) and SAH patients with no-iron load (SAH-NIO; Group B: n = 10). One sample was excluded from the last group due to poor liver RNA quality (RIN < 7). The levels of serum creatinine, bilirubin, INR, total leucocyte count were significantly elevated in Group A patients as compared to other groups. Serum iron, ferritin, sCD163, TNF-α and severity indices were similarly higher in Group A (Table 1).

**Table 1:** Demographic profile of the study cohort.

Parameters	Discovery cohort			Validation cohort							
	(A)	(B)	(A) vs (B)	(C)	(D)	(E)	(C) vs (D)	(C) vs (E)	(F)	(G)	(F) vs. (G)
	Gr.A: SAH-IO(n = 5)	Gr.B:SAH-NIO(n = 9)	p-value	SAH (n = 100)	Alcoholic Cirrhosis (n = 20)	Healthy controls (n = 20)	p-value	p-value	SAH Non-survivors(n = 56)	SAH Survivors (n = 44)	p-value
M:F	5:00	9:01	—	92:08:00	18:02	16:04	—	—	51:05:00	41:03:00	—
Age (yrs.)	41 (30–55)	40 (20–60)	—	43 (19–66)	35 (28–57)	33 (30–40)	—	—	43 (38–57)	40 (28–65)	—
TLC (×10^3^/mm^3^)	9.5 (6.0–18)	13 (8.9–20)	0.0012	9.5 (3.9–29.8)	8.3 (3.1–20)	9.6 (4–11)	0.022	—	10.7 (5.0–28)	11.5 (3.9–23.7)	0.2776
Platelet (×10^3^/mm^3^)	95 (48–154)	135 (110–180)	0.011	126 (45–260)	171 (60–300)	155 (135–400)	0.015	0.031	100 (45–240)	120 (50–260)	0.0852
Bilirubin (mg/dL)	29 (15–42)	15 (6.8–20)	<0.01	23.30 (6.8–43)	1.5 (0.9–34)	0.9 (0.5–1.5)	<0.01	<0.01	26 (9–43)	21 (6.8–27)	0.0026
INR	2.8 (1.9–4.1)	2.1 (1.5–2.5)	<0.01	2.5 (1.1–4.6)	1.3 (1–2.5)	0.9 (0.8–1.2)	<0.01	<0.01	2.3 (1.5–4.6)	2.2 (1.1–4.0)	0.0573
ALT (IU/L)	110 (75–252)	65 (35–150)	0.004	69 (22–339)	60 (33–300)	27 (18–45)	0.052	0.021	96 (25–339)	51 (22–250)	0.0017
Albumin (gm/dL)	2.3 (2.1–3.7)	2.6 (2.1–4.5)	0.022	2.1 (1.8–4.9)	2.6 (2.9–4.6)	4 (3.4–5.4)	0.048	0.0112	2.5 (2.0–4.9)	2.6 (1.8–3.1)	0.8237
Serum creatinine (mg/dL)	2.2 (1.5–5.8)	0.8 (0.4–2.5)	<0.001	2.4 (0.4–6.2)	0.75 (0.1–0.9)	0.7 (0.6–0.9)	<0.01	<0.01	2.3 (0.7–6.2)	0.6 (0.4–2.9)	< 0.01
HE at baseline	(80%),3 (0–4)	(20%), 1 (0–3)	—	(85%), 2 (0–4)	—	—	—	—	(72%),2 (0–4)	(25%), 0(0–3)	0.001
Na (mEq/L)	130 (116–138)	131 (120–140)	0.12	131 (115–149)	133 (132–141)	136 (135–140)	0.08	0.048	130 (115–149)	131 (124–139)	0.2784
Serum Iron (ug/ml)	**1100** (**750–2512)**	**295** (**150–680)**	**0.0001**	**446.2** (**109.4–2644)**	**344.1** (**106.3–862.5)**	**114.1** (**40.63–243.8)**	**0.04**	**0.012**	**856.9** (**109.4–2644)**	**312.5** (**109.4–1134)**	** < 0.001**
Serum Ferritin (ug/ml)	754 (390–1123)	450 (59–1100)	0.01	602.5 (158–1318)	104 (59.44–745.1)	113.7 (39.83–554.4)	0.01	0.01	632.1 (158–1318)	461.9 (61.7–1157)	0.0156
Soluble CD163 (ng/ml)	**1855** (**1295–2216)**	**500** (**200–800)**	**<0.01**	**1150** (**220–2216)**	**550** (**370–760)**	**495** (**125–610)**	**<0.01**	**<0.01**	**1583** (**595–2216)**	**590** (**180–1235)**	** < 0.01**
Hemoglobin-Haptoglobin complex (ng/ml)	1.8 (1.4–5)	6 (1.5–20)	<0.01	3 (1.4–23.2)	13.25 (4–26)	13.75 (8–25)	<0.01	<0.01	2 (1.4–20)	4.5 (1.5–23.2)	0.01
TNF-α (picogram/ml)	**100** (**85–118)**	**37** (**28–70)**	**<0.001**	**68.5** (**28–126)**	**16** (**4.6–45)**	**8.5** (**3–27)**	**<0.001**	**<0.001**	**80** (**28–118)**	**39.5** (**28–86)**	** < 0.001**
DF score	125 (70–243)	87 (30–150)	<0.01	103 (30–243)	16.5 (5.2–60)	—	<0.01	—	125 (47.6–243)	87 (30–191)	0.0137
CTP-Score	14 (10–15)	12 (4–13)	<0.01	12 (4–14)	8 (5–13)	—	<0.01	—	14 (9–14)	13 (4–14)	0.0429
MELD-Score	35 (27–40)	25 (15–30)	<0.01	24 (12–40)	12 (8–17)	—	<0.01	—	30 (12–40)	29 (15–36)	0.043
SOFA-Score	10 (8–12)	5 (4–7)	<0.01	7 (4–12)	2 (1–2)	—	<0.01	—	8 (4–12)	5 (4–8)	0.001
Median Survival	—	—	—	—	—	—	—	—	24 (7–60)	130 (110–330)	0.0001

Demographic profile of the study cohort (discovery and validation cohort), the values are expressed in medians and range unless stated otherwise, p-value < 0.05 is significant.

### Validation phase

Circulatory protein levels were validated in a separate cohort of 100 SAH patients (92 males), 20 alcoholic cirrhotic (AC) (18 males, 90%) and 20 healthy controls (HC) (16-males, 80%). Total leucocyte counts, iron, ferritin, sCD163, TNF-α levels were significantly increased in SAH patients (Table 1). All the patients were followed for 3 months or until death.

#### SAH patients with iron overload show a distinct hepatic transcriptomic profile

**Figure 1:**
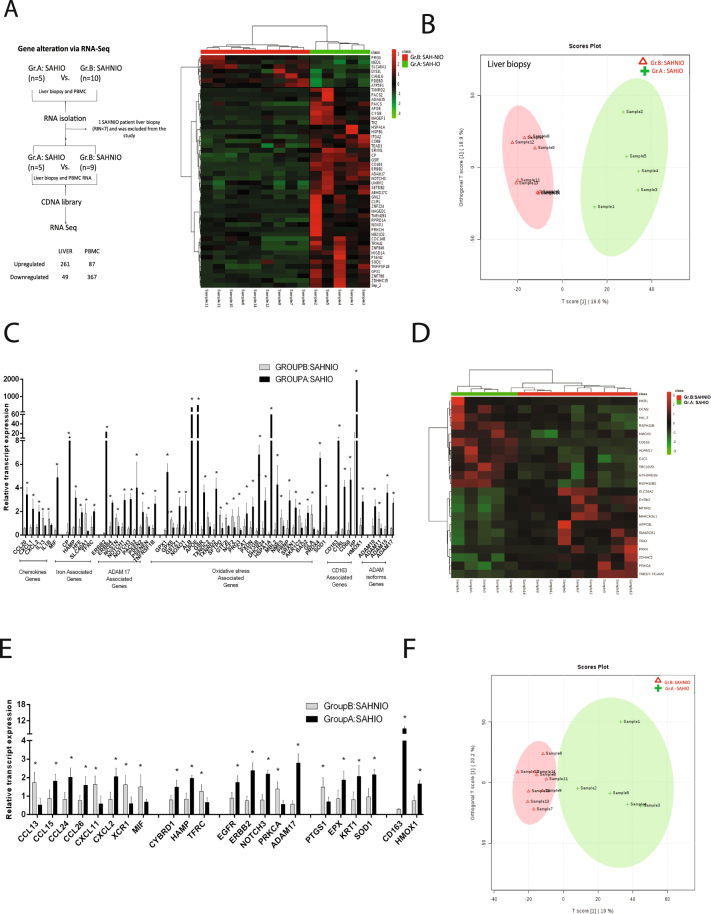
Hepatic and PBMC transcriptome in distinct in Group A: SAH-IO. (**A**) Workflow and summary of the RNA-Seq performed in Group A: SAH-IO (n = 5) vs. Group B: SAH-NIO (n = 9) shown in left-panel and supervised clustering of hepatic transcriptome for genes with p < 0.05, Fold Change > 1.5in Group A: SAH-IO (n = 5) vs. Group B: SAH-NIO (n = 9) of discovery cohort shown in right panel. See also Supplementary Table-1. (**B**) Orthogonal Projections to Latent Structures Discriminant Analysis (OPLS-DA) of hepatic transcriptome in Group A and Group B. See also supplementary table-3. (**C**) Selection of differentially regulated genes linked to iron-regulation, oxidative-stress and inflammation modules ([*] = p < 0.05) in the Hepatic transcriptome of Group A: SAH-IO vs. Group B: SAHNIO. (**D**) Supervised clustering of PBMC transcriptome for genes with p < 0.05, Fold Change >1.5 in Group A: SAH-IO (n = 5) vs. Group B: SAH-NIO (n = 9) of discovery cohort. See also supplementary table-5. (**E**) Orthogonal Projections to Latent Structures Discriminant Analysis (OPLS-DA) of PBMC transcriptome in Group A: SAH-IO and Group B: SAHNIO. See also supplementary table-6. (**F**) Selection of differentially regulated genes linked to iron-regulation, oxidative-stress and inflammation modules ([*] = p < 0.05) in the PBMC transcriptome of Group A: SAH-IO vs. Group B: SAHNIO.

Discovery phase: Gene expressions linked to systemic iron-overload were studied in the liver tissue and PBMCs of Group A and B patients (Fig. 1A, left-panel). Liver transcriptome identified 19,807 protein coding genes of which 261 were upregulated and 49 downregulated (≥1.5 folds, p < 0.05) (Fig. 1A, right-panel, Supplementary Table 1). In Group A patients, the upregulated genes were linked to oxidative/oxido-reductase activity, ROS detoxification, proteolytic cleavage of receptors (Notch, NICD), and TNF-α signaling (GO/KEGG, Supplementary Table 2). The downregulated genes were linked with IL-2 biosynthesis, nitric-oxide synthase, cytokines, glutathione peroxidase and NF-kappa B signaling (Supplementary Table 2). Orthogonal partial least square discriminating analysis (OPLS-DA) documented a clear distinction between Group A and B (Fig. 1B) and identified 268 genes with VIP (Variable important in projection) score >1 (Supplementary Table 3) in Group A. These genes were linked to iron-regulation (*CP, HAMP, and TFRC*), iron-processing by macrophage (*CD163, CD68, HP, and HMOX1*), oxidative-stress/antioxidants (*NOXO1, HMOX1, GSR, and SOD1*), chemokine (*CCL20, CXCL1*) and metalloproteases (*ADAM10, ADAM17, NOTCH1*, and others) (Fig. 1C, Supplementary Table 4).

#### SAH patients with iron overload exhibit a distinct PBMC transcriptomic profile

PBMC transcriptome identified 19,807 protein coding genes, 87 of which were upregulated and 367 were downregulated (p < 0.05, folds >1.5) in Group A than B (Fig. 1D, Supplementary Table 5). GO and pathway analysis highlighted a significant increase in cellular iron homeostasis, ROS/hydrogen peroxide metabolism, chemokine activity, iron uptake/transport in Group A (Supplementary Table 6). Genes linked to cytokine synthesis, interleukin-17 production, apoptosis, NF-kappa B were significantly reduced in Group A (Supplementary Table 6). OPLS-DA clearly differentiated Group A from B (Fig. 1E) and identified 620-genes (VIP > 1; Supplementary Table 7) majorly associated to chemokines (*CXCL2, CCL24* and others), iron-regulation (*HAMP, CYBRD1*), macrophage iron (*HMOX1, CD163*), oxidative/antioxidant stress (*SOD1, KRT1*) and metalloproteases (*ADAM17, ERBB2* and others) (Fig. 1F, Supplementary Table 4).

These observations suggest a direct association between iron-overload and induction of genes related to iron loading, oxidative-stress, inflammation and metalloproteases in the liver and PBMC of SAH patients.

#### SAH patients show increased CD163 and ADAM17 expression

**Figure 2:**
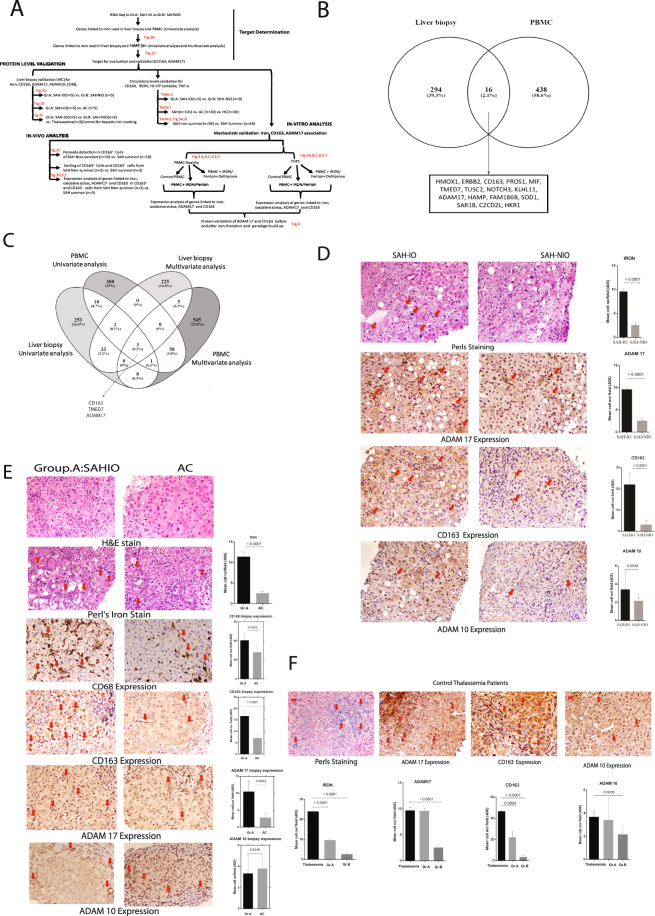
Increased CD163 and ADAM17 expression is associated to iron-overload in SAH. (**A**) Workflow and summary of the experiments performed and number of samples used in target determination, and validation experiments including the *In-vivo* and *In-vitro* analysis. (**B**) Venn-diagram for unique and shared genes (p < 0.05, fold change >1.5) in the liver and PBMC transcriptome of Group A: SAH-IO.16 shared genes in the liver and PBMC were associated to cellular iron ion homeostasis, cytokine receptor binding and notch signaling. (**C**) Venn-diagram for unique and shared genes identified in the univariate and multivariate projection analysis for the liver and PBMC transcriptome of Group A: SAH-IO documenting CD163, ADAM17 and TMED7 significantly associated to systemic iron overload. (**D**) Immuno-histochemistry (IHC) for iron deposition (Perl’s-staining) and expression of ADAM17, CD163, ADAM10 in Group A: SAH-IO (n = 5) vs. Group B: SAH-NIO (n = 5). [For all IHC analysis relative quantization of positively stained cells are expressed as mean number of positive cells/10 high power field (40×)]. (**E**) Perl’s staining and expression of CD68, ADAM17, CD163 and ADAM10 in Group A: SAH-IO (n = 5) vs. AC (n = 5). (**F**) Perl’s staining, and expression of ADAM17, CD163 and ADAM10 in Thalassemia (n = 3) patients compared to Group A and B.

Validation-phase: Systemic iron-overload associated genes identified in the liver and PBMC transcriptome were validated in the liver biopsy and plasma of study groups, and by *in-vivo* and *in-vitro* analyses (Fig. 2A). A total of 16 genes were dysregulated in the liver and PBMC transcriptome of Group A versus B. These genes were linked to cellular iron homeostasis, response to hydrogen peroxide and notch receptor signaling (C-score > 2; Fig. 2B). Of these only 3 genes (*CD163, ADAM17, and TMED7*) were significantly linked to systemic iron-overload based on multivariate projection model analyses (Fig. 2C, Supplementary Table 8). The expression of CD163 and ADAM17 were validated on the representative liver sections of Group A and B patients, while *TMED7* was excluded from analysis due to its variable expression (Supplementary Table 8). The liver section of Group A showed a significant increase in ADAM17 and CD163 expression in the sinusoidal spaces with no alteration in ADAM10 expression (p < 0.05; Fig. 2D). To establish a relationship between macrophage iron-overload and expression of CD163 and ADAM 17, the liver of Group A patients was compared with AC patients. In Group A, the expression of CD68, CD163 and ADAM17 was higher (p < 0.05, Fig. 2E).

This observation was further evaluated in thalassemia patients with iron-overload (3 + Scheuer grade). Expression of ADAM17 and CD163 was increased (p < 0.05) in the hepatic tissue (cytoplasmic) and macrophages (sinusoidal spaces) in thalassemia patient’s liver tissue (Fig. 2F). These results clearly support a role of iron load in modulation of ADAM17 and CD163 expression in SAH patients.

#### Serum iron, sCD163, He-Hp complex and TNF-Alpha correlate with outcome of SAH patients

**Figure 3:**
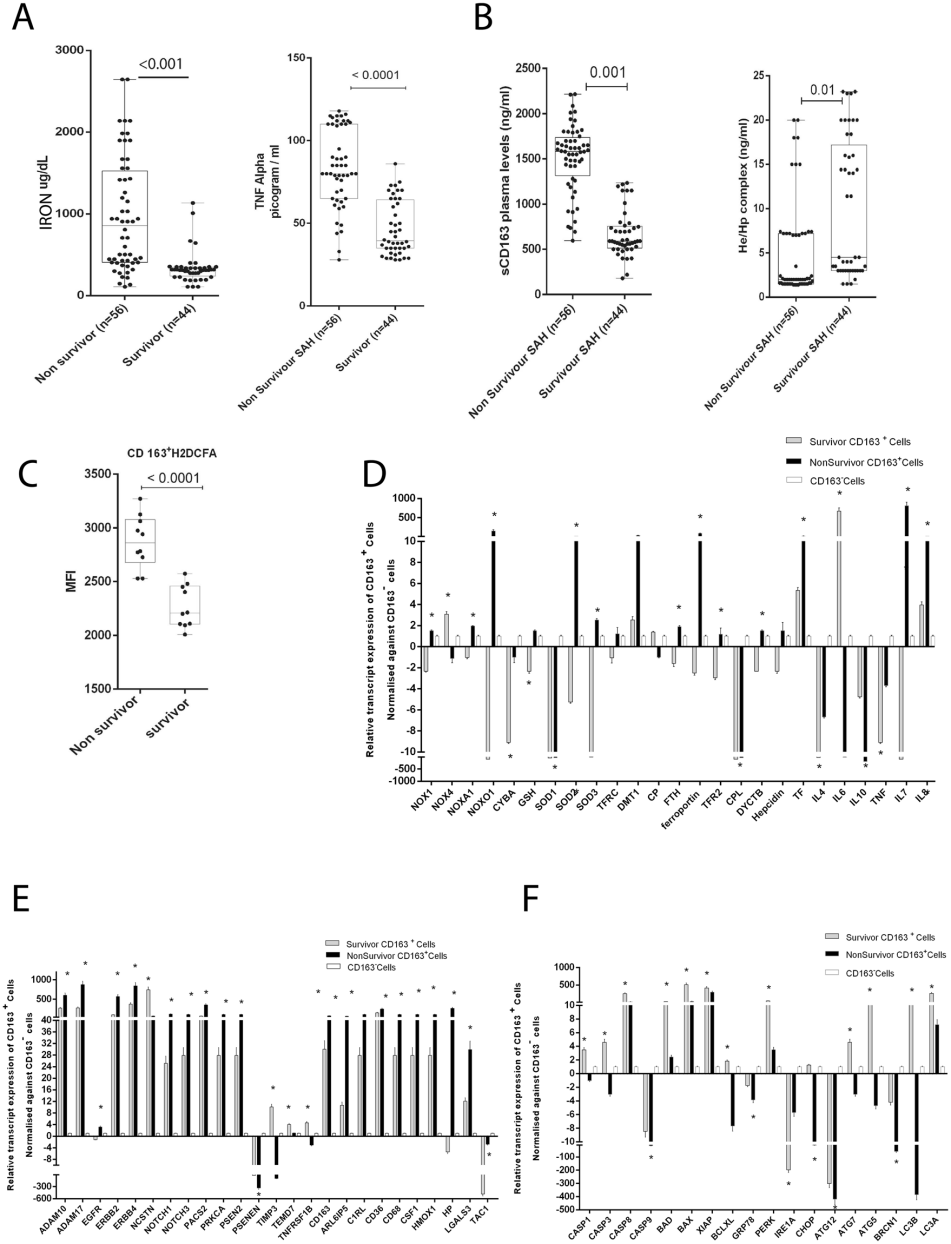
Increase in circulatory inflammation and alteration of gene expression in CD163^+^macrophages of non-survivors in SAH. (**A**) Circulatory iron in Non-survivors (n = 56;856.9[109.4-2744]ug/mL) vs. Survivors (n = 44; 312.5[109.4-1134] ug/mL) [p ≤ 0.01]. TNF-α levels in Non-survivors (80(28-118) pg/mL) vs. Survivors (39.5(28-86) pg/mL) [p ≤ 0.01]. (**B**) sCD163 levels in Non-survivors(1583[595-2216]ng/ml) vs. Survivors(590[180-1235]ng/ml) [p ≤ 0.01].He-Hp complex levels in Non-survivors(2[1.4–20]ng/ml) vs. Survivors(4.5[1.5–23]ng/ml) [p ≤ 0.05]. (**C**) Intracellular peroxide levels of CD163^+^ cells in (n = 10) Non-survivors 2750[2500–3300] MFI vs. (n = 10) Survivors 2250[2000–2550] MFI (p < 0.01). (**D**) Relative expression of oxidative-stress, inflammation and iron-regulatory genes in CD163^+^ cells of Non-survivors (n = 3) vs. Survivors (n = 3) ([*] = p < 0.05). (**E**) Relative expression of ADAM17, CD163 and linked genes in CD163^+^ cells of Non-survivors vs. Survivors ([*] = p < 0.05). (**F**) Relative expression of apoptosis, ER-stress and autophagy genes in CD163^+^ cells of Non-survivors vs. Survivors ([*] = p < 0.05).

In our study, the levels of circulating iron, sCD163, TNF-α directly correlated with hepatic encephalopathy and inversely with disease severity scores and survival (r^2^ > −0.3, p < 0.05, Supplementary Table 9). The levels of serum iron, ferritin, sCD163, TNF- α were significantly higher in SAH patients who did not survive (Fig. 3A,B, Table 1). In fact, sCD163 and TNF-α levels had high predictability for mortality (AUROC >0.90) (Table 2). Univariate and multivariate Cox regression analyses identified a significant association of sCD163, TNF-α, and hepatic encephalopathy (≥grade 2) with mortality (HR > 1.5, p < 0.05) in SAH patients (Table 2). Since ADAM17 expression is associated with ectodomain shedding of TNF-α and sCD163^7^, our results suggest that the increase in sCD163 and TNF-α is associated with iron-overload and activation of ADAM17 in SAH patients.

**Table 2:** AUROC, Univariate and Multivariate COX Regression Analysis, C-index and Somer’s D.

AUROC for Non survivor prediction					Univariate analysis					Multivariate analysis					C-index	Somers D
Variable (s)	Area	P-value	95% CI		Wald	P-value	HR	95.0% CI(HR)		Wald	P-value	HR	95.0% CI (HR)			
			LB	UB				LB	UB				LB	UB		
sCD163	0.96	0.0001*	0.92	0.99	53.60	0.0001*	17.19	7.48	39.52	9.91	0.0016*	6.15	1.98	19.06	0.71	0.41
He-Hp	0.23	0.001*	0.14	0.33	10.53	0.001*	0.52	0.37	0.73	1.68	0.20	0.80	0.58	1.12	—	—
Ferritin	0.52	0.71	0.41	0.64	0.16	0.69	0.95	0.73	1.24	—	—	—	—	—	—	—
Iron	0.81	0.0001*	0.72	0.90	18.99	0.001*	2.09	1.53	2.85	0.00	0.98	1.00	0.70	1.45	—	—
TNF-α	0.90	0.0001*	0.84	0.96	29.41	0.001*	6.64	3.35	13.18	4.59	0.0320*	3.40	1.11	10.43	0.61	0.21
HE (grade >2)	0.70	0.004*	0.56	0.77	22.54	0.001*	1.72	1.37	2.15	10.07	0.0015*	1.62	1.20	2.18	0.63	0.32
DF	0.69	0.001*	0.59	0.79	6.19	0.013*	1.08	1.02	1.15	0.07	0.79	1.01	0.93	1.10	—	—
MELD	0.65	0.013*	0.54	0.75	7.76	0.005*	1.05	1.01	1.08	0.01	0.92	1.00	0.96	1.05	—	—
SOFA	0.69	0.001*	0.59	0.79	18.08	0.001*	1.34	1.17	1.54	2.25	0.13	1.16	0.96	1.41	—	—

Area under the receiver operating characteristic curve (AUROC) followed by Univariate, Multivariate COX Regression, C-index and Somers D Analysis of the most significant parameters for the determination of mortality in Severe alcoholic hepatitis, p < 0.05 is significant.

#### CD163+ macrophages of SAH non-survivors exhibit iron linked oxidative stress and inflammation (*In-vivo* analysis)

Recently our group showed that CD163^+ve^ macrophages contribute to the circulating iron pool^12,13^. Patients of SAH who do not survive, showed higher CD163^+ve^ macrophages (Supplementary Fig. 2) and higher levels of intracellular peroxide stress (p < 0.05, Fig. 3C). CD163^+ve^ macrophages of non-survivors showed increased expression (p < 0.05, FC > 1.5) of iron-import and regulation (*TFRC, TFR2, Ferroportin, Hepcidin*), inflammatory-cytokines (*IL7, IL8*), oxidative stress (*NOX1, NOXO1*), *ADAM17* and linked (*EGFR, Notch1* and others), *CD163* and linked (*HMOX1, HP* and others) genes (Fig. 3D,E). Caspase dependent cell death and autophagy genes were relatively higher in survivors (Fig. 3F). These results suggest a prominent dysregulation of iron homeostasis, oxidative stress, inflammatory cytokines, induction of *ADAM17* and *CD163* signaling cascade in CD163^+^ macrophages of non-survivors.

#### Iron chelator reverses the effect of Iron and Fenton in healthy Monocyte Derived Macrophages (MDMs)

**Figure 4:**
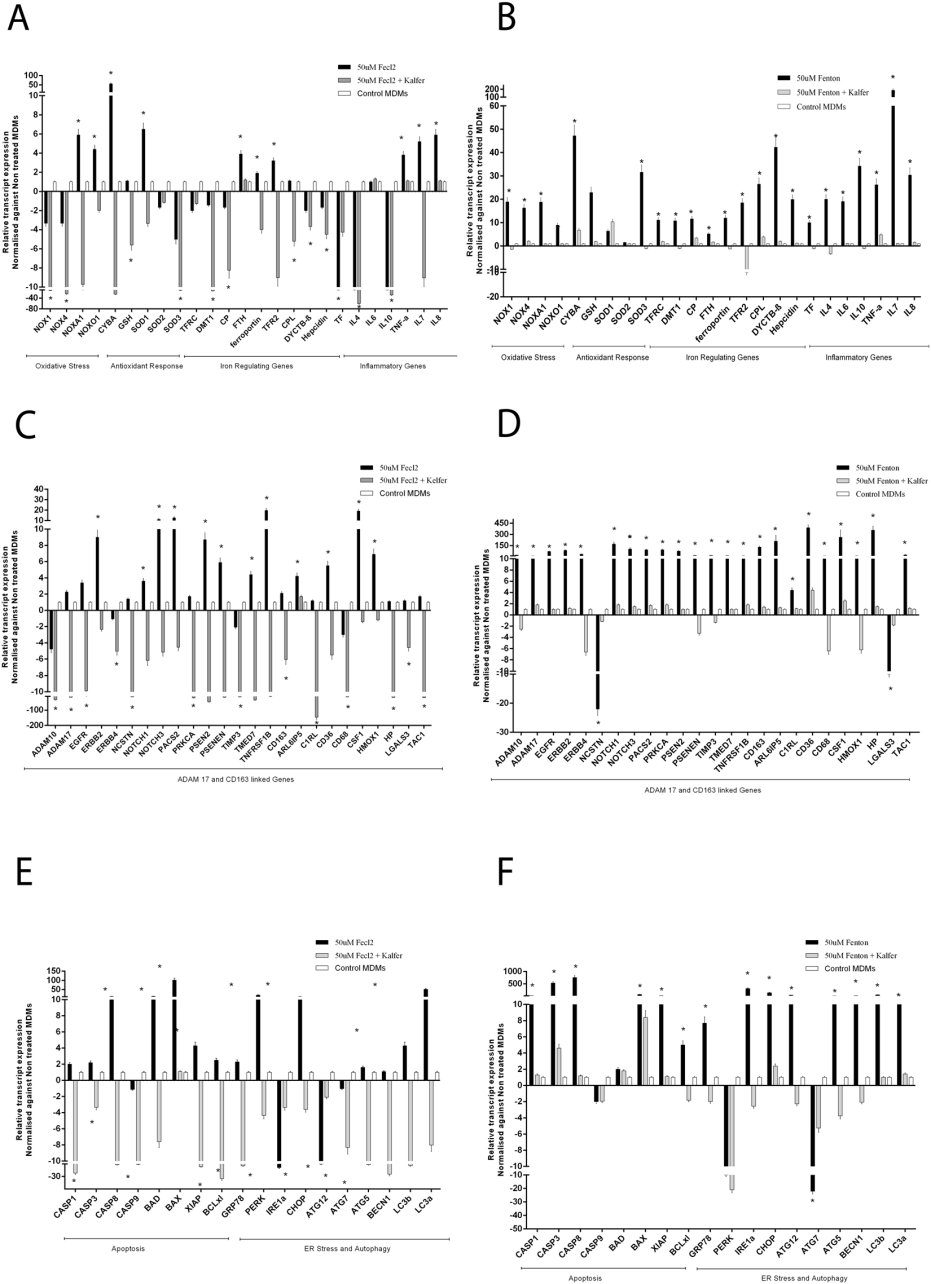
Effect of Iron or Fenton can be reversed by iron chelation in healthy MDMs cells. (**A**) Relative expression of oxidative-stress, inflammation and iron-regulatory genes in MDMs treated with 50 uM-Fecl2 in presence or absence of 150 uM Kelfer. Genes significantly altered are labelled as ([*] = p < 0.05). (**B**) Relative expression of oxidative-stress, inflammation and iron-regulatory in MDMs treated with 50 uM-Fenton in presence or absence of 150 uM Kelfer ([*] = p < 0.05). (**C**) Relative expression of ADAM17, CD163 and associated genes in MDMs treated with 50 uM-Fecl2 in presence or absence of 150 uM Kelfer ([*] = p < 0.05). (**D**) Relative expression of ADAM17, CD163 and associated genes in MDMs treated with 50 uM-Fenton reagent in presence or absence of 150 uM Kelfer ([*] = p < 0.05). (**E**) Relative expression of apoptosis, ER stress and autophagy genes in MDMs treated with 50 uM-Fecl2 in presence or absence of 150 uM Kelfer ([*] = p < 0.05). (**F**) Relative expression of apoptosis, ER stress and autophagy genes in MDMs treated with 50 uM-Fenton reagent in presence or absence of 150 uM Kelfer ([*] = p < 0.05).

To understand the role of circulating iron or Fenton in the induction of metalloproteases (ADAM17), inflammation and stress, MDMs from healthy controls were treated with iron/Fenton reagent at increasing concentrations in presence or absence of iron chelator, deferiprone. Fenton was more prominent in stimulation of genes related to oxidative-response, iron-regulation, matalloproteases, ER-stress and autophagy as compared to iron alone (Supplementary Fig. 3). Deferiprone treatment could significantly (p < 0.05) ameliorate expression of genes linked to superoxide/antioxidant response, iron-homeostasis, and inflammatory-cytokines (*TNF-α, IL-7, IL-8*), (Fig. 4A,B). Iron alone failed to induce other metalloproteases (*ADAM10*) but was able to significantly induce ADAM17 and genes associated to macrophage iron production (*CD163, HMOX1*) which got neutralized by deferiprone (Fig. 4C). Fenton on the other hand, induced expression of both *ADAM10, ADAM17* and macrophage iron processing genes (*CD163, HP, HMOX1*), which also got reduced (p < 0.05) under deferiprone (Fig. 4D). Iron/Fenton significantly increased apoptosis, ER-stress and autophagy genes which got ameliorated (p < 0.05) under deferiprone (Fig. 4F,E). These results clearly suggest that iron invariably induces *ADAM17* and genes linked to oxidative-stress, iron-regulation, apoptosis which get more pronounced in presence of peroxide stress but can be neutralized if iron is chelated.

#### Iron chelation can reverse effect of iron or Fenton reagent in THP-1 derived macrophages

**Figure 5:**
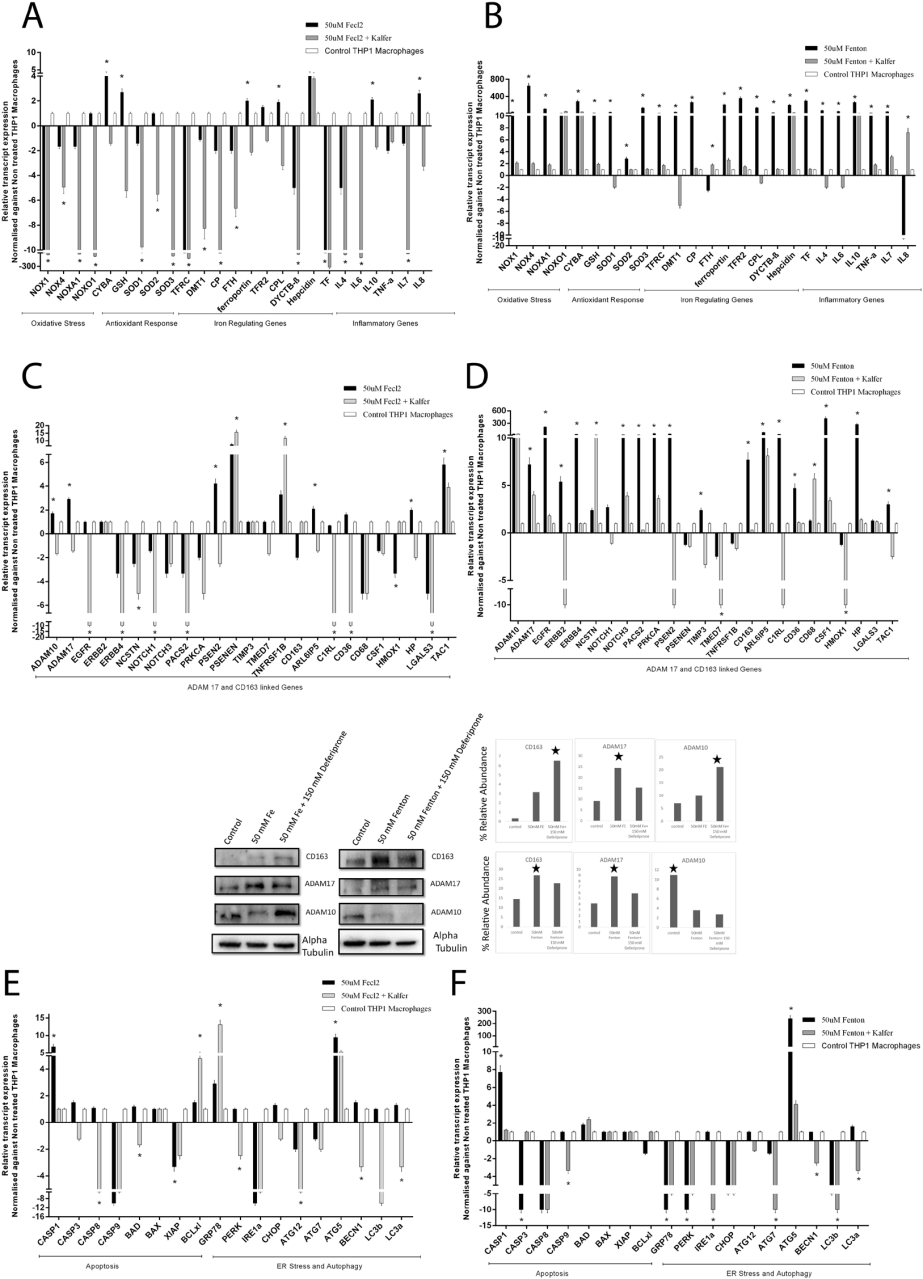
Effect of Iron or Fenton can be reversed by iron chelation in THP1 derived macrophages. (**A**) Relative expression of oxidative-stress, inflammation and iron-regulation genes in THP1-macrophages treated with 50 uM-Fecl2 in presence or absence of 150-uM Kelfer ([*] = p < 0.05). (**B**) Relative expression of oxidative-stress, inflammation and iron-regulation genes in THP1-macrophages treated with 50 uM-Fenton in presence or absence of 150 uM Kelfer ([*] = p < 0.05). (**C**) Relative expression of ADAM17, CD163 and associated genes in THP1-macrophages treated with 50 uM-Fecl2 in presence or absence of 150 uM Kelfer. The lower panel documents protein expression validation of ADAM17, CD163 and ADAM10 in the same condition ([*] = p < 0.05). (**D**) Relative expression of ADAM17, CD163 and associated genes in THP1-macrophages treated with 50 uM-Fenton in presence or absence of 150 uM Kelfer. The lower panel documents protein expression validation of ADAM17, CD163 and ADAM10 in the same condition ([*] = p < 0.05). (**E**) Relative expression of apoptosis, ER-stress and autophagy genes in THP1-macrophages treated with 50 uM-Fecl2 in presence or absence of 150 uM Kelfer ([*] = p < 0.05). (**F**) Relative expression of apoptosis, ER-stress and autophagy genes in THP1-macrophages treated with 50 uM-Fenton in presence or absence of 150 uM Kelfer ([*] = p < 0.05).

The result of THP-1 stimulation assay (induction by Iron/Fenton and neutralization by deferiprone) was similar to the MDMs stimulation assay (Supplementary Fig. 3). In brief, 50 mM iron was able to (FC > 1.5, p < 0.05) induce genes linked to superoxide generation (*CYBA*), antioxidants (*GSH*), iron-homeostasis (*FERROPORTIN, HEPCIDIN*), and inflammation (*IL-10, IL-8*), which were reduced under deferiprone (Fig. 5A). Again, iron in the presence of peroxide stress (Fenton) was more potent in the induction of oxidative/superoxide response, iron-homeostasis, inflammatory-cytokines, and deferiprone could inhibit (p < 0.05) their expression (Fig. 5B). Both iron and Fenton stimulated expression of *ADAM17, CD163* and linked genes (p < 0.05, FC >1.5) which were significantly reduced under deferiprone (Fig. 5C,D). Iron/Fenton stress significantly increased the protein levels of *ADAM17* (Fig. 5C,D lower panel) which got reduced with chelation. Iron chelation resulted in higher cellular expression of CD163 and ADAM 10 (Fig. 5C,D) suggesting that expression of ADAM17, ADAM10 and CD163 are under the influence of iron regulation. Iron/Fenton stress also increased *CASP1* and *ATG5* expression which was suppressed with Deferiprone treatment (Fig. 5E,F). These observations suggest that in THP-1 macrophages, iron/Fenton significantly increases iron loading, oxidative stress, pyroptosis and autophagy. Further, the cellular level of CD163 is regulated by iron-load via activation of ADAM17 in both MDMs and THP-1 derived macrophages.

#### ADAM 17 inhibition decreases TNF-α and sCD163 expression in THP1 macrophages

**Figure 6:**
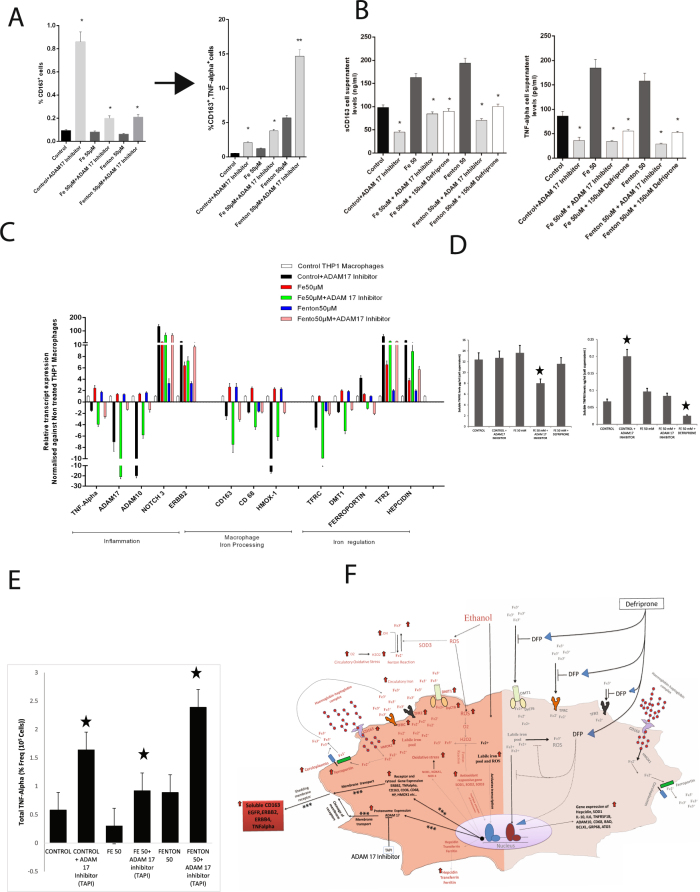
Causality for iron-mediated activation of ADAM17, sCD163 and inflammation. (**A**) Percentage frequency of CD163 + and CD163^+^, TNF-α^+^ in THP1 cells (n = 3) stimulated by Iron/Fenton (50 uM) under ADAM17 inhibition. (*p < 0.05) (**B**) Cell supernatant level of sCD163 and TNF-α + inTHP1 cells (n = 3) stimulated by Iron/Fenton (50 uM) under ADAM17 inhibition. (*p < 0.05) (**C**) Expression of genes linked to inflammation, iron regulation and macrophage iron regulation inTHP1 cells stimulated by Iron/Fenton (50 uM) under ADAM17 inhibition. (**D**) Soluble TNFR1 and TNFR2 levels in the cell supernatant of THP1 macrophages under iron/Fenton stress treated with or without ADAM17 inhibitor (TAPI-1) (*p < 0.05). (**E**) Percentage frequency of Total TNF-α positive THP1 macrophages under iron/Fenton stress treated with or without ADAM17 inhibitor (TAPI-1) (*p < 0.05). (**F**) Paradigm for iron-stress, metalloprotease activation and induction of inflammatory cascade in SAH patients: Iron loading in macrophage is by increased expression of iron import receptor (TFR2, DMT1) and secondary iron load contributors (CD163, HMOX1). Iron overload induces peroxide stress and expression of genes linked to iron homeostasis (hepcidin, ferritin, transferrin), oxidative stress (NOX1, NOXA1) and inflammation (TNF-α, ADAM17). Increased expression of ADAM17 increases sCD163 and TNF-α in circulation, which correlates to mortality in SAH patients. Iron chelation and ADAM17 inhibition remarkably neutralizes these alteration and could be used as attractive therapeutic option in such patients. Pathways marked with [***] is significantly increased in SAH patients particularly in those with iron-load.

ADAM17 inhibition was performed on THP1 macrophages under Iron/Fenton stress to establish the causality between iron-mediated activation of ADAM17, sCD163 and inflammation. Cellular expression of CD163 and TNF-α were increased, while sCD163 and TNF-α levels in the cell-supernatant were decreased (p < 0.05) under ADAM17 inhibition irrespective of iron/Fenton stress (Fig. 6A,B). Expression of inflammatory genes was also lower and iron regulatory genes were higher under ADAM17 inhibition irrespective of iron/Fenton stress (Fig. 6C). Inhibition of ADAM17 increased the CD163 expression (Supplementary Figure 4). Similar observations were seen in response to temporal iron/Fenton stress (Supplementary Figure 5, 6, 7). The soluble level of TNF-R1 was unchanged while the levels of TNF-R2 were significantly increased under ADAM 17 (TAPI-1) inhibition (Fig. 6D) in the absence of iron stress. However, under iron stress, TNF-R1 levels were significantly lower in TAPI-1 treated cell supernatant (Fig. 6D). Our data clearly shows that TAPI-1 has a differential activity towards TNF-R1 and TNF-R2 in the presence or absence of iron. Since the activation of TNF-R1 is mainly involved in the activation of inflammation, use of TAPI-1 may prevent iron induced inflammation. ADAM 17 inhibition by TAPI-1 increased membrane TNF-α levels in the macrophages irrespective of iron/Fenton stress (Fig. 6E and Supplementary Fig. 8). These observations suggest that ADAM 17 inhibition modulates inflammation by inhibiting TNF-α shedding in macrophages.

## Discussion

Severe alcoholic hepatitis is accompanied by significant hepatocellular necrosis and in a proportion of patients, increased iron load in hepatocytes and macrophages^18^. Our results based on the RNA-Seq on liver tissue and PBMCs, clearly show that genes linked to iron-loading, oxidative-stress and inflammation, including expression of CD163, ADAM17 are over expressed in SAH patients with iron-overload and this plays a major role in progressive hepatic injury and correlates with non-survival. Furthermore, iron chelation prevented induction of ADAM17 expression and mediated an increase in expression of CD163, both at the gene and protein level. Expression of genes linked to inflammation and oxidative-stress were decreased with iron chelation providing an explanation that chelation prevents TNF-α and CD163 shedding and suppresses inflammation via reducing the expression of ADAM17. We validated these results in a large validation cohort and confirmed this by *in-vivo* and *in-vitro* studies.

In the present study, the increase in the liver iron in SAH was due to a marked increase in expression of transferrin receptor protein 1 (*TFRC*) and decrease in *FPN1* (ferroportin) which is consistent with our previous study^13^. Liver and PBMC transcriptome showed significant increase in secondary iron loading genes (*CD163, HP, HMOX1*), indicating that iron overload in SAH is via activation of CD163 signaling^12^. Iron-overload in liver/PBMC mediated iron dysregulation and significantly induced genes linked to oxidative/antioxidant activity, proteolytic cleavage of receptors, iron-homeostasis, ROS/hydrogen peroxide metabolism^18,24^. In SAH patients with iron-overload, pathways linked to TNF-α maturation and ROS detoxification were augmented. This indicates that iron accumulation promotes inflammation and oxidative damage^10,21^; dominantly by activation of TNF-α signaling.

Liver and PBMC transcriptome identified 16 genes linked to cellular iron homeostasis, notch receptor signaling and secondary cleavage of receptors. Univariate and multivariate projection analysis identified ADAM17 and CD163 as dominantly upregulated genes related to systemic iron-overloaded in SAH. Immuno-histochemistry validated a significant increase in ADAM17 and CD163 levels with minimal alteration of ADAM10 expression in the Kupffer cells (Fig. 2D). This suggests that in SAH, iron load may play a significant role in the induction of ADAM17 and CD163 expression^10^. This observation was further confirmed in thalassemia patients who have a high iron-load (Fig. 2F).

Increase in ADAM17 expression increases cleavage of CD163 receptor^7^. Livers of patients with iron-overload, showed significantly increased levels of CD163 and ADAM17, correlating with the increase in sCD163 and TNF-α levels^11^. This could be due to activation of ADAM17 by iron (Fe^++^) replacing zinc (zn^++^) from its catalytic domain. In our study, the circulatory levels of TNF-α, sCD163, iron and ferritin were significantly higher along with increase in ADAM17 expression in SAH patients who died as compared to those who survived. In addition, high sCD163, TNF-α levels and presence of hepatic encephalopathy were significantly associated with mortality in SAH.

Soluble CD163 works as an anti-inflammatory molecule while TNF-α is pro-inflammatory^7,10^. Iron accumulation in SAH patients is by iron import receptor or by processing of He-Hp complex and activation of HMOX1 in CD163^+^ macrophage^12^. We tried to correlate the iron disbalance and transcriptomic profile with survival in patients with SAH. In our study He-Hp complex was significantly downregulated in non-survivors with concomitant upregulation of *CD163, HMOX1, HP*, and *CD68* genes, suggesting a hyper-regulated CD163 signaling cascade in hepatic lineage macrophages. This increase in *CD68* expression on circulating macrophages could have many functions and warrants further studies. This observation was validated in PBMCs where there was an increase in CD163 receptor expression. SAH patients showed increase in the intracellular ROS/ peroxide pool, which serves as a precursor for Fenton reaction^22^. We have earlier shown that increase in CD163^+^ cells are associated with an increase in free labile iron pool^13^.

To understand the role of CD163^+^ macrophages in iron processing, oxidative-stress and ADAM17 induction, gene expression analysis was performed on purified CD163^+^ and CD163^-^ cells from SAH patients. A significant increase in oxidative-stress, antioxidant response and iron processing genes in response to the circulatory iron stress was observed in CD163^+^ macrophages of non-survivors. Result of our current study show that there is an increase in labile iron pool in CD163^+^ macrophages of non-survivors^13^ and this might be due to increase activation of DMT1 receptor and secondary iron loading. CD163^+^ macrophages of non-survivors documented significant increase in the expression of ADAM17 and associated genes while expression of genes linked to ER-stress, autophagy and apoptosis were reduced. This observation indicates that macrophage death in iron loaded SAH is predominantly due to necrosis or lipid peroxidation (ferroapoptosis)^16,25^.

To ascertain whether iron alone or iron in presence of peroxidative stress (Fenton) is responsible for gene expression alteration observed in CD163^+^ macrophages, we subjected MDMs and THP-1 derived macrophages to iron/Fenton stress at increasing concentrations in the presence or absence of iron chelator, deferiprone. A temporal and dose-dependent increase in expression of genes related to oxidative-stress, iron-regulation, macrophage iron processing, and metalloproteases was seen in macrophages which were neutralized under deferiprone, suggesting a central role of iron in inflammation, oxidative-stress and metalloprotease activation^24^. Iron and Fenton both induced the protein expression of ADAM17, CD163 and ADAM10. Under iron chelation, the expression of ADAM17 decreased and the expression of CD163 increased. Blocking of ADAM 17 increased cellular expression of CD163, TNF-α and reduced the inflammation irrespective of iron/Fenton stress. These novel observations suggest that Iron/Fenton produces inflammation through ADAM17 and iron-chelation reduces ADAM17 induction and inflammation. This also signifies that ADAM17 and CD163 expression are under iron regulation^10^ and inversely correlate with each other and could modulate inflammation in SAH patients.

In summary, the results of this novel study clearly show that in patients with SAH, iron overload induces a Fenton reaction and a vicious cycle of oxidative stress^21,26^. Increased circulatory iron stimulates macrophage iron loading via activation of *TFRC, TFR2, DMT1* and induction of the CD163 signaling cascade. Intracellular labile iron pool generates peroxide stress and induces genes linked to iron homeostasis (*hepcidin, ferritin*), inflammation (*TNF-α* and *ADAM17*) and oxidative-stress. This results in increased circulatory sCD163 and TNF-α levels (Fig. 6F). Iron chelation on the other hand, reduces the expression of ADAM17 and TNF-α underlining the fact that iron plays a major role in inflammation and progression of disease to organ failure in SAH. It would be worthwhile to explore iron and ADAM17 as attractive therapeutic targets in these patients.

## Methods

### Patients

In this prospective study, 120 liver-biopsy proven SAH patients were enrolled between January 2013 to January 2015. Nineteen patients were excluded due to hepatocellular carcinoma (10), portal vein thrombosis (5) and previous history of plasmapheresis. SAH was diagnosed by histological criteria and Maddery’s discriminant function (DF) of >32^23^. Alcoholic Cirrhosis (AC) was diagnosed on history of chronic heavy alcohol intake (with >1-month alcohol abstinence) and a combination of clinical, biochemical, endoscopic and radiological criteria^27^. Healthy controls (HC) had no evidence of present/past liver disease (Supplementary Fig. 1). For comparison of iron-load (n = 5; Scheuer-grade ≥3+), thalassemic patients were included as positive control.

The patient groups received standard medical therapy based on their clinical status, including albumin, lactulose, bowel washes, antibiotics and pentoxifylline. At the time of patient enrolment, treatment of SAH with corticosteroid was not a part of standard of care at our center and hence, none of the enrolled patients received corticosteroids. Patients were followed-up for a period of three months or until death. Only baseline samples were analyzed and correlated with outcomes. The laboratory staff were unaware of the clinical details. The study was approved by the institutional ethical committee/ institutional review board Institute of liver and biliary science (ILBS) New Delhi, India and written informed consent was obtained in all cases. Further all the experiments were performed in accordance with relevant guidelines and regulations of the concerned ethical committee.

## Methods

### Discovery

RNA-Seq was performed in the initial 15 SAH patients, which were further grouped into SAH with iron load (SAH-IO; Scheuer-grade ≥1+, Group A: n = 5) and SAH with no-iron load (SAH-NIO; Group B: n = 10) based on histological evidences. One sample was excluded from the last group due to poor liver RNA quality (RIN < 7). Differentially expressed genes (DEG) identified in Gr.A versus B were cherry picked and validated in the validation cohort (Supplementary Fig. 1).

### Validation

Target genes and linked mechanisms were identified after univariate and multivariate projection analysis and were validated in the representative liver tissue from 5 SAH patients and 5 alcoholic cirrhosis using immuno-histochemistry. Circulatory protein levels were validated in 100 SAH patients and compared to 20 alcoholic cirrhosis and 20 controls (Supplementary Fig. 1). *In-vivo* and *in-vitro* experiments were performed to understand the mechanisms of iron-loading, inflammation, and stress in the macrophages of SAH patients.

### Transcriptomic analysis

RNA-Seq was performed in the liver and PBMC samples from both Group A and B patients. Heat map for the differentially expressed genes (DEG) was generated on ‘R’, and supervised clustering was performed only on significantly (p < 0.05) modulated genes. GO and Pathway analysis was performed using Enrichr^28^. Genes segregating Group A from B were validated for expression and mechanisms using immuno-histochemistry, ELISA or Western-blot (Supplementary methods).

### Immuno-histochemistry

Immunohistochemistry was performed on formalin-fixed paraffin embedded liver tissues (n = 5 each) of Group A, B, AC and thalassemia. Expression of CD163 (CAT-No: PA5-14215) and CD68 (CAT-No: PA5-32331) was estimated in the sinusoidal spaces. Expression of ADAM10 (CAT-No: PA5-28161), and ADAM17 (CAT-No: PA5-27395) was estimated in the membrane and cytoplasmic space of the positive stained cells. The positively stained cells were counted in 10 consecutive high power fields (40×) and relative quantization in terms of mean number of cells/10 high power field (40×) was calculated.

### Enzyme-linked immunosorbent assay

Serum iron was measured using Quanti-Chrom™ Iron-Assay [CAT-No: DIFE-250 (Sensitivity >27 μg/dL-1,000 μg/dL)], Ferritin [Cat-No: EF2003-1(Sensitivity >1.5 ng/mL)]. Soluble CD163 (sCD163) [CAT-No: DC1630 (Sensitivity: 0.613 ng/mL (1.56–100) ng/mL)], TNF-α levels [CAT-No: 88-7346-22 (Sensitivity >4 pg/mL)], Hemoglobin-Haptoglobin (He-Hp) complex [CAT-No: K7837D (Sensitivity >0.036 ug/ml)], TNF-R1 [CAT-No: SEB 499 hu (Sensitivity >6.5 pg/ml) and TNF-R2 [CAT-No: SEB 504 hu (Sensitivity >0.055 ng/ml) were also measured using manufacturer’s protocols.

### Flow cytometry analysis

PBMCs were isolated^13^ and incubated with Fc blocker (CD16/CD32) BD bioscience USA for a period of 15 minutes to avoid nonspecific binding this was followed by surface stained with a combination of FITC anti-CD14, phycoerythrin anti-CD45, and phycoerythrin-cy7 anti-CD11b, allophycocanin anti-CD163 antibodies for 30 minutes at 4 °C, washed with PBS and fixed in 0.5% paraformaldehyde. The cells were analyzed on CYAN flow-cytometer (DAKO, USA) using Summit-4.3 software.

### Peroxide detection in CD163^+^ cells using H2DCFA

A minimum of 10^6^ PBMCs from 1 month non-survivors (10) and survivors (10) of SAH were first stained with APC-CD163 antibody for 30 minutes followed by H2DCFA for a 10 minutes. The cells were washed thrice with PBS and analyzed on CYAN flow-cytometry (DAKO, USA) using Summit-4.3 software.

### Sorting of CD163^+ve^ and CD163^−ve^Cell population

PBMCs from randomly chosen 3 survivors and 3 non-survivors of SAH were stained with FITC-anti-CD163 antibodies for 30 minutes at 4°C and sorted on a BD FACS Aria II (BD Biosciences San-Jose, CA 95131-1807USA) for CD163^+^ and CD163^−^ cell populations. Purity of positive stained cells was found to be 98% Flowjo (v-10, USA). Total RNA was isolated from CD163^+^ and CD163^-^cells and RT-PCR analysis was performed for 68 genes linked to oxidative-stress, antioxidant response, iron-regulation, cytokines, metalloproteases, macrophages iron processing, apoptosis, ER-stress and autophagy.

### MDM or THP-1 stimulation assay

Monocyte derived Macrophages (MDMs) were obtained from PBMCs of HC and were maintained in culture system^13,29^. THP-1 monocytes were also differentiated into macrophages by 24 h incubation with 150 nM phorbol 12-myristate 13-acetate (PMA, Sigma) followed by 24 h incubation in RPMI medium. A total of 10^6^ cells of each type were treated in triplicate with iron (FeCl_2_) or Fenton reagent (Iron and H_2_0_2_) at a concentration of 50 uM/L, 100uM/L, 500uM/L in presence or absence of Deferiprone at a concentration of 150 uM/L, 300uM/L and 1500uM/L^22,30^. Deferiprone (Kelfer) is an iron chelator which binds iron at 3:1 ratio. Total RNA and protein was isolated from the treated and non-treated MDM and THP1 cells and subjected to RT-PCR analysis for a panel of 68 genes. The protein samples were used for validation of expression of CD163 (CAT-No:PA5-14215), ADAM10 (CAT-No:PA5–28161), and ADAM17 (CAT-No:PA5-27395) using western blot.

### ADAM17 blocking assay

THP1 macrophages were treated with Iron/Fenton reagent in presence or absence of TAPI-1 (Inhibitor of ADAM17; CAS 171235-71-5, Merck, USA)^31^ or left untreated. Frequency of CD163^+^ and CD163^+^TNF-α^+^ cells was analysed. sCD163 and TNF-α levels were analysed in the cell supernatant and total RNA was used for RT-PCR analysis (Supplementary methods).

### Statistical Analysis

Statistical analyses were performed using SPSS version 20. Unpaired two-tail Student’s t-test and one-way analysis of variance was used for normally distributed continuous variables. Comparison between three groups was performed using one way-ANOVA and Kruskal-Wallis test. Comparison between two groups was performed using Mann–Whitney-U test. Spearman’s correlation was drawn comparing TNF-α, sCD163 and He-Hp complex levels and severity scores. Receiver Operating Characteristic Curve (ROC) analysis was performed and Youden’s index was calculated for non-survivors; Cox regression and C-statistic logistic regression model were performed to determine the significance of TNF-α and sCD163 in predicting the outcome in SAH.

## Electronic supplementary material

Supplementary Information
