# Supplementary material for: Iron-Overload triggers ADAM-17 mediated inflammation in Severe Alcoholic Hepatitis
Source: Sci Rep. 2018 Jul 6;8:10264. doi: 10.1038/s41598-018-28483-x (PMC6035223; doi:10.1038/s41598-018-28483-x)
Supplement: Supplementary file 1 — Supplementary Information [file 41598_2018_28483_MOESM1_ESM.pdf]

Supplementary material for the manuscript

Iron-Overload triggers ADAM-17 mediated inflammation in Severe Alcoholic Hepatitis

Jaswinder Singh Maras[1], Sukanta Das[1], Sachin Sharma[1], Sukriti Sukriti[1], Jitendra kumar[1], Ashish Kumar Vyas[1], Dhananjay Kumar[1], Adil Bhat[1], Gaurav Yadav[1], Manish Chandra Chaudhary[1], Shvetank Sharma[1], Guresh kumar[1], Chhagan Bihari[2], Nirupma Trehanpati[1], Rakhi Maiwall[3], Shiv Kumar Sarin[1,3*]

Department of Molecular and Cellular Medicine[1], Department of Pathology[2], Department of Hepatology[3], Institute of Liver and Biliary Sciences, New Delhi 110070 India

This document contains

1) **Supplementary method**
2) **Supplementary figures and figure legends**
3) **Supplementary tables and table legends**

Supplementary methods

Transcriptomics analysis:

RNA Seq was performed on paired samples for liver biopsy and Peripheral Blood Mononuclear Cells (PBMC) from severe alcoholic hepatitis patients with hepatic iron load (Gr.A: SAHIO: n=5; scheuer grade 1+, Perl's Staining positive) as compared to no hepatic iron load (Gr.B: SAHNIO: n=10; scheuer grade < 1+, Perl's staining negative) form the study cohort. Total RNA was isolated (mirVana™ miRNA Isolation Kit Life technologies USA) from the liver biopsy and PBMC samples. cDNA library was prepared using mRNA-Seq-8 Sample Preparation Kit (Illumina, San Diego, CA). Briefly poly-A containing mRNA was isolated and purified from total RNA from the samples. The purified m-RNA was fragmented using divalent cations at 94°C for 5 minutes. cDNA was prepared using random hexamer primers and reverse transcriptase (Fermentas USA). cDNA was purified using QIAquick PCR Purification Kit (Qiagen). The purified cDNA fragments were end repaired to convert the 5′ and 3′ overhangs into blunt phosphorylated ends using T4 DNA polymerase and Klenow DNA polymerase before adding a single “A” base to the 3′ end of the blunt phosphorylated cDNA fragments using 3′-to-5′ exo-nuclease. Adaptors were ligated to the ends of the cDNA fragments. Size selection was done using 2% agarose gel. Approximately 200 bpcDNA was excised and gel purified then enriched by PCR amplification for 15 cycles. Each library was quantified using Agilent DNA 1000 kit (Agilent) on Agilent 2100 Bioanalyzer. These libraries were denaturated using NaOH and diluted to a final concentration of 6 pM. 100 µl of these diluted libraries were used on Cluster Station using Clustering Generation Kit v4 (Illumina). Sequencing was done HiSeq 2000 (Illumina). Image analysis and base calling was done using Genome Analyzer Pipeline software v1.5.0 (Illumina) to generate raw fastq files. The raw data was analysed and normalized on AVADIS (strand life sciences, USA) after normalization the data was compared for the expression of genes in Gr.A as compared to Gr.B. Genes which significantly segregated Gr.A from Gr.B were identified

and validated for mechanism and expression using immuno-histochemistry and ELISA analysis. Heat map for the differentially regulated gene was generated on R, and supervised clustering analysis was performed only on $p<0.05$ significant genes. Pathway, and GO analysis of the differentially regulated genes was performed using a web based server Enrichr [1]. All the Raw data has been submitted to NCBI SRA archive database under NCBI Bioproject – PRJNA415466.

ADAM17 blocking assay

THP1 Monocytes were cultures in RPMI medium. A total of 10^6 cells in triplicate were first treated with PMA (150nM) and then with Iron and Fenton at 50, 100 and 500uM/L in presence or absence of TAPI-1(50mM/L) [2] (Inhibitor of ADAM17; CAS 171235-71-5, Merk USA) or left untreated. Cells were used for frequency calculation of $CD163^+$ and $CD163^+TNF\alpha^+$ cells. The cell supernatant was subjected to the measurement of CD163 (sCD163) [CAT-No: DC1630 (Sensitivity: 0.613ng/mL (1.56-100) ng/mL)] and TNF-alpha levels [CAT-No:88-7346-22 (Sensitivity>4pg/mL)]. Total RNA was isolated form the cells and was subjected to cDNA and RT-PCR analysis for the panel of 68 genes used in the study.

Reference:

1. Kuleshov MV, Jones MR, Rouillard AD, et al. Enrichr: a comprehensive gene set enrichment analysis web server 2016 update. Nucleic acids research 2016;**44**(W1):W90-7 doi: 10.1093/nar/gkw377[published Online First: Epub Date]|.
2. Breshears LM, Schlievert PM, Peterson ML. A disintegrin and metalloproteinase 17 (ADAM17) and epidermal growth factor receptor (EGFR) signaling drive the epithelial response to Staphylococcus aureus toxic shock syndrome toxin-1 (TSST-1). The Journal of biological chemistry 2012;**287**(39):32578-87 doi: 10.1074/jbc.M112.352534[published Online First: Epub Date]|.

Supplementary Figures

Supplementary Figure-1: Flow chart documenting patients inclusion in the discovery and validation experiments of the study.

Supplementary Figure-2: %$CD11b^{+}CD163^{+}$ macrophages in PBMC of (n=56) non-survivors [2.0(0.5-10.2)] vs. (n=44) survivors[1.2(0.5-4.7)] of SAH (p=0.015).

Supplementary Figure-3: Heat map for the gene expression normalized against control for the MDMs and THP1 cells treated with varied concentration of Iron or Fenton in presence or absence of iron chelator (Defriprone). Red colour represent upregulated and green colour represents downregulated expression genes analysed in various modules.

Supplementary Figure-4: Percentage frequency of CD163+ and CD163+, TNF-α+ in THP1 cells stimulated by Iron/Fenton(100uM and 500uM) under ADAM17 inhibition.(*=p<0.05)

Supplementary Figure-5: Cell supernatant level of sCD163 and TNF-α+ in THP1 cells stimulated by Iron/Fenton(100uM and 500uM) under ADAM17 inhibition.(*=p<0.05)

Supplementary Figure-6: Expression of genes linked to inflamation, iron regulation and macrophage iron regulation in THP1 cells stimulated by Iron/Fenton(100uM and 500uM) under ADAM17 inhibition.

Supplementary Figure 7 : Raw data for figure 5C,D western blot analysis

Supplementary Figure 8: Percentage frequency of CD163-, TNF-α+ cells in THP1 cells stimulated by Iron/Fenton(50mM) under ADAM17 inhibition.(*=p<0.05)

Supplementary Table legends:

Supplementary Table-1: Genes Differentially expressed between Gr.A:SAH-IO and Gr.B:SAH-NIO, in the Liver biopsy transcriptome :gene expression values expressed in mean and SD, p<0.05 is significant

Supplementary Table-2: GO Classification and Pathway analysis for the Genes differentially expressed in the liver biopsy transcriptome of Gr.A:SAH-IO, p<0.05 is significant and combined score >1 (C-score>1) is significant.

Supplementary Table-3: Variable important in Projection Score (VIP) for the Genes Differentially expressed in liver biopsy of Gr.A:SAH-IO, VIP>1 is significant.

Supplementary Table-4: Expression changes in the genes associated to inflamation, oxidative stress and iron processing in liver biopsy and PBMC of Gr.A:SAH-IO vs. Gr.B:SAH-NIO. Values are expressed in Mean-SD and $p<0.05$ is significant.

Supplementary Table-5: Genes Differentially expressed between Gr.A:SAH-IO and Gr.B:SAH-NIO in PBMC transcriptome: gene expression values expressed in Mean and SD, $p<0.05$ is significant

Supplementary Table-6: GO Classification and Pathway analysis for the Genes differentially expressed in the PBMC transcriptome of Gr.A:SAH-IO, $p<0.05$ and c-score>1 is significant.

Supplementary Table-7: VIP of the Genes Differentially expressed in PBMC of Gr.A:SAH-IO as compared to Gr.B:SAH-NIO, VIP>1 is significant.

Supplementary Table-8: Univariate (Mann-witney) and Multivariate (OPLS-DA) analysis of the genes differentially expressed in Liver and PBMC of Gr.A:SAH-IO vs. Gr.B:SAH-NIO, Gene expression values expressed as Mean-SD, $p<0.05$ and VIP>1 is significant.

Supplementary Table-9: Spearman Correlation Analysis for sCD163 and TNF-α with mortality in SAH patients $p<0.05$ is significant

Supplementary Table-10: ROC curve comparative analysis of the most significantly altered and validated variables (Hanley & McNeil, 1983 method) $p<0.05$ is significant.

.

Supplementary Figure 1

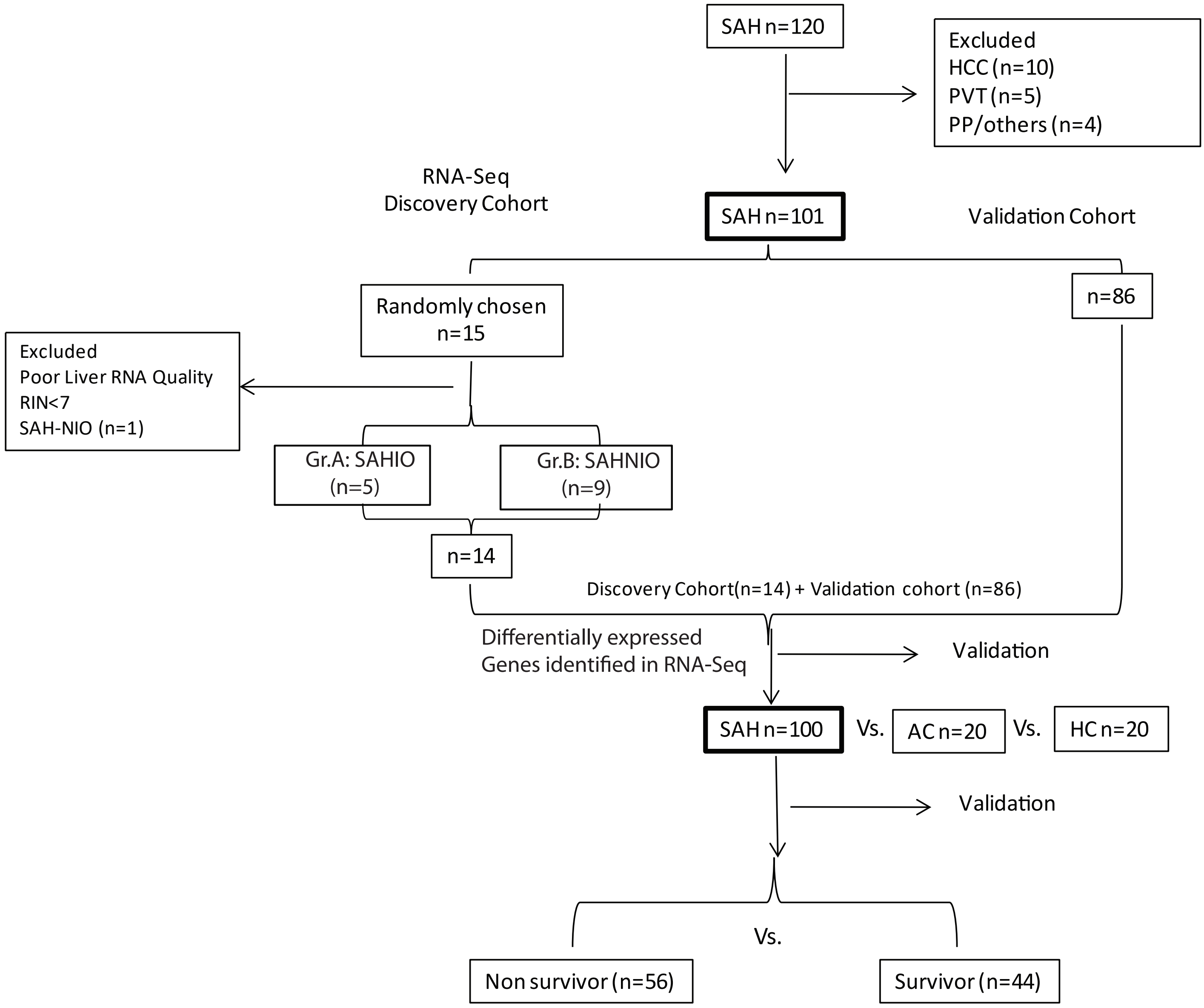

Supplementary Figure 2

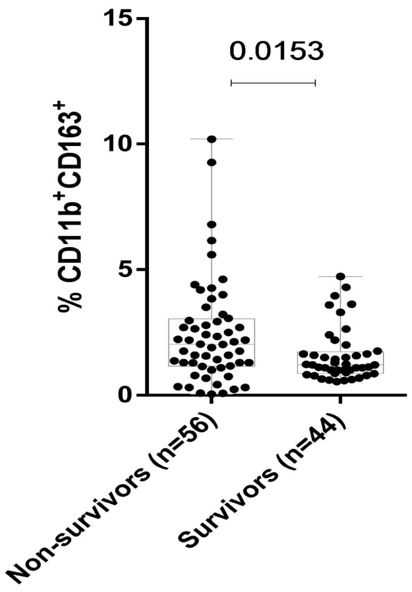

Supplementary Figure 3

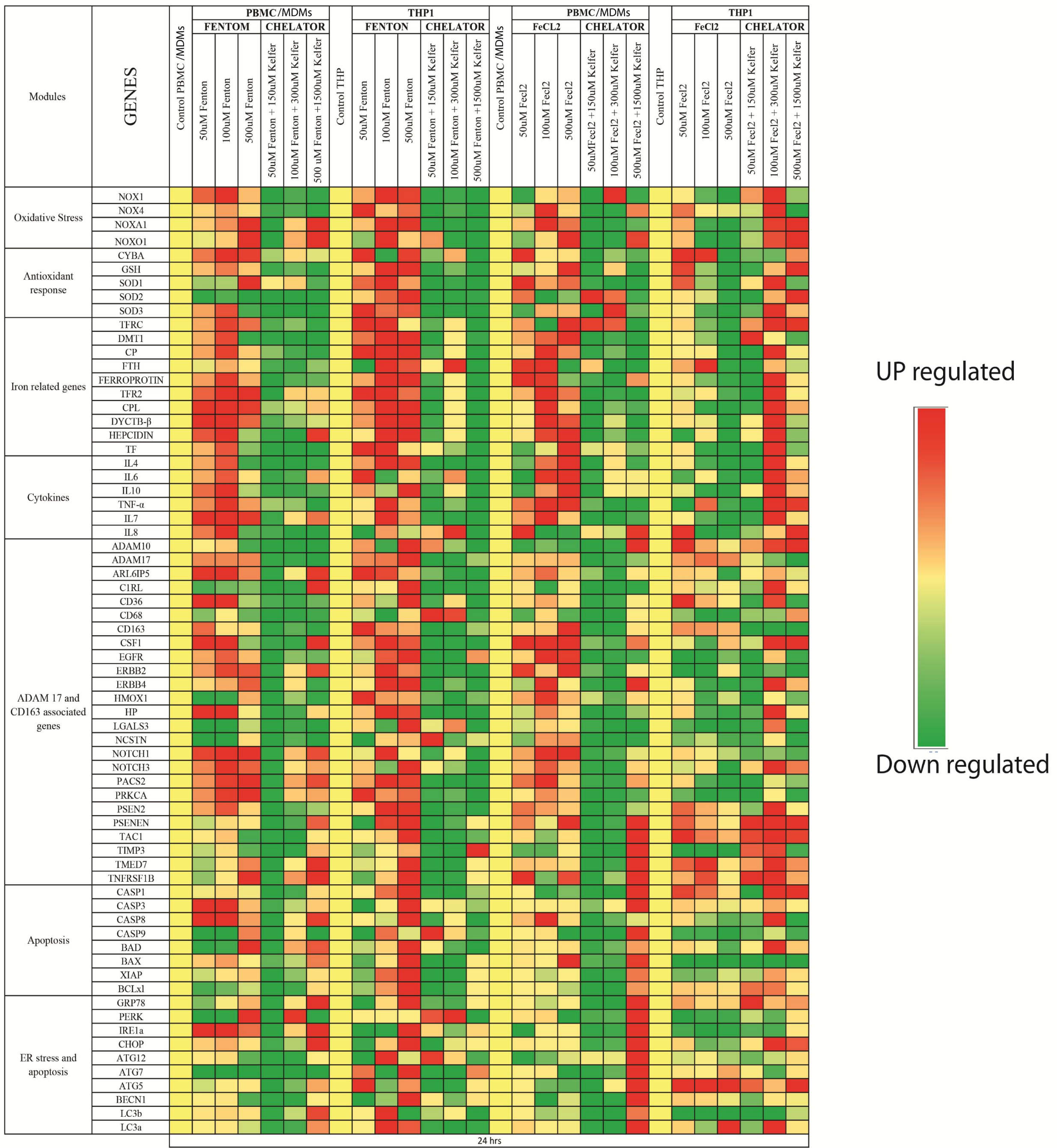

Figure 4

Supplementary Figure

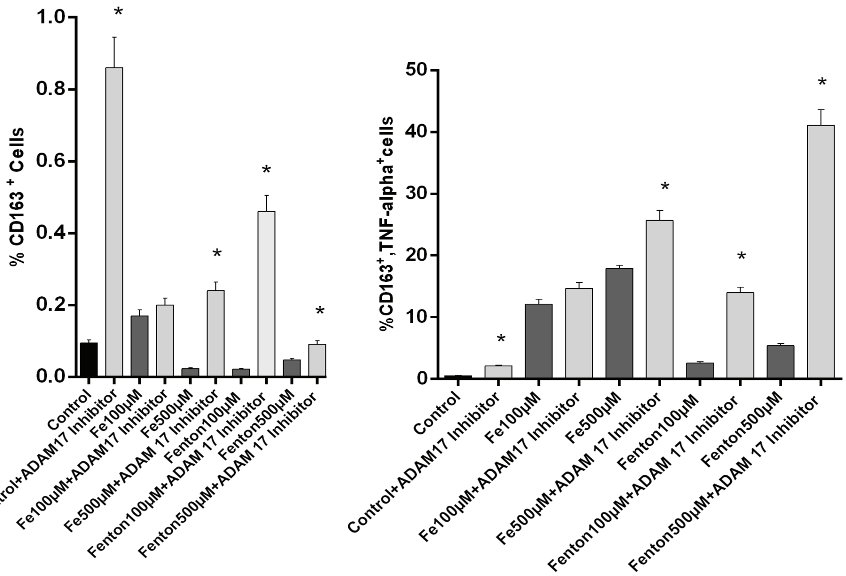

Figure 5

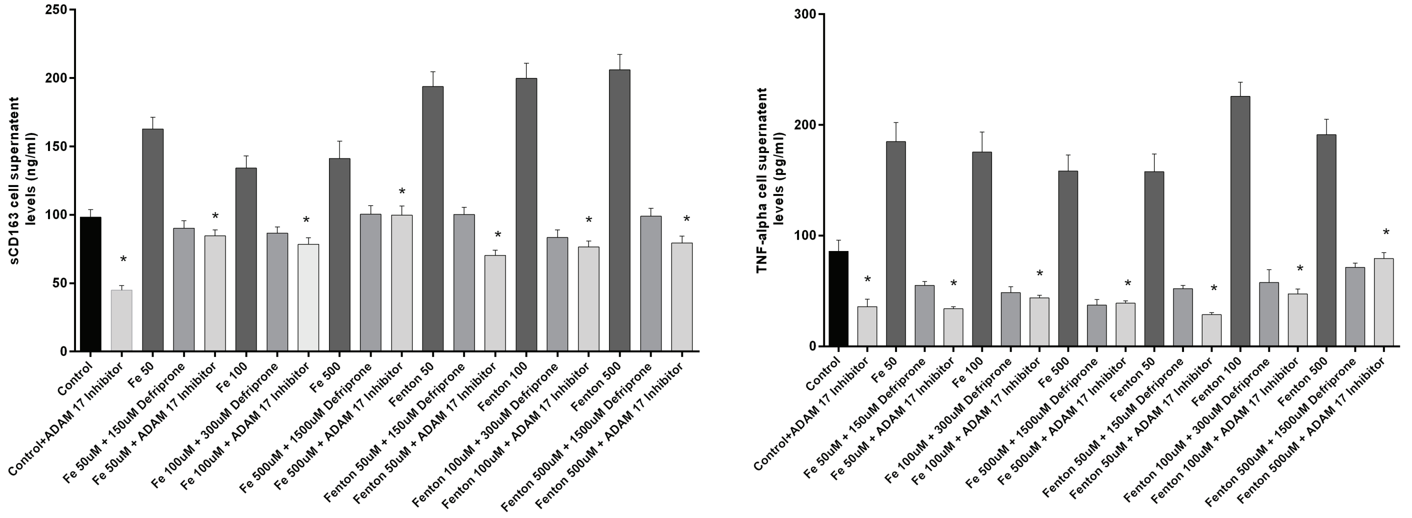

Figure 6

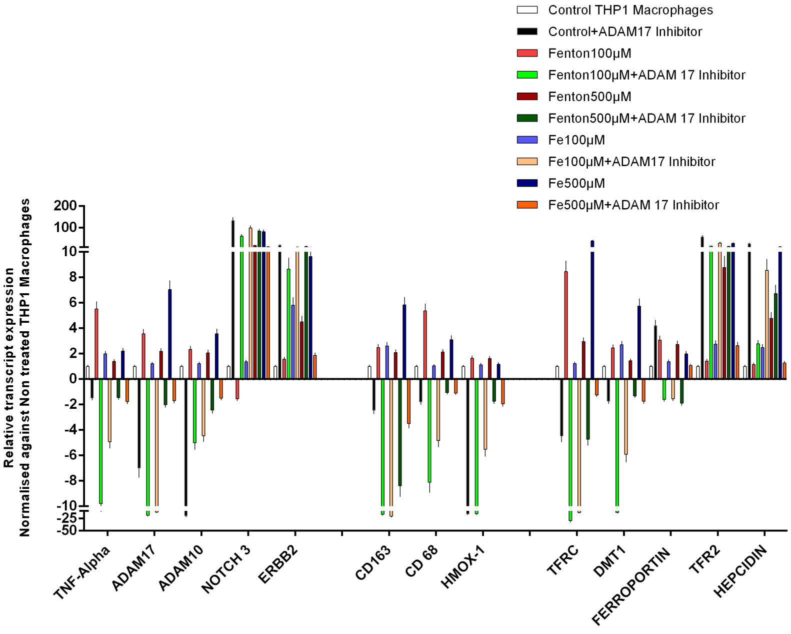

Figure 7

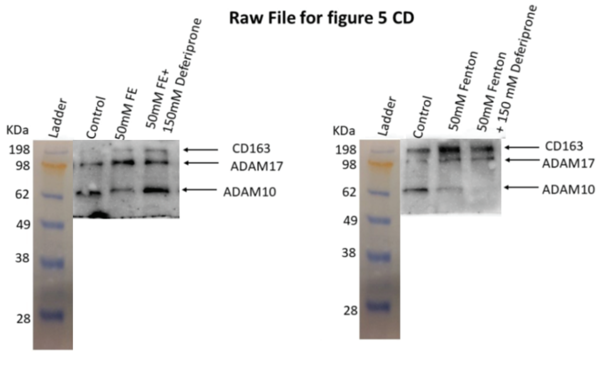

Figure 8

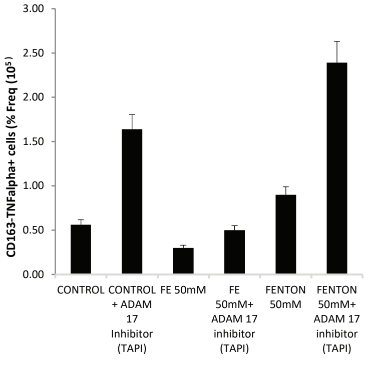

Supplementary Table-1 : Liver biopsy transcriptome : Genes Differentially regulated between GR.A:SAHIO and GR.B:SAHNIO

Univariate analysis Liver biopsy GR.A:SAHIO vs GR.B:SAHNIO						
SR.NO	GENE	Mean (SD) of GR.B:SAHNIO	Mean (SD) of GR.A:SAHIO	p.value	FC (log) GR.A:SAHIO/GR.B:SAHNIO	Status
1	APOE	-19.759 (2.153)	35.566 (39.034)	0.000999	55.325	UP
2	HP	-17.132 (44.209)	30.838 (36.572)	0.041958	47.97	UP
3	ALB	-10.261 (32.248)	18.469 (36.673)	0.018981	28.73	UP
4	HSPA1B	-3.416 (0.975)	6.149 (19.053)	0.041958	9.565	UP
5	HSPA1A	-3.379 (0.382)	6.083 (16.523)	0.001998	9.462	UP
6	HSPB1	-3.108 (2.345)	5.595 (7.179)	0.003996	8.703	UP
7	AGRN	-2.875 (1.362)	5.175 (9.628)	0.028971	8.05	UP
8	**HMOX1**	-0.278 (1.205)	1.501 (1.080)	0.02536	7.779	UP
9	CYB5A	-2.588 (1.895)	4.658 (8.886)	0.041958	7.246	UP
10	**ERBB2**	-2.523 (0.091)	4.541 (0.927)	0.000999	7.064	UP
11	HSPA1L	-2.442 (0.768)	4.396 (12.776)	0.028971	6.838	UP
12	MAGED1	-2.378 (0.668)	4.281 (9.081)	0.011209	6.659	UP
13	SERPINA5	-2.116 (4.246)	3.809 (10.693)	0.044553	5.925	UP
14	TAGLN	-2.096 (1.181)	3.772 (5.530)	0.028971	5.868	UP
15	CP	-2.033 (1.535)	3.660 (1.173)	0.000999	5.693	UP
16	**CD163**	-1.898 (0.174)	3.416 (1.567)	0.000999	5.314	UP
17	MAF	-1.750 (1.937)	3.150 (5.128)	0.018981	4.901	UP
18	NFKBIL1	-1.737 (3.962)	3.126 (8.922)	0.044553	4.863	UP
19	C21orf33	-1.712 (1.221)	3.081 (6.429)	0.023258	4.793	UP
20	ZBED1	-1.670 (1.261)	3.007 (6.627)	0.041958	4.677	UP
21	TPST2	-1.642 (1.094)	2.955 (5.241)	0.041958	4.597	UP
22	PRKCDBP	-1.627 (1.079)	2.929 (4.161)	0.032131	4.556	UP
23	TSKU	-1.587 (2.414)	2.857 (8.584)	0.023258	4.444	UP
24	NUDC	-1.552 (3.125)	2.794 (6.732)	0.018981	4.346	UP
25	CYGB	-1.540 (0.434)	2.771 (4.617)	0.001998	4.311	UP
26	IQCJ-SCHIP1	-1.515 (1.290)	2.728 (4.069)	0.041958	4.243	UP
27	PRDX4	-1.503 (0.216)	2.705 (7.031)	0.032131	4.208	UP
28	SLC25A1	-1.489 (1.343)	2.680 (4.249)	0.032705	4.169	UP
29	MAGEF1	-1.447 (1.202)	2.605 (2.844)	0.0049175	4.052	UP
30	ST13	-1.416 (1.596)	2.548 (3.622)	0.023258	3.964	UP
31	BLVRB	-1.410 (1.157)	2.539 (4.390)	0.022804	3.949	UP
32	EFHD1	-1.391 (0.589)	2.505 (5.490)	0.028971	3.896	UP
33	**PROS1**	-1.357 (0.525)	2.443 (5.520)	0.028971	3.802	UP

34	TRAPPC12	-1.353 (1.197)	2.436 (2.897)	0.045263	3.789	UP
35	PCBD1	-1.351 (0.381)	2.431 (4.140)	0.040809	3.782	UP
36	GPX1	-1.343 (0.343)	2.418 (2.413)	0.001998	3.761	UP
37	ZNF622	-1.333 (2.299)	2.400 (4.940)	0.045263	3.733	UP
38	MAZ	-1.310 (1.892)	2.358 (3.500)	0.041958	3.668	UP
39	EI24	-1.303 (0.413)	2.345 (4.286)	0.015925	3.648	UP
40	SLC27A4	-1.295 (0.306)	2.330 (4.207)	0.015925	3.625	UP
41	**MIF**	-1.272 (3.372)	2.289 (4.781)	0.01249	3.561	UP
42	CUX2	-1.245 (0.561)	2.241 (3.911)	0.045263	3.486	UP
43	CD68	-1.237 (1.240)	2.227 (2.020)	0.006993	3.464	UP
44	ZNF224	-1.230 (0.356)	2.215 (3.883)	0.007008	3.445	UP
45	HOPX	-1.230 (0.390)	2.214 (4.073)	0.044191	3.444	UP
46	SNRPC	-1.229 (0.872)	2.212 (4.817)	0.029122	3.441	UP
47	DHRSX	-1.219 (0.864)	2.194 (4.184)	0.018981	3.413	UP
48	RDH10	-1.191 (1.949)	2.145 (2.617)	0.022804	3.336	UP
49	ROMO1	-1.191 (3.178)	2.144 (3.996)	0.029122	3.335	UP
50	ABHD17C	-1.188 (0.552)	2.139 (2.509)	0.0063859	3.327	UP
51	GSPT2	-1.187 (1.442)	2.136 (4.030)	0.022804	3.323	UP
52	HIGD1A	-1.184 (0.354)	2.131 (2.208)	0.0032159	3.315	UP
53	KIAA1586	-1.171 (1.032)	2.107 (4.766)	0.015925	3.278	UP
54	TMEM56-RWDD3	-1.167 (0.876)	2.100 (4.345)	0.015232	3.267	UP
55	ESRP2	-1.166 (1.280)	2.098 (3.286)	0.041958	3.264	UP
56	RPS16	-1.163 (1.310)	2.094 (3.682)	0.043141	3.257	UP
57	MBL2	-1.163 (1.167)	2.093 (3.328)	0.041958	3.256	UP
58	AUP1	-1.150 (0.573)	2.071 (3.000)	0.032705	3.221	UP
59	OTC	-1.148 (0.693)	2.067 (3.160)	0.040809	3.215	UP
60	MFRP	-1.140 (0.545)	2.053 (2.190)	0.016277	3.193	UP
61	SNX22	-1.139 (1.249)	2.051 (2.929)	0.041958	3.19	UP
62	C1RL	-1.128 (0.216)	2.030 (3.098)	0.000999	3.158	UP
63	PCSK6	-1.123 (0.849)	2.022 (5.367)	0.028971	3.145	UP
64	DHFR	-1.117 (0.874)	2.010 (3.192)	0.016277	3.127	UP
65	PACS2	-1.115 (0.158)	2.006 (4.348)	0.000999	3.121	UP
66	SPRY2	-1.108 (0.826)	1.994 (5.020)	0.044553	3.102	UP
67	PKIG	-1.097 (2.820)	1.975 (3.153)	0.030992	3.072	UP
68	TXNRD2	-1.093 (0.839)	1.968 (3.846)	0.006993	3.061	UP
69	ZBED1a	-1.090 (2.125)	1.963 (3.155)	0.045263	3.053	UP
70	**TMED7**	-1.080 (0.319)	1.945 (4.951)	0.041958	3.025	UP
71	SRP9	-1.059 (0.941)	1.906 (3.697)	0.032131	2.965	UP
72	RENBP	-1.056 (1.119)	1.900 (2.988)	0.045263	2.956	UP
73	CPE	-1.053 (1.160)	1.895 (2.854)	0.022804	2.948	UP
74	GPT2	-1.048 (2.322)	1.886 (5.125)	0.028971	2.934	UP

75	PRKCH	-1.047 (0.278)	1.884 (3.998)	0.0032159	2.931	UP
76	DFFA	-1.045 (0.585)	1.880 (2.903)	0.015925	2.925	UP
77	ZNF766	-1.044 (0.519)	1.879 (2.414)	0.007008	2.923	UP
78	KIF1C	-1.040 (0.673)	1.872 (3.131)	0.028971	2.912	UP
79	TMEM261	-1.037 (0.780)	1.867 (2.741)	0.0063859	2.904	UP
80	LARP6	-1.035 (0.973)	1.863 (3.174)	0.021906	2.898	UP
81	XPC	-1.030 (0.636)	1.854 (3.940)	0.018981	2.884	UP
82	GSR	-1.029 (0.546)	1.852 (1.302)	0.001998	2.881	UP
83	VAMP3	-1.028 (0.374)	1.850 (3.286)	0.044553	2.878	UP
84	PNPLA2	-1.024 (1.877)	1.843 (3.346)	0.041958	2.867	UP
85	ADAM15	-1.023 (0.521)	1.842 (3.465)	0.011988	2.865	UP
86	PFN1	-1.022 (0.854)	1.839 (3.736)	0.041958	2.861	UP
87	FNIP2	-1.015 (0.307)	1.827 (3.726)	0.032705	2.842	UP
88	FAM46A	-1.002 (1.569)	1.804 (3.145)	0.022804	2.806	UP
89	SCD	-0.987 (1.290)	1.777 (4.512)	0.041958	2.764	UP
90	SMARCD2	-0.986 (0.872)	1.775 (4.516)	0.041958	2.761	UP
91	TIMP2	-0.985 (3.182)	1.773 (5.175)	0.041958	2.758	UP
92	TSG101	-0.982 (1.936)	1.767 (2.977)	0.015232	2.749	UP
93	HSD17B10	-0.976 (2.537)	1.758 (4.477)	0.040809	2.734	UP
94	FOXO4	-0.968 (0.872)	1.742 (3.403)	0.032131	2.71	UP
95	RNF103-CHMP3	-0.952 (1.509)	1.714 (2.486)	0.022804	2.666	UP
96	ARMC6	-0.947 (1.492)	1.704 (2.798)	0.022804	2.651	UP
97	TPD52L2	-0.946 (1.328)	1.703 (3.887)	0.045263	2.649	UP
98	TOMM40L	-0.939 (0.562)	1.691 (4.632)	0.030992	2.63	UP
99	**TUSC2**	-0.938 (0.986)	1.689 (2.358)	0.021906	2.627	UP
100	YARS	-0.930 (1.250)	1.674 (2.957)	0.041958	2.604	UP
101	TSPYL2	-0.927 (3.249)	1.668 (5.412)	0.041958	2.595	UP
102	TRPV1	-0.924 (0.586)	1.663 (2.548)	0.045263	2.587	UP
103	PCBP1	-0.923 (1.220)	1.662 (3.643)	0.041958	2.585	UP
104	CDR2	-0.919 (0.893)	1.655 (2.881)	0.015925	2.574	UP
105	MPLKIP	-0.915 (1.399)	1.648 (3.645)	0.044553	2.563	UP
106	JTB	-0.914 (0.538)	1.645 (2.657)	0.015232	2.559	UP
107	PLBD2	-0.912 (0.653)	1.642 (2.860)	0.032131	2.554	UP
108	Sep_2	-0.907 (0.546)	1.633 (2.491)	0.011988	2.54	UP
109	SMOC1	-0.907 (0.798)	1.632 (4.374)	0.022804	2.539	UP
110	H2AFV	-0.905 (0.296)	1.628 (4.307)	0.043141	2.533	UP
111	IQSEC1	-0.902 (0.584)	1.624 (2.763)	0.018981	2.526	UP
112	**KLHL11**	-0.898 (2.663)	1.617 (3.850)	0.044553	2.515	UP
113	ARMCX3	-0.891 (0.410)	1.604 (3.585)	0.032131	2.495	UP
114	**NOTCH3**	-0.891 (0.325)	1.603 (1.046)	0.000999	2.494	UP
115	CCL20	-0.75 (0.174)	1.78 (3.271)	0.003	2.493	UP

116	ABHD6	-0.890 (1.789)	1.602 (3.024)	0.032131	2.492	UP
117	IGSF8	-0.879 (0.740)	1.581 (2.194)	0.041958	2.46	UP
118	TK2	-0.867 (0.582)	1.561 (1.621)	0.0075941	2.428	UP
119	EGLN1	-0.866 (0.589)	1.559 (1.932)	0.041958	2.425	UP
120	PAICS	-0.861 (1.020)	1.551 (1.318)	0.007396	2.412	UP
121	PRNP	-0.861 (1.391)	1.550 (1.707)	0.028971	2.411	UP
122	SYCE1	-0.857 (1.855)	1.542 (4.412)	0.044553	2.399	UP
123	DHCR24	-0.856 (1.194)	1.541 (2.225)	0.018981	2.397	UP
124	HEXA	-0.856 (2.034)	1.541 (3.519)	0.041958	2.397	UP
125	APBB2	-0.850 (1.196)	1.530 (2.287)	0.041958	2.38	UP
126	KIAA1919	-0.850 (1.334)	1.530 (2.445)	0.045263	2.38	UP
127	FOXO3	-0.847 (1.341)	1.525 (2.102)	0.028971	2.372	UP
128	TRAM2	-0.838 (0.657)	1.508 (1.885)	0.007396	2.346	UP
129	FAM199X	-0.836 (0.435)	1.506 (3.703)	0.032705	2.342	UP
130	TSPYL4	-0.834 (0.513)	1.501 (2.716)	0.045263	2.335	UP
131	IGFBP1	-0.831 (3.155)	1.496 (3.513)	0.041958	2.327	UP
132	RFTN1	-0.824 (1.545)	1.483 (2.632)	0.032131	2.307	UP
133	TJP2	-0.823 (1.032)	1.482 (2.777)	0.041958	2.305	UP
134	CADPS2	-0.823 (0.729)	1.481 (2.627)	0.032131	2.304	UP
135	LRRC19	-0.819 (0.858)	1.475 (3.532)	0.040809	2.294	UP
136	KLHL42	-0.818 (1.470)	1.472 (1.882)	0.022804	2.29	UP
137	STIM1	-0.813 (0.908)	1.464 (2.292)	0.044553	2.277	UP
138	ZNF398	-0.808 (0.351)	1.454 (3.341)	0.032131	2.262	UP
139	**ADAM17**	-0.804 (0.226)	1.448 (0.794)	0.000999	2.252	UP
140	ZDHHC15	-0.803 (0.557)	1.446 (1.798)	0.010942	2.249	UP
141	PSMD7	-0.800 (1.386)	1.440 (2.605)	0.032705	2.24	UP
142	SHROOM3	-0.799 (1.144)	1.438 (1.557)	0.018981	2.237	UP
143	**HAMP**	-0.61 (0.215)	1.66 (1.239)	0.001	2.235	UP
144	NOA1	-0.795 (2.701)	1.432 (4.979)	0.015232	2.227	UP
145	GLB1	-0.793 (0.530)	1.427 (2.133)	0.044553	2.22	UP
146	SETDB2	-0.791 (0.580)	1.424 (1.285)	0.0033187	2.215	UP
147	DYNC1LI1	-0.783 (1.568)	1.409 (3.937)	0.044553	2.192	UP
148	TNFRSF1B	-0.780 (0.498)	1.404 (1.322)	0.000999	2.184	UP
149	CTPS2	-0.778 (0.377)	1.400 (3.493)	0.018981	2.178	UP
150	PIN1	-0.774 (0.671)	1.393 (1.945)	0.045263	2.167	UP
151	MB21D2	-0.773 (0.531)	1.392 (2.327)	0.007008	2.165	UP
152	ZNF846	-0.772 (0.725)	1.389 (1.472)	0.010418	2.161	UP
153	CMPK2	-0.769 (0.357)	1.385 (3.209)	0.045263	2.154	UP
154	DNAJA3	-0.765 (1.298)	1.377 (2.372)	0.028971	2.142	UP
155	GNL2	-0.762 (0.445)	1.372 (2.014)	0.0032159	2.134	UP
156	EFNB2	-0.757 (0.654)	1.362 (2.123)	0.044553	2.119	UP

157	NOTCH1	-0.562 (0.720)	1.563(1.057)	0.0009	2.085	UP
158	TATDN2	-0.739 (0.678)	1.331 (3.372)	0.045263	2.072	UP
159	**FAM186B**	-0.739 (0.640)	1.330 (2.193)	0.040809	2.069	UP
160	RNF220	-0.738 (0.670)	1.329 (2.111)	0.045263	2.067	UP
161	**SOD1**	-0.735 (0.964)	1.324 (1.603)	0.011988	2.059	UP
162	MBNL2	-0.730 (0.714)	1.314 (1.754)	0.041958	2.044	UP
163	RBMX2	-0.728 (0.827)	1.310 (2.138)	0.021906	2.038	UP
164	GSTZ1	-0.721 (0.639)	1.297 (1.515)	0.023258	2.018	UP
165	C1orf115	-0.721 (0.919)	1.297 (1.927)	0.028971	2.018	UP
166	COX4I2	-0.720 (0.589)	1.296 (2.141)	0.032131	2.016	UP
167	C4orf29	-0.716 (0.804)	1.289 (2.988)	0.023258	2.005	UP
168	SMAD1	-0.715 (1.503)	1.287 (2.061)	0.022804	2.002	UP
169	MTIF3	-0.715 (0.591)	1.287 (2.149)	0.045263	2.002	UP
170	PHF8	-0.712 (0.819)	1.282 (3.240)	0.041958	1.994	UP
171	NOXO1	-0.708 (0.538)	1.274 (2.050)	0.0075941	1.982	UP
172	KIAA2022	-0.706 (0.899)	1.270 (1.639)	0.021906	1.976	UP
173	PXDN	-0.702 (1.118)	1.263 (1.635)	0.041958	1.965	UP
174	RNF31	-0.701 (0.439)	1.261 (2.457)	0.018981	1.962	UP
175	PRICKLE2	-0.700 (0.638)	1.259 (1.959)	0.045263	1.959	UP
176	ERBB4	-0.50 (0.784)	1.46 (0.394)	0.0003	1.932	UP
177	TIMMDC1	-0.686 (1.773)	1.235 (2.550)	0.044553	1.921	UP
178	STARD13	-0.685 (1.094)	1.234 (0.958)	0.015232	1.919	UP
179	**SAR1B**	-0.675 (0.711)	1.215 (3.223)	0.032705	1.893	UP
180	FADD	-0.674 (0.884)	1.212 (1.578)	0.023258	1.886	UP
181	ITGA2	-0.672 (0.602)	1.209 (1.306)	0.011209	1.881	UP
182	SRXN1	-0.671 (0.470)	1.208 (1.499)	0.011988	1.879	UP
183	ZNF275	-0.669 (1.404)	1.205 (1.944)	0.032705	1.874	UP
184	APLN	-0.667 (0.976)	1.201 (1.365)	0.030992	1.868	UP
185	FNDC3A	-0.643 (0.771)	1.157 (1.556)	0.045263	1.852	UP
186	TNFRSF11B	-0.643 (0.911)	1.157 (1.910)	0.022804	1.823	UP
187	CCDC50	-0.649 (1.037)	1.169 (2.007)	0.018981	1.818	UP
188	MZT2B	-0.645 (1.872)	1.161 (1.883)	0.032705	1.806	UP
189	ANKRD13C	-0.640 (0.802)	1.151 (1.653)	0.044553	1.791	UP
190	CXCL1	-0.637 (0.444)	1.147 (2.712)	0.018981	1.784	UP
191	RHOBTB3	-0.634 (1.089)	1.142 (1.259)	0.032705	1.776	UP
192	ZC3H10	-0.625 (2.422)	1.125 (3.954)	0.044553	1.751	UP
193	DDIT3	-0.622 (1.318)	1.120 (3.336)	0.045263	1.742	UP
194	SEC24A	-0.621 (0.800)	1.118 (1.715)	0.044553	1.739	UP
195	TTC5	-0.614 (0.697)	1.105 (1.538)	0.032705	1.719	UP
196	CDC14B	-0.597 (0.810)	1.075 (1.076)	0.010418	1.672	UP
197	RPRD1A	-0.590 (0.502)	1.061 (1.688)	0.011988	1.651	UP

198	UHRF2	-0.589 (0.672)	1.060 (0.730)	0.0050617	1.649	UP
199	ADAM10	-0.40(1.257)	1.28(1.146)	0.0327	1.644	UP
200	PPP1R3E	-0.582 (2.346)	1.047 (3.064)	0.015925	1.629	UP
201	SSRP1	-0.581 (0.950)	1.047 (1.769)	0.041958	1.628	UP
202	NIPSNAP3B	-0.580 (0.697)	1.044 (1.303)	0.043141	1.624	UP
203	CCDC7	-0.578 (0.848)	1.041 (0.943)	0.020439	1.619	UP
204	SERBP1	-0.577 (1.427)	1.039 (1.971)	0.028971	1.616	UP
205	TEAD1	-0.575 (0.842)	1.036 (0.705)	0.006993	1.611	UP
206	SCAP	-0.572 (1.309)	1.030 (1.564)	0.041958	1.602	UP
207	GATA1	-0.571 (0.549)	1.028 (2.092)	0.044553	1.599	UP
208	PDE8B	-0.571 (0.955)	1.027 (0.911)	0.015232	1.598	UP
209	G2E3	-0.571 (1.042)	1.027 (1.936)	0.032131	1.598	UP
210	ZSCAN25	-0.570 (0.937)	1.026 (1.583)	0.015925	1.596	UP
211	MTA2	-0.568 (1.189)	1.023 (1.448)	0.041958	1.591	UP
212	ZBTB18	-0.567 (0.576)	1.021 (1.704)	0.032131	1.588	UP
213	FLVCR2	-0.564 (1.190)	1.015 (2.181)	0.044553	1.579	UP
214	ZNF879	-0.560 (1.103)	1.008 (1.173)	0.022804	1.568	UP
215	**C2CD2L**	-0.556 (0.627)	1.001 (1.183)	0.018981	1.557	UP
216	LARP4	-0.554 (1.092)	0.997 (1.152)	0.032705	1.551	UP
217	IKBKB	-0.550 (0.544)	0.990 (1.689)	0.028971	1.54	UP
218	CEP112	-0.549 (1.566)	0.988 (1.835)	0.045263	1.537	UP
219	TCEB3	-0.543 (0.504)	0.978 (1.826)	0.041958	1.521	UP
220	KCNJ3	-0.541 (1.523)	0.974 (1.978)	0.030992	1.515	UP
221	PGBD1	-0.539 (0.869)	0.970 (1.359)	0.044553	1.509	UP
222	PSEN2	-0.532 (0.332)	0.958 (1.714)	0.0075941	1.49	UP
223	TACC2	-0.532 (0.751)	0.957 (1.631)	0.041958	1.489	UP
224	COL4A5	-0.528 (1.055)	0.951 (1.199)	0.029122	1.479	UP
225	WBP4	-0.517 (0.858)	0.931 (0.958)	0.045263	1.448	UP
226	ZNF655	-0.515 (0.569)	0.927 (0.946)	0.016277	1.442	UP
227	LNX2	-0.514 (0.809)	0.925 (1.279)	0.021906	1.439	UP
228	PARPBP	-0.506 (0.772)	0.911 (1.216)	0.043141	1.417	UP
229	BAG2	-0.505 (2.342)	0.909 (1.189)	0.018981	1.414	UP
230	CCNG2	-0.505 (0.879)	0.908 (1.673)	0.041958	1.413	UP
231	RIOK1	-0.503 (1.550)	0.905 (2.161)	0.032131	1.408	UP
232	KSR1	-0.499 (0.757)	0.899 (1.313)	0.041958	1.398	UP
233	PSMF1	-0.496 (1.019)	0.893 (1.012)	0.032705	1.389	UP
234	BRE	-0.496 (1.094)	0.893 (1.079)	0.043141	1.389	UP
235	CEP152	-0.495 (0.798)	0.890 (1.168)	0.043141	1.385	UP
236	TLK1	-0.493 (0.764)	0.887 (1.071)	0.018981	1.38	UP
237	C15orf38	-0.493 (2.877)	0.887 (1.999)	0.043141	1.38	UP
238	TMEM57	-0.491 (0.554)	0.883 (1.527)	0.041958	1.374	UP

239	TOM1L1	-0.489 (0.772)	0.881 (1.029)	0.032705	1.371	UP
240	CASP9	-0.481 (1.649)	0.865 (1.384)	0.032705	1.346	UP
241	CAT	-0.480 (1.254)	0.864 (1.748)	0.041958	1.344	UP
242	AKR1C4	-0.478 (1.065)	0.860 (1.148)	0.032131	1.338	UP
243	SUMO2	-0.476 (0.731)	0.856 (1.086)	0.041958	1.332	UP
244	SAV1	-0.464 (1.931)	0.835 (1.095)	0.045263	1.299	UP
245	RC3H2	-0.450 (0.825)	0.810 (1.299)	0.045263	1.26	UP
246	LTBP1	-0.445 (1.051)	0.802 (1.820)	0.023258	1.247	UP
247	CAPN5	-0.435 (0.642)	0.783 (1.528)	0.041958	1.218	UP
248	C1orf112	-0.433 (0.932)	0.779 (1.155)	0.045263	1.212	UP
249	TRPA1	-0.427 (1.106)	0.768 (0.694)	0.045263	1.195	UP
250	PELO	-0.415 (2.580)	0.746 (0.679)	0.032131	1.161	UP
251	FBXL17	-0.407 (2.016)	0.732 (2.267)	0.022804	1.139	UP
252	CAST	-0.404 (0.917)	0.728 (0.685)	0.028971	1.132	UP
253	BTBD7	-0.395 (0.933)	0.712 (0.728)	0.022804	1.107	UP
254	NEXN	-0.390 (1.804)	0.701 (1.038)	0.041958	1.091	UP
255	HERPUD2	-0.384 (0.946)	0.691 (1.013)	0.041958	1.075	UP
256	NYNRIN	-0.376 (0.635)	0.677 (0.840)	0.041958	1.053	UP
257	KIAA0232	-0.344 (0.529)	0.618 (0.688)	0.028971	0.962	UP
258	MANBA	-0.328 (1.098)	0.591 (1.142)	0.045263	0.919	UP
259	APMAP	-0.317 (0.661)	0.570 (0.602)	0.028971	0.887	UP
260	ATXN2	-0.304 (0.573)	0.548 (0.514)	0.018981	0.852	UP
261	**HKR1**	-0.222 (1.525)	0.400 (0.601)	0.044553	0.622	UP
262	PLCG2	0.227 (0.646)	-0.408 (0.365)	0.028971	-0.635	Down
263	CLNS1A	0.236 (0.614)	-0.425 (0.392)	0.028971	-0.661	Down
264	FAF2	0.237 (0.804)	-0.427 (0.388)	0.041958	-0.664	Down
265	IL1B	0.450 (1.238)	-0.222(0.257)	0.0312	-0.714	Down
266	XRCC1	0.287 (0.580)	-0.517 (0.472)	0.028971	-0.804	Down
267	ZSCAN2	0.317 (0.840)	-0.571 (0.705)	0.038552	-0.888	Down
268	IL13	0.51 (2.135)	-0.336(0.132)	0.0225	-0.896	Down
269	DIP2C	0.320 (0.990)	-0.576 (0.485)	0.041958	-0.896	Down
270	GTF2I	0.52 (0.696)	-0.39 (0.183)	0.007	-0.957	Down
271	NME5	0.530 (1.396)	-0.403(0.946)	0.029	-0.975	Down
272	NCSTN	0.533 (0.738)	-0.407(0.659)	0.0294	-0.982	Down
273	FCER2	0.359 (0.839)	-0.646 (0.492)	0.028971	-1.005	Down
274	MED1	0.362 (0.576)	-0.652 (0.406)	0.011988	-1.014	Down
275	PATL1	0.374 (1.001)	-0.673 (0.486)	0.028971	-1.047	Down
276	MSTO1	0.379 (0.991)	-0.683 (0.412)	0.011988	-1.062	Down
277	GPX6	0.382 (0.401)	-0.687 (0.790)	0.032705	-1.069	Down
278	TXNDC2	0.571 (0.955)	-0.476(0.713)	0.0471	-1.089	Down
279	PSENEN	0.583 (0.595)	-0.497(0.266)	0.001	-1.122	Down

280	MUC20	0.402 (1.153)	-0.724 (0.453)	0.028971	-1.126	Down
281	NUDT2	0.427 (1.685)	-0.768 (0.446)	0.028971	-1.195	Down
282	CHMP5	0.429 (1.576)	-0.772 (0.592)	0.028971	-1.201	Down
283	DIS3L	0.429 (1.010)	-0.773 (0.598)	0.011988	-1.202	Down
284	EIF3K	0.431 (1.239)	-0.777 (0.556)	0.011988	-1.208	Down
285	ATP5F1	0.444 (0.778)	-0.800 (0.516)	0.003996	-1.244	Down
286	ZNF473	0.447 (1.005)	-0.804 (0.649)	0.038552	-1.251	Down
287	PLEKHH1	0.450 (1.304)	-0.811 (0.475)	0.038552	-1.261	Down
288	MAP7D3	0.452 (1.634)	-0.813 (0.289)	0.041958	-1.265	Down
289	NCF2	0.651 (1.200)	-0.621(0.886)	0.0444	-1.314	Down
290	SIGLEC9	0.476 (1.238)	-0.857 (0.417)	0.038552	-1.333	Down
291	PDE6D	0.480 (0.714)	-0.864 (0.519)	0.003996	-1.344	Down
292	LOC100133301	0.514 (1.127)	-0.925 (0.557)	0.018981	-1.439	Down
293	SFTA2	0.519 (0.956)	-0.934 (0.316)	0.019496	-1.453	Down
294	AKR1C2	0.70 (1.110)	-0.71 (0.861)	0.027	-1.453	Down
295	CAMLG	0.519 (1.214)	-0.935 (0.785)	0.011988	-1.454	Down
296	LMLN	0.533 (1.351)	-0.959 (0.657)	0.018981	-1.492	Down
297	SFTPD	0.748 (0.763)	-0.795(0.918)	0.0046	-1.585	Down
298	C10orf76	0.569 (1.797)	-1.025 (0.387)	0.028971	-1.594	Down
299	HSCB	0.597 (1.786)	-1.075 (0.445)	0.011988	-1.672	Down
300	SULF1	0.611 (1.648)	-1.100 (0.618)	0.028971	-1.711	Down
301	CSNK2A1	0.614 (2.309)	-1.106 (0.383)	0.038552	-1.72	Down
302	GLA	0.82 (1.593)	-0.92 (0.263)	0.0096	-1.792	Down
303	PRG3	0.695 (1.436)	-1.251 (0.292)	0.000999	-1.946	Down
304	BRD8	0.722 (2.349)	-1.299 (0.226)	0.018981	-2.021	Down
305	GORAB	0.761 (2.634)	-1.370 (0.175)	0.038552	-2.131	Down
306	TTC16	0.773 (2.362)	-1.392 (0.870)	0.028971	-2.165	Down
307	FAM86B2	0.850 (5.563)	-1.530 (1.156)	0.030992	-2.38	Down
308	SLC40A1	0.884 (1.630)	-1.592 (0.123)	0.000999	-2.476	Down
309	PLA2G2A	0.907 (3.648)	-1.633 (0.786)	0.041958	-2.54	Down
310	GTF2A1L	0.945 (4.962)	-1.700 (0.103)	0.040809	-2.645	Down

Supplementary Table 2 : GO Classification and Pathway analysis for the Genes differentially regulated in GR.A:SAHIO as compared GR.B:SAHNIO

Upregulated genes in liver biopsy of GR.A:SAHIO vs. GR.B:SAHNIO				
**GO Biological process**	**P-value**	**Z-score**	**Combined Score**	**Genes involved**
response to oxidative stress (GO:0006979)	0.00	-2.39	19.68	EGLN1;PRNP;GPX1;SRXN1;TRPA1;TXNRD2;HP;DHCR24;FOXO3;CYGB;SOD1;PXDN;CAT;HMOX1;ROMO1;APOE;ZNF622;HSPA1A;MBL2
response to reactive oxygen species (GO:0000302)	0.00	-2.30	9.63	EGLN1;GPX1;TRPA1;TXNRD2;PXDN;CAT;HP;HMOX1;ROMO1;APOE;SOD1
Notch receptor processing (GO:0007220)	0.00	-2.74	9.61	NOTCH3;ADAM17;NOTCH1;PSEN2;ADAM10
negative regulation of steroid biosynthetic process (GO:0010894)	0.00	-2.81	6.37	SCAP;APOE;PDE8B;SOD1
negative regulation of steroid metabolic process (GO:0045939)	0.00	-2.74	6.22	SCAP;APOE;PDE8B;SOD1
positive regulation of lymphocyte migration (GO:2000403)	0.00	-2.59	5.87	ADAM17;CCL20;ADAM10;FADD
positive regulation of T cell migration (GO:2000406)	0.00	-2.53	5.75	ADAM17;CCL20;ADAM10;FADD
disruption of cells of other organism (GO:0044364)	0.00	-2.51	5.70	TUSC2;ALB;HAMP;MBL2
killing of cells of other organism (GO:0031640)	0.00	-2.51	5.69	TUSC2;ALB;HAMP;MBL2
regulation of T cell migration (GO:2000404)	0.00	-2.48	5.63	ADAM17;CCL20;ADAM10;FADD
response to inorganic substance (GO:0010035)	0.00	-2.48	5.62	EGLN1;PRNP;GPX1;TRPA1;TXNRD2;HP;TNFRSF11B;SOD1;CASP9;STIM1;CAT;ALB;PXDN;HMOX1;OTC
response to nutrient levels (GO:0031667)	0.00	-2.44	5.54	CADPS2;GPX1;ITGA2;TNFRSF11B;HIGD1A;SOD1;DDIT3;ALB;CAT;HMOX1;APOE;SLC27A4;OTC
tissue regeneration (GO:0042246)	0.00	-2.49	5.34	IGFBP1;GPX1;ADAM15;ERBB4
aging (GO:0007568)	0.00	-2.34	5.32	CASP9;GPX1;DNAJA3;CAT;TIMP2;SCAP;ROMO1;MIF;APOE;TNFRSF1B;SOD1
regulation of cholesterol biosynthetic process (GO:0045540)	0.00	-2.48	5.24	SCAP;APOE;SOD1
response to extracellular stimulus (GO:0009991)	0.00	-2.41	5.21	CADPS2;GPX1;ITGA2;TNFRSF11B;HIGD1A;SOD1;DDIT3;ALB;CAT;HMOX1;APOE;SLC27A4;OTC
cellular response to oxidative stress (GO:0034599)	0.00	-2.28	5.19	GPX1;PXDN;CAT;HMOX1;ROMO1;ZNF622;FOXO3;SOD1;HSPA1A
homeostasis of number of cells (GO:0048872)	0.00	-2.25	5.10	ZBTB18;IKBKB;ATXN2;HMOX1;FADD;RC3H2;SOD1
response to hydrogen peroxide (GO:0042542)	0.00	-2.21	5.02	GPX1;TRPA1;PXDN;CAT;HP;HMOX1;SOD1
extracellular matrix organization (GO:0030198)	0.00	-2.33	5.01	ITGA2;ADAM10;NOXO1;TNFRSF11B;LTBP1;ADAM17;PRDX4;ADAM15;SMOC1;PXDN;TIMP2;COL4A5;APBB2;AGRN
extracellular structure organization (GO:0043062)	0.00	-2.33	5.01	ITGA2;NOXO1;ADAM10;TNFRSF11B;LTBP1;ADAM17;PRDX4;ADAM15;SMOC1;PXDN;TIMP2;COL4A5;APBB2;AGRN
disruption of cells of other organism involved in symbiotic interaction (GO:0051818)	0.00	-2.18	4.94	TUSC2;ALB;MBL2
killing of cells in other organism involved in symbiotic interaction (GO:0051883)	0.00	-2.15	4.87	TUSC2;ALB;MBL2
apoptotic signaling pathway (GO:0097190)	0.00	-2.32	4.76	GPX1;MAGED1;EI24;PSEN2;TNFRSF1B;FOXO3;CASP9;ADAM17;DDIT3;HMOX1;FADD;ZNF622;FNIP2
regulation of lymphocyte migration (GO:2000401)	0.00	-2.45	4.70	ADAM17;CCL20;ADAM10;FADD
response to unfolded protein (GO:0006986)	0.00	-2.18	4.68	TSPYL2;IGFBP1;HERPUD2;TATDN2;HSPA1L;DDIT3;HSPB1;HSPA1A

hydrogen peroxide metabolic process (GO:0042743)	0.00	-2.36	4.47	GPX1;CAT;PXDN;SOD1
negative regulation of alcohol biosynthetic process (GO:1902931)	0.00	-2.37	4.43	SCAP;APOE;SOD1
negative regulation of apoptotic signaling pathway (GO:2001234)	0.00	-2.32	4.32	GPX1;G2E3;PSEN2;HSPB1;HMOX1;MIF;HIGD1A;GATA1;HSPA1A
neuromuscular junction development (GO:0007528)	0.00	-2.28	4.26	DNAJA3;ERBB2;COL4A5;AGRN
response to topologically incorrect protein (GO:0035966)	0.00	-2.19	4.22	TSPYL2;IGFBP1;HERPUD2;TATDN2;HSPA1L;DDIT3;HSPB1;HSPA1A
regulation of apoptotic signaling pathway (GO:2001233)	0.00	-2.45	4.16	GPX1;MAGED1;EI24;PSEN2;HSPB1;MIF;HIGD1A;GATA1;SOD1;G2E3;HMOX1;FADD;HSPA1A
positive regulation of neuron death (GO:1901216)	0.00	-2.15	4.11	CASP9;DDIT3;APOE;FOXO3;AGRN
positive regulation of growth (GO:0045927)	0.00	-2.25	4.11	IGFBP1;ADAM17;NOTCH1;ERBB4;ERBB2;ADAM10;APOE;AGRN;HOPX
reactive oxygen species metabolic process (GO:0072593)	0.00	-2.17	4.10	PRDX4;GPX1;PXDN;CAT;NOXO1;SOD1
peptide metabolic process (GO:0006518)	0.00	-2.12	4.06	GSTZ1;GPX1;GSR;PSEN2;CPE;PCSK6;SOD1
negative regulation of lipid biosynthetic process (GO:0051055)	0.00	-2.35	3.82	SCAP;APOE;PDE8B;SOD1
regulation of cholesterol metabolic process (GO:0090181)	0.00	-2.50	3.76	SCAP;APOE;SOD1
protein refolding (GO:0042026)	0.00	-2.41	3.69	HSPA1L;ST13;HSPA1A
ventricular septum morphogenesis (GO:0060412)	0.00	-2.41	3.63	EGLN1;NOTCH1;SAV1
developmental growth (GO:0048589)	0.00	-2.21	3.56	IGFBP1;GPX1;NOTCH1;ADAM15;ERBB4;RDH10;SPRY2;FOXO3
positive regulation of erythrocyte differentiation (GO:0045648)	0.00	-2.42	3.55	FOXO3;GATA1;HSPA1A
regulation of receptor biosynthetic process (GO:0010869)	0.00	-2.35	3.54	ANKRD13C;SEC24A;SCAP
response to nutrient (GO:0007584)	0.00	-2.19	3.52	GPX1;ITGA2;ALB;CAT;HMOX1;TNFRSF11B;SLC27A4;OTC
negative regulation of sequence-specific DNA binding transcription factor activity (GO:0043433)	0.00	-2.13	3.47	EGLN1;PRNP;DDIT3;DNAJA3;NFKBIL1;CAT;HMOX1
hydrogen peroxide catabolic process (GO:0042744)	0.00	-2.31	3.47	GPX1;CAT;PXDN
negative regulation of cell cycle (GO:0045786)	0.00	-2.35	3.38	TSPYL2;PRNP;TSG101;XPC;DHCR24;MIF;FOXO4;TOM1L1;PSMD7;DDIT3;TIMP2;APBB2;ZNF655;PSMF1
cell redox homeostasis (GO:0045454)	0.00	-2.02	3.30	GPX1;PRDX4;TXNRD2;DDIT3;GSR
intrinsic apoptotic signaling pathway (GO:0097193)	0.00	-2.19	3.30	CASP9;GPX1;DDIT3;EI24;HMOX1;ZNF622;TNFRSF1B;FNIP2
membrane protein ectodomain proteolysis (GO:0006509)	0.00	-2.23	3.29	ADAM17;PSEN2;ADAM10
regulation of cellular response to oxidative stress (GO:1900407)	0.01	-2.22	3.18	GPX1;HP;HSPB1;SOD1
regulation of extrinsic apoptotic signaling pathway via death domain receptors (GO:1902041)	0.01	-2.20	3.15	GPX1;PSEN2;HMOX1;FADD
regulation of neuron death (GO:1901214)	0.01	-2.32	3.11	CASP9;GPX1;DDIT3;HMOX1;DHCR24;APOE;AGRN;FOXO3;SOD1
cellular amide metabolic process (GO:0043603)	0.01	-2.15	3.07	GSTZ1;GPX1;GSR;PSEN2;CPE;PCSK6;SOD1;OTC
cell killing (GO:0001906)	0.00	-2.07	3.04	TUSC2;ALB;HAMP;MBL2
negative regulation of extrinsic apoptotic signaling pathway via death domain receptors (GO:1902042)	0.01	-2.28	3.00	GPX1;PSEN2;HMOX1
cellular component disassembly (GO:0022411)	0.01	-2.21	3.00	DFFA;SMARCD2;ADAM17;RPS16;ADAM15;TIMP2;COL4A5;NOXO1;ADAM10;MTIF3;GSPT2;TJP2

regulation of neuron apoptotic process (GO:0043523)	0.01	-2.26	2.98	CASP9;GPX1;DDIT3;HMOX1;APOE;FOXO3;AGRN;SOD1
response to hypoxia (GO:0001666)	0.01	-2.26	2.98	EGLN1;ADAM17;NOTCH1;ITGA2;CAT;PSEN2;SCAP;HMOX1;HIGD1A
hippo signaling (GO:0035329)	0.01	-2.25	2.97	TEAD1;SAV1;TJP2
neurotrophin TRK receptor signaling pathway (GO:0048011)	0.01	-2.25	2.97	IKBKB;CASP9;ADAM17;MAGED1;ERBB4;ERBB2;PSEN2;FOXO4;PCSK6;FOXO3
negative regulation of endopeptidase activity (GO:0010951)	0.01	-2.19	2.97	CAST;GPX1;DNAJA3;PROS1;TIMP2;DHCR24;PSMF1;RENBP;SERPINA5
regulation of oxidative stress-induced intrinsic apoptotic signaling pathway (GO:1902175)	0.01	-2.25	2.96	GPX1;HSPB1;SOD1
response to decreased oxygen levels (GO:0036293)	0.01	-2.23	2.94	EGLN1;ADAM17;NOTCH1;ITGA2;CAT;PSEN2;SCAP;HMOX1;HIGD1A
neurotrophin signaling pathway (GO:0038179)	0.01	-2.23	2.94	IKBKB;CASP9;ADAM17;ERBB4;MAGED1;ERBB2;PSEN2;FOXO4;PCSK6;FOXO3
protein maturation (GO:0051604)	0.01	-2.17	2.93	CASP9;TSG101;PRDX4;DNAJA3;PSEN2;CPE;DHCR24;FADD;PCSK6
positive regulation of neuron apoptotic process (GO:0043525)	0.01	-2.17	2.93	CASP9;DDIT3;AGRN;FOXO3
regulation of DNA-templated transcription in response to stress (GO:0043620)	0.01	-2.12	2.93	EGLN1;NOTCH1;DDIT3;HMOX1
positive regulation of kinase activity (GO:0033674)	0.01	-2.21	2.91	TSPYL2;IGFBP1;MAGED1;MIF;SOD1;TOM1L1;ADAM17;TATDN2;ERBB4;DDIT3;ERBB2;JTB;SPRY2;ZNF622
positive regulation of developmental growth (GO:0048639)	0.00	-1.97	2.90	NOTCH1;ERBB4;APOE;AGRN;HOPX
regulation of leukocyte migration (GO:0002685)	0.01	-2.16	2.89	ADAM17;CCL20;ITGA2;HMOX1;ADAM10;FADD
negative regulation of peptidase activity (GO:0010466)	0.01	-2.18	2.87	CAST;GPX1;DNAJA3;PROS1;TIMP2;DHCR24;PSMF1;RENBP;SERPINA5
cellular amino acid metabolic process (GO:0006520)	0.01	-2.17	2.86	EGLN1;YARS;GPX1;GPT2;GSR;CTPS2;HSD17B10;SOD1;GSTZ1;DHFR;PRDX4;PCBD1;OTC
epidermal growth factor receptor signaling pathway (GO:0007173)	0.01	-2.17	2.86	CASP9;ADAM17;ERBB4;ERBB2;SPRY2;ADAM10;FOXO4;FOXO3
negative regulation of response to oxidative stress (GO:1902883)	0.01	-2.16	2.85	GPX1;HP;HSPB1
ERBB signaling pathway (GO:0038127)	0.01	-2.16	2.85	CASP9;ADAM17;ERBB4;ERBB2;SPRY2;ADAM10;FOXO4;FOXO3
inflammatory response (GO:0006954)	0.01	-2.16	2.84	IKBKB;SMAD1;CD163;NOTCH1;CCL20;TUSC2;CEP152;HP;CXCL1;MIF;TNFRSF1B;MBL2
regulation of cell growth (GO:0001558)	0.01	-2.15	2.84	TSPYL2;IGFBP1;ADAM17;TSG101;ADAM15;EI24;ERBB2;ADAM10;APBB2;APOE;HSPA1A
regulation of response to oxidative stress (GO:1902882)	0.01	-2.12	2.84	GPX1;HP;HSPB1;SOD1
negative regulation of cellular response to oxidative stress (GO:1900408)	0.01	-2.15	2.83	GPX1;HP;HSPB1
positive regulation of leukocyte migration (GO:0002687)	0.01	-2.09	2.79	ADAM17;CCL20;ITGA2;ADAM10;FADD
regulation of chemotaxis (GO:0050920)	0.01	-2.11	2.79	EFNB2;ADAM17;NOTCH1;ITGA2;HSPB1;ADAM10
regulation of cardiac muscle tissue development (GO:0055024)	0.01	-2.07	2.77	EFNB2;NOTCH1;ERBB4;SAV1
regulation of behavior (GO:0050795)	0.01	-2.10	2.76	EFNB2;ADAM17;NOTCH1;ITGA2;ALB;ADAM10;HSPB1
posttranscriptional regulation of gene expression (GO:0010608)	0.01	-2.17	2.73	ITGA2;HSPB1;MTIF3;TNFRSF1B;FOXO3;LARP6;RC3H2;SRP9;ATXN2;DNAJA3;SERBP1;ERBB2;HSPA1A
regulation of intrinsic apoptotic signaling pathway (GO:2001242)	0.01	-2.07	2.72	GPX1;EI24;G2E3;HSPB1;MIF;SOD1
response to oxygen levels (GO:0070482)	0.02	-2.17	2.72	EGLN1;ADAM17;NOTCH1;ITGA2;CAT;PSEN2;SCAP;HMOX1;HIGD1A

positive regulation of cardiac muscle tissue development (GO:0055025)	0.01	-2.05	2.71	EFNB2;NOTCH1;ERBB4
regulation of steroid biosynthetic process (GO:0050810)	0.01	-2.05	2.71	SCAP;APOE;PDE8B;SOD1
cellular modified amino acid metabolic process (GO:0006575)	0.01	-2.05	2.70	GSTZ1;DHFR;EGLN1;GPX1;PRDX4;GSR;SOD1;OTC
glutathione metabolic process (GO:0006749)	0.01	-2.01	2.70	GSTZ1;GPX1;GSR;SOD1
intrinsic apoptotic signaling pathway in response to DNA damage (GO:0008630)	0.01	-2.03	2.69	CASP9;EI24;HMOX1;TNFRSF1B;FNIP2
negative regulation of intracellular signal transduction (GO:1902532)	0.01	-2.14	2.69	PRNP;GPX1;DNAJA3;PRKCDBP;G2E3;TIMP2;SPRY2;PIN1;HSPB1;APOE;MIF
protein processing (GO:0016485)	0.01	-2.02	2.66	CASP9;TSG101;DNAJA3;PSEN2;CPE;DHCR24;FADD;PCSK6
regulation of cardiac muscle cell proliferation (GO:0060043)	0.01	-2.00	2.64	NOTCH1;ERBB4;SAV1
regulation of reactive oxygen species metabolic process (GO:2000377)	0.01	-2.00	2.64	TUSC2;HP;NOXO1;ROMO1;SOD1
extracellular matrix disassembly (GO:0022617)	0.01	-1.99	2.63	ADAM17;ADAM15;TIMP2;COL4A5;NOXO1;ADAM10
regulation of endopeptidase activity (GO:0052548)	0.02	-2.09	2.62	CAST;CASP9;GPX1;PROS1;DNAJA3;TIMP2;DHCR24;FADD;PSMF1;RENBP;SERPINA5
collagen metabolic process (GO:0032963)	0.01	-1.95	2.57	ADAM17;ADAM15;ADAM10;COL4A5;TRAM2
negative regulation of hydrolase activity (GO:0051346)	0.02	-2.06	2.57	EGLN1;CAST;GPX1;PROS1;DNAJA3;TIMP2;SPRY2;DHCR24;PSMF1;RENBP;SERPINA5
cellular transition metal ion homeostasis (GO:0046916)	0.01	-1.95	2.57	PRNP;HMOX1;HAMP;CP;SOD1
membrane protein proteolysis (GO:0033619)	0.01	-1.95	2.56	ADAM17;PSEN2;ADAM10
cellular response to reactive oxygen species (GO:0034614)	0.01	-1.94	2.56	GPX1;PXDN;CAT;ROMO1;SOD1
triglyceride metabolic process (GO:0006641)	0.01	-1.93	2.55	SLC25A1;GPX1;CAT;APOE;PNPLA2
multicellular organismal macromolecule metabolic process (GO:0044259)	0.01	-1.91	2.52	ADAM17;ADAM15;COL4A5;ADAM10;TRAM2
alpha-amino acid metabolic process (GO:1901605)	0.01	-1.98	2.47	GSTZ1;DHFR;EGLN1;PRDX4;GPT2;CTPS2;PCBD1;OTC
negative regulation of extrinsic apoptotic signaling pathway (GO:2001237)	0.02	-1.96	2.45	GPX1;PSEN2;HMOX1;GATA1;HSPA1A
interaction with symbiont (GO:0051702)	0.01	-1.96	2.45	GPX1;TUSC2;MBL2
hematopoietic or lymphoid organ development (GO:0048534)	0.02	-1.93	2.41	ADAM17;TXNRD2;PSEN2;FADD;GATA1;RC3H2;SOD1
spleen development (GO:0048536)	0.01	-1.92	2.40	ADAM17;FADD;RC3H2
regulation of developmental growth (GO:0048638)	0.02	-1.89	2.37	NOTCH1;ERBB4;APOE;AGRN;HOPX;SAV1
regeneration (GO:0031099)	0.02	-1.89	2.36	IGFBP1;GPX1;ADAM15;ERBB4;APOE
cell fate commitment (GO:0045165)	0.02	-1.88	2.35	NOTCH3;SMAD1;NOTCH1;ERBB4;SPRY2;GATA1
ER-nucleus signaling pathway (GO:0006984)	0.01	-1.87	2.35	TSPYL2;IGFBP1;TATDN2;DDIT3;SCAP
negative regulation of growth (GO:0045926)	0.02	-1.94	2.34	TSPYL2;ATXN2;ADAM15;EI24;APBB2;SAV1;HSPA1A;MBL2
acylglycerol metabolic process (GO:0006639)	0.01	-1.86	2.34	SLC25A1;GPX1;CAT;APOE;PNPLA2
neutral lipid metabolic process (GO:0006638)	0.01	-1.86	2.33	SLC25A1;GPX1;CAT;APOE;PNPLA2
T cell differentiation in thymus	0.02	-1.86	2.32	ADAM17;DNAJA3;FADD

(GO:0033077)				
multicellular organismal metabolic process (GO:0044236)	0.01	-1.83	2.30	ADAM17;ADAM15;COL4A5;ADAM10;TRAM2
cell aging (GO:0007569)	0.01	-1.83	2.28	DNAJA3;ROMO1;MIF;SOD1
regulation of peptidase activity (GO:0052547)	0.02	-2.00	2.28	CAST;CASP9;GPX1;PROS1;DNAJA3;TIMP2;DHCR24;FADD;PSMF1;RENBP;SERPINA5
cell cycle arrest (GO:0007050)	0.02	-1.89	2.27	PRNP;TSG101;DDIT3;APBB2;DHCR24;FOXO4
activation of signaling protein activity involved in unfolded protein response (GO:0006987)	0.02	-1.77	2.21	TSPYL2;IGFBP1;TATDN2;DDIT3
growth (GO:0040007)	0.02	-1.94	2.21	IGFBP1;GPX1;NOTCH1;ADAM15;ERBB4;RDH10;RFTN1;SPRY2;FOXO3;RC3H2
receptor clustering (GO:0043113)	0.02	-1.83	2.20	DNAJA3;APOE;AGRN
G2 DNA damage checkpoint (GO:0031572)	0.02	-1.73	2.17	BRE;FOXO4;CDC14B
regulation of erythrocyte differentiation (GO:0045646)	0.02	-1.79	2.15	FOXO3;GATA1;HSPA1A
regulation of cardiac muscle tissue growth (GO:0055021)	0.02	-1.74	2.15	NOTCH1;ERBB4;SAV1
cellular iron ion homeostasis (GO:0006879)	0.02	-1.78	2.14	HMOX1;HAMP;CP;SOD1
protein homooligomerization (GO:0051260)	0.02	-1.88	2.14	PRNP;ST13;CAT;HMOX1;PCBD1;MIF;LNX2;OTC
positive regulation of behavior (GO:0048520)	0.02	-1.88	2.14	ADAM17;ITGA2;ALB;ADAM10;HSPB1
protein localization to membrane (GO:0072657)	0.02	-1.88	2.14	RPS16;PACS2;DNAJA3;RFTN1;CPE;APOE;AGRN;SRP9;VAMP3
negative regulation of lipid metabolic process (GO:0045833)	0.02	-1.73	2.12	SCAP;APOE;PDE8B;SOD1
response to transition metal nanoparticle (GO:1990267)	0.02	-1.85	2.11	PRNP;ALB;HMOX1;SOD1;OTC
positive regulation of nuclease activity (GO:0032075)	0.02	-1.74	2.09	TSPYL2;IGFBP1;TATDN2;DDIT3
signal transduction in response to DNA damage (GO:0042770)	0.02	-1.82	2.07	CASP9;PSMD7;PSMF1;MIF;FOXO3
nucleobase metabolic process (GO:0009112)	0.02	-1.72	2.07	GPX1;CAT;TK2;PAICS
regulation of oxidative stress-induced cell death (GO:1903201)	0.02	-1.78	2.02	GPX1;HSPB1;SOD1
regulation of striated muscle tissue development (GO:0016202)	0.03	-1.78	2.02	EFNB2;NOTCH1;ERBB4;DDIT3;SAV1
positive regulation of cell growth (GO:0030307)	0.02	-1.77	2.02	IGFBP1;ADAM17;ERBB2;ADAM10;APOE
regulation of sequence-specific DNA binding transcription factor activity (GO:0051090)	0.03	-1.84	2.01	IKBKB;EGLN1;RNF31;PRNP;PRKCH;DNAJA3;DDIT3;NFKBIL1;CAT;HMOX1
maintenance of location (GO:0051235)	0.03	-1.74	1.98	ANKRD13C;NFKBIL1;ALB;APOE;LTBP1;PNPLA2
lung alveolus development (GO:0048286)	0.02	-1.73	1.97	PSEN2;RC3H2;HOPX
regulation of muscle tissue development (GO:1901861)	0.03	-1.76	1.96	EFNB2;NOTCH1;ERBB4;DDIT3;SAV1
cytokine production (GO:0001816)	0.02	-1.68	1.96	MAF;TUSC2;CEP152;RFTN1
retina homeostasis (GO:0001895)	0.02	-1.70	1.93	ALB;HSPB1;SOD1
regulation of nuclease activity (GO:0032069)	0.02	-1.69	1.92	TSPYL2;IGFBP1;TATDN2;DDIT3
regulation of muscle organ development (GO:0048634)	0.03	-1.75	1.92	EFNB2;NOTCH1;ERBB4;DDIT3;SAV1
negative regulation of intrinsic apoptotic signaling pathway (GO:2001243)	0.03	-1.67	1.90	GPX1;G2E3;HSPB1;MIF

purine nucleobase metabolic process (GO:0006144)	0.02	-1.66	1.88	GPX1;CAT;PAICS
organic acid catabolic process (GO:0016054)	0.03	-1.72	1.88	GSTZ1;GPT2;PCBD1;HSD17B10;RENBP;SLC27A4;OTC
carboxylic acid catabolic process (GO:0046395)	0.03	-1.72	1.88	GSTZ1;GPT2;PCBD1;HSD17B10;RENBP;SLC27A4;OTC
negative regulation of proteolysis (GO:0045861)	0.04	-1.82	1.88	CAST;GPX1;DNAJA3;PROS1;TIMP2;DHCR24;PSMF1;RENBP;SERPINA5
positive regulation of dendrite development (GO:1900006)	0.02	-1.64	1.87	SMAD1;CUX2;APOE
wound healing (GO:0042060)	0.03	-1.64	1.87	SMAD1;ITGA2;ERBB2;HMOX1
regulation of ERK1 and ERK2 cascade (GO:0070372)	0.03	-1.79	1.87	ERBB4;ERBB2;PRKCDBP;SPRY2;PIN1;MIF
acute-phase response (GO:0006953)	0.02	-1.63	1.86	CD163;HP;MBL2
positive regulation of myeloid cell differentiation (GO:0045639)	0.03	-1.63	1.86	FADD;FOXO3;GATA1;HSPA1A
regulation of extrinsic apoptotic signaling pathway (GO:2001236)	0.03	-1.77	1.85	GPX1;PSEN2;HMOX1;FADD;GATA1;HSPA1A
response to metal ion (GO:0010038)	0.04	-1.80	1.85	CASP9;PRNP;STIM1;ALB;HMOX1;TNFRSF11B;SOD1;OTC
negative regulation of muscle cell differentiation (GO:0051148)	0.03	-1.62	1.85	NOTCH1;DDIT3;FOXO4
regulation of transcription from RNA polymerase II promoter in response to stress (GO:0043618)	0.03	-1.62	1.84	EGLN1;NOTCH1;HMOX1
response to vitamin (GO:0033273)	0.03	-1.61	1.83	GPX1;ITGA2;CAT;OTC
positive regulation of protein kinase activity (GO:0045860)	0.04	-1.81	1.82	TSPYL2;IGFBP1;TOM1L1;ADAM17;TATDN2;MAGED1;DDIT3;ERBB2;JTB;SPRY2;MIF;SOD1
response to drug (GO:0042493)	0.04	-1.80	1.81	SMAD1;ADAM17;TRPA1;ITGA2;EI24;TIMP2;XPC;TNFRSF11B;SOD1;OTC
pyrimidine-containing compound biosynthetic process (GO:0072528)	0.03	-1.58	1.79	CTPS2;CMPK2;TK2
fibroblast growth factor receptor signaling pathway (GO:0008543)	0.04	-1.72	1.77	CASP9;ERBB4;ERBB2;SPRY2;FOXO4;FOXO3
collagen catabolic process (GO:0030574)	0.03	-1.56	1.77	ADAM17;ADAM15;ADAM10;COL4A5
regulation of steroid metabolic process (GO:0019218)	0.03	-1.62	1.77	SCAP;APOE;PDE8B;SOD1
regulation of organ growth (GO:0046620)	0.03	-1.56	1.77	NOTCH1;ERBB4;SAV1;SOD1
regulation of alcohol biosynthetic process (GO:1902930)	0.03	-1.55	1.76	SCAP;APOE;SOD1
regulation of heart growth (GO:0060420)	0.03	-1.55	1.76	NOTCH1;ERBB4;SAV1
modification of morphology or physiology of other organism (GO:0035821)	0.03	-1.58	1.74	TUSC2;ALB;HAMP;MBL2
cardiac septum morphogenesis (GO:0060411)	0.03	-1.57	1.73	EGLN1;NOTCH1;SAV1
thymus development (GO:0048538)	0.03	-1.51	1.72	PSEN2;FADD;SOD1
response to wounding (GO:0009611)	0.04	-1.71	1.72	SMAD1;ITGA2;ERBB2;HMOX1;APOE;SOD1
regulation of translation (GO:0006417)	0.04	-1.76	1.71	ATXN2;ITGA2;ERBB2;HSPB1;MTIF3;FOXO3;LARP6;SRP9
negative regulation of immune system process (GO:0002683)	0.04	-1.75	1.71	PRNP;GPX1;NOTCH1;DNAJA3;NFKBIL1;ERBB2;HMOX1;MIF;FADD
cellular amino acid catabolic process (GO:0009063)	0.03	-1.61	1.70	GSTZ1;GPT2;PCBD1;HSD17B10;OTC
positive regulation of chemokine production (GO:0032722)	0.03	-1.50	1.70	ADAM17;HMOX1;MIF
response to other organism (GO:0051707)	0.04	-1.73	1.69	IKBKB;GPX1;CCL20;ITGA2;TUSC2;HP;HSPB1;ROMO1;FADD;HAMP;HSPA1A;MBL2

transition metal ion homeostasis (GO:0055076)	0.03	-1.62	1.68	PRNP;HMOX1;HAMP;CP;SOD1
regulation of muscle cell differentiation (GO:0051147)	0.04	-1.62	1.67	EFNB2;NOTCH1;DDIT3;FOXO4;HOPX
regulation of homeostatic process (GO:0032844)	0.04	-1.73	1.66	ADAM17;SEC24A;DDIT3;TNFRSF11B;MIF;FADD;FOXO3;GATA1;HSPA1A
small molecule catabolic process (GO:0044282)	0.04	-1.68	1.66	GSTZ1;GPT2;PCBD1;APOE;HSD17B10;RENBP;SLC27A4;OTC
DNA damage checkpoint (GO:0000077)	0.03	-1.51	1.65	BRE;XPC;FOXO4;CDC14B
negative regulation of protein processing (GO:0010955)	0.05	-1.70	1.61	CAST;GPX1;DNAJA3;PROS1;TIMP2;DHCR24;PSMF1;RENBP;SERPINA5
negative regulation of protein maturation (GO:1903318)	0.05	-1.69	1.61	CAST;GPX1;DNAJA3;PROS1;TIMP2;DHCR24;PSMF1;RENBP;SERPINA5
lymphocyte homeostasis (GO:0002260)	0.03	-1.44	1.58	IKBKB;FADD;RC3H2
sulfur compound metabolic process (GO:0006790)	0.04	-1.64	1.57	SLC25A1;GSTZ1;TPST2;GPX1;GLB1;HEXA;GSR;AGRN;SOD1
negative regulation of cell motility (GO:2000146)	0.04	-1.61	1.57	NOTCH1;ADAM15;HMOX1;PIN1;ABHD6;APOE
multicellular organismal catabolic process (GO:0044243)	0.03	-1.50	1.57	ADAM17;ADAM15;ADAM10;COL4A5
retinoid metabolic process (GO:0001523)	0.03	-1.50	1.56	RDH10;AKR1C4;APOE;AGRN
regulation of striated muscle cell differentiation (GO:0051153)	0.04	-1.55	1.56	EFNB2;NOTCH1;DDIT3;HOPX
regulation of binding (GO:0051098)	0.04	-1.61	1.54	ADAM15;ITGA2;PSEN2;HMOX1;PCBD1;GATA1;HOPX
negative regulation of mitotic cell cycle (GO:0045930)	0.04	-1.62	1.54	TOM1L1;PSMD7;TIMP2;XPC;FOXO4;PSMF1;ZNF655
platelet degranulation (GO:0002576)	0.03	-1.47	1.52	PROS1;ALB;PFN1;SOD1
regulation of blood vessel endothelial cell migration (GO:0043535)	0.03	-1.43	1.51	NOTCH1;HSPB1;APOE
endoplasmic reticulum unfolded protein response (GO:0030968)	0.04	-1.45	1.49	TSPYL2;IGFBP1;TATDN2;DDIT3
protein folding (GO:0006457)	0.05	-1.56	1.48	PRDX4;BAG2;HSPA1L;ST13;DNAJA3;PIN1;HSPA1A
regulation of coagulation (GO:0050818)	0.04	-1.46	1.47	PROS1;FAM46A;PSEN2;APOE
regulation of epithelial cell migration (GO:0010632)	0.04	-1.53	1.47	NOTCH1;ITGA2;HSPB1;APOE;PFN1
positive regulation of muscle organ development (GO:0048636)	0.03	-1.40	1.47	EFNB2;NOTCH1;ERBB4
positive regulation of striated muscle tissue development (GO:0045844)	0.03	-1.39	1.46	EFNB2;NOTCH1;ERBB4
positive regulation of smooth muscle cell proliferation (GO:0048661)	0.03	-1.39	1.45	NOTCH3;ITGA2;HMOX1
regulation of protein binding (GO:0043393)	0.04	-1.49	1.45	ADAM15;ITGA2;PSEN2;PCBD1;HOPX
detection of external stimulus (GO:0009581)	0.05	-1.51	1.43	TRPA1;ITGA2;RDH10;APOE;AKR1C4;AGRN
Notch signaling pathway (GO:0007219)	0.05	-1.50	1.42	NOTCH3;ADAM17;NOTCH1;PSEN2;ADAM10
positive regulation of muscle tissue development (GO:1901863)	0.04	-1.37	1.41	EFNB2;NOTCH1;ERBB4
positive regulation of ubiquitin-protein transferase activity (GO:0051443)	0.04	-1.44	1.40	PSMD7;PIN1;PSMF1;CDC14B
cellular response to unfolded protein (GO:0034620)	0.04	-1.42	1.40	TSPYL2;IGFBP1;TATDN2;DDIT3
negative regulation of homeostatic	0.04	-1.40	1.38	DDIT3;TNFRSF11B;MIF;FADD

process (GO:0032845)				
diterpenoid metabolic process (GO:0016101)	0.04	-1.41	1.38	RDH10;AKR1C4;APOE;AGRN
positive regulation of chemotaxis (GO:0050921)	0.05	-1.45	1.37	ADAM17;ITGA2;ADAM10;HSPB1
detection of abiotic stimulus (GO:0009582)	0.05	-1.49	1.37	TRPA1;ITGA2;RDH10;APOE;AKR1C4;AGRN
DNA integrity checkpoint (GO:0031570)	0.04	-1.38	1.36	BRE;XPC;FOXO4;CDC14B
cell-type specific apoptotic process (GO:0097285)	0.04	-1.37	1.35	CASP9;DFFA;DNAJA3;FADD
DNA damage response, signal transduction by p53 class mediator (GO:0030330)	0.05	-1.41	1.31	PSMD7;PSMF1;MIF;FOXO3
iron ion homeostasis (GO:0055072)	0.05	-1.41	1.30	HMOX1;HAMP;CP;SOD1
phototransduction, visible light (GO:0007603)	0.05	-1.36	1.30	RDH10;AKR1C4;APOE;AGRN
positive regulation of ligase activity (GO:0051351)	0.05	-1.37	1.26	PSMD7;PIN1;PSMF1;CDC14B
cellular response to topologically incorrect protein (GO:0035967)	0.05	-1.34	1.24	TSPYL2;IGFBP1;TATDN2;DDIT3
alpha-amino acid catabolic process (GO:1901606)	0.05	-1.32	1.22	GSTZ1;GPT2;PCBD1;OTC
leukocyte homeostasis (GO:0001776)	0.04	-1.25	1.22	IKBKB;FADD;RC3H2
forebrain development (GO:0030900)	0.05	-1.18	1.13	NOTCH3;NOTCH1;PSEN2
regulation of interleukin-8 production (GO:0032677)	0.04	-1.15	1.10	ADAM17;DDIT3;FADD
negative regulation of myoblast differentiation (GO:0045662)	0.02	-0.89	1.01	NOTCH1;DDIT3
keratinocyte differentiation (GO:0030216)	0.05	-1.09	1.01	TSG101;NOTCH1;SAV1
**GO Cellular component**	**P-value**	**Z-score**	**Combined Score**	**Genes**
mitochondrial matrix (GO:0005759)	0.00	-2.34	5.23	GPX1;HSPA1L;ERBB4;TXNRD2;DNAJA3;GPT2;GSR;TK2;HSD17B10;SOD1;OTC
cell surface (GO:0009986)	0.00	-2.31	4.44	PRNP;NOTCH1;ITGA2;PSEN2;ADAM10;MIF;PCSK6;RC3H2;ADAM17;ADAM15;APMAP;CAPN5;TIMP2;AGRN;VAMP3;MBL2
blood microparticle (GO:0072562)	0.00	-2.57	3.70	HSPA1L;PROS1;ALB;HP;APOE;PFN1;CP;HSPA1A
cytosol (GO:0005829)	0.00	-2.06	2.97	SRXN1;HSPB1;IKBKB;CASP9;RPS16;PSMD7;PDE8B;CAST;NUDC;DFFA;PRKCH;GPX1;NFKBIL1;CTPS2;FNDC3A;SRP9;TOM1L1;CAT;BLVRB;PFN1;NOTCH3;NOTCH1;SAR1B;PSEN2;FOXO4;FOXO3;GSPT2;SAV1;PRDX4;ERBB4;HMOX1;PCBD1;FADD;PSMF1;EGLN1;YARS;SMAD1;STARD13;SEC24A;HSPA1L;ST13;CEP152;GSR;DHCR24;AKR1C4;PAICS;SOD1;DHFR;GSTZ1;DNAJA3;SPRY2;TJP2;PNPLA2;HSPA1A
membrane raft (GO:0045121)	0.01	-2.19	2.26	PRNP;ADAM17;RFTN1;PSEN2;PRKCDBP;HMOX1;FADD;TNFRSF1B
focal adhesion (GO:0005925)	0.02	-2.17	2.25	EFNB2;ADAM17;RPS16;ITGA2;CAT;CAPN5;HSPB1;NEXN;ADAM10;PFN1;HSPA1A
extracellular vesicular exosome (GO:0070062)	0.01	-2.17	2.24	SLC25A1;PROS1;HEXA;MAGEF1;HP;HSPB1;SERPINA5;RPS16;PSMD7;APMAP;CAPN5;C1RL;TIMP2;EFHD1;TMED7;PRKCH;GPX1;H2AFV;ADAM10;MIF;RENBP;SRP9;ZDHHC15;TPST2;TOM1L1;ADAM15;CAT;PXDN;BLVRB;PFN1;PLBD2;IGSF8;TSG101;XPC;ABHD6;PRDX4;CUX2;PCBP1;PCBD1;APOE;PRNP;CYB5A;ST13;GSR;RFTN1;AKR1C4;CP;PAICS;SOD1;GLB1;SERBP1;ALB;CPE;AGRN;HSPA1A
adherens junction (GO:0005912)	0.02	-2.13	2.20	EFNB2;ADAM17;RPS16;ITGA2;CAT;CAPN5;NEXN;SHROOM3;HSPB1;ADAM10;PFN1;HSPA1A
cell-substrate adherens junction (GO:0005924)	0.02	-2.13	2.20	EFNB2;ADAM17;RPS16;ITGA2;CAT;CAPN5;NEXN;HSPB1;ADAM10;PFN1;HSPA1A

cell-substrate junction (GO:0030055)	0.02	-2.10	2.18	EFNB2;ADAM17;RPS16;ITGA2;CAT;CAPN5;NEXN;HSPB1;ADAM10;PFN1;HSPA1A
cytoplasmic vesicle part (GO:0044433)	0.02	-2.10	2.17	CD163;SEC24A;SAR1B;PROS1;ALB;HP;CPE;APOE;TMED7;SERPINA5;VAMP3
nucleoplasm (GO:0005654)	0.02	-2.09	2.16	NOTCH3;NOTCH1;XPC;FOXO4;FOXO3;GATA1;CDC14B;PHF8;PSMD7;TATDN2;ERBB4;PCBP1;SUMO2;TCEB3;PSMF1;TEAD1;TSPYL2;SMAD1;NUDC;DFFA;SSRP1;DHFR;DDIT3;PIN1;TJP2
endoplasmic reticulum membrane (GO:0005789)	0.02	-2.08	2.15	NOTCH3;SEC24A;NOTCH1;SAR1B;PROS1;PSEN2;DHCR24;STIM1;RDH10;SCAP;HMOX1;SLC27A4;PNPLA2
Golgi apparatus (GO:0005794)	0.02	-2.07	2.14	PRNP;SAR1B;PSEN2;RHOBTB3;ADAM10;DHCR24;FNDC3A;ZDHHC15;TPST2;TOM1L1;ATXN2;GLB1;CAT;CPE;KIF1C;SCAP;APOE;ZNF622;TACC2;TMED7;TEAD1
anchoring junction (GO:0070161)	0.03	-2.04	2.06	EFNB2;ADAM17;RPS16;ITGA2;CAT;CAPN5;NEXN;SHROOM3;HSPB1;ADAM10;PFN1;HSPA1A
synapse (GO:0045202)	0.03	-1.86	1.83	CEP112;DNAJA3;TMEM57;PSEN2;COL4A5;APBB2;AGRN;VAMP3
cytoplasmic membrane-bounded vesicle lumen (GO:0060205)	0.03	-1.72	1.74	PROS1;ALB;HP;APOE
vesicle lumen (GO:0031983)	0.03	-1.70	1.71	PROS1;ALB;HP;APOE
CD40 receptor complex (GO:0035631)	0.02	-1.47	1.52	IKBKB;RNF31
IkappaB kinase complex (GO:0008385)	0.02	-1.46	1.51	IKBKB;DNAJA3
neuromuscular junction (GO:0031594)	0.04	-1.55	1.42	DNAJA3;PSEN2;COL4A5
COPII vesicle coat (GO:0030127)	0.01	-1.30	1.35	SEC24A;TMED7
extracellular space (GO:0005615)	0.05	-1.72	1.30	IGFBP1;YARS;CCL20;PROS1;HP;HSPB1;CXCL1;TNFRSF11B;MIF;PCSK6;CP;TSKU;SOD1;SERPINA5;PRDX4;ALB;C1RL;PXDN;TCEB3;TIMP2;HMOX1;APOE;AGRN;MBL2
extracellular matrix part (GO:0044420)	0.05	-1.65	1.27	SMOC1;ALB;TIMP2;COL4A5;AGRN
endocytic vesicle lumen (GO:0071682)	0.03	-1.18	1.19	HP;APOE
basal lamina (GO:0005605)	0.03	-1.17	1.18	COL4A5;AGRN
**GO Molecular functions**	**P-value**	**Z-score**	**Combined Score**	**Genes involved**
antioxidant activity (GO:0016209)	0.00	-2.31	39.98	GPX1;SRXN1;TXNRD2;GSR;HP;CYGB;SOD1;GSTZ1;PRDX4;ALB;PXDN;CAT;APOE
oxidoreductase activity, acting on peroxide as acceptor (GO:0016684)	0.00	-2.44	12.41	GSTZ1;PRDX4;GPX1;CAT;PXDN;CYGB
peroxidase activity (GO:0004601)	0.00	-2.41	12.30	GSTZ1;PRDX4;GPX1;CAT;PXDN;CYGB
chaperone binding (GO:0051087)	0.00	-2.21	7.92	PRNP;BAG2;ST13;ALB;CP;SOD1
aldo-keto reductase (NADP) activity (GO:0004033)	0.00	-3.10	5.05	CYB5A;RDH10;AKR1C4
chromatin DNA binding (GO:0031490)	0.00	-2.10	3.42	SMARCD2;NOTCH1;FOXO3;MTA2;GATA1
protein kinase binding (GO:0019901)	0.00	-2.47	4.03	SMAD1;KSR1;CEP152;HSPB1;ADAM10;FOXO3;CASP9;IKBKB;TOM1L1;DNAJA3;PRKCDBP;PIN1;JTB;SPRY2
cytokine receptor binding (GO:0005126)	0.00	-2.36	3.37	YARS;ADAM17;BRE;CCL20;DNAJA3;PXDN;CXCL1;MIF;FADD
ubiquitin binding (GO:0043130)	0.00	-2.29	3.27	RNF31;TOM1L1;TSG101;BRE;HSPB1
NADP binding (GO:0050661)	0.01	-2.26	3.19	DHFR;TXNRD2;GSR;CAT
SH3 domain binding (GO:0017124)	0.01	-2.16	3.04	TOM1L1;CASP9;ADAM17;GPX1;ADAM15;ADAM10
copper ion binding (GO:0005507)	0.01	-2.04	2.88	PRNP;ALB;CP;SOD1
protein serine/threonine kinase inhibitor activity (GO:0030291)	0.01	-2.49	3.51	SPRY2;HSPB1;PKIG
kinase binding (GO:0019900)	0.01	-2.40	3.38	SMAD1;KSR1;CEP152;HSPB1;ADAM10;FOXO3;IKBKB;CASP9;TOM1L1;DNAJA3;PRKCDBP;JTB;PIN1;SPRY2

small conjugating protein binding (GO:0032182)	0.01	-2.28	3.21	RNF31;TOM1L1;TSG101;BRE;HSPB1
receptor signaling protein tyrosine kinase activity (GO:0004716)	0.01	-1.07	1.48	ERBB4;ERBB2
oxidoreductase activity, acting on a sulfur group of donors, NAD(P) as acceptor (GO:0016668)	0.01	-1.26	1.60	TXNRD2;GSR
heme binding (GO:0020037)	0.01	-2.12	2.47	CYB5A;PXDN;CAT;FLVCR2;HMOX1;CYGB
unfolded protein binding (GO:0051082)	0.01	-2.06	2.33	HSPA1L;ST13;DNAJA3;SCAP;HSPA1A
integrin binding (GO:0005178)	0.01	-2.08	2.35	ADAM17;ADAM15;ITGA2;TIMP2;ADAM10
tetrapyrrole binding (GO:0046906)	0.02	-2.10	2.20	CYB5A;PXDN;CAT;FLVCR2;HMOX1;CYGB
enzyme inhibitor activity (GO:0004857)	0.02	-2.28	2.31	CAST;NOTCH1;PROS1;TIMP2;HSPB1;SPRY2;PSMF1;PKIG;RENBP;SERPINA5
mitogen-activated protein kinase kinase binding (GO:0031434)	0.02	-1.57	1.58	KSR1;PIN1
mRNA binding (GO:0003729)	0.02	-2.03	2.00	DHFR;ESRP2;SERBP1;SNRPC;RC3H2
carboxylic acid binding (GO:0031406)	0.02	-2.10	2.07	EGLN1;GPX1;ALB;PIN1;AGRN;SERPINA5;OTC
organic acid binding (GO:0043177)	0.02	-2.10	2.07	EGLN1;GPX1;ALB;PIN1;AGRN;SERPINA5;OTC
oxidoreductase activity, acting on the CH-OH group of donors, NAD or NADP as acceptor (GO:0016616)	0.02	-1.98	1.95	CYB5A;RDH10;DHCR24;AKR1C4;HSD17B10
endopeptidase inhibitor activity (GO:0004866)	0.03	-2.06	1.90	CAST;PROS1;TIMP2;PSMF1;RENBP;SERPINA5
glutathione peroxidase activity (GO:0004602)	0.03	-1.68	1.55	GSTZ1;GPX1
mannose binding (GO:0005537)	0.03	-1.55	1.43	MANBA;MBL2
growth factor binding (GO:0019838)	0.03	-1.97	1.82	IGFBP1;ERBB2;PXDN;PCSK6;LTBP1
cell adhesion molecule binding (GO:0050839)	0.03	-1.96	1.82	ADAM17;ADAM15;ITGA2;TIMP2;CPE;ADAM10
oxidoreductase activity, acting on the CH-NH group of donors, NAD or NADP as acceptor (GO:0016646)	0.03	-1.45	1.34	DHFR;BLVRB
proline-rich region binding (GO:0070064)	0.03	-1.44	1.33	WBP4;PFN1
endopeptidase regulator activity (GO:0061135)	0.03	-1.97	1.82	CAST;PROS1;TIMP2;PSMF1;RENBP;SERPINA5
oxidoreductase activity, acting on a sulfur group of donors (GO:0016667)	0.03	-1.60	1.48	SRXN1;TXNRD2;GSR
peptidase inhibitor activity (GO:0030414)	0.03	-1.97	1.82	CAST;PROS1;TIMP2;PSMF1;RENBP;SERPINA5
nucleobase-containing compound kinase activity (GO:0019205)	0.03	-1.72	1.60	CMPK2;TK2;TJP2
extracellular matrix binding (GO:0050840)	0.04	-1.65	1.50	ITGA2;SMOC1;AGRN
oxidoreductase activity, acting on CH-OH group of donors (GO:0016614)	0.04	-1.81	1.65	CYB5A;RDH10;DHCR24;AKR1C4;HSD17B10
nucleotide kinase activity (GO:0019201)	0.04	-1.36	1.23	CMPK2;TJP2
Hsp70 protein binding (GO:0030544)	0.04	-1.36	1.23	ST13;DNAJA3
protein kinase inhibitor activity (GO:0004860)	0.04	-1.63	1.47	SPRY2;HSPB1;PKIG
small conjugating protein ligase binding (GO:0044389)	0.04	-1.85	1.66	RNF31;TSG101;SUMO2;TNFRSF1B;CCDC50;HSPA1A
ubiquitin protein ligase binding (GO:0031625)	0.04	-1.83	1.65	RNF31;TSG101;SUMO2;TNFRSF1B;CCDC50;HSPA1A

oxidoreductase activity, acting on the CH-CH group of donors, NAD or NADP as acceptor (GO:0016628)	0.04	-1.18	1.02	BLVRB;DHCR24
kinase inhibitor activity (GO:0019210)	0.05	-1.53	1.30	SPRY2;HSPB1;PKIG
chemokine receptor binding (GO:0042379)	0.05	-1.48	1.26	YARS;CCL20;CXCL1
**KEGG PATHWAYS**	**P-value**	**Z-score**	**Combined Score**	**Genes**
Amyotrophic lateral sclerosis (ALS)_Homo sapiens_hsa05014	0.00	-1.86	6.48	CASP9;GPX1;CAT;TNFRSF1B;TOMM40L;SOD1
Legionellosis_Homo sapiens_hsa05134	0.00	-1.88	6.53	CASP9;HSPA1L;SAR1B;CXCL1;HSPA1B;HSPA1A
Longevity regulating pathway - multiple species_Homo sapiens_hsa04213	0.00	-1.94	6.09	HSPA1L;CAT;FOXO3;HSPA1B;HSPA1A;SOD1
Prion diseases_Homo sapiens_hsa05020	0.00	-1.68	3.39	PRNP;NOTCH1;SOD1;HSPA1A
Other glycan degradation_Homo sapiens_hsa00511	0.00	-1.33	2.61	MANBA;GLB1;HEXA
Alzheimer's disease_Homo sapiens_hsa05010	0.01	-1.72	2.94	CASP9;ADAM17;COX4I2;PSEN2;ADAM10;APOE;FADD;HSD17B10
Notch signaling pathway_Homo sapiens_hsa04330	0.01	-1.50	2.32	NOTCH3;ADAM17;NOTCH1;PSEN2
Protein processing in endoplasmic reticulum_Homo sapiens_hsa04141	0.02	-1.65	1.36	SEC24A;HSPA1L;BAG2;SAR1B;DDIT3;HSPA1B;HSPA1A
Epithelial cell signaling in Helicobacter pylori infection_Homo sapiens_hsa05120	0.03	-1.66	1.20	IKBKB;ADAM17;ADAM10;CXCL1
Glycosphingolipid biosynthesis - ganglio series_Homo sapiens_hsa00604	0.03	-0.63	0.45	GLB1;HEXA
Porphyrin and chlorophyll metabolism_Homo sapiens_hsa00860	0.03	-1.33	0.95	BLVRB;HMOX1;CP
TNF signaling pathway_Homo sapiens_hsa04668	0.03	-1.62	1.16	IKBKB;CCL20;CXCL1;FADD;TNFRSF1B
Glycosaminoglycan degradation_Homo sapiens_hsa00531	0.04	-0.35	0.22	GLB1;HEXA
Toxoplasmosis_Homo sapiens_hsa05145	0.04	-1.57	1.02	CASP9;IKBKB;HSPA1L;HSPA1B;HSPA1A
Arginine biosynthesis_Homo sapiens_hsa00220	0.05	-0.40	0.26	GPT2;OTC
**REACTOME PATHWAYS**	**P-value**	**Z-score**	**Combined Score**	**Genes**
Detoxification of Reactive Oxygen Species_Homo sapiens_R-HSA-3299685	0.00	-2.33	6.38	GPX1;TXNRD2;GSR;CAT;SOD1
Receptor-ligand binding initiates the second proteolytic cleavage of Notch receptor_Homo sapiens_R-HSA-156988	0.00	-1.99	5.44	NOTCH3;ADAM17;NOTCH1;ADAM10
A third proteolytic cleavage releases NICD_Homo sapiens_R-HSA-157212	0.00	-1.91	4.55	NOTCH3;NOTCH1;PSEN2
Signaling by NOTCH1 t(7;9)(NOTCH1:M1580_K2555) Translocation Mutant_Homo sapiens_R-HSA-2660825	0.00	-1.47	4.04	ADAM17;NOTCH1;ADAM10
Constitutive Signaling by NOTCH1 t(7;9)(NOTCH1:M1580_K2555) Translocation Mutant_Homo sapiens_R-HSA-2660826	0.00	-1.41	3.86	ADAM17;NOTCH1;ADAM10

Signaling by NOTCH3_Homo sapiens_R-HSA-1980148	0.00	-1.75	3.65	NOTCH3;PSEN2;ADAM10
Activated NOTCH1 Transmits Signal to the Nucleus_Homo sapiens_R-HSA-2122948	0.00	-1.99	3.62	ADAM17;NOTCH1;PSEN2;ADAM10
Metabolism of nucleotides_Homo sapiens_R-HSA-15869	0.00	-1.96	3.18	GPX1;GSR;CAT;CTPS2;TK2;PAICS
Diseases of signal transduction_Homo sapiens_R-HSA-5663202	0.01	-2.31	3.13	CASP9;ADAM17;PSMD7;NOTCH1;ERBB4;ERBB2;PSEN2;ADAM10;PSMF1;FOXO4;FOXO3
Signaling by NOTCH1 HD Domain Mutants in Cancer_Homo sapiens_R-HSA-2691230	0.00	-1.81	3.12	ADAM17;NOTCH1;ADAM10
Downstream signaling events of B Cell Receptor (BCR)_Homo sapiens_R-HSA-1168372	0.01	-2.29	3.10	CASP9;IKBKB;PSMD7;ERBB4;ERBB2;FOXO4;PSMF1;FOXO3
Constitutive Signaling by NOTCH1 HD Domain Mutants_Homo sapiens_R-HSA-2691232	0.00	-1.80	3.10	ADAM17;NOTCH1;ADAM10
Signaling by the B Cell Receptor (BCR)_Homo sapiens_R-HSA-983705	0.01	-2.26	3.07	IKBKB;CASP9;PSMD7;STIM1;ERBB4;ERBB2;PSMF1;FOXO4;FOXO3
Disease_Homo sapiens_R-HSA-1643685	0.01	-2.23	3.02	NOTCH3;NOTCH1;TSG101;PSEN2;ADAM10;FOXO4;SSRP1;FOXO3;IKBKB;CASP9;ADAM17;PSMD7;RPS16;ERBB4;ALB;ERBB2;TCEB3;PSMF1;AGRN;HSPA1A
TNF signaling_Homo sapiens_R-HSA-75893	0.00	-1.99	2.69	RNF31;IKBKB;ADAM17;FADD
AUF1 (hnRNP D0) binds and destabilizes mRNA_Homo sapiens_R-HSA-450408	0.01	-1.93	2.61	PSMD7;HSPB1;PSMF1;HSPA1A
Attenuation phase_Homo sapiens_R-HSA-3371568	0.01	-1.92	2.59	HSPA1L;HSPA1B;HSPA1A
Death Receptor Signalling_Homo sapiens_R-HSA-73887	0.01	-1.89	2.56	RNF31;IKBKB;ADAM17;FADD
MAPK6/MAPK4 signaling_Homo sapiens_R-HSA-5687128	0.02	-1.90	2.54	PSMD7;HSPB1;PSMF1;FOXO3;CDC14B
Degradation of the extracellular matrix_Homo sapiens_R-HSA-1474228	0.01	-1.87	2.53	CAST;ADAM17;ADAM15;CAPN5;TIMP2;ADAM10
Signaling by EGFR_Homo sapiens_R-HSA-177929	0.03	-2.14	2.51	CASP9;ADAM17;PSMD7;KSR1;ERBB4;ERBB2;ADAM10;SPRY2;PSMF1;FOXO4;FOXO3
Extracellular matrix organization_Homo sapiens_R-HSA-1474244	0.01	-1.83	2.46	CAST;ADAM17;ADAM15;ITGA2;CAPN5;TIMP2;ADAM10;COL4A5;AGRN;LTBP1
Apoptosis_Homo sapiens_R-HSA-109581	0.02	-1.84	2.46	CASP9;DFFA;PSMD7;MAGED1;PSMF1;FADD;TJP2
Programmed Cell Death_Homo sapiens_R-HSA-5357801	0.02	-1.83	2.44	CASP9;DFFA;PSMD7;MAGED1;PSMF1;FADD;TJP2
Regulation of HSF1-mediated heat shock response_Homo sapiens_R-HSA-3371453	0.01	-1.80	2.44	HSPA1L;BAG2;ST13;HSPA1B;HSPA1A
Signalling by NGF_Homo sapiens_R-HSA-166520	0.03	-2.01	2.36	KSR1;MAGED1;PSEN2;FOXO4;FOXO3;PCSK6;IKBKB;CASP9;ADAM17;PSMD7;ERBB4;ERBB2;PSMF1
Regulation of cholesterol biosynthesis by SREBP (SREBF)_Homo sapiens_R-HSA-1655829	0.01	-1.72	2.33	SEC24A;SCD;SAR1B;SCAP
PI3K/AKT Signaling in Cancer_Homo sapiens_R-HSA-2219528	0.01	-1.69	2.29	CASP9;ERBB4;ERBB2;FOXO4;FOXO3
HSF1 activation_Homo sapiens_R-HSA-3371511	0.01	-1.67	2.27	HSPA1L;HSPA1B;HSPA1A
Caspase activation via extrinsic apoptotic signalig pathway_Homo sapiens_R-HSA-5357769	0.01	-1.67	2.26	CASP9;MAGED1;FADD
Signaling by NOTCH1 in	0.01	-1.62	2.20	ADAM17;NOTCH1;PSEN2;ADAM10

Cancer_Homo sapiens_R-HSA-2644603				
Cellular response to heat stress_Homo sapiens_R-HSA-3371556	0.02	-1.66	2.19	HSPA1L;BAG2;ST13;HSPA1B;HSPA1A
Constitutive Signaling by NOTCH1 HD+PEST Domain Mutants_Homo sapiens_R-HSA-2894862	0.01	-1.62	2.19	ADAM17;NOTCH1;PSEN2;ADAM10
Signaling by NOTCH1 HD+PEST Domain Mutants in Cancer_Homo sapiens_R-HSA-2894858	0.01	-1.60	2.17	ADAM17;NOTCH1;PSEN2;ADAM10
HSF1-dependent transactivation_Homo sapiens_R-HSA-3371571	0.02	-1.62	2.17	HSPA1L;HSPA1B;HSPA1A
Constitutive Signaling by NOTCH1 PEST Domain Mutants_Homo sapiens_R-HSA-2644606	0.01	-1.59	2.16	ADAM17;NOTCH1;PSEN2;ADAM10
Signaling by NOTCH1 PEST Domain Mutants in Cancer_Homo sapiens_R-HSA-2644602	0.01	-1.59	2.15	ADAM17;NOTCH1;PSEN2;ADAM10
Constitutive Signaling by AKT1 E17K in Cancer_Homo sapiens_R-HSA-5674400	0.01	-1.57	2.12	CASP9;FOXO4;FOXO3
Cellular responses to stress_Homo sapiens_R-HSA-2262752	0.03	-1.86	2.03	EGLN1;GPX1;HSPA1L;BAG2;ST13;TXNRD2;GSR;CAT;HSPA1B;HSPA1A;SOD1
Signaling by ERBB4_Homo sapiens_R-HSA-1236394	0.04	-1.98	2.03	CASP9;ADAM17;PSMD7;KSR1;ERBB4;ERBB2;PSEN2;PSMF1;FOXO4;FOXO3
Lipoprotein metabolism_Homo sapiens_R-HSA-174824	0.02	-1.40	1.86	SAR1B;ALB;APOE
Purine metabolism_Homo sapiens_R-HSA-73847	0.02	-1.36	1.81	GPX1;CAT;PAICS
Signaling by NOTCH1_Homo sapiens_R-HSA-1980143	0.03	-1.48	1.61	ADAM17;NOTCH1;PSEN2;ADAM10
NCAM1 interactions_Homo sapiens_R-HSA-419037	0.02	-1.30	1.60	PRNP;COL4A5;AGRN
Nuclear signaling by ERBB4_Homo sapiens_R-HSA-1251985	0.03	-1.35	1.56	ADAM17;ERBB4;PSEN2
PI-3K cascade:FGFR1_Homo sapiens_R-HSA-5654689	0.05	-1.53	1.55	CASP9;ERBB4;ERBB2;FOXO4;FOXO3
PI-3K cascade:FGFR2_Homo sapiens_R-HSA-5654695	0.05	-1.52	1.54	CASP9;ERBB4;ERBB2;FOXO4;FOXO3
PIP3 activates AKT signaling_Homo sapiens_R-HSA-1257604	0.05	-1.51	1.54	CASP9;ERBB4;ERBB2;FOXO4;FOXO3
PI-3K cascade:FGFR3_Homo sapiens_R-HSA-5654710	0.05	-1.50	1.53	CASP9;ERBB4;ERBB2;FOXO4;FOXO3
PI-3K cascade:FGFR4_Homo sapiens_R-HSA-5654720	0.05	-1.49	1.52	CASP9;ERBB4;ERBB2;FOXO4;FOXO3
PI3K events in ERBB4 signaling_Homo sapiens_R-HSA-1250342	0.05	-1.48	1.51	CASP9;ERBB4;ERBB2;FOXO4;FOXO3
GAB1 signalosome_Homo sapiens_R-HSA-180292	0.05	-1.48	1.50	CASP9;ERBB4;ERBB2;FOXO4;FOXO3
PI3K/AKT activation_Homo sapiens_R-HSA-198203	0.05	-1.47	1.49	CASP9;ERBB4;ERBB2;FOXO4;FOXO3
Binding and Uptake of Ligands by Scavenger Receptors_Homo sapiens_R-HSA-2173782	0.03	-1.32	1.44	CD163;ALB;HP;APOE
Regulation of mRNA stability by proteins that bind AU-rich elements_Homo sapiens_R-HSA-450531	0.05	-1.39	1.40	PSMD7;HSPB1;PSMF1;HSPA1A
Signaling by NOTCH_Homo sapiens_R-HSA-157118	0.05	-1.35	1.38	NOTCH3;ADAM17;NOTCH1;PSEN2;ADAM10
Retinoid metabolism and	0.03	-1.19	1.30	AKR1C4;APOE;AGRN

transport_Homo sapiens_R-HSA-975634				
Unfolded Protein Response (UPR)_Homo sapiens_R-HSA-381119	0.05	-1.28	1.29	IGFBP1;TSPYL2;TATDN2;DDIT3
EPH-ephrin mediated repulsion of cells_Homo sapiens_R-HSA-3928665	0.04	-1.21	1.23	EFNB2;PSEN2;ADAM10
Pre-NOTCH Processing in the Endoplasmic Reticulum_Homo sapiens_R-HSA-1912399	0.01	-0.90	1.21	NOTCH3;NOTCH1
p75 NTR receptor-mediated signalling_Homo sapiens_R-HSA-193704	0.04	-1.18	1.20	IKBKB;ADAM17;MAGED1;PSEN2
Heme degradation_Homo sapiens_R-HSA-189483	0.01	-0.85	1.16	HMOX1;BLVRB
Scavenging of heme from plasma_Homo sapiens_R-HSA-2168880	0.04	-1.10	1.12	CD163;ALB;HP
AKT phosphorylates targets in the nucleus_Homo sapiens_R-HSA-198693	0.01	-0.75	1.01	FOXO4;FOXO3

Downregulated genes in liver biopsy of GR.A:SAHIO vs. GR.B:SAHNIO

GO Biological process	P-value	Z-score	Combined Score	Genes
regulation of interleukin-2 biosynthetic process (GO:0045076)	0.00	-2.90	5.28	IL1B;SFTPD
glycoside metabolic process (GO:0016137)	0.00	-2.87	5.24	AKR1C2;GLA
superoxide anion generation (GO:0042554)	0.00	-2.81	5.13	NCF2;PRG3
positive regulation of monooxygenase activity (GO:0032770)	0.00	-2.71	4.94	FCER2;IL1B
amyloid precursor protein metabolic process (GO:0042982)	0.00	-2.68	4.89	PSENEN;NCSTN
Notch receptor processing (GO:0007220)	0.00	-2.67	4.87	PSENEN;NCSTN
membrane protein intracellular domain proteolysis (GO:0031293)	0.00	-2.64	4.82	PSENEN;NCSTN
positive regulation vascular endothelial growth factor production (GO:0010575)	0.00	-2.57	4.68	IL1B;SULF1
membrane protein ectodomain proteolysis (GO:0006509)	0.00	-2.55	4.65	PSENEN;NCSTN
regulation of vascular endothelial growth factor production (GO:0010574)	0.00	-2.60	4.33	IL1B;SULF1
regulation of cytokine biosynthetic process (GO:0042035)	0.00	-2.32	4.23	IL1B;SFTPD;PRG3
regulation of monooxygenase activity (GO:0032768)	0.00	-2.30	4.19	FCER2;IL1B;GLA
amyloid precursor protein catabolic process (GO:0042987)	0.00	-2.24	4.09	PSENEN;NCSTN
reactive oxygen species metabolic process (GO:0072593)	0.00	-2.23	4.06	NCF2;SFTPD;PRG3
regulation of oxidoreductase activity (GO:0051341)	0.00	-2.20	4.01	FCER2;IL1B;GLA
membrane protein proteolysis (GO:0033619)	0.00	-2.51	4.01	PSENEN;NCSTN

response to lipopolysaccharide (GO:0032496)	0.00	-2.37	3.78	NCF2;IL1B;IL13;PLCG2
superoxide metabolic process (GO:0006801)	0.00	-2.37	3.77	NCF2;PRG3
regulation of nitric-oxide synthase activity (GO:0050999)	0.01	-2.48	3.69	FCER2;GLA
positive regulation of oxidoreductase activity (GO:0051353)	0.01	-2.43	3.63	FCER2;IL1B
regulation of cytokine production (GO:0001817)	0.01	-2.42	3.61	IL1B;IL13;SFTPD;PRG3;SULF1
response to molecule of bacterial origin (GO:0002237)	0.00	-2.39	3.59	NCF2;IL1B;IL13;PLCG2
negative regulation of epithelial cell proliferation (GO:0050680)	0.00	-2.25	3.58	MED1;PLA2G2A;SULF1
positive regulation of cytokine production (GO:0001819)	0.01	-2.35	3.51	IL1B;IL13;PRG3;SULF1
regulation of interleukin-2 production (GO:0032663)	0.01	-2.34	3.49	IL1B;SFTPD
positive regulation of interleukin-8 production (GO:0032757)	0.01	-2.33	3.47	IL1B;PRG3
positive regulation of intracellular transport (GO:0032388)	0.02	-2.32	3.46	MED1;IL1B;IL13
regulation of lymphocyte proliferation (GO:0050670)	0.01	-2.32	3.46	IL1B;IL13;SFTPD
regulation of mononuclear cell proliferation (GO:0032944)	0.01	-2.31	3.45	IL1B;IL13;SFTPD
regulation of leukocyte proliferation (GO:0070663)	0.01	-2.31	3.44	IL1B;IL13;SFTPD
positive regulation of immune effector process (GO:0002699)	0.01	-2.27	3.39	FCER2;IL1B;IL13
positive regulation of ion transport (GO:0043270)	0.01	-2.27	3.39	IL1B;IL13;PLCG2
regulation of nitric oxide biosynthetic process (GO:0045428)	0.01	-2.27	3.38	IL1B;GLA
positive regulation of protein transport (GO:0051222)	0.03	-2.26	3.36	MED1;IL1B;IL13
Notch signaling pathway (GO:0007219)	0.01	-2.21	3.30	PSENEN;FCER2;NCSTN
ribonucleoprotein complex assembly (GO:0022618)	0.01	-2.20	3.28	EIF3K;CLNS1A;PATL1
regulation of angiogenesis (GO:0045765)	0.01	-2.20	3.28	IL1B;SULF1;GTF2I
ribonucleoprotein complex subunit organization (GO:0071826)	0.01	-2.19	3.27	EIF3K;CLNS1A;PATL1
regulation of vasculature development (GO:1901342)	0.02	-2.19	3.27	IL1B;SULF1;GTF2I
positive regulation of leukocyte mediated immunity (GO:0002705)	0.02	-2.17	3.24	FCER2;IL1B
positive regulation of adaptive immune response based on somatic recombination of immune receptors built from immunoglobulin superfamily domains (GO:0002824)	0.02	-2.17	3.23	FCER2;IL1B
positive regulation of adaptive immune response (GO:0002821)	0.02	-2.16	3.23	FCER2;IL1B
protein folding (GO:0006457)	0.02	-2.16	3.22	CSNK2A1;TXNDC2;HSCB
organophosphate biosynthetic process (GO:0090407)	0.03	-2.16	3.21	PLA2G2A;PLCG2;NME5;ATP5F1
positive regulation of epithelial cell differentiation (GO:0030858)	0.01	-2.15	3.21	MED1;IL13
transcription initiation from RNA polymerase II promoter (GO:0006367)	0.01	-2.15	3.21	GTF2A1L;MED1;GTF2I

DNA-templated transcription, initiation (GO:0006352)	0.02	-2.15	3.21	GTF2A1L;MED1;GTF2I
regulation of epithelial cell proliferation (GO:0050678)	0.03	-2.15	3.21	MED1;PLA2G2A;SULF1
positive regulation of lymphocyte mediated immunity (GO:0002708)	0.02	-2.15	3.21	FCER2;IL1B
positive regulation of cytokine biosynthetic process (GO:0042108)	0.01	-2.14	3.19	IL1B;PRG3
purine ribonucleoside triphosphate biosynthetic process (GO:0009206)	0.01	-2.14	3.19	NME5;ATP5F1
regulation of immune effector process (GO:0002697)	0.04	-2.12	3.16	FCER2;IL1B;IL13
multicellular organismal homeostasis (GO:0048871)	0.01	-2.12	3.16	IL1B;SLC40A1
purine nucleoside triphosphate biosynthetic process (GO:0009145)	0.01	-2.11	3.15	NME5;ATP5F1
positive regulation of protein import into nucleus (GO:0042307)	0.02	-2.10	3.14	MED1;IL1B
ribonucleoside triphosphate biosynthetic process (GO:0009201)	0.01	-2.10	3.13	NME5;ATP5F1
positive regulation of protein localization to nucleus (GO:1900182)	0.02	-2.09	3.12	MED1;IL1B
positive regulation of Wnt signaling pathway (GO:0030177)	0.02	-2.09	3.12	CSNK2A1;SULF1
lipid catabolic process (GO:0016042)	0.03	-2.08	3.10	PLA2G2A;PLCG2;GLA
epithelial cell proliferation (GO:0050673)	0.02	-2.08	3.09	MED1;NCSTN
negative regulation of blood vessel morphogenesis (GO:2000181)	0.01	-2.07	3.08	SULF1;GTF2I
cellular response to mechanical stimulus (GO:0071260)	0.02	-2.06	3.08	IL1B;IL13
myeloid leukocyte migration (GO:0097529)	0.02	-2.06	3.08	IL1B;SFTPD
negative regulation of angiogenesis (GO:0016525)	0.01	-2.06	3.07	SULF1;GTF2I
positive regulation of inflammatory response (GO:0050729)	0.02	-2.06	3.07	IL1B;PLA2G2A
positive regulation of defense response (GO:0031349)	0.04	-2.05	3.06	MED1;IL1B;PLA2G2A
positive regulation of calcium ion transport (GO:0051928)	0.03	-2.05	3.06	IL13;PLCG2
multicellular organismal reproductive process (GO:0048609)	0.04	-2.05	3.06	MED1;TXNDC2;NME5;ZSCAN2
negative regulation of vasculature development (GO:1901343)	0.02	-2.05	3.06	SULF1;GTF2I
nucleoside triphosphate biosynthetic process (GO:0009142)	0.02	-2.04	3.04	NME5;ATP5F1
cellular hormone metabolic process (GO:0034754)	0.03	-2.03	3.03	MED1;AKR1C2
regulation of interleukin-8 production (GO:0032677)	0.01	-2.03	3.03	IL1B;PRG3
transcription from RNA polymerase II promoter (GO:0006366)	0.04	-2.02	3.02	GTF2A1L;MED1;ZNF473;GTF2I
regulation of lymphocyte mediated immunity (GO:0002706)	0.03	-2.02	3.01	FCER2;IL1B
gene expression (GO:0010467)	0.04	-2.01	3.00	MED1;ZNF473;EIF3K;CLNS1A;PATL1
positive regulation of nucleocytoplasmic transport (GO:0046824)	0.03	-2.01	2.99	MED1;IL1B
homeostasis of number of cells (GO:0048872)	0.03	-2.00	2.99	NCSTN;SLC40A1

regulation of adaptive immune response based on somatic recombination of immune receptors built from immunoglobulin superfamily domains (GO:0002822)	0.03	-1.98	2.95	FCER2;IL1B
icosanoid metabolic process (GO:0006690)	0.02	-1.96	2.93	AKR1C2;PRG3
fatty acid derivative metabolic process (GO:1901568)	0.02	-1.96	2.92	AKR1C2;PRG3
regulation of epithelial cell differentiation (GO:0030856)	0.04	-1.93	2.88	MED1;IL13
purine nucleoside biosynthetic process (GO:0042451)	0.03	-1.93	2.87	NME5;ATP5F1
purine ribonucleoside biosynthetic process (GO:0046129)	0.03	-1.92	2.87	NME5;ATP5F1
regulation of adaptive immune response (GO:0002819)	0.04	-1.91	2.85	FCER2;IL1B
lipid biosynthetic process (GO:0008610)	0.04	-1.91	2.84	MED1;PLA2G2A;PLCG2;PRG3
unsaturated fatty acid metabolic process (GO:0033559)	0.03	-1.89	2.81	AKR1C2;PRG3
leukocyte chemotaxis (GO:0030595)	0.03	-1.88	2.80	IL1B;SFTPD
ribonucleoside biosynthetic process (GO:0042455)	0.04	-1.83	2.72	NME5;ATP5F1
regulation of leukocyte mediated immunity (GO:0002703)	0.04	-1.82	2.71	FCER2;IL1B
positive regulation of lymphocyte proliferation (GO:0050671)	0.04	-1.81	2.71	IL1B;IL13
positive regulation of mononuclear cell proliferation (GO:0032946)	0.04	-1.81	2.70	IL1B;IL13
positive regulation of leukocyte proliferation (GO:0070665)	0.04	-1.75	2.61	IL1B;IL13
regulation of T cell proliferation (GO:0042129)	0.05	-1.74	2.58	IL1B;SFTPD
nucleoside biosynthetic process (GO:0009163)	0.04	-1.72	2.57	NME5;ATP5F1
glycosyl compound biosynthetic process (GO:1901659)	0.04	-1.72	2.56	NME5;ATP5F1
phosphatidylinositol metabolic process (GO:0046488)	0.05	-1.73	2.56	PLA2G2A;PLCG2
purine ribonucleotide biosynthetic process (GO:0009152)	0.05	-1.71	2.53	NME5;ATP5F1
cellular biogenic amine metabolic process (GO:0006576)	0.05	-1.69	2.51	PLA2G2A;PRG3
cellular amine metabolic process (GO:0044106)	0.05	-1.69	2.50	PLA2G2A;PRG3
positive regulation of icosanoid secretion (GO:0032305)	0.03	-0.97	1.44	IL1B
negative regulation of fibroblast growth factor receptor signaling pathway (GO:0040037)	0.04	-0.96	1.43	SULF1
positive regulation of fatty acid transport (GO:2000193)	0.04	-0.93	1.39	IL1B
regulation of icosanoid secretion (GO:0032303)	0.04	-0.93	1.39	IL1B
regulation of chemokine biosynthetic process (GO:0045073)	0.04	-0.93	1.38	IL1B
regulation of interleukin-8 biosynthetic process (GO:0045414)	0.03	-0.92	1.37	PRG3
positive regulation of interleukin-2 biosynthetic process (GO:0045086)	0.04	-0.91	1.36	IL1B
regulation of fever generation (GO:0031620)	0.02	-0.91	1.35	IL1B

positive regulation of humoral immune response (GO:0002922)	0.04	-0.91	1.35	FCER2
negative regulation of interleukin-2 production (GO:0032703)	0.04	-0.90	1.34	SFTPD
regulation of mammary gland epithelial cell proliferation (GO:0033599)	0.04	-0.90	1.34	MED1
regulation of proton transport (GO:0010155)	0.04	-0.90	1.34	IL13
renal filtration (GO:0097205)	0.04	-0.89	1.33	SULF1
negative regulation of nitric oxide biosynthetic process (GO:0045019)	0.03	-0.88	1.32	GLA
positive regulation of protein export from nucleus (GO:0046827)	0.04	-0.88	1.31	IL1B
positive regulation of epidermal cell differentiation (GO:0045606)	0.04	-0.87	1.29	MED1
regulation of humoral immune response mediated by circulating immunoglobulin (GO:0002923)	0.03	-0.86	1.28	FCER2
thyroid hormone generation (GO:0006590)	0.04	-0.85	1.26	MED1
mRNA transcription from RNA polymerase II promoter (GO:0042789)	0.04	-0.85	1.26	MED1
positive regulation of macrophage derived foam cell differentiation (GO:0010744)	0.04	-0.85	1.26	PLA2G2A
positive regulation of nitric-oxide synthase activity (GO:0051000)	0.04	-0.85	1.26	FCER2
lipid particle organization (GO:0034389)	0.04	-0.84	1.25	FAF2
chemical homeostasis within a tissue (GO:0048875)	0.03	-0.82	1.23	SFTPD
glycosylceramide metabolic process (GO:0006677)	0.04	-0.82	1.23	GLA
trabecula morphogenesis (GO:0061383)	0.04	-0.82	1.22	MED1
heart trabecula morphogenesis (GO:0061384)	0.03	-0.82	1.22	MED1
ceramide catabolic process (GO:0046514)	0.04	-0.82	1.22	GLA
glomerular filtration (GO:0003094)	0.03	-0.82	1.22	SULF1
megakaryocyte development (GO:0035855)	0.04	-0.81	1.21	MED1
regulation of granulocyte macrophage colony-stimulating factor production (GO:0032645)	0.03	-0.81	1.21	IL1B
mature B cell differentiation (GO:0002335)	0.03	-0.80	1.20	PLCG2
response to laminar fluid shear stress (GO:0034616)	0.04	-0.80	1.20	NCF2
positive regulation of vascular endothelial growth factor receptor signaling pathway (GO:0030949)	0.05	-0.80	1.19	IL1B
receptor recycling (GO:0001881)	0.02	-0.80	1.19	CAMLG
positive regulation of granulocyte macrophage colony-stimulating factor production (GO:0032725)	0.03	-0.79	1.18	IL1B
regulation of vitamin D biosynthetic process (GO:0060556)	0.03	-0.79	1.17	IL1B
single strand break repair (GO:0000012)	0.05	-0.78	1.17	XRCC1

positive regulation of keratinocyte differentiation (GO:0045618)	0.03	-0.78	1.17	MED1
regulation of prostaglandin secretion (GO:0032306)	0.03	-0.78	1.16	IL1B
regulation of vitamin metabolic process (GO:0030656)	0.03	-0.77	1.16	IL1B
positive regulation of membrane protein ectodomain proteolysis (GO:0051044)	0.04	-0.77	1.15	IL1B
positive regulation of steroid biosynthetic process (GO:0010893)	0.04	-0.77	1.15	IL1B
chondrocyte development (GO:0002063)	0.04	-0.77	1.15	SULF1
regulation of heterotypic cell-cell adhesion (GO:0034114)	0.04	-0.77	1.15	IL1B
negative regulation of monooxygenase activity (GO:0032769)	0.03	-0.77	1.14	GLA
low-density lipoprotein particle remodeling (GO:0034374)	0.03	-0.77	1.14	PLA2G2A
UTP biosynthetic process (GO:0006228)	0.04	-0.77	1.14	NME5
positive regulation of prostaglandin secretion (GO:0032308)	0.02	-0.76	1.14	IL1B
embryonic heart tube development (GO:0035050)	0.04	-0.76	1.13	MED1
negative regulation of cytosolic calcium ion concentration (GO:0051481)	0.02	-0.76	1.13	GTF2I
monocyte differentiation (GO:0030224)	0.05	-0.76	1.12	MED1
negative regulation of glucose transport (GO:0010829)	0.04	-0.74	1.11	IL1B
ncRNA catabolic process (GO:0034661)	0.04	-0.74	1.10	DIS3L
positive regulation of chemokine biosynthetic process (GO:0045080)	0.03	-0.74	1.10	IL1B
macrophage chemotaxis (GO:0048246)	0.04	-0.73	1.09	SFTPD
UTP metabolic process (GO:0046051)	0.04	-0.73	1.09	NME5
mitochondrion distribution (GO:0048311)	0.03	-0.73	1.08	MSTO1
glycerol ether metabolic process (GO:0006662)	0.04	-0.73	1.08	TXNDC2
positive regulation of interleukin-8 biosynthetic process (GO:0045416)	0.02	-0.72	1.08	PRG3
beta-amyloid metabolic process (GO:0050435)	0.03	-0.72	1.07	NCSTN
positive regulation of protein import into nucleus, translocation (GO:0033160)	0.03	-0.72	1.07	MED1
regulation of G0 to G1 transition (GO:0070316)	0.02	-0.71	1.06	MED1
histone H2A acetylation (GO:0043968)	0.03	-0.71	1.06	BRD8
progesterone metabolic process (GO:0042448)	0.04	-0.71	1.06	AKR1C2
iron-sulfur cluster assembly (GO:0016226)	0.04	-0.70	1.05	HSCB
regulation of nitric-oxide synthase biosynthetic process (GO:0051769)	0.04	-0.69	1.03	FCER2
regulation of interleukin-6 biosynthetic process (GO:0045408)	0.05	-0.69	1.03	IL1B

ventricular trabecula myocardium morphogenesis (GO:0003222)	0.03	-0.69	1.03	MED1
regulation of transcription from RNA polymerase I promoter (GO:0006356)	0.04	-0.69	1.03	MED1
ether metabolic process (GO:0018904)	0.04	-0.69	1.03	TXNDC2
metallo-sulfur cluster assembly (GO:0031163)	0.04	-0.69	1.03	HSCB
mitochondrial ATP synthesis coupled proton transport (GO:0042776)	0.05	-0.68	1.02	ATP5F1
positive regulation of heat generation (GO:0031652)	0.02	-0.68	1.02	IL1B
mRNA transcription (GO:0009299)	0.05	-0.68	1.02	MED1
positive regulation of nitric-oxide synthase biosynthetic process (GO:0051770)	0.03	-0.68	1.01	FCER2
androgen biosynthetic process (GO:0006702)	0.04	-0.67	1.00	MED1
daunorubicin metabolic process (GO:0044597)	0.02	-0.67	1.00	AKR1C2
**GO Cellular component**	**P-value**	**Z-score**	**Combined Score**	**Genes**
lipid particle (GO:0005811)	0.01	-2.41	3.80	LMLN;FAF2
cytoplasmic membrane-bounded vesicle (GO:0016023)	0.01	-2.39	3.77	NCF2;IL1B;SFTPD;PLA2G2A;SLC40A1
secretory granule (GO:0030141)	0.02	-2.26	3.56	NCF2;IL1B;PLA2G2A
SWI/SNF superfamily-type complex (GO:0070603)	0.02	-2.27	3.57	CSNK2A1;BRD8
motile cilium (GO:0031514)	0.02	-2.04	3.21	TXNDC2;NME5
Swr1 complex (GO:0000812)	0.03	-1.16	1.82	BRD8
outer dense fiber (GO:0001520)	0.03	-0.99	1.56	TXNDC2
methylosome (GO:0034709)	0.03	-1.42	2.24	CLNS1A
NADPH oxidase complex (GO:0043020)	0.03	-1.26	1.98	NCF2
endocytic vesicle (GO:0030139)	0.03	-2.06	3.25	NCF2;SFTPD
Sin3 complex (GO:0016580)	0.04	-1.41	2.22	CSNK2A1
Sin3-type complex (GO:0070822)	0.04	-1.32	2.08	CSNK2A1
sperm part (GO:0097223)	0.04	-1.98	3.12	NCF2;NME5
sperm flagellum (GO:0036126)	0.04	-1.25	1.97	NME5
proton-transporting ATP synthase complex, coupling factor F(o) (GO:0045263)	0.04	-1.19	1.87	ATP5F1
lysosome (GO:0005764)	0.04	-2.04	3.20	NCF2;SFTPD;GLA
lytic vacuole (GO:0000323)	0.04	-2.02	3.17	NCF2;SFTPD;GLA
NuA4 histone acetyltransferase complex (GO:0035267)	0.05	-1.14	1.80	BRD8
CCR4-NOT complex (GO:0030014)	0.05	-1.10	1.74	PATL1
eukaryotic 48S preinitiation complex (GO:0033290)	0.05	-1.04	1.63	EIF3K
H4/H2A histone acetyltransferase complex (GO:0043189)	0.05	-1.33	2.09	BRD8
H4 histone acetyltransferase complex (GO:1902562)	0.05	-1.09	1.71	BRD8

eukaryotic 43S preinitiation complex (GO:0016282)	0.05	-0.95	1.49	EIF3K
translation preinitiation complex (GO:0070993)	0.05	-0.86	1.36	EIF3K
**GO Molecular functions**	**P-value**	**Z-score**	**Combined Score**	**Genes**
carbohydrate binding (GO:0030246)	0.01	-2.43	3.45	FCER2;SIGLEC9;SFTPD;PRG3
antioxidant activity (GO:0016209)	0.02	-2.30	3.25	GPX6;TXNDC2
thyroid hormone receptor activity (GO:0004887)	0.02	-2.27	3.22	BRD8
phosphoric diester hydrolase activity (GO:0008081)	0.02	-2.26	3.20	PLCG2;PDE6D
phospholipase activity (GO:0004620)	0.02	-2.25	3.19	PLA2G2A;PLCG2
nucleotide diphosphatase activity (GO:0004551)	0.03	-2.03	2.87	NUDT2
calcium-dependent phospholipase A2 activity (GO:0047498)	0.03	-2.02	2.87	PLA2G2A
peroxisome proliferator activated receptor binding (GO:0042975)	0.03	-2.29	3.25	MED1
oxidoreductase activity, acting on a sulfur group of donors, NAD(P) as acceptor (GO:0016668)	0.03	-2.04	2.89	TXNDC2
oxidoreductase activity, acting on NAD(P)H (GO:0016651)	0.03	-2.24	3.18	AKR1C2;TXNDC2
lipase activity (GO:0016298)	0.03	-2.20	3.12	PLA2G2A;PLCG2
proton-transporting ATP synthase activity, rotational mechanism (GO:0046933)	0.03	-2.14	3.04	ATP5F1
alcohol dehydrogenase (NADP+) activity (GO:0008106)	0.03	-2.06	2.92	AKR1C2
prostaglandin receptor activity (GO:0004955)	0.03	-2.06	2.91	AKR1C2
arylsulfatase activity (GO:0004065)	0.03	-2.04	2.90	SULF1
prostanoid receptor activity (GO:0004954)	0.04	-2.19	3.10	AKR1C2
poly(U) RNA binding (GO:0008266)	0.04	-1.71	2.43	PATL1
GTPase inhibitor activity (GO:0005095)	0.04	-2.02	2.86	PDE6D
poly-purine tract binding (GO:0070717)	0.04	-2.03	2.88	PATL1
vitamin D receptor binding (GO:0042809)	0.04	-1.84	2.60	MED1
poly-pyrimidine tract binding (GO:0008187)	0.04	-1.81	2.56	PATL1
interleukin-1 receptor binding (GO:0005149)	0.05	-1.98	2.81	IL1B
icosanoid receptor activity (GO:0004953)	0.05	-1.92	2.73	AKR1C2
Hsp90 protein binding (GO:0051879)	0.05	-1.99	2.82	CSNK2A1
aldo-keto reductase (NADP) activity (GO:0004033)	0.05	-1.97	2.78	AKR1C2
glutathione peroxidase activity (GO:0004602)	0.05	-1.93	2.73	GPX6
lipase inhibitor activity (GO:0055102)	0.05	-1.81	2.56	FAF2
**KEGG PATHWAYS**	**P-value**	**Z-score**	**Combined Score**	**Genes**

Alzheimer's disease_Homo sapiens_hsa05010	0.00	-1.84	2.60	PSENEN;NCSTN;IL1B;ATP5F1
NF-kappa B signaling pathway_Homo sapiens_hsa04064	0.01	-1.74	2.45	CSNK2A1;IL1B;PLCG2
Basal transcription factors_Homo sapiens_hsa03022	0.01	-1.79	2.52	GTF2A1L;GTF2I
Osteoclast differentiation_Homo sapiens_hsa04380	0.01	-1.79	2.52	NCF2;IL1B;PLCG2
Notch signaling pathway_Homo sapiens_hsa04330	0.02	-1.59	2.24	PSENEN;NCSTN
Measles_Homo sapiens_hsa05162	0.02	-1.73	2.44	CSNK2A1;IL1B;IL13
Arachidonic acid metabolism_Homo sapiens_hsa00590	0.02	-1.76	2.35	GPX6;PLA2G2A
Inflammatory bowel disease (IBD)_Homo sapiens_hsa05321	0.03	-1.70	2.27	IL1B;IL13
Fc epsilon RI signaling pathway_Homo sapiens_hsa04664	0.03	-1.77	2.37	IL13;PLCG2
Purine metabolism_Homo sapiens_hsa00230	0.03	-1.56	2.08	PDE6D;NUDT2;NME5
Leishmaniasis_Homo sapiens_hsa05140	0.03	-1.55	2.06	NCF2;IL1B
Herpes simplex infection_Homo sapiens_hsa05168	0.03	-1.52	2.02	CSNK2A1;IL1B;GTF2I
RNA degradation_Homo sapiens_hsa03018	0.04	-1.49	1.98	DIS3L;PATL1
Epstein-Barr virus infection_Homo sapiens_hsa05169	0.04	-1.63	2.03	FCER2;CSNK2A1;PLCG2
Hematopoietic cell lineage_Homo sapiens_hsa04640	0.05	-1.38	1.71	FCER2;IL1B
**REACTOME PATHWAYS**	**P-value**	**Z-score**	**Combined Score**	**Genes**
Signaling by NOTCH2_Homo sapiens_R-HSA-1980145	0.00	-2.12	7.60	PSENEN;FCER2;NCSTN
A third proteolytic cleavage releases NICD_Homo sapiens_R-HSA-157212	0.00	-2.08	7.46	PSENEN;NCSTN
Regulated proteolysis of p75NTR_Homo sapiens_R-HSA-193692	0.00	-1.60	5.76	PSENEN;NCSTN
Signaling by NOTCH4_Homo sapiens_R-HSA-1980150	0.00	-1.85	6.65	PSENEN;NCSTN
Signaling by NOTCH3_Homo sapiens_R-HSA-1980148	0.00	-1.83	6.59	PSENEN;NCSTN
NRIF signals cell death from the nucleus_Homo sapiens_R-HSA-205043	0.00	-1.75	5.65	PSENEN;NCSTN
NOTCH2 Activation and Transmission of Signal to the Nucleus_Homo sapiens_R-HSA-2979096	0.00	-1.85	5.14	PSENEN;NCSTN
Activated NOTCH1 Transmits Signal to the Nucleus_Homo sapiens_R-HSA-2122948	0.00	-1.98	4.45	PSENEN;NCSTN
Detoxification of Reactive Oxygen Species_Homo sapiens_R-HSA-3299685	0.01	-2.21	4.83	GPX6;NUDT2
Signaling by NOTCH_Homo sapiens_R-HSA-157118	0.01	-1.94	4.03	PSENEN;FCER2;NCSTN
Nuclear signaling by ERBB4_Homo sapiens_R-HSA-1251985	0.01	-1.97	4.11	PSENEN;NCSTN
EPH-ephrin mediated repulsion of cells_Homo sapiens_R-HSA-3928665	0.01	-1.94	3.52	PSENEN;NCSTN

Signaling by NOTCH1 PEST Domain Mutants in Cancer_Homo sapiens_R-HSA-2644602	0.01	-1.82	3.31	PSENEN;NCSTN
Signaling by NOTCH1 in Cancer_Homo sapiens_R-HSA-2644603	0.01	-1.81	3.28	PSENEN;NCSTN
Constitutive Signaling by NOTCH1 PEST Domain Mutants_Homo sapiens_R-HSA-2644606	0.01	-1.80	3.27	PSENEN;NCSTN
Constitutive Signaling by NOTCH1 HD+PEST Domain Mutants_Homo sapiens_R-HSA-2894862	0.01	-1.79	3.25	PSENEN;NCSTN
Signaling by NOTCH1 HD+PEST Domain Mutants in Cancer_Homo sapiens_R-HSA-2894858	0.01	-1.78	3.23	PSENEN;NCSTN
Cell death signalling via NRAGE, NRIF and NADE_Homo sapiens_R-HSA-204998	0.02	-1.68	2.94	PSENEN;NCSTN
Defective ABCA3 causes pulmonary surfactant metabolism dysfunction type 3 (SMDP3)_Homo sapiens_R-HSA-5683678	0.02	0.02	-0.04	SFTPD
CLEC7A/inflammasome pathway_Homo sapiens_R-HSA-5660668	0.02	-0.42	0.70	IL1B
Signaling by NOTCH1_Homo sapiens_R-HSA-1980143	0.02	-1.83	3.04	PSENEN;NCSTN
Interleukin-1 processing_Homo sapiens_R-HSA-448706	0.02	-0.59	0.98	IL1B
APEX1-Independent Resolution of AP Sites via the Single Nucleotide Replacement Pathway_Homo sapiens_R-HSA-5649702	0.02	-0.38	0.63	XRCC1
WNT mediated activation of DVL_Homo sapiens_R-HSA-201688	0.03	-0.50	0.83	CSNK2A1
Defective CSF2RA causes pulmonary surfactant metabolism dysfunction 4 (SMDP4)_Homo sapiens_R-HSA-5688890	0.03	-0.48	0.80	SFTPD
Cross-presentation of particulate exogenous antigens (phagosomes)_Homo sapiens_R-HSA-1236973	0.03	-0.34	0.57	NCF2
Defective CSF2RB causes pulmonary surfactant metabolism dysfunction 5 (SMDP5)_Homo sapiens_R-HSA-5688849	0.03	-0.33	0.55	SFTPD
ABC transporter disorders_Homo sapiens_R-HSA-5619084	0.03	-0.25	0.41	SFTPD
p75 NTR receptor-mediated signalling_Homo sapiens_R-HSA-193704	0.03	-1.58	2.64	PSENEN;NCSTN
SLBP independent Processing of Histone Pre-mRNAs_Homo sapiens_R-HSA-111367	0.03	-0.62	1.01	ZNF473
Dectin-2 family_Homo sapiens_R-HSA-5621480	0.03	-0.59	0.96	PLCG2
Diseases associated with surfactant metabolism_Homo sapiens_R-HSA-5687613	0.03	-0.57	0.93	SFTPD
HDR through MMEJ (alt-NHEJ)_Homo sapiens_R-HSA-5685939	0.03	-0.44	0.72	XRCC1
EPH-Ephrin signaling_Homo sapiens_R-HSA-2682334	0.04	-1.70	2.77	PSENEN;NCSTN

Condensation of Prometaphase Chromosomes_Homo sapiens_R-HSA-2514853	0.04	-0.57	0.93	CSNK2A1
SLBP Dependent Processing of Replication-Dependent Histone Pre-mRNAs_Homo sapiens_R-HSA-77588	0.04	-0.55	0.89	ZNF473
CLEC7A (Dectin-1) signaling_Homo sapiens_R-HSA-5607764	0.04	-1.82	2.92	IL1B;PLCG2
NOTCH2 intracellular domain regulates transcription_Homo sapiens_R-HSA-2197563	0.04	-0.76	1.21	FCER2
RHO GTPases Activate NADPH Oxidases_Homo sapiens_R-HSA-5668599	0.04	-0.73	1.14	NCF2
Synthesis of bile acids and bile salts via 24-hydroxycholesterol_Homo sapiens_R-HSA-193775	0.05	-0.89	1.36	AKR1C2
Synthesis of bile acids and bile salts via 27-hydroxycholesterol_Homo sapiens_R-HSA-193807	0.05	-0.89	1.35	AKR1C2
mRNA decay by 5' to 3' exoribonuclease_Homo sapiens_R-HSA-430039	0.05	-0.79	1.20	PATL1
Disorders of transmembrane transporters_Homo sapiens_R-HSA-5619115	0.05	-0.71	1.09	SFTPD

Supplementary Table-3 : Variable important in Projection Score for the Genes Differentially expressed in GR.A:SAHIO as compared to GR.B:SAHNIO

Multivariate analysis Liver biopsy GR.A:SAHIO vs Gr.B:SAHNIO					
**Genes**	**Component. 1**	**Component. 2**	**Component. 3**	**Component. 4**	**Component. 5**
APOA1	28.59	27.55	25.43	24.14	23.97
APOE	14.92	13.36	12.29	12.07	11.94
HP	12.94	10.21	9.36	9.18	9.56
MTRNR2L1	8.77	7.04	8.26	7.92	8.42
A1BG	8.36	7.11	8.46	8.55	8.45
PPEF2	7.9	11.84	11.34	12.34	12.3
ALB	7.75	6.75	6.27	6.33	6.26
FGA	7.43	10.85	10.08	9.64	9.55
CYP2A6	6.69	6.3	5.77	5.57	5.51
MTRNR2L2	6.32	5.17	5.69	5.41	5.67
ALDOB	5.54	4.63	4.36	4.15	4.1
FABP1	5.46	4.35	4.06	4.66	4.63
AMBP	5.38	4.99	4.6	4.37	4.31
HRG	4.92	3.82	3.74	3.64	3.64
SERPINC1	4.34	5.39	4.95	4.71	4.65
CES1	4.11	4.03	3.81	4.34	4.33
IGFBP4	3.87	3	2.98	2.85	2.82
VTN	3.45	4.93	4.65	4.62	4.77
APOC3	3.2	2.97	2.76	2.62	2.59
HPX	3.05	2.55	2.35	2.35	2.4
APOA2	3.04	2.36	2.33	2.27	2.62
C1R	3.04	3.18	2.92	2.83	2.81
RBP4	2.97	2.77	2.55	2.42	2.4
CYP2D6	2.95	2.41	2.22	2.22	2.19
RNF141	2.94	3.49	3.64	3.61	3.67
C4A	2.92	2.26	2.11	2.18	2.17
FN1	2.9	2.92	2.74	3.28	3.3
AHSG	2.75	2.18	2.1	2	2.01
MAT1A	2.74	3.75	3.44	3.27	3.23
C4B	2.72	2.12	2.02	2.06	2.06
HSPA1B	2.58	4.27	5.54	6.83	6.89
HSPA1A	2.55	3.95	5.07	6.13	6.17
C9orf172	2.55	3.02	3.42	3.28	3.26
OPLAH	2.51	2.83	3.14	3	2.98
COL18A1	2.49	2.01	1.86	1.77	1.75

C8G	2.49	1.92	1.85	1.77	1.75
CFHR1	2.42	2.41	2.31	2.55	2.52
DUX4	2.41	2.85	3.17	3.04	3.02
COL1A1	2.41	2.71	2.71	2.68	2.67
HSPB1	2.35	2.79	3.11	3.7	3.72
HPN	2.34	1.81	1.72	1.66	1.64
LRFN4	2.32	2.55	2.92	2.8	2.79
C1S	2.31	2.43	2.26	2.33	2.38
DUX4L5	2.31	2.73	3.05	2.94	2.93
FASN	2.28	2.32	2.54	2.43	2.41
APOB	2.25	2.45	2.31	2.69	2.68
SDS	2.18	2.38	2.18	2.08	2.06
AGRN	2.17	2.3	2.2	2.09	2.06
SIX5	2.17	2.42	2.66	2.56	2.55
DGKQ	2.16	2.59	2.9	2.78	2.77
APOA5	2.15	1.87	1.71	1.76	1.73
AGT	2.14	1.8	1.66	2.18	2.24
CYP2C9	2.14	3.5	3.21	3.1	3.06
DUX2	2.07	2.42	2.79	2.69	2.67
PCDH10	2.04	3.33	3.08	2.93	2.89
SAA2-SAA4	2.03	2.35	2.2	2.63	2.63
GC	2.02	1.99	1.84	2.43	2.5
SLC35C1	2.01	4.4	4.29	4.3	4.31
CYB5A	1.95	1.53	1.6	1.56	1.54
METTL7B	1.95	1.53	1.45	1.54	1.52
ABCA2	1.95	2.24	2.5	2.39	2.37
DUX4L7	1.92	2.3	2.62	2.53	2.52
ADH1C	1.92	1.66	1.61	1.67	1.67
CYP2A7	1.91	2.6	2.4	2.28	2.25
ERBB2	1.91	2	1.99	2	1.97
UGT2B10	1.9	1.89	1.88	1.93	1.93
PLXNB3	1.89	2.27	2.56	2.45	2.43
KLHL34	1.89	1.77	2.39	2.39	2.37
APCS	1.89	1.54	1.41	2.06	2.13
EPS8L2	1.89	1.68	1.82	1.73	1.72
CDT1	1.89	2.19	2.49	2.4	2.39
A2M	1.87	1.71	1.62	1.84	1.86
CFB	1.87	1.63	1.53	1.93	2.1
NNMT	1.85	1.8	1.65	1.61	1.62
HSPA1L	1.84	2.92	3.79	4.68	4.72
SDC1	1.84	1.43	1.39	1.32	1.32

CPSF1	1.82	2.11	2.37	2.27	2.25
TMED7	1.82	1.67	1.62	1.61	1.6
ASGR2	1.81	1.4	1.29	1.41	1.44
DUX4L4	1.81	2.13	2.44	2.34	2.33
MAGED1	1.8	1.41	1.31	1.3	1.28
SAA4	1.79	2.06	1.95	2.26	2.26
SERPINF2	1.77	1.72	1.59	1.51	1.49
BAIAP3	1.76	2.07	2.32	2.23	2.21
FBXW5	1.74	1.87	2.04	1.97	1.96
RASSF7	1.72	1.73	1.91	1.82	1.82
TAT	1.72	2.58	2.42	2.35	2.33
COL4A2	1.72	1.7	1.68	1.67	1.67
CD81	1.71	1.74	1.62	1.56	1.55
SLC22A18	1.7	1.32	1.25	1.22	1.21
ADCK5	1.66	1.91	2.23	2.13	2.12
LIME1	1.66	1.35	1.61	1.69	1.68
SLC16A8	1.64	1.3	1.49	1.42	1.42
SERPIND1	1.63	1.33	1.35	1.31	1.3
ACAP3	1.62	1.86	2.1	2.03	2.02
ELFN1	1.62	1.63	1.9	1.82	1.81
ADAM17	1.61	1.71	1.7	1.72	1.71
SERPINA5	1.6	1.58	1.45	1.38	1.36
IRF2BP1	1.6	1.46	1.48	1.48	1.47
SLC26A1	1.59	1.72	1.99	1.92	1.9
TAGLN	1.58	1.45	1.62	1.59	1.59
C4BPA	1.58	1.48	1.4	1.58	1.57
CFH	1.58	1.43	1.32	1.78	1.86
VASN	1.57	1.5	1.53	1.46	1.44
AADAC	1.55	1.77	1.68	1.9	1.91
LRRC3	1.54	1.24	1.38	1.31	1.3
CP	1.54	1.63	1.61	1.67	1.65
UGT2B4	1.53	1.58	1.45	2.07	2.13
GJB1	1.53	2.3	2.14	2.65	2.64
AGXT	1.52	1.61	1.47	1.4	1.38
CDKN1C	1.52	1.54	1.66	1.59	1.58
MEGF6	1.52	1.75	1.94	1.92	1.91
C7	1.52	1.62	1.49	1.43	1.41
EMILIN1	1.52	1.42	1.43	1.4	1.38
HELZ2	1.52	1.73	1.93	1.86	1.85
NOC4L	1.5	1.46	1.75	1.67	1.66
ANGPTL4	1.49	1.23	1.15	1.28	1.27

AHDC1	1.49	1.46	1.77	1.71	1.7
C19orf26	1.48	1.71	2	1.92	1.91
POU3F1	1.48	1.59	1.76	1.75	1.75
SAA1	1.47	1.28	1.18	1.65	1.69
INO80E	1.45	1.42	1.3	1.25	1.24
ZBTB16	1.44	1.37	1.25	1.22	1.21
UGT2B7	1.44	1.35	1.25	1.6	1.68
CD163	1.43	1.42	1.42	1.45	1.43
POR	1.43	1.42	1.34	1.3	1.29
CLDN3	1.43	1.76	1.92	1.94	1.92
MST1	1.43	1.63	1.57	1.51	1.49
HELT	1.42	1.28	1.48	1.41	1.4
LAMA5	1.42	1.59	1.92	1.83	1.81
TF	1.41	2.83	2.86	2.73	2.73
ID1	1.41	1.19	1.2	1.22	1.21
SLC9A3R2	1.41	1.16	1.17	1.33	1.31
PTGR1	1.41	1.45	1.34	1.74	1.74
C11orf35	1.4	1.11	1.76	1.68	1.66
MFSD10	1.4	1.69	1.97	1.9	1.88
AMDHD2	1.4	1.4	1.59	1.52	1.51
C2	1.4	1.1	1.01	1.15	1.18
DVL1	1.39	1.5	1.73	1.66	1.65
RDH16	1.39	1.29	1.25	1.26	1.25
PLEC	1.38	1.17	1.08	1.13	1.11
ITPKA	1.38	1.58	1.83	1.78	1.76
PIDD	1.37	1.4	1.72	1.66	1.65
SPATC1	1.35	1.05	1.18	1.2	1.18
CBR1	1.35	1.05	0.96	1.21	1.25
GCGR	1.35	1.37	1.56	1.51	1.5
TSPAN4	1.34	1.46	1.66	1.61	1.6
C11orf96	1.33	1.44	1.34	1.43	1.41
TM4SF4	1.32	1.21	1.12	1.23	1.32
MAF	1.32	1.03	0.94	0.9	0.89
ISYNA1	1.32	1.5	1.78	1.75	1.74
C9orf173	1.31	1.03	1.49	1.41	1.4
NFKBIL1	1.31	1.3	1.21	1.2	1.19
VWA1	1.31	1.33	1.35	1.37	1.36
UGT1A1	1.29	1.45	1.39	1.53	1.52
MMP14	1.29	1	0.95	0.96	0.97
C21orf33	1.29	1.01	0.93	0.88	0.88
GCDH	1.29	1.34	1.24	1.19	1.18

TACSTD2	1.29	1.23	1.16	1.22	1.2
TST	1.28	1.59	1.5	1.49	1.47
HIC1	1.28	1.49	1.73	1.67	1.66
KNOP1	1.28	1.26	1.16	1.11	1.1
RHOT2	1.27	1.43	1.65	1.6	1.58
C1orf122	1.27	1.32	1.24	1.23	1.22
HEXDC	1.27	0.98	1.04	0.99	0.99
ZBED1	1.26	1.03	0.96	0.92	0.91
PLXNB2	1.25	1.24	1.33	1.26	1.25
CTU2	1.25	1.39	1.51	1.44	1.44
IRF2BPL	1.24	1.33	1.54	1.49	1.47
TPST2	1.24	1.18	1.14	1.13	1.12
HNF4A	1.24	1.15	1.07	1.02	1.01
CDKN1A	1.23	1.31	1.3	1.26	1.26
GLYCTK	1.23	1.13	1.08	1.08	1.06
KRT19	1.23	1.01	0.96	0.91	0.9
PRKCDBP	1.23	1.23	1.15	1.09	1.08
SLC25A22	1.23	1.25	1.43	1.36	1.35
FOXL2	1.22	1.13	1.94	1.85	1.83
DPT	1.22	1.39	1.27	1.21	1.19
GATM	1.21	1.13	1.06	1.02	1.01
C16orf13	1.2	1.32	1.74	1.73	1.71
MXRA8	1.2	1.11	1.47	1.42	1.4
TSKU	1.2	1.42	1.33	1.32	1.31
IDUA	1.2	1.41	1.58	1.51	1.5
FAHD1	1.19	0.93	0.9	0.91	0.91
ARFRP1	1.19	1.41	1.48	1.41	1.39
TBL3	1.19	1.37	1.62	1.55	1.54
FAM83H	1.18	1.11	1.12	1.06	1.05
RAB43	1.17	0.93	0.85	0.86	0.85
NUDC	1.17	0.91	0.83	0.92	0.92
APOC1	1.16	1.21	1.13	1.11	1.1
STARD10	1.16	1.17	1.18	1.13	1.15
CYGB	1.16	0.92	0.86	0.82	0.82
NFYB	1.16	1.01	1.18	1.16	1.23
HRAS	1.15	1.28	1.72	1.64	1.62
CAPN15	1.15	1.32	1.48	1.43	1.42
IQCJ-SCHIP1	1.14	0.93	0.89	0.86	0.85
PRDX4	1.14	1.3	1.48	1.49	1.47
ITGB5	1.13	0.88	0.82	0.85	0.84
SLC25A1	1.12	0.88	0.83	0.79	0.78

MFSD3	1.12	1.16	1.41	1.34	1.33
ANKDD1A	1.12	1.31	1.41	1.35	1.39
ACADVL	1.12	1.32	1.22	1.21	1.22
UGT1A10	1.12	1.24	1.18	1.32	1.31
PEBP1	1.12	1.31	1.2	1.15	1.14
ACO2	1.12	0.97	0.92	1.01	1
DPP7	1.12	1.21	1.39	1.36	1.35
DOLK	1.12	1.1	1.13	1.07	1.07
PCSK1N	1.11	1.06	0.98	0.96	0.96
CAPS	1.11	0.97	1.56	1.57	1.56
RPN1	1.11	0.86	0.79	0.85	0.85
HYOU1	1.11	0.86	0.79	0.9	0.89
LGALS4	1.1	0.92	0.96	0.92	0.91
TMEM86B	1.1	0.98	1.02	0.97	0.96
ZGPAT	1.1	0.87	1.25	1.23	1.21
CTGF	1.1	1.08	0.99	0.97	0.96
ISY1-RAB43	1.1	0.85	0.8	0.8	0.79
TRIL	1.1	1.2	1.76	1.72	1.7
MAGEF1	1.09	0.94	0.86	0.82	0.81
MACROD1	1.09	1.25	1.54	1.5	1.48
CYP2C8	1.09	1.67	1.54	1.46	1.44
RGS11	1.09	0.85	0.99	0.98	0.97
HDHD3	1.08	1.26	1.16	1.1	1.09
OSGIN1	1.08	0.91	0.84	0.81	0.8
ODF3B	1.08	1.29	1.63	1.58	1.56
CDK5R2	1.08	1.34	1.57	1.51	1.5
PVRIG	1.08	1.03	1.07	1.01	1.01
IL17RC	1.08	1.09	1.03	0.98	0.97
APOA4	1.07	1.24	1.15	1.41	1.41
CYC1	1.07	1.23	1.14	1.09	1.07
BST2	1.07	1.23	1.13	1.08	1.07
ST13	1.07	0.91	0.97	0.92	0.91
PCK2	1.07	1.09	1	0.94	0.93
C6	1.07	0.95	0.87	0.86	0.86
BLVRB	1.07	1.2	1.14	1.12	1.1
SLC25A47	1.06	1.16	1.07	1.03	1.02
TMEM259	1.06	1.11	1.27	1.25	1.24
FOXD3	1.06	1.23	1.66	1.65	1.64
KRTAP10-9	1.06	1.11	1.65	1.59	1.57
CDR1	1.05	1.43	1.51	1.44	1.45
FAM213B	1.05	1.11	1.44	1.38	1.37

EFHD1	1.05	0.84	0.77	0.75	0.74
ADAMTSL2	1.05	0.82	0.75	0.77	0.76
PPIB	1.04	0.92	0.84	0.82	0.81
PNPLA3	1.04	0.96	0.88	0.84	0.83
DAP	1.04	0.91	0.85	0.81	0.8
C7orf49	1.04	0.95	0.87	0.83	0.83
PCDHGB5	1.04	0.94	0.92	0.9	0.89
BRF1	1.03	1.04	1.18	1.12	1.12
RECQL4	1.03	1.12	1.48	1.41	1.39
VNN1	1.03	1.16	1.1	1.26	1.27
EGFL7	1.03	1.04	1.41	1.36	1.34
ARHGEF16	1.03	1.14	1.27	1.21	1.21
PROS1	1.03	1.04	1.01	1.05	1.04
TRAPPC12	1.02	0.84	0.77	0.73	0.73
BOK	1.02	0.99	1.09	1.05	1.05
PCBD1	1.02	1.38	1.53	1.48	1.46
C11orf95	1.02	0.94	0.93	0.9	0.89
NAPRT1	1.02	1.11	1.19	1.15	1.15
RTEL1	1.02	1.12	1.46	1.42	1.41
GPX1	1.01	0.92	0.9	0.85	0.84
LRP1	1.01	0.78	0.74	0.75	0.75
NUMA1	1.01	0.79	0.77	0.75	0.74
MYL5	1.01	0.9	1.04	1	0.99
PTK6	1.01	1.12	1.4	1.34	1.33
SP9	1.01	1.23	1.65	1.57	1.56
ZNF622	1.01	0.84	0.8	0.78	0.78
MYH11	1	0.84	0.8	0.85	0.89
SEPHS2	1	0.93	1.04	1.02	1.01

Supplementary table 4 : Expression changes in the genes associated to inflamation, oxidative stress and iron processing in GR.A:SAHIO as compared to GR.B:SAHNIO

Modules	Sr.no	Liver biopsy					PBMC			
		Name	Mean (SD) of GR.B:SAHNIO	Mean (SD) of GR.A:SAHIO	p-value	FC (log) GR.A:SAHIO/GR.B:SAHNIO	Mean (SD) of GR.B:SAHNIO	Mean (SD) of GR.A:SAHIO	p-value	FC (log)GR.A:SAHIO/GR.B:SAHNIO
Chemokine Genes:	1	C5	-0.073 (1.591)	0.121 (1.301)	0.56	0.19	0.116 (1.089)	-0.209 (1.163)	0.61	-0.33
	2	CCL1	0.048 (0.863)	-0.080 (0.406)	0.74	-0.13	0.043 (0.707)	-0.077 (0.469)	0.74	-0.12
	3	CCL11	0.117 (1.204)	-0.195 (1.201)	0.43	-0.31	0.195 (0.998)	-0.351 (1.481)	0.30	-0.55
	4	CCL13	-0.018 (0.860)	0.030 (0.892)	0.87	0.05	0.795 (1.118)	-0.921 (0.735)	0.01	-5.56
	5	CCL15	0.525 (2.579)	-0.875 (0.549)	0.56	-1.40	-0.194 (0.925)	0.861 (0.739)	0.05	2.87
	6	CCL16	0.532 (2.906)	-0.886 (0.646)	0.79	-1.42	-0.261 (0.691)	0.470 (0.668)	0.08	0.73
	7	CCL17	0.171 (1.528)	-0.284 (0.085)	1.00	-0.46	0.280 (1.496)	-0.505 (1.298)	0.35	-0.79
	8	CCL18	-0.089 (0.754)	0.149 (0.577)	0.52	0.24	-0.044 (0.861)	0.080 (0.803)	0.90	0.12
	9	CCL19	0.176 (1.103)	-0.294 (0.881)	0.39	-0.47	-0.044 (0.819)	0.079 (0.491)	0.77	0.12
	10	CCL2	-0.044 (1.021)	0.073 (1.360)	0.87	0.12	-0.007 (1.109)	0.013 (0.608)	0.97	0.02
	11	CCL20	-0.75 (0.174)	1.78 (3.271)	0.00	2.49	0.365 (1.176)	-0.658 (1.665)	0.19	-1.02
	12	CCL21	-0.027 (1.694)	0.045 (0.829)	0.56	0.07	-0.009 (1.297)	0.015 (1.295)	0.97	0.02
	13	CCL23	0.523 (3.188)	-0.872 (0.224)	0.15	-1.40	-0.158 (1.173)	0.284 (0.369)	0.32	0.44
	14	CCL24	0.128 (0.819)	-0.213 (0.338)	0.35	-0.34	-0.279 (0.736)	1.0143 (1.002)	0.02	3.65
	15	CCL25	0.209 (1.546)	-0.348 (0.555)	0.71	-0.56	-0.136 (0.948)	0.245 (1.134)	0.51	0.38
	16	CCL26	0.210 (0.852)	-0.351 (0.790)	0.22	-0.56	-0.375 (0.523)	0.675 (0.902)	0.02	1.05
	17	CCL3	-0.302 (0.252)	0.503 (2.275)	0.96	0.81	0.008 (1.382)	-0.014 (1.032)	1.00	-0.02
	18	CCL4	0.154 (1.218)	-0.257 (0.446)	0.56	-0.41	0.324 (1.071)	-0.584 (1.155)	0.16	-0.91
	19	CCL5	0.167 (3.001)	-0.279 (1.978)	0.56	-0.45	-0.005 (1.546)	0.010 (1.518)	0.99	0.02
	20	CCL7	-0.118 (1.999)	0.196 (1.706)	0.49	0.31	0.089 (1.055)	-0.160 (1.580)	0.73	-0.25
	21	CCL8	0.113 (0.777)	-0.189 (0.776)	0.31	-0.30	0.073 (0.977)	-0.131 (1.400)	0.75	-0.20
	22	CXCL1	-0.637 (0.444)	1.147 (2.712)	0.02	1.78	0.139 (0.818)	-0.250 (1.298)	0.50	-0.39
	23	CXCL10	0.355 (3.234)	-0.591 (1.093)	0.96	-0.95	0.246 (1.227)	-0.442 (0.670)	0.27	-0.69
	24	CXCL11	0.187 (0.812)	-0.312 (0.203)	0.09	-0.50	0.711 (0.918)	-0.769 (0.845)	0.01	-4.35
	25	CXCL12	0.013 (2.553)	-0.021 (1.639)	0.71	-0.03	0.020 (1.070)	-0.035 (0.668)	0.92	-0.05
	26	CXCL13	-0.153 (0.781)	0.255 (0.818)	0.34	0.41	0.005 (0.750)	-0.009 (1.484)	0.98	-0.01
	27	CXCL14	-0.097 (0.544)	0.161 (1.240)	1.00	0.26	-0.008 (0.832)	0.014 (0.840)	1.00	0.02
	28	CXCL2	-0.587 (2.504)	0.978 (2.116)	0.02	1.57	-0.295 (0.805)	1.042 (0.892)	0.01	3.81
	29	CXCL3	-0.073 (2.298)	0.122 (2.791)	0.96	0.20	0.207 (1.022)	-0.372 (1.570)	0.52	-0.58
	30	CXCL5	0.391 (3.364)	-0.651 (0.204)	0.96	-1.04	0.297 (1.183)	-0.534 (0.943)	0.20	-0.83
	31	CXCL6	0.506 (3.492)	-0.843 (0.928)	1.00	-1.35	-0.058 (0.959)	0.105 (1.479)	0.80	0.16

	32	CXCL9	-0.422 (1.168)	0.703 (2.384)	0.22	1.13	0.019 (1.191)	-0.034 (0.556)	0.80	-0.05
	33	IL13	0.51 (2.135)	-0.336(0.132)	0.02	-0.90	0.110 (1.216)	-0.198 (1.435)	0.68	-0.31
	34	IL8	0.249 (2.730)	-0.415 (1.079)	0.87	-0.66	-0.149 (1.077)	0.268 (1.542)	0.56	0.42
**Chemokine Receptors:**	35	CCR1	-0.101 (0.616)	0.168 (0.562)	0.40	0.27	-0.012 (1.453)	0.022 (1.152)	0.97	0.03
	36	CCR2	-0.181 (0.341)	0.301 (1.429)	1.00	0.48	0.293 (1.244)	-0.528 (0.879)	0.22	-0.82
	37	CCR3	0.118 (1.068)	-0.197 (1.079)	0.43	-0.32	0.134 (1.032)	-0.242 (1.852)	1.00	-0.38
	38	CCR4	-0.010 (0.657)	0.017 (0.981)	0.95	0.03	-0.102 (0.917)	0.184 (0.627)	0.70	0.29
	39	CCR5	0.129 (0.901)	-0.215 (0.572)	0.42	-0.34	0.183 (1.007)	-0.330 (0.502)	0.31	-0.51
	40	CCR6	-0.141 (0.733)	0.235 (0.912)	0.49	0.38	0.353 (0.819)	-0.636 (1.402)	0.12	-0.99
	41	CCR7	-0.013 (1.798)	0.021 (1.711)	0.96	0.03	0.025 (1.222)	-0.046 (1.032)	0.91	-0.07
	42	CCR8	-0.229 (1.715)	0.382 (2.790)	0.79	0.61	-0.041 (1.105)	0.073 (1.625)	0.88	0.11
	43	CCR9	0.470 (2.522)	-0.783 (0.471)	0.49	-1.25	-0.123 (1.014)	0.221 (0.777)	0.52	0.34
	44	CX3CR1	0.281 (1.955)	-0.468 (0.367)	0.87	-0.75	0.019 (1.360)	-0.033 (1.103)	0.94	-0.05
	45	CXCR1	0.134 (2.120)	-0.223 (1.514)	0.71	-0.36	0.026 (1.244)	-0.048 (1.336)	0.92	-0.07
	46	XCR1	-0.315 (0.701)	0.525 (1.838)	0.43	0.84	0.697(0.988)	-0.743(0.726)	0.01	-4.17
	47	CD40LG	0.143 (0.869)	-0.238 (0.599)	0.56	-0.38	0.027 (1.293)	-0.049 (0.522)	0.90	-0.08
**Cytokine Genes:**	48	IFNA2	-0.007 (1.219)	0.011 (0.632)	0.31	0.02	0.284 (1.199)	-0.511 (1.043)	0.24	-0.80
	49	IL10	0.158 (0.904)	-0.263 (0.468)	0.31	-0.42	0.172 (0.936)	-0.309 (1.642)	0.49	-0.48
	50	IL17C	-0.199 (1.701)	0.331 (1.860)	0.15	0.53	0.135 (1.311)	-0.243 (1.420)	1.00	-0.38
	51	IL1A	-0.104 (0.828)	0.174 (0.644)	0.50	0.28	0.009 (1.045)	-0.017 (0.629)	0.96	-0.03
	52	IL1B	0.450 (1.238)	-0.222(0.257)	0.03	-0.71	0.108 (1.641)	-0.195 (0.452)	0.62	-0.30
	53	IL1F10	-0.114 (0.763)	0.190 (1.303)	0.79	0.30	-0.292 (1.008)	0.526 (1.728)	0.28	0.82
	54	IL36RN	0.340 (1.051)	-0.566 (0.514)	0.18	-0.91	0.201 (1.564)	-0.362 (0.308)	0.32	-0.56
	55	IL36A	0.010 (1.068)	-0.016 (0.894)	0.96	-0.03	0.127 (1.323)	-0.228 (0.457)	0.58	-0.36
	56	IL37	-0.324 (0.469)	0.541 (1.835)	0.71	0.87	0.129 (1.561)	-0.232 (0.808)	0.64	-0.36
	57	IL36B	-0.053 (1.066)	0.089 (1.172)	1.00	0.14	0.368 (1.042)	-0.663 (1.520)	0.24	-1.03
	58	IL36G	-0.064 (1.142)	0.107 (0.874)	0.71	0.17	0.277 (1.229)	-0.498 (1.470)	0.44	-0.78
	59	IL22	0.197 (1.059)	-0.328 (0.526)	0.31	-0.53	0.046 (0.969)	-0.082 (1.154)	0.83	-0.13
	60	IL5	0.067 (0.856)	-0.112 (0.504)	0.96	-0.18	0.435 (1.174)	-0.783 (1.450)	0.11	-1.22
	61	IL9	-0.128 (0.721)	0.213 (1.085)	0.46	0.34	-0.015 (1.075)	0.027 (0.660)	0.94	0.04
	62	LTA	0.129 (2.044)	-0.216 (0.805)	1.00	-0.35	0.080 (1.200)	-0.143 (1.482)	0.76	-0.22
	63	LTB	0.015 (2.328)	-0.026 (1.259)	0.43	-0.04	-0.142 (1.450)	0.255 (0.769)	0.58	0.40
	64	MIF	-1.272 (3.372)	2.289 (4.781)	0.01	3.56	0.588(1.295)	-0.547(0.262)	0.03	-3.13
	65	AIMP1	0.277 (1.811)	-0.462 (0.628)	0.49	-0.74	0.371 (1.130)	-0.667 (0.777)	0.09	-1.04
	66	SPP1	0.103 (2.229)	-0.172 (2.485)	0.82	-0.28	0.128 (1.379)	-0.231 (0.544)	0.70	-0.36
	67	TNF	0.121 (0.719)	-0.201 (0.572)	0.37	-0.32	0.132 (1.170)	-0.238 (1.380)	0.60	-0.37
**Cytokine Receptors:**	68	IL10RA	0.152 (0.847)	-0.253 (0.274)	0.43	-0.41	0.091 (1.439)	-0.164 (0.765)	0.72	-0.26
	69	IL10RB	0.161 (1.847)	-0.268 (0.938)	0.96	-0.43	-0.022 (1.359)	0.040 (0.803)	0.93	0.06
	70	IL13RA1	0.193 (2.630)	-0.322 (1.691)	0.96	-0.52	0.405 (1.409)	-0.728 (0.949)	0.14	-1.13

	71	IL5RA	0.054 (0.844)	-0.090 (0.411)	0.71	-0.14	-0.102 (0.989)	0.184 (0.832)	0.60	0.29
	72	IL9R	0.031 (1.242)	-0.051 (1.750)	0.15	-0.08	0.395 (1.233)	-0.711 (0.427)	0.08	-1.11
**Other Genes Involved in Inflammatory Response:**	73	ABCF1	0.059 (1.912)	-0.098 (1.596)	0.79	-0.16	0.114 (1.382)	-0.206 (1.138)	0.67	-0.32
	74	BCL6	-0.040 (1.254)	0.067 (1.399)	1.00	0.11	-0.008 (1.522)	0.014 (0.990)	0.98	0.02
	75	C3	-0.421 (1.583)	0.701 (2.742)	0.31	1.12	-0.123 (1.366)	0.221 (0.922)	0.52	0.34
	76	C4A	-0.123 (2.045)	0.205 (2.076)	0.64	0.33	0.080 (1.577)	-0.143 (1.059)	0.78	-0.22
	77	CEBPB	0.259 (3.766)	-0.432 (1.525)	0.49	-0.69	-0.091 (1.439)	0.163 (1.438)	0.76	0.25
	78	CRP	-0.015 (1.998)	0.026 (1.858)	0.97	0.04	-0.435 (1.120)	0.782 (0.957)	0.06	1.22
	79	CARD18	0.169 (1.471)	-0.281 (0.228)	1.00	-0.45	-0.009 (1.021)	0.016 (0.777)	0.96	0.03
	80	IL1R1	0.086 (0.929)	-0.144 (0.527)	0.87	-0.23	-0.049 (0.825)	0.088 (0.945)	0.80	0.14
	81	IL1RN	0.212 (1.628)	-0.354 (0.229)	0.64	-0.57	0.141 (1.241)	-0.253 (1.131)	0.57	-0.39
	82	CXCR2	0.400 (2.695)	-0.666 (0.581)	0.71	-1.07	-0.038 (1.566)	0.068 (0.852)	0.89	0.11
	83	LTB4R	-0.407 (1.231)	0.678 (2.592)	0.31	1.09	-0.054 (1.121)	0.096 (1.199)	0.82	0.15
	84	TOLLIP	-0.144 (1.686)	0.241 (1.937)	0.43	0.39	-0.085 (1.288)	0.152 (1.104)	0.74	0.24
**Iron Associated Genes**	85	CP	-2.033 (1.535)	3.660 (1.173)	0.00	5.69	-0.054 (0.834)	0.097 (0.436)	1.00	0.15
	86	CYBRD1	0.218 (0.830)	-0.392 (0.505)	0.16	-0.61	-0.320 (0.465)	0.577 (0.743)	0.02	0.90
	87	FTH	-0.345 (1.267)	0.620 (1.012)	0.17	0.97	-0.411 (1.260)	0.741 (0.618)	0.36	1.15
	88	HAMP	-0.61 (0.215)	1.66 (1.239)	0.00	2.24	-0.259(0.651)	0.977(0.352)	0.01	3.45
	89	HFE	-0.521 (1.357)	0.938 (0.709)	0.04	1.46	0.137 (0.944)	-0.247 (0.440)	0.41	-0.38
	90	SLC11A2	0.111 (0.882)	-0.200 (0.878)	0.54	-0.31	0.111 (0.769)	-0.201 (0.496)	0.43	-0.31
	91	SLC40A1	0.884 (1.630)	-1.592 (0.123)	0.00	-2.48	-0.158 (0.942)	0.285 (0.385)	0.34	0.44
	92	TF	0.327 (1.670)	-0.588 (1.387)	0.32	-0.92	-0.110 (1.110)	0.197 (0.576)	0.58	0.31
	93	TFR2	-0.204 (1.096)	0.367 (1.398)	0.41	0.57	0.130 (1.016)	-0.234 (0.386)	0.46	-0.36
	94	TFRC	-0.557 (1.788)	1.003 (0.450)	0.05	1.56	0.334 (0.730)	-0.601 (0.499)	0.03	-0.94
**Metalloprotease associated genes**	95	EGFR	0.078 (0.743)	-0.141 (0.931)	0.64	-0.22	-0.161 (0.627)	0.802 (0.726)	0.02	2.62
	96	ERBB2	-2.523 (0.091)	4.541 (0.927)	0.00	7.06	-0.415(0.486)	1.258(0.818)	0.00	5.33
	97	ERBB4	-0.50 (0.784)	1.46 (0.394)	0.00	1.93	0.143 (0.821)	-0.257 (0.706)	0.38	-0.40
	98	NCSTN	0.533 (0.738)	-0.407(0.659)	0.03	-0.98	-0.053 (0.561)	0.095 (0.790)	0.69	0.15
	99	NOTCH1	-0.562 (0.720)	1.563(1.057)	0.00	2.09	0.032 (1.050)	-0.058 (0.530)	0.86	-0.09
	100	NOTCH3	-0.891 (0.325)	1.603 (1.046)	0.00	2.49	-0.349(0.610)	1.140(0.402)	0.00	4.44
	101	PACS2	-1.115 (0.158)	2.006 (4.348)	0.00	3.12	0.170 (0.920)	-0.306 (0.745)	0.34	-0.48
	102	PRKCA	0.244 (1.232)	-0.439 (0.406)	0.61	-0.68	0.471 (0.780)	-0.847 (0.440)	0.00	-1.32
	103	PSEN2	-0.532 (0.332)	0.958 (1.714)	0.01	1.49	0.065 (0.788)	-0.116 (1.121)	0.73	-0.18
	104	PSENEN	0.583 (0.595)	-0.497(0.266)	0.00	-1.12	-0.110 (1.178)	0.198 (0.238)	0.47	0.31
	105	TIMP3	-0.042 (1.274)	0.076 (0.740)	0.80	0.12	-0.179 (1.233)	0.323 (0.358)	1.00	0.50
	106	TNFRSF1B	-0.780 (0.498)	1.404 (1.322)	0.00	2.18	0.416 (1.399)	-0.748 (0.680)	0.11	-1.16
**Glutathione Peroxidases(GPx)**	108	GPX1	-1.343 (0.343)	2.418 (2.413)	0.00	3.76	0.054 (1.551)	-0.096 (0.706)	0.80	-0.15
	109	GPX2	0.331 (1.204)	-0.596 (0.954)	0.17	-0.93	-0.218 (1.481)	0.393 (0.413)	0.27	0.61
	110	GPX3	-0.099 (1.439)	0.178 (1.105)	0.72	0.28	-0.208 (0.906)	0.374 (0.419)	0.21	0.58

	111	GPX4	-0.184 (1.612)	0.331 (1.401)	0.56	0.52	0.137 (1.388)	-0.247 (0.590)	0.57	-0.38
	112	GPX5	0.173 (0.752)	-0.312 (0.854)	0.19	-0.49	-0.220 (1.200)	0.397 (0.698)	0.32	0.62
	113	GPX6	0.382 (0.401)	-0.687 (0.790)	0.03	-1.07	-0.048 (1.406)	0.086 (0.526)	0.61	0.13
	114	GPX7	0.011 (1.203)	-0.020 (0.868)	0.96	-0.03	-0.083 (0.825)	0.149 (0.716)	0.61	0.23
	115	GSTP1	0.243 (0.713)	-0.437 (1.150)	0.19	-0.68	0.072 (0.582)	-0.130 (0.809)	0.60	-0.20
	116	GSTZ1	-0.721 (0.639)	1.297 (1.515)	0.02	2.02	0.037 (1.193)	-0.067 (0.655)	0.44	-0.10
	117	GSS	-0.022 (1.364)	0.039 (0.758)	0.93	0.06	0.104 (0.985)	-0.186 (0.758)	0.58	-0.29
**Mediation of oxidative stress**	118	NOX1	0.018 (0.835)	-0.032 (1.324)	0.70	-0.05	-0.470 (1.287)	0.847 (0.704)	0.06	1.32
	119	NOX4	0.104 (1.153)	-0.187 (0.851)	0.63	-0.29	-0.372 (1.102)	0.670 (0.433)	0.07	1.04
	120	NOXA1	0.285 (0.897)	-0.512 (1.194)	0.30	-0.80	-0.322 (1.287)	0.580 (1.044)	0.21	0.90
	121	NOXO1	-0.708 (0.538)	1.274 (2.050)	0.01	1.98	-0.287 (1.090)	0.517 (1.299)	0.44	0.80
**Other Antioxidants**	122	ALB	-10.261 (32.248)	18.469 (36.673)	0.02	28.73	-0.210 (1.262)	0.379 (0.227)	0.21	0.59
	123	APOE	-19.759 (2.153)	35.566 (39.034)	0.00	55.33	0.349 (1.225)	-0.629 (0.572)	0.12	-0.98
	124	GSR	-1.029 (0.546)	1.852 (1.302)	0.00	2.88	-0.080 (1.127)	0.144 (0.923)	0.71	0.22
	125	MT3	0.596 (0.873)	-1.073 (1.392)	0.11	-1.67	-0.260 (1.102)	0.469 (0.860)	0.24	0.73
	126	TXNDC2	0.571 (0.955)	-0.476(0.713)	0.05	-1.09	-0.048 (1.239)	0.086 (0.608)	0.83	0.13
	127	TXNRD2	-1.093 (0.839)	1.968 (3.846)	0.01	3.06	-0.282 (1.125)	0.508 (1.083)	0.44	0.79
	128	VIMP	0.146 (1.239)	-0.263 (0.926)	0.53	-0.41	-0.030 (1.075)	0.053 (0.560)	0.52	0.08
**Other Genes Involved in ROS Metabolism**	129	AOX1	0.107 (1.255)	-0.193 (1.286)	0.70	-0.30	-0.107 (1.187)	0.192 (0.421)	0.60	0.30
	130	BNIP3	-0.088 (0.606)	0.158 (1.157)	0.61	0.25	-0.207 (0.592)	0.372 (1.075)	0.21	0.58
	131	EPHX2	-0.158 (1.114)	0.284 (1.471)	0.54	0.44	-0.076 (1.009)	0.138 (0.689)	0.68	0.21
	132	MPV17	-0.159 (0.852)	0.286 (0.990)	0.39	0.45	-0.088 (0.879)	0.159 (0.719)	0.60	0.25
	133	SFTPD	0.748 (0.763)	-0.795(0.918)	0.00	-1.59	-0.126 (1.327)	0.226 (0.441)	0.48	0.35
**Other Genes Involved in Superoxide Metabolism**	134	ALOX12	0.271 (1.285)	-0.487 (0.560)	0.24	-0.76	0.094 (1.196)	-0.170 (0.730)	0.66	-0.26
	135	CCS	0.344 (1.152)	-0.619 (0.919)	0.14	-0.96	-0.157 (1.118)	0.282 (1.086)	0.49	0.44
	136	CYBA	0.102 (1.620)	-0.183 (0.917)	0.73	-0.29	-0.069 (1.246)	0.125 (1.070)	0.78	0.19
	137	DUOX1	0.261 (0.475)	-0.470 (1.538)	0.35	-0.73	-0.114 (0.872)	0.206 (0.957)	0.54	0.32
	138	DUOX2	0.314 (0.691)	-0.566 (1.934)	0.44	-0.88	-0.211 (0.942)	0.379 (0.569)	0.23	0.59
	139	GTF2I	0.52 (0.696)	-0.39 (0.183)	0.01	-0.96	0.241 (0.840)	-0.434 (0.608)	0.14	-0.68
	140	NCF1	0.128 (1.145)	-0.231 (1.147)	0.58	-0.36	-0.223 (0.989)	0.401 (1.533)	0.37	0.62
	141	NCF2	0.651 (1.200)	-0.621(0.886)	0.04	-1.31	-0.225 (1.198)	0.405 (1.314)	0.38	0.63
	142	NOS2	0.321 (1.260)	-0.578 (1.218)	0.22	-0.90	-0.026 (0.968)	0.047 (0.689)	0.89	0.07
	143	NOX5	0.348 (0.995)	-0.626 (0.647)	0.07	-0.97	-0.131 (1.064)	0.236 (0.628)	0.90	0.37
	144	PREX1	0.257 (1.101)	-0.463 (0.695)	0.21	-0.72	-0.045 (1.071)	0.082 (1.280)	0.85	0.13
	145	PRG3	0.695 (1.436)	-1.251 (0.292)	0.00	-1.95	0.151 (0.556)	-0.272 (1.098)	0.35	-0.42
	146	UCP2	-0.158 (1.533)	0.284 (1.208)	0.59	0.44	0.093 (1.119)	-0.167 (0.921)	0.67	-0.26
**Other Peroxidases**	147	CAT	-0.480 (1.254)	0.864 (1.748)	0.04	1.34	0.023 (1.315)	-0.041 (0.348)	0.89	-0.06
	148	CSDE1	0.033 (1.212)	-0.060 (0.808)	0.88	-0.09	0.029 (1.212)	-0.052 (0.609)	0.89	-0.08
	149	CYBB	0.149 (1.160)	-0.269 (1.204)	0.53	-0.42	0.080 (1.420)	-0.144 (0.474)	0.67	-0.22

	150	GPR156	0.211 (1.397)	-0.379 (1.281)	0.61	-0.59	-0.187 (1.223)	0.337 (0.847)	0.41	0.52
	151	IPCEF1	-0.049 (0.771)	0.089 (1.134)	0.90	0.14	-0.052 (1.273)	0.093 (0.665)	0.82	0.15
	152	LPO	0.034 (1.350)	-0.062 (0.982)	0.89	-0.10	-0.033 (1.041)	0.060 (0.599)	1.00	0.09
	153	MGST3	0.273 (1.333)	-0.492 (1.083)	0.30	-0.77	0.067 (0.772)	-0.120 (0.562)	0.65	-0.19
	154	MPO	0.088 (0.981)	-0.158 (1.742)	0.52	-0.25	-0.371 (0.906)	0.668 (0.965)	0.07	1.04
	155	PTGS1	-0.224 (1.092)	0.404 (0.364)	0.14	0.63	0.577(1.031)	-0.528(0.521)	0.05	-3.03
	156	PTGS2	0.014 (1.461)	-0.024 (1.169)	0.96	-0.04	0.239 (0.951)	-0.429 (0.765)	0.20	-0.67
	157	PXDN	-0.702 (1.118)	1.263 (1.635)	0.04	1.97	-0.193 (0.978)	0.347 (0.966)	0.34	0.54
	158	PXDNL	0.263 (0.970)	-0.474 (1.929)	0.35	-0.74	-0.099 (0.968)	0.178 (0.799)	0.60	0.28
	159	TPO	0.417 (1.008)	-0.750 (1.235)	0.08	-1.17	-0.073 (1.239)	0.131 (0.756)	0.75	0.20
**Oxidative Stress Responsive Genes**	160	ANGPTL 7	-0.463 (1.431)	0.833 (0.853)	0.09	1.30	-0.218 (1.207)	0.392 (0.929)	0.35	0.61
	161	ATOX1	0.216 (0.728)	-0.389 (1.540)	0.33	-0.61	-0.104 (1.017)	0.188 (0.726)	0.58	0.29
	163	CYGB	-1.540 (0.434)	2.771 (4.617)	0.00	4.31	-0.330 (1.244)	0.593 (0.864)	0.17	0.92
	164	DGKK	0.153 (1.342)	-0.275 (2.098)	0.90	-0.43	-0.211 (1.215)	0.379 (0.737)	0.35	0.59
	165	DHCR24	-0.856 (1.194)	1.541 (2.225)	0.02	2.40	-0.247 (0.886)	0.444 (0.585)	0.30	0.69
	166	DUSP1	0.118 (1.613)	-0.212 (1.298)	0.70	-0.33	-0.403 (1.215)	0.725 (1.749)	0.18	1.13
	167	EPX	0.227 (1.270)	-0.409 (0.893)	0.34	-0.64	-0.219 (0.910)	0.906 (0.993)	0.04	3.08
	168	FOXM1	0.309 (1.145)	-0.557 (1.126)	0.20	-0.87	0.320 (0.797)	-0.576 (1.027)	0.09	-0.90
	169	GCLC	0.374 (0.538)	-0.674 (1.341)	0.16	-1.05	-0.186 (1.259)	0.335 (0.262)	0.26	0.52
	170	GCLM	0.022 (0.973)	-0.039 (0.753)	0.91	-0.06	-0.017 (1.166)	0.030 (0.573)	0.94	0.05
	171	GLRX2	-0.122 (1.419)	0.220 (0.883)	0.64	0.34	-0.167 (1.201)	0.301 (0.783)	0.45	0.47
	172	HMOX1	-0.278 (1.205)	1.501 (1.080)	0.03	7.78	-0.408 (0.525)	0.735 (0.370)	0.00	1.14
	173	HSPA1A	-3.379 (0.382)	6.083 (16.523)	0.00	9.46	-0.099 (1.234)	0.178 (1.245)	0.70	0.28
	174	KRT1	-0.352 (1.012)	0.634 (1.776)	0.36	0.99	-0.300(0.821)	1.051(1.189)	0.03	3.87
	175	MBL2	-1.163 (1.167)	2.093 (3.328)	0.04	3.26	0.019 (1.077)	-0.034 (0.640)	0.92	-0.05
	176	MSRA	0.314 (0.816)	-0.565 (1.311)	0.14	-0.88	-0.187 (1.038)	0.337 (0.269)	0.18	0.52
	177	MTL5	0.106 (1.319)	-0.191 (0.851)	0.44	-0.30	0.021 (0.789)	-0.038 (0.838)	0.90	-0.06
	178	NME5	0.530 (1.396)	-0.403(0.946)	0.03	-0.98	-0.417 (1.256)	0.751 (0.824)	0.09	1.17
	179	NQO1	-0.129 (1.440)	0.231 (1.483)	0.67	0.36	-0.386 (1.161)	0.695 (0.907)	0.10	1.08
	180	NUDT1	0.285 (1.300)	-0.513 (1.569)	0.33	-0.80	-0.154 (0.867)	0.278 (1.201)	0.45	0.43
	181	OXR1	-0.050 (1.702)	0.089 (0.583)	0.86	0.14	0.136 (1.261)	-0.244 (0.411)	0.43	-0.38
	182	OXSR1	-0.168 (1.268)	0.303 (0.683)	0.46	0.47	-0.005 (0.970)	0.009 (0.582)	0.98	0.01
	183	PDLIM1	0.130 (0.925)	-0.235 (0.979)	0.50	-0.37	0.166 (0.821)	-0.299 (0.750)	0.32	-0.47
	184	PNKP	-0.132 (1.616)	0.237 (1.562)	0.69	0.37	-0.381 (1.165)	0.687 (1.047)	0.12	1.07
	185	PRDX2	-0.120 (1.658)	0.216 (0.849)	0.68	0.34	-0.321 (0.955)	0.579 (1.198)	0.15	0.90
	186	PRDX5	-0.098 (1.779)	0.177 (1.028)	0.76	0.28	-0.066 (1.398)	0.119 (0.864)	0.79	0.19
	187	PRDX6	-0.023 (1.369)	0.041 (0.796)	0.93	0.06	0.221 (0.933)	-0.398 (0.636)	0.21	-0.62
	188	PRNP	-0.861 (1.391)	1.550 (1.707)	0.03	2.41	0.044 (1.148)	-0.080 (0.831)	0.80	-0.12
	189	RNF7	-0.291 (1.319)	0.523 (1.424)	0.30	0.81	0.205 (1.105)	-0.369 (0.740)	0.32	-0.57

	190	SCARA3	0.223 (1.149)	-0.401 (0.638)	0.29	-0.62	-0.197 (0.826)	0.354 (0.599)	0.22	0.55
	191	SEPP1	-0.167 (0.971)	0.301 (1.241)	0.45	0.47	-0.209 (1.205)	0.376 (0.329)	0.20	0.59
	192	SGK2	-0.168 (1.062)	0.303 (1.193)	0.46	0.47	0.123 (0.903)	-0.221 (0.811)	0.49	-0.34
	193	SIRT2	0.217 (1.278)	-0.391 (0.918)	0.37	-0.61	0.078 (0.932)	-0.141 (0.723)	0.66	-0.22
	194	SQSTM1	-0.121 (1.410)	0.218 (1.236)	0.80	0.34	-0.072 (0.960)	0.130 (1.198)	0.73	0.20
	195	SRXN1	-0.671 (0.470)	1.208 (1.499)	0.01	1.88	-0.025 (1.221)	0.045 (0.539)	0.91	0.07
	196	STK25	0.126 (1.336)	-0.226 (1.596)	0.67	-0.35	-0.062 (1.144)	0.112 (0.906)	0.77	0.17
	197	TTN	0.022 (1.185)	-0.040 (0.959)	0.92	-0.06	-0.215 (1.165)	0.386 (0.790)	0.33	0.60
**Oxygen Transporters**	198	MB	0.371 (1.472)	-0.667 (0.872)	0.11	-1.04	0.024 (0.963)	-0.043 (1.093)	0.91	-0.07
**Pathway Activity Signature Genes**	199	AKR1C2	0.70 (1.110)	-0.71 (0.861)	0.03	-1.45	-0.126 (1.290)	0.227 (0.542)	0.58	0.35
	200	BAG2	-0.505 (2.342)	0.909 (1.189)	0.02	1.41	-0.242 (0.934)	0.435 (0.874)	0.61	0.68
	201	FHL2	0.113 (1.273)	-0.203 (0.689)	0.36	-0.32	-0.190 (1.073)	0.342 (0.603)	0.33	0.53
	202	GLA	0.82 (1.593)	-0.92 (0.263)	0.01	-1.79	0.112 (0.985)	-0.201 (1.167)	0.60	-0.31
	203	HSP90AA1	-0.364 (1.988)	0.656 (1.346)	0.36	1.02	-0.141 (1.543)	0.254 (0.709)	0.52	0.40
	204	LHPP	-0.193 (1.557)	0.348 (1.254)	0.44	0.54	-0.145 (1.026)	0.261 (1.053)	0.50	0.41
	205	NCOA7	0.038 (1.028)	-0.069 (0.697)	0.84	-0.11	-0.136 (1.213)	0.244 (0.434)	0.52	0.38
	206	PTGR1	-0.150 (1.119)	0.271 (2.018)	0.62	0.42	-0.060 (0.927)	0.108 (0.653)	0.73	0.17
	207	SLC7A11	-0.012 (1.077)	0.022 (1.094)	0.96	0.03	-0.300 (1.201)	0.540 (0.692)	0.18	0.84
	208	SPINK1	0.015 (0.876)	-0.027 (1.965)	0.08	-0.04	-0.340 (0.913)	0.612 (0.263)	0.11	0.95
	209	TRAPPC6A	-0.193 (1.189)	0.348 (1.499)	0.47	0.54	-0.217 (0.863)	0.390 (1.186)	0.29	0.61
	210	TXNRD1	-0.116 (1.161)	0.210 (1.207)	0.52	0.33	0.253 (0.953)	-0.455 (0.351)	0.14	-0.71
**Peroxiredoxins (TPx)**	211	PRDX1	0.201 (1.292)	-0.361 (1.283)	0.45	-0.56	-0.087 (1.280)	0.157 (0.515)	0.69	0.24
	212	PRDX3	0.081 (1.170)	-0.145 (1.537)	0.70	-0.23	-0.195 (0.582)	0.351 (0.818)	0.61	0.55
	213	PRDX4	-1.503 (0.216)	2.705 (7.031)	0.03	4.21	0.197 (1.040)	-0.354 (0.553)	0.30	-0.55
**Superoxide Dismutases (SOD)**	214	SOD1	-0.735 (0.964)	1.324 (1.603)	0.01	2.06	-0.061 (0.908)	1.110 (0.535)	0.01	2.84
	215	SOD2	-0.158 (1.796)	0.284 (1.095)	0.63	0.44	-0.135 (1.449)	0.244 (0.726)	0.80	0.38
	216	SOD3	-0.280 (1.400)	0.503 (1.472)	0.34	0.78	-0.037 (1.037)	0.067 (0.769)	0.85	0.10
**Macrophages and Iron processing**	217	CD163	-1.898 (0.174)	3.416 (1.567)	0.00	5.31	-1.765 (0.096)	3.176 (1.304)	0.00	4.94
	218	ARL6IP5	0.403 (0.454)	-0.725 (1.027)	0.07	-1.13	-0.021 (0.668)	0.038 (0.910)	0.89	0.06
	219	C1RL	-1.128 (0.216)	2.030 (3.098)	0.00	3.16	-0.166 (1.806)	0.300 (0.182)	0.46	0.47
	220	CD36	-0.283 (1.170)	0.509 (0.231)	0.08	0.79	0.551 (1.612)	-0.992 (0.707)	0.07	-1.54
	221	CD68	-1.237 (1.240)	2.227 (2.020)	0.01	3.46	0.468 (2.008)	-0.842 (0.972)	0.20	-1.31
	222	CSF1	-0.081 (1.073)	0.145 (0.833)	0.69	0.23	0.133 (1.534)	-0.239 (0.678)	0.62	-0.37
	223	HP	-17.132 (44.209)	30.838 (36.572)	0.04	47.97	-0.025 (1.521)	0.044 (1.459)	1.00	0.07
	224	IL4	0.027 (0.993)	-0.049 (0.650)	1.00	-0.08	0.270 (1.117)	-0.485 (1.145)	0.25	-0.76
	225	IL6	0.249 (1.556)	-0.448 (0.617)	0.36	-0.70	-0.003 (0.873)	0.006 (0.741)	1.00	0.01
	226	LGALS3	0.000 (1.508)	-0.000 (1.690)	1.00	0.00	0.471 (1.880)	-0.847 (1.145)	0.18	-1.32
	227	TAC1	-0.048 (1.480)	0.087 (1.225)	0.80	0.14	-0.129 (1.033)	0.232 (1.042)	0.54	0.36

Metalloproteases	228	ADAM2	0.233 (1.242)	-0.419 (0.946)	0.33	-0.65	-0.165 (1.151)	0.298 (0.927)	0.46	0.46
	229	ADAM7	0.121 (1.673)	-0.217 (1.085)	0.69	-0.34	0.178 (1.169)	-0.321 (1.234)	0.47	-0.50
	230	ADAM8	0.198 (1.650)	-0.356 (1.290)	0.53	-0.55	-0.167 (1.106)	0.301 (1.478)	0.51	0.47
	231	ADAM9	0.199 (0.945)	-0.358 (0.925)	0.31	-0.56	-0.050 (1.047)	0.091 (1.058)	0.81	0.14
	232	ADAM10	-0.40(1.257)	1.28(1.146)	0.03	1.64	0.420 (1.663)	-0.756 (0.737)	0.16	-1.18
	233	ADAM11	0.452 (1.163)	-0.814 (0.768)	0.05	-1.27	-0.149 (1.158)	0.268 (0.760)	0.49	0.42
	234	ADAM12	0.162 (1.150)	-0.292 (1.689)	0.56	-0.45	0.019 (0.786)	-0.035 (0.986)	0.91	-0.05
	235	ADAM15	-1.023 (0.521)	1.842 (3.465)	0.01	2.87	0.292 (1.357)	-0.525 (0.556)	0.23	-0.82
	107	ADAM17	-0.804 (0.226)	1.448 (0.794)	0.00	2.25	-0.823 (0.390)	1.482 (0.971)	0.00	2.31
	236	ADAM18	0.293 (1.089)	-0.528 (1.087)	0.11	-0.82	0.190 (0.729)	-0.342 (1.294)	0.34	-0.53
	237	ADAM19	-0.153 (1.725)	0.275 (0.715)	0.90	0.43	0.057 (0.722)	-0.102 (0.731)	0.70	-0.16
	238	ADAM20	-0.001 (1.056)	0.002 (1.596)	1.00	0.00	-0.202 (1.501)	0.363 (0.649)	1.00	0.57
	239	ADAM21	0.125 (1.308)	-0.226 (1.062)	0.62	-0.35	0.130 (0.915)	-0.235 (1.497)	0.80	-0.37
	240	ADAM22	0.162 (1.140)	-0.291 (1.110)	0.49	-0.45	-0.187 (1.372)	0.337 (0.942)	0.46	0.52
	241	ADAM23	0.182 (1.313)	-0.327 (1.281)	0.50	-0.51	0.210 (0.791)	-0.378 (1.544)	0.36	-0.59
	242	ADAM28	0.200 (1.439)	-0.361 (0.907)	0.45	-0.56	0.166 (1.220)	-0.298 (1.458)	0.54	-0.46
	243	ADAM29	0.268 (1.294)	-0.483 (1.011)	0.29	-0.75	0.030 (1.187)	-0.055 (0.965)	0.89	-0.09
	244	ADAM30	-0.146 (1.118)	0.262 (0.771)	0.49	0.41	-0.023 (0.843)	0.041 (0.690)	0.89	0.06
	245	ADAM32	0.210 (1.422)	-0.377 (0.945)	0.43	-0.59	0.343 (0.738)	-0.618 (0.821)	0.15	-0.96
	246	ADAM33	0.463 (0.852)	-0.834 (1.737)	0.08	-1.30	-0.103 (1.176)	0.185 (0.843)	0.64	0.29

Liver biopsy transcriptome in blue, PBMC transcriptome in green and those significantly different stated in Pink ($p<0.05$)

Supplementary Table-5: PBMC transcriptome : Genes Differentially expressed in GR.A:SAHIO vs. GR.B:SAHNIO

Univariate analysis PBMC of GR.A:SAHIO vs GR.B:SAHNIO						
SR.No	GENE	Mean (SD) of GR.B:SAHNIO	Mean (SD) of GR.A:SAHIO	Fold Change LOG GR.A:SAHIO/GR.B:SAHNIO	p-value	GR.A:SAHIO/GR.B:SAHNIO
1	**ERBB2**	-0.415(0.486)	1.258(0.818)	5.33	0.00	UP
2	**CD163**	-1.765 (0.096)	3.176 (1.304)	4.94	0.00	UP
3	**NOTCH3**	-0.349(0.610)	1.140(0.402)	4.44	0.00	UP
4	KRT1	-0.300(0.821)	1.051(1.189)	3.87	0.03	UP
5	CXCL2	-0.295 (0.805)	1.042 (0.892)	3.81	0.01	UP
6	CCL24	-0.279 (0.736)	1.0143 (1.002)	3.65	0.02	UP
7	**HAMP**	-0.259(0.651)	0.977(0.352)	3.45	0.01	UP
8	GPR149	-1.204 (0.248)	2.168 (5.232)	3.37	0.01	UP
9	EPX	-0.219 (0.910)	0.906 (0.993)	3.08	0.04	UP
10	LENEP	-1.087 (0.433)	1.957 (5.830)	3.04	0.03	UP
11	NRIP2	-1.065 (0.509)	1.918 (6.125)	2.98	0.03	UP
12	CCL15	-0.194 (0.925)	0.861 (0.739)	2.87	0.05	UP
13	**SOD1**	-0.061 (0.908)	1.110 (0.535)	2.84	0.01	UP
14	EGFR	-0.161 (0.627)	0.802 (0.726)	2.62	0.02	UP
15	VPS45	-0.872 (0.595)	1.569 (4.806)	2.44	0.04	UP
16	**ADAM17**	-0.823 (0.390)	1.482 (0.971)	2.31	0.00	UP
17	BCL2L15	-0.791 (0.742)	1.425 (3.496)	2.22	0.04	UP
18	RSPH10B	-0.769 (0.648)	1.384 (1.347)	2.15	0.01	UP
19	SNAPIN	-0.764 (0.770)	1.376 (2.459)	2.14	0.03	UP
20	**HKR1**	-0.735 (0.249)	1.323 (3.224)	2.06	0.01	UP
21	USP17L30	-0.733 (0.409)	1.319 (3.161)	2.05	0.02	UP
22	RSPH10B2	-0.726 (0.723)	1.307 (1.050)	2.03	0.01	UP
23	SYT3	-0.691 (0.237)	1.244 (3.530)	1.94	0.03	UP
24	APOL5	-0.687 (0.607)	1.236 (2.324)	1.92	0.03	UP
25	OCM2	-0.685 (0.677)	1.233 (1.123)	1.92	0.00	UP
26	DGAT2	-0.683 (0.315)	1.229 (3.460)	1.91	0.03	UP
27	OR5C1	-0.650 (0.471)	1.170 (2.678)	1.82	0.03	UP
28	LCTL	-0.626 (0.708)	1.127 (1.656)	1.75	0.02	UP
29	ERAL1	-0.596 (0.660)	1.072 (1.131)	1.67	0.01	UP
30	UBE2C	-0.596 (0.404)	1.072 (2.541)	1.67	0.04	UP
31	ARSG	-0.593 (0.589)	1.068 (2.169)	1.66	0.02	UP
32	NDUFAF1	-0.567 (0.797)	1.020 (0.844)	1.59	0.01	UP
33	CD79A	-0.566 (0.483)	1.019 (2.250)	1.59	0.04	UP
34	C19orf45	-0.559 (1.434)	1.006 (1.990)	1.57	0.03	UP

35	TSKS	-0.556 (0.439)	1.000 (2.231)	1.56	0.04	UP
36	ACTL6B	-0.550 (0.832)	0.990 (2.469)	1.54	0.02	UP
37	GJC1	-0.538 (0.630)	0.969 (0.472)	1.51	0.00	UP
38	GPATCH1	-0.522 (0.527)	0.940 (2.578)	1.46	0.05	UP
39	NLRP12	-0.520 (0.398)	0.937 (1.107)	1.46	0.02	UP
40	COPS7A	-0.519 (0.264)	0.934 (2.574)	1.45	0.03	UP
41	DUSP14	-0.517 (0.453)	0.931 (1.116)	1.45	0.01	UP
42	PGA4	-0.508 (1.443)	0.914 (1.229)	1.42	0.04	UP
43	sep_3	-0.486 (0.492)	0.875 (0.974)	1.36	0.00	UP
44	DNAH3	-0.483 (0.746)	0.869 (0.701)	1.35	0.01	UP
45	CALN1	-0.474 (0.679)	0.854 (1.036)	1.33	0.05	UP
46	ARAP3	-0.463 (0.361)	0.834 (2.301)	1.30	0.04	UP
47	GTF2IRD2B	-0.446 (0.482)	0.803 (0.500)	1.25	0.00	UP
48	SMTNL2	-0.436 (0.679)	0.784 (1.519)	1.22	0.04	UP
49	ASAP3	-0.429 (0.493)	0.772 (0.963)	1.20	0.01	UP
50	IGSF23	-0.425 (0.572)	0.764 (0.794)	1.19	0.03	UP
51	WDR45B	-0.424 (0.317)	0.764 (1.511)	1.19	0.04	UP
52	GIP	-0.413 (0.559)	0.744 (0.715)	1.16	0.01	UP
53	**HMOX1**	-0.408 (0.525)	0.735 (0.370)	1.14	0.00	UP
54	NPC1L1	-0.407 (0.291)	0.733 (1.644)	1.14	0.02	UP
55	ZNF491	-0.405 (0.838)	0.730 (0.516)	1.14	0.03	UP
56	PSMD9	-0.399 (0.456)	0.717 (0.793)	1.12	0.01	UP
57	RNF122	-0.398 (0.771)	0.717 (0.775)	1.12	0.03	UP
58	WIBG	-0.398 (0.600)	0.717 (0.583)	1.12	0.02	UP
59	TM4SF5	-0.395 (0.664)	0.711 (0.862)	1.11	0.02	UP
60	ASRGL1	-0.389 (0.684)	0.700 (0.797)	1.09	0.04	UP
61	FAM222B	-0.385 (0.401)	0.693 (0.793)	1.08	0.01	UP
62	CPAMD8	-0.384 (0.602)	0.691 (0.613)	1.08	0.02	UP
63	BFAR	-0.383 (0.562)	0.690 (0.658)	1.07	0.01	UP
64	ULK4	-0.382 (0.879)	0.688 (0.451)	1.07	0.03	UP
65	CCDC63	-0.382 (0.344)	0.687 (1.264)	1.07	0.02	UP
66	HDC	-0.378 (0.852)	0.681 (0.547)	1.06	0.04	UP
67	CCL26	-0.375 (0.523)	0.675 (0.902)	1.05	0.02	UP
68	GTF2IRD2	-0.371 (0.467)	0.668 (0.572)	1.04	0.01	UP
69	FAM107A	-0.366 (0.545)	0.659 (0.653)	1.03	0.01	UP
70	ZNF337	-0.365 (0.655)	0.656 (0.627)	1.02	0.01	UP
71	TBC1D20	-0.364 (0.420)	0.656 (0.625)	1.02	0.00	UP
72	PTK7	-0.359 (0.579)	0.646 (0.672)	1.01	0.04	UP
73	ADORA2B	-0.352 (0.700)	0.633 (0.556)	0.99	0.02	UP
74	PRKAG3	-0.351 (0.514)	0.632 (1.128)	0.98	0.05	UP
75	C12orf76	-0.345 (0.639)	0.621 (0.717)	0.97	0.04	UP

76	TTC21A	-0.342 (0.524)	0.615 (0.943)	0.96	0.03	UP
77	CTCFL	-0.337 (0.921)	0.607 (0.365)	0.94	0.04	UP
78	TMEM214	-0.335 (0.542)	0.603 (0.548)	0.94	0.03	UP
79	CYBRD1	-0.320 (0.465)	0.577 (0.743)	0.90	0.02	UP
80	SYTL3	-0.301 (0.508)	0.542 (0.551)	0.84	0.03	UP
81	FXYD4	-0.291 (0.653)	0.524 (0.519)	0.82	0.04	UP
82	CHKA	-0.291 (0.630)	0.523 (0.455)	0.81	0.02	UP
83	SLBP	-0.290 (0.622)	0.521 (0.542)	0.81	0.05	UP
84	ZFP90	-0.289 (0.563)	0.520 (0.631)	0.81	0.03	UP
85	SH3PXD2B	-0.238 (0.592)	0.428 (0.413)	0.67	0.04	UP
86	TRAF3IP1	-0.235 (0.480)	0.423 (0.591)	0.66	0.04	UP
87	KRT8	-0.199 (0.493)	0.357 (0.380)	0.56	0.04	UP
88	LRP8	0.296 (0.689)	-0.533 (0.689)	-0.83	0.05	Down
89	C6orf89	0.299 (0.842)	-0.538 (0.501)	-0.84	0.03	Down
90	SCHIP1	0.304 (0.752)	-0.548 (0.792)	-0.85	0.05	Down
91	AGTRAP	0.324 (0.739)	-0.583 (0.683)	-0.91	0.04	Down
92	PGAM5	0.324 (1.147)	-0.583 (0.730)	-0.91	0.05	Down
93	ZNF641	0.328 (0.595)	-0.590 (0.726)	-0.92	0.04	Down
94	TFRC	0.334 (0.730)	-0.601 (0.499)	-0.94	0.03	Down
95	CCDC116	0.336 (1.050)	-0.604 (0.414)	-0.94	0.04	Down
96	POTEF	0.339 (0.773)	-0.611 (0.521)	-0.95	0.03	Down
97	ERMAP	0.345 (0.879)	-0.621 (0.632)	-0.97	0.04	Down
98	SCML4	0.348 (0.841)	-0.626 (0.606)	-0.97	0.03	Down
99	MYT1L	0.348 (0.943)	-0.627 (0.610)	-0.98	0.04	Down
100	**C2CD2L**	0.349 (0.602)	-0.628 (0.836)	-0.98	0.04	Down
101	HTT	0.351 (0.608)	-0.631 (0.583)	-0.98	0.03	Down
102	ILF2	0.351 (0.916)	-0.632 (0.577)	-0.98	0.04	Down
103	ARSB	0.354 (0.977)	-0.637 (0.661)	-0.99	0.02	Down
104	ZNF587	0.354 (0.854)	-0.637 (0.595)	-0.99	0.04	Down
105	**TUSC2**	0.360 (1.028)	-0.648 (0.371)	-1.01	0.02	Down
106	KIAA0391	0.361 (0.826)	-0.650 (0.706)	-1.01	0.03	Down
107	TRAM1	0.361 (0.802)	-0.650 (0.622)	-1.01	0.02	Down
108	C20orf27	0.363 (1.462)	-0.653 (0.636)	-1.02	0.04	Down
109	ANKRD10	0.363 (1.026)	-0.654 (0.440)	-1.02	0.04	Down
110	SP140	0.364 (0.666)	-0.655 (0.609)	-1.02	0.01	Down
111	HIST1H2BC	0.365 (0.904)	-0.657 (0.481)	-1.02	0.02	Down
112	MLH1	0.367 (0.666)	-0.661 (0.660)	-1.03	0.03	Down
113	ARHGAP21	0.368 (1.021)	-0.663 (0.583)	-1.03	0.04	Down
114	STOM	0.368 (0.850)	-0.663 (0.539)	-1.03	0.01	Down
115	MUTYH	0.370 (1.209)	-0.665 (0.690)	-1.04	0.04	Down
116	MED26	0.371 (1.120)	-0.668 (0.434)	-1.04	0.02	Down

117	KIR2DS4	0.372 (1.036)	-0.669 (0.491)	-1.04	0.03	Down
118	CEPT1	0.374 (0.800)	-0.674 (1.046)	-1.05	0.05	Down
119	HIRA	0.374 (0.914)	-0.674 (0.782)	-1.05	0.04	Down
120	STRADA	0.374 (0.973)	-0.674 (0.910)	-1.05	0.03	Down
121	MED8	0.377 (0.692)	-0.678 (0.725)	-1.06	0.03	Down
122	API5	0.378 (0.612)	-0.681 (0.868)	-1.06	0.03	Down
123	MKLN1	0.379 (0.944)	-0.682 (0.541)	-1.06	0.03	Down
124	RAB28	0.380 (1.127)	-0.684 (0.463)	-1.06	0.03	Down
125	TM2D2	0.380 (0.912)	-0.684 (0.983)	-1.06	0.04	Down
126	ZMAT1	0.382 (0.915)	-0.688 (0.688)	-1.07	0.03	Down
127	SLC46A2	0.384 (0.938)	-0.692 (0.736)	-1.08	0.05	Down
128	MRPL44	0.386 (0.773)	-0.695 (0.878)	-1.08	0.05	Down
129	CSRP2BP	0.386 (1.101)	-0.696 (0.681)	-1.08	0.05	Down
130	TNFRSF25	0.387 (1.239)	-0.696 (0.525)	-1.08	0.05	Down
131	PHKG2	0.387 (1.549)	-0.697 (0.457)	-1.08	0.03	Down
132	USP24	0.389 (0.972)	-0.701 (0.516)	-1.09	0.02	Down
133	ZMYM6	0.389 (0.764)	-0.701 (0.874)	-1.09	0.03	Down
134	B3GAT3	0.390 (0.947)	-0.701 (0.603)	-1.09	0.05	Down
135	C12orf45	0.390 (0.795)	-0.702 (0.751)	-1.09	0.03	Down
136	C8orf82	0.390 (1.103)	-0.702 (0.669)	-1.09	0.04	Down
137	ZNF558	0.390 (0.908)	-0.702 (0.824)	-1.09	0.02	Down
138	ZNF852	0.391 (1.508)	-0.704 (0.325)	-1.10	0.02	Down
139	HYAL3	0.391 (1.550)	-0.705 (0.874)	-1.10	0.03	Down
140	DENND2D	0.392 (0.907)	-0.705 (0.660)	-1.10	0.03	Down
141	KRAS	0.392 (0.967)	-0.705 (0.692)	-1.10	0.04	Down
142	TCF12	0.392 (0.855)	-0.705 (0.961)	-1.10	0.03	Down
143	KDM5A	0.392 (1.587)	-0.706 (0.495)	-1.10	0.04	Down
144	PRDM4	0.393 (1.068)	-0.708 (0.498)	-1.10	0.02	Down
145	CMTR2	0.394 (1.002)	-0.709 (0.514)	-1.10	0.03	Down
146	FAS	0.394 (0.853)	-0.709 (0.784)	-1.10	0.03	Down
147	RAD52	0.395 (0.926)	-0.711 (0.581)	-1.11	0.04	Down
148	SNRPA1	0.395 (0.910)	-0.711 (0.524)	-1.11	0.04	Down
149	DDX56	0.396 (0.971)	-0.713 (0.541)	-1.11	0.04	Down
150	CHD9	0.399 (0.974)	-0.719 (0.734)	-1.12	0.04	Down
151	PDHX	0.399 (0.844)	-0.719 (0.871)	-1.12	0.03	Down
152	MRPS26	0.401 (1.402)	-0.722 (0.476)	-1.12	0.04	Down
153	FKBP7	0.402 (0.900)	-0.723 (0.490)	-1.13	0.02	Down
154	HADHB	0.402 (0.872)	-0.724 (0.503)	-1.13	0.03	Down
155	MCFD2	0.402 (1.296)	-0.724 (0.457)	-1.13	0.04	Down
156	KCNV1	0.403 (1.249)	-0.725 (0.510)	-1.13	0.03	Down
157	SLC35B2	0.404 (1.284)	-0.727 (0.328)	-1.13	0.01	Down

158	AKAP9	0.405 (1.392)	-0.729 (0.422)	-1.13	0.05	Down
159	SNTB1	0.406 (1.467)	-0.730 (0.432)	-1.14	0.04	Down
160	PBX1	0.409 (1.695)	-0.736 (0.398)	-1.15	0.04	Down
161	ZBTB38	0.410 (1.138)	-0.739 (0.515)	-1.15	0.02	Down
162	RBM17	0.411 (1.081)	-0.739 (0.573)	-1.15	0.02	Down
163	KAT5	0.412 (1.464)	-0.741 (0.391)	-1.15	0.05	Down
164	ARL15	0.413 (1.513)	-0.744 (0.470)	-1.16	0.04	Down
165	MGAT5	0.413 (1.043)	-0.744 (0.684)	-1.16	0.03	Down
166	NF2	0.414 (1.290)	-0.745 (0.483)	-1.16	0.03	Down
167	KANSL1	0.416 (0.918)	-0.749 (0.639)	-1.17	0.02	Down
168	SSR1	0.417 (0.962)	-0.750 (0.641)	-1.17	0.03	Down
169	HAUS3	0.417 (0.938)	-0.751 (0.722)	-1.17	0.02	Down
170	TRIO	0.417 (0.851)	-0.751 (0.425)	-1.17	0.00	Down
171	BCL10	0.418 (1.218)	-0.752 (0.516)	-1.17	0.02	Down
172	C14orf166B	0.418 (1.494)	-0.753 (0.616)	-1.17	0.03	Down
173	MTFR1L	0.419 (1.169)	-0.753 (0.462)	-1.17	0.02	Down
174	PITPNC1	0.419 (1.060)	-0.755 (0.767)	-1.17	0.05	Down
175	MTMR11	0.420 (1.142)	-0.756 (0.481)	-1.18	0.02	Down
176	GDAP2	0.421 (1.275)	-0.757 (0.827)	-1.18	0.05	Down
177	SLC25A46	0.421 (0.965)	-0.757 (0.649)	-1.18	0.02	Down
178	TMEM131	0.421 (0.470)	-0.757 (0.739)	-1.18	0.02	Down
179	LDOC1L	0.422 (1.303)	-0.760 (0.493)	-1.18	0.04	Down
180	NSMAF	0.422 (1.186)	-0.760 (0.562)	-1.18	0.05	Down
181	MAN2A1	0.423 (1.060)	-0.761 (0.553)	-1.18	0.02	Down
182	SLC30A7	0.423 (0.693)	-0.762 (1.012)	-1.19	0.04	Down
183	NMD3	0.425 (0.729)	-0.765 (0.830)	-1.19	0.02	Down
184	AAGAB	0.425 (1.087)	-0.766 (0.425)	-1.19	0.01	Down
185	EEA1	0.425 (1.682)	-0.766 (0.456)	-1.19	0.03	Down
186	HNRNPR	0.426 (0.818)	-0.766 (0.651)	-1.19	0.02	Down
187	FYTTD1	0.426 (0.914)	-0.767 (0.449)	-1.19	0.02	Down
188	GTF3A	0.427 (0.983)	-0.768 (0.509)	-1.20	0.01	Down
189	GALNT6	0.427 (1.196)	-0.769 (0.605)	-1.20	0.03	Down
190	WHSC1L1	0.427 (1.262)	-0.769 (0.458)	-1.20	0.04	Down
191	NBPF16	0.428 (0.972)	-0.770 (0.627)	-1.20	0.02	Down
192	TRAPPC11	0.428 (1.145)	-0.770 (0.746)	-1.20	0.04	Down
193	LRRC37A2	0.430 (1.290)	-0.773 (0.464)	-1.20	0.02	Down
194	MRPL55	0.430 (1.469)	-0.774 (0.329)	-1.20	0.03	Down
195	SYPL1	0.431 (0.940)	-0.776 (0.532)	-1.21	0.02	Down
196	ST7	0.432 (1.702)	-0.778 (0.438)	-1.21	0.04	Down
197	ST8SIA5	0.432 (2.097)	-0.778 (0.354)	-1.21	0.05	Down
198	DARS	0.433 (1.031)	-0.779 (0.535)	-1.21	0.02	Down

199	EIF2S2	0.433 (0.715)	-0.780 (0.715)	-1.21	0.01	Down
200	CCDC169-SOHLH2	0.434 (0.912)	-0.782 (0.578)	-1.22	0.03	Down
201	LOC729020	0.434 (1.781)	-0.782 (0.476)	-1.22	0.05	Down
202	NPHP3	0.435 (0.873)	-0.783 (0.586)	-1.22	0.04	Down
203	MTMR2	0.436 (0.562)	-0.785 (0.473)	-1.22	0.01	Down
204	SUZ12	0.437 (1.143)	-0.786 (0.791)	-1.22	0.04	Down
205	DGCR8	0.437 (1.470)	-0.787 (0.272)	-1.22	0.01	Down
206	PKIA	0.437 (1.194)	-0.787 (0.357)	-1.22	0.02	Down
207	ICA1L	0.438 (0.702)	-0.788 (0.738)	-1.23	0.02	Down
208	CD3E	0.439 (0.876)	-0.789 (0.830)	-1.23	0.03	Down
209	MIER1	0.440 (1.132)	-0.792 (0.553)	-1.23	0.02	Down
210	SLC29A3	0.442 (1.098)	-0.795 (0.590)	-1.24	0.02	Down
211	ZNF513	0.442 (1.229)	-0.795 (0.354)	-1.24	0.04	Down
212	VEGFC	0.442 (2.263)	-0.796 (0.258)	-1.24	0.03	Down
213	STXBP6	0.443 (1.142)	-0.797 (0.435)	-1.24	0.02	Down
214	FAM175A	0.444 (1.396)	-0.799 (0.606)	-1.24	0.04	Down
215	MDM4	0.445 (1.106)	-0.801 (0.453)	-1.25	0.02	Down
216	POLR3E	0.445 (1.534)	-0.801 (0.380)	-1.25	0.02	Down
217	PRKRA	0.445 (1.233)	-0.801 (0.759)	-1.25	0.02	Down
218	ZNF436	0.445 (1.119)	-0.802 (0.501)	-1.25	0.04	Down
219	RAB6A	0.446 (1.463)	-0.802 (0.329)	-1.25	0.04	Down
220	NUTM1	0.447 (0.904)	-0.804 (1.051)	-1.25	0.04	Down
221	CEP85L	0.448 (1.227)	-0.806 (0.647)	-1.25	0.04	Down
222	TMX1	0.448 (0.854)	-0.807 (0.814)	-1.26	0.02	Down
223	USP17L27	0.448 (1.325)	-0.807 (0.315)	-1.26	0.02	Down
224	ARPC5L	0.449 (1.124)	-0.808 (0.415)	-1.26	0.00	Down
225	PDCD7	0.451 (1.227)	-0.812 (0.388)	-1.26	0.03	Down
226	NSFL1C	0.451 (1.176)	-0.813 (0.515)	-1.26	0.05	Down
227	WDR43	0.452 (0.906)	-0.813 (0.670)	-1.27	0.02	Down
228	PITPNB	0.453 (1.102)	-0.815 (0.511)	-1.27	0.02	Down
229	QTRTD1	0.453 (1.224)	-0.816 (0.591)	-1.27	0.04	Down
230	**SAR1B**	0.454 (1.206)	-0.816 (0.592)	-1.27	0.02	Down
231	NOL8	0.454 (0.794)	-0.817 (0.849)	-1.27	0.05	Down
232	TTLL5	0.454 (0.845)	-0.817 (0.728)	-1.27	0.01	Down
233	FER	0.455 (0.771)	-0.818 (0.946)	-1.27	0.03	Down
234	NMB	0.455 (1.226)	-0.818 (0.468)	-1.27	0.03	Down
235	DLX4	0.455 (1.157)	-0.820 (0.655)	-1.28	0.05	Down
236	BTBD10	0.456 (0.940)	-0.820 (0.614)	-1.28	0.04	Down
237	C16orf52	0.456 (1.379)	-0.820 (0.454)	-1.28	0.04	Down
238	HELZ	0.456 (1.438)	-0.820 (0.393)	-1.28	0.04	Down
239	CSNK1G3	0.457 (0.874)	-0.822 (0.789)	-1.28	0.02	Down

240	RNF111	0.457 (1.658)	-0.823 (0.560)	-1.28	0.03	Down
241	GOPC	0.458 (1.247)	-0.824 (0.711)	-1.28	0.02	Down
242	GALC	0.458 (0.946)	-0.825 (0.631)	-1.28	0.04	Down
243	COX18	0.460 (1.184)	-0.827 (0.523)	-1.29	0.01	Down
244	DNAJC25-GNG10	0.460 (1.269)	-0.828 (0.304)	-1.29	0.01	Down
245	EVI5	0.460 (0.952)	-0.828 (0.872)	-1.29	0.03	Down
246	LNPEP	0.461 (1.323)	-0.830 (0.324)	-1.29	0.01	Down
247	GUF1	0.464 (0.849)	-0.835 (0.749)	-1.30	0.03	Down
248	UBA6	0.464 (0.959)	-0.835 (0.973)	-1.30	0.02	Down
249	CNOT6	0.464 (1.106)	-0.836 (0.704)	-1.30	0.03	Down
250	FAM214B	0.465 (1.339)	-0.837 (0.387)	-1.30	0.03	Down
251	MRPS6	0.465 (1.101)	-0.837 (0.925)	-1.30	0.04	Down
252	TMEM18	0.465 (0.927)	-0.837 (0.739)	-1.30	0.04	Down
253	ZHX1	0.465 (1.327)	-0.838 (0.363)	-1.30	0.02	Down
254	TSPO	0.466 (1.472)	-0.838 (0.331)	-1.30	0.04	Down
255	TMEFF1	0.468 (1.030)	-0.842 (0.426)	-1.31	0.01	Down
256	ZNF845	0.469 (1.336)	-0.844 (0.480)	-1.31	0.03	Down
257	PPP2R5C	0.469 (0.888)	-0.845 (0.616)	-1.31	0.01	Down
258	FMR1	0.470 (1.241)	-0.845 (0.407)	-1.32	0.04	Down
259	STYX	0.470 (1.699)	-0.845 (0.410)	-1.32	0.05	Down
260	**PROS1**	0.470 (1.823)	-0.846 (0.350)	-1.32	0.01	Down
261	PRKCA	0.471 (0.780)	-0.847 (0.440)	-1.32	0.00	Down
262	RORA	0.471 (1.144)	-0.849 (0.490)	-1.32	0.02	Down
263	TTC12	0.472 (1.119)	-0.849 (0.506)	-1.32	0.03	Down
264	PGGT1B	0.475 (1.598)	-0.855 (0.580)	-1.33	0.04	Down
265	SRSF10	0.477 (1.326)	-0.858 (0.581)	-1.34	0.02	Down
266	MCM5	0.477 (1.215)	-0.859 (0.369)	-1.34	0.02	Down
267	**FAM186B**	0.478 (1.288)	-0.861 (0.450)	-1.34	0.03	Down
268	MYCBP2	0.479 (1.385)	-0.862 (0.565)	-1.34	0.04	Down
269	CPNE3	0.479 (1.141)	-0.863 (0.499)	-1.34	0.02	Down
270	IARS2	0.480 (1.498)	-0.864 (0.668)	-1.34	0.05	Down
271	ERP44	0.480 (1.525)	-0.865 (0.621)	-1.35	0.04	Down
272	FIBP	0.480 (1.495)	-0.865 (0.426)	-1.35	0.02	Down
273	POTEE	0.481 (1.425)	-0.866 (0.237)	-1.35	0.02	Down
274	RIIAD1	0.481 (1.364)	-0.867 (0.666)	-1.35	0.02	Down
275	RPAP3	0.482 (1.396)	-0.868 (0.549)	-1.35	0.03	Down
276	NEUROD2	0.483 (1.836)	-0.869 (0.665)	-1.35	0.05	Down
277	BTBD3	0.483 (0.901)	-0.870 (0.657)	-1.35	0.03	Down
278	RAB4A	0.484 (1.601)	-0.870 (0.254)	-1.35	0.01	Down
279	ERC1	0.484 (1.394)	-0.871 (0.389)	-1.36	0.04	Down
280	FAM127B	0.485 (2.397)	-0.873 (1.099)	-1.36	0.05	Down

281	GLCE	0.486 (0.983)	-0.874 (0.766)	-1.36	0.02	Down
282	CNOT2	0.486 (1.403)	-0.875 (0.558)	-1.36	0.04	Down
283	MTM1	0.488 (0.817)	-0.878 (0.957)	-1.37	0.02	Down
284	RFPL4AL1	0.488 (1.637)	-0.878 (0.293)	-1.37	0.04	Down
285	BAHD1	0.488 (1.210)	-0.879 (0.342)	-1.37	0.03	Down
286	LEPROT	0.490 (2.156)	-0.882 (0.517)	-1.37	0.05	Down
287	SERPINB8	0.490 (1.103)	-0.882 (0.648)	-1.37	0.05	Down
288	S1PR3	0.491 (1.515)	-0.883 (0.412)	-1.37	0.04	Down
289	HNRNPAB	0.491 (1.743)	-0.884 (0.444)	-1.38	0.04	Down
290	AFG3L2	0.493 (0.775)	-0.887 (0.696)	-1.38	0.01	Down
291	SLC39A9	0.493 (1.218)	-0.887 (0.682)	-1.38	0.04	Down
292	NKIRAS1	0.493 (1.020)	-0.888 (0.534)	-1.38	0.03	Down
293	PIH1D1	0.494 (1.658)	-0.889 (0.305)	-1.38	0.02	Down
294	FAM204A	0.495 (1.226)	-0.891 (0.373)	-1.39	0.04	Down
295	HNMT	0.497 (0.994)	-0.894 (0.698)	-1.39	0.02	Down
296	MYCT1	0.498 (1.424)	-0.896 (0.658)	-1.39	0.03	Down
297	ELK4	0.499 (1.645)	-0.898 (0.464)	-1.40	0.03	Down
298	VEGFB	0.501 (1.673)	-0.902 (0.333)	-1.40	0.03	Down
299	TNFSF4	0.502 (2.106)	-0.903 (0.671)	-1.41	0.05	Down
300	VBP1	0.502 (1.575)	-0.904 (0.476)	-1.41	0.03	Down
301	USO1	0.503 (1.370)	-0.905 (0.721)	-1.41	0.02	Down
302	PGC	0.504 (1.494)	-0.908 (0.443)	-1.41	0.04	Down
303	RPSA	0.505 (1.621)	-0.909 (0.329)	-1.41	0.03	Down
304	SMDT1	0.505 (0.644)	-0.910 (0.618)	-1.42	0.01	Down
305	STK3	0.507 (1.370)	-0.913 (0.354)	-1.42	0.01	Down
306	LRRC8C	0.508 (1.342)	-0.914 (1.408)	-1.42	0.05	Down
307	CEP350	0.508 (1.430)	-0.915 (0.390)	-1.42	0.03	Down
308	CD1D	0.510 (2.193)	-0.917 (0.251)	-1.43	0.03	Down
309	CNOT11	0.510 (1.718)	-0.917 (0.482)	-1.43	0.04	Down
310	TRIT1	0.510 (1.242)	-0.918 (0.479)	-1.43	0.03	Down
311	ATRX	0.511 (1.763)	-0.920 (0.340)	-1.43	0.03	Down
312	DYRK2	0.512 (0.866)	-0.921 (0.520)	-1.43	0.00	Down
313	RFX5	0.512 (1.745)	-0.922 (0.382)	-1.43	0.05	Down
314	C14orf119	0.513 (1.609)	-0.923 (0.522)	-1.44	0.03	Down
315	MMP1	0.513 (1.011)	-0.923 (0.790)	-1.44	0.04	Down
316	RABGGTB	0.514 (1.469)	-0.925 (0.949)	-1.44	0.04	Down
317	HSBP1	0.515 (1.905)	-0.927 (0.405)	-1.44	0.02	Down
318	PDP1	0.515 (1.516)	-0.927 (0.477)	-1.44	0.02	Down
319	CTNND1	0.516 (1.745)	-0.928 (0.285)	-1.44	0.01	Down
320	ELOVL5	0.517 (1.705)	-0.930 (0.467)	-1.45	0.05	Down
321	CNOT4	0.517 (1.303)	-0.931 (0.505)	-1.45	0.03	Down

322	ERCC5	0.517 (1.807)	-0.931 (0.443)	-1.45	0.04	Down
323	SIGMAR1	0.517 (1.642)	-0.931 (0.420)	-1.45	0.01	Down
324	AHR	0.518 (1.533)	-0.933 (0.418)	-1.45	0.04	Down
325	CHPT1	0.519 (0.853)	-0.933 (0.605)	-1.45	0.01	Down
326	NYAP1	0.519 (1.833)	-0.934 (0.431)	-1.45	0.04	Down
327	BRWD3	0.520 (1.536)	-0.936 (0.407)	-1.46	0.03	Down
328	NFKB1	0.520 (1.291)	-0.936 (0.522)	-1.46	0.03	Down
329	COMMD3-BMI1	0.521 (1.006)	-0.937 (0.907)	-1.46	0.02	Down
330	RUFY4	0.522 (2.060)	-0.940 (0.412)	-1.46	0.05	Down
331	GTF2F2	0.525 (2.109)	-0.945 (0.414)	-1.47	0.02	Down
332	ACTR1B	0.525 (2.167)	-0.946 (0.346)	-1.47	0.01	Down
333	TBC1D9	0.526 (1.506)	-0.946 (0.490)	-1.47	0.03	Down
334	ABHD10	0.528 (1.253)	-0.950 (0.596)	-1.48	0.01	Down
335	TCF20	0.529 (1.587)	-0.952 (0.417)	-1.48	0.05	Down
336	KCNMB1	0.530 (1.783)	-0.954 (0.490)	-1.48	0.05	Down
337	RBAK	0.531 (1.496)	-0.955 (0.309)	-1.49	0.02	Down
338	IPMK	0.532 (1.300)	-0.958 (0.734)	-1.49	0.02	Down
339	RCOR1	0.532 (1.684)	-0.958 (0.563)	-1.49	0.05	Down
340	SMARCB1	0.532 (0.970)	-0.958 (0.431)	-1.49	0.00	Down
341	CCT4	0.534 (1.586)	-0.961 (0.375)	-1.50	0.04	Down
342	PBRM1	0.536 (2.035)	-0.964 (0.327)	-1.50	0.05	Down
343	ZNF350	0.536 (1.760)	-0.966 (0.403)	-1.50	0.04	Down
344	FGF21	0.537 (2.778)	-0.967 (0.204)	-1.50	0.02	Down
345	ELK1	0.540 (1.380)	-0.972 (0.680)	-1.51	0.02	Down
346	GPR18	0.541 (2.114)	-0.974 (0.253)	-1.52	0.01	Down
347	TICAM2	0.541 (1.394)	-0.974 (0.918)	-1.52	0.02	Down
348	CTSC	0.543 (1.418)	-0.977 (0.564)	-1.52	0.03	Down
349	ZBTB33	0.543 (1.864)	-0.977 (0.418)	-1.52	0.03	Down
350	TM6SF1	0.543 (1.259)	-0.978 (0.782)	-1.52	0.03	Down
351	KIAA1432	0.545 (0.929)	-0.982 (0.689)	-1.53	0.01	Down
352	SLC35A2	0.546 (1.101)	-0.982 (0.387)	-1.53	0.00	Down
353	MARCKSL1	0.547 (1.092)	-0.985 (0.434)	-1.53	0.01	Down
354	USP3	0.548 (1.873)	-0.986 (0.465)	-1.53	0.03	Down
355	FUT4	0.548 (1.436)	-0.987 (0.854)	-1.54	0.04	Down
356	RFWD2	0.550 (1.026)	-0.989 (0.816)	-1.54	0.01	Down
357	RNF139	0.552 (1.879)	-0.993 (0.616)	-1.55	0.05	Down
358	XIAP	0.552 (1.738)	-0.993 (0.632)	-1.55	0.05	Down
359	CD2AP	0.552 (1.044)	-0.994 (0.767)	-1.55	0.02	Down
360	PRKAA1	0.553 (1.568)	-0.995 (0.361)	-1.55	0.03	Down
361	**KLHL11**	0.553 (1.261)	-0.996 (0.727)	-1.55	0.01	Down
362	TMEM171	0.553 (1.766)	-0.996 (0.544)	-1.55	0.03	Down

363	MPG	0.556 (2.548)	-1.000 (0.577)	-1.56	0.02	Down
364	TIMM23	0.557 (1.615)	-1.003 (0.413)	-1.56	0.03	Down
365	FAM196B	0.558 (1.055)	-1.004 (0.934)	-1.56	0.03	Down
366	AKAP11	0.561 (1.930)	-1.010 (0.435)	-1.57	0.04	Down
367	VPS16	0.561 (1.415)	-1.010 (0.426)	-1.57	0.02	Down
368	PHKB	0.562 (0.559)	-1.012 (0.887)	-1.57	0.01	Down
369	TM2D1	0.563 (1.649)	-1.013 (0.670)	-1.58	0.04	Down
370	ZNF639	0.563 (1.316)	-1.014 (0.405)	-1.58	0.02	Down
371	APC	0.564 (1.207)	-1.016 (0.457)	-1.58	0.01	Down
372	ARL2	0.564 (2.126)	-1.016 (0.306)	-1.58	0.03	Down
373	CERS6	0.567 (1.691)	-1.020 (0.436)	-1.59	0.02	Down
374	ACMSD	0.569 (1.542)	-1.025 (0.542)	-1.59	0.02	Down
375	MSH6	0.571 (1.293)	-1.028 (0.450)	-1.60	0.02	Down
376	POPDC2	0.571 (1.040)	-1.028 (0.441)	-1.60	0.01	Down
377	VPS25	0.571 (1.794)	-1.028 (0.575)	-1.60	0.02	Down
378	COPS2	0.572 (1.353)	-1.030 (0.787)	-1.60	0.02	Down
379	MSR1	0.573 (1.829)	-1.031 (0.284)	-1.60	0.04	Down
380	TMEM167B	0.574 (2.047)	-1.034 (0.307)	-1.61	0.03	Down
381	ARHGEF6	0.575 (1.461)	-1.036 (0.455)	-1.61	0.01	Down
382	ZNF430	0.577 (1.274)	-1.038 (0.563)	-1.62	0.03	Down
383	CALHM2	0.578 (1.371)	-1.041 (0.532)	-1.62	0.01	Down
384	MESDC1	0.581 (1.516)	-1.045 (0.902)	-1.63	0.05	Down
385	SLC9A9	0.581 (1.719)	-1.045 (0.646)	-1.63	0.03	Down
386	TBPL2	0.581 (1.486)	-1.046 (0.411)	-1.63	0.02	Down
387	NIP7	0.583 (1.514)	-1.049 (0.302)	-1.63	0.01	Down
388	REN	0.583 (2.057)	-1.049 (0.264)	-1.63	0.03	Down
389	RMND5A	0.584 (1.644)	-1.052 (0.341)	-1.64	0.02	Down
390	GLRX	0.586 (2.957)	-1.054 (0.223)	-1.64	0.03	Down
391	PTRH1	0.591 (2.187)	-1.063 (0.379)	-1.65	0.02	Down
392	SOX17	0.591 (3.547)	-1.063 (0.227)	-1.65	0.04	Down
393	YTHDF2	0.592 (1.861)	-1.066 (0.393)	-1.66	0.02	Down
394	IFI44L	0.593 (2.452)	-1.067 (0.437)	-1.66	0.03	Down
395	COMMD8	0.594 (1.558)	-1.069 (0.477)	-1.66	0.04	Down
396	NHLRC4	0.598 (2.269)	-1.076 (0.356)	-1.67	0.01	Down
397	SPOPL	0.600 (1.866)	-1.080 (0.523)	-1.68	0.04	Down
398	N4BP2L2	0.604 (1.931)	-1.086 (0.391)	-1.69	0.03	Down
399	TBL1XR1	0.607 (2.141)	-1.093 (0.319)	-1.70	0.02	Down
400	UBAC2	0.608 (1.616)	-1.094 (0.446)	-1.70	0.04	Down
401	ARFIP1	0.610 (1.359)	-1.099 (0.825)	-1.71	0.02	Down
402	KIAA1429	0.610 (1.580)	-1.099 (0.443)	-1.71	0.02	Down
403	HMGN2	0.612 (1.965)	-1.101 (0.351)	-1.71	0.01	Down

404	FAM45A	0.613 (2.119)	-1.103 (0.323)	-1.72	0.02	Down
405	SUB1	0.613 (2.013)	-1.103 (0.437)	-1.72	0.04	Down
406	ADO	0.614 (1.185)	-1.104 (0.578)	-1.72	0.01	Down
407	TNFSF8	0.615 (1.513)	-1.108 (0.708)	-1.72	0.02	Down
408	GPR52	0.616 (1.894)	-1.108 (0.648)	-1.72	0.04	Down
409	RPS12	0.616 (2.551)	-1.108 (0.352)	-1.72	0.05	Down
410	ARL4C	0.617 (2.285)	-1.110 (0.285)	-1.73	0.05	Down
411	PAK1	0.619 (3.072)	-1.114 (0.303)	-1.73	0.03	Down
412	TMEM50B	0.621 (1.705)	-1.118 (0.533)	-1.74	0.04	Down
413	NBN	0.622 (2.173)	-1.119 (0.233)	-1.74	0.04	Down
414	ZFY	0.625 (1.323)	-1.125 (0.440)	-1.75	0.01	Down
415	C12orf75	0.626 (2.122)	-1.127 (0.432)	-1.75	0.02	Down
416	SHOC2	0.629 (1.832)	-1.133 (0.595)	-1.76	0.04	Down
417	NUTF2	0.631 (1.386)	-1.135 (0.086)	-1.77	0.01	Down
418	COIL	0.640 (1.968)	-1.151 (0.258)	-1.79	0.02	Down
419	TMED7-TICAM2	0.640 (1.394)	-1.152 (0.263)	-1.79	0.01	Down
420	ZNF813	0.641 (1.306)	-1.153 (0.933)	-1.79	0.01	Down
421	RBM38	0.642 (2.236)	-1.156 (0.998)	-1.80	0.02	Down
422	ZDHHC2	0.642 (1.002)	-1.156 (0.902)	-1.80	0.00	Down
423	DNASE1L1	0.652 (2.241)	-1.173 (0.594)	-1.83	0.05	Down
424	**TMED7**	0.655 (2.177)	-1.180 (0.371)	-1.84	0.05	Down
425	ZFP91	0.657 (2.149)	-1.182 (0.255)	-1.84	0.03	Down
426	ASCL2	0.661 (1.393)	-1.190 (0.897)	-1.85	0.02	Down
427	THAP11	0.662 (2.419)	-1.191 (0.438)	-1.85	0.03	Down
428	SLC48A1	0.678 (2.097)	-1.221 (0.239)	-1.90	0.01	Down
429	PRKX	0.684 (2.551)	-1.231 (0.190)	-1.92	0.00	Down
430	CFD	0.698 (1.700)	-1.256 (0.637)	-1.95	0.03	Down
431	TAF10	0.728 (3.552)	-1.311 (0.235)	-2.04	0.02	Down
432	ERAP2	0.739 (3.211)	-1.330 (0.342)	-2.07	0.04	Down
433	IFI44	0.742 (3.239)	-1.335 (0.280)	-2.08	0.04	Down
434	VAMP7	0.752 (3.079)	-1.353 (0.265)	-2.11	0.05	Down
435	GNG13	0.761 (3.462)	-1.369 (0.184)	-2.13	0.04	Down
436	GABARAPL2	0.765 (2.890)	-1.378 (0.367)	-2.14	0.05	Down
437	ATP6V1A	0.769 (2.804)	-1.384 (0.376)	-2.15	0.02	Down
438	IGSF6	0.783 (3.772)	-1.410 (0.344)	-2.19	0.05	Down
439	COX14	0.801 (2.824)	-1.441 (0.158)	-2.24	0.02	Down
440	TLR4	0.805 (3.504)	-1.450 (0.164)	-2.26	0.04	Down
441	C3orf38	0.830 (2.541)	-1.494 (0.565)	-2.32	0.02	Down
442	ITGA4	0.851 (3.123)	-1.532 (0.327)	-2.38	0.01	Down
443	C2orf88	0.869 (6.050)	-1.564 (0.210)	-2.43	0.04	Down
444	CLEC1B	0.881 (6.553)	-1.585 (0.208)	-2.47	0.03	Down

445	TNFAIP8L2	0.917 (4.235)	-1.651 (0.179)	-2.57	0.01	Down
446	PIAS1	0.936 (2.982)	-1.684 (0.153)	-2.62	0.03	Down
447	CD300C	0.943 (2.529)	-1.697 (0.313)	-2.64	0.02	Down
448	MMGT1	0.973 (3.289)	-1.752 (0.233)	-2.73	0.01	Down
449	PTGS1	0.577(1.031)	-0.528(0.521)	-3.03	0.05	Down
450	**MIF**	0.588(1.295)	-0.547(0.262)	-3.13	0.03	Down
451	CLEC12A	1.468 (6.261)	-2.642 (0.147)	-4.11	0.02	Down
452	XCR1	0.697(0.988)	-0.743(0.726)	-4.17	0.01	Down
453	CXCL11	0.711 (0.918)	-0.769 (0.845)	-4.35	0.01	Down
454	CCL13	0.795 (1.118)	-0.921 (0.735)	-5.56	0.01	Down

Supplementary table-6: GO Classification and Pathway analysis for the Genes differentially expressed in GR.A:SAHIO

Upregulated genes in PBMC of GR.A:SAHIO vs GR.B:SAHNIO				
**GO Biological process**	**P-value**	**Z-score**	**Combined Score**	**Genes involved**
response to inorganic substance (GO:0010035)	0.00	-2.49	6.04	SYT3;KRT8;CYBRD1;HMOX1;EPX;EGFR;GIP;SOD1
cellular iron ion homeostasis (GO:0006879)	0.00	-2.28	5.52	CYBRD1;HMOX1;HAMP;SOD1
hydrogen peroxide metabolic process (GO:0042743)	0.00	-2.51	6.08	EPX;EGFR;SOD1
reactive oxygen species metabolic process (GO:0072593)	0.00	-2.24	5.44	SH3PXD2B;EPX;EGFR;SOD1
positive regulation of inflammatory response (GO:0050729)	0.00	-2.28	5.53	CCL24;NLRP12;ADORA2B;EGFR
cellular transition metal ion homeostasis (GO:0046916)	0.00	-2.22	5.38	CYBRD1;HMOX1;HAMP;SOD1
iron ion homeostasis (GO:0055072)	0.00	-2.27	5.51	CYBRD1;HMOX1;HAMP;SOD1
positive regulation of cyclin-dependent protein serine/threonine kinase activity involved in G1/S transition of mitotic cell cycle (GO:0031659)	0.00	-2.32	5.61	ADAM17;EGFR
low-density lipoprotein particle clearance (GO:0034383)	0.00	-2.10	5.08	DGAT2;HMOX1
relaxation of smooth muscle (GO:0044557)	0.00	-1.96	4.73	ADORA2B;SOD1
regulation of Ras GTPase activity (GO:0032318)	0.00	-2.37	5.75	CCL24;TBC1D20;ERBB2;ASAP3;ARAP3;CCL26;SOD1
response to wounding (GO:0009611)	0.00	-2.36	5.71	PTK7;ERBB2;HMOX1;SOD1;GIP
positive regulation of chemokine production (GO:0032722)	0.00	-2.24	5.42	ADAM17;ADORA2B;HMOX1
regulation of cyclin-dependent protein serine/threonine kinase activity involved in G1/S transition of mitotic cell cycle (GO:0031657)	0.00	-2.54	6.14	ADAM17;EGFR
regulation of Rho GTPase activity (GO:0032319)	0.00	-2.20	5.32	CCL24;ERBB2;ARAP3;CCL26;SOD1
eosinophil migration (GO:0072677)	0.00	-2.32	5.61	CCL24;EPX
regulation of superoxide anion generation (GO:0032928)	0.00	-2.27	5.50	EGFR;SOD1
positive regulation of smooth muscle cell proliferation (GO:0048661)	0.00	-2.21	5.34	NOTCH3;HMOX1;EGFR
response to transition metal nanoparticle (GO:1990267)	0.00	-2.27	5.49	CYBRD1;HMOX1;EGFR;SOD1
negative regulation of cytokine production (GO:0001818)	0.00	-2.33	5.65	NLRP12;ADAM17;TRAF3IP1;HMOX1;EPX
microtubule-based movement (GO:0007018)	0.00	-2.25	5.46	DNAH3;SNAPIN;TRAF3IP1;ULK4;SOD1
positive regulation of Ras GTPase activity (GO:0032320)	0.00	-2.32	5.22	CCL24;TBC1D20;ERBB2;ASAP3;ARAP3;CCL26
positive regulation of response to external stimulus (GO:0032103)	0.00	-2.34	5.24	CCL24;ADAM17;NLRP12;ADORA2B;EGFR
transition metal ion homeostasis (GO:0055076)	0.00	-2.20	4.92	CYBRD1;HMOX1;HAMP;SOD1
regulation of chemokine production (GO:0032642)	0.00	-2.04	4.57	ADAM17;ADORA2B;HMOX1
negative regulation of smooth muscle contraction (GO:0045986)	0.00	-2.50	5.60	ADORA2B;SOD1
positive regulation of response to wounding	0.00	-2.21	4.67	CCL24;NLRP12;ADORA2B;EGFR

(GO:1903036)				
regulation of superoxide metabolic process (GO:0090322)	0.00	-2.42	5.11	EGFR;SOD1
positive regulation of intracellular transport (GO:0032388)	0.00	-2.40	5.06	NLRP12;SNAPIN;TBC1D20;ADORA2B;EGFR
positive regulation of cytokine production (GO:0001819)	0.00	-2.38	4.77	ADAM17;NLRP12;ADORA2B;HMOX1;EPX;SOD1
regulation of mast cell degranulation (GO:0043304)	0.00	-2.59	5.12	ADORA2B;HMOX1
myeloid cell homeostasis (GO:0002262)	0.00	-2.64	5.23	HMOX1;SOD1
regulation of mast cell activation involved in immune response (GO:0033006)	0.00	-2.54	5.03	ADORA2B;HMOX1
positive regulation of cyclin-dependent protein serine/threonine kinase activity (GO:0045737)	0.00	-2.31	4.57	ADAM17;EGFR
positive regulation of epithelial cell proliferation (GO:0050679)	0.00	-2.29	4.54	CCL24;ERBB2;EGFR;CCL26
negative regulation of muscle contraction (GO:0045932)	0.00	-2.55	5.06	ADORA2B;SOD1
plasma lipoprotein particle clearance (GO:0034381)	0.00	-2.43	4.82	DGAT2;HMOX1
relaxation of muscle (GO:0090075)	0.00	-2.27	4.50	ADORA2B;SOD1
positive regulation of Rho GTPase activity (GO:0032321)	0.00	-2.12	4.13	CCL24;ERBB2;ARAP3;CCL26
regulation of inflammatory response (GO:0050727)	0.00	-2.33	4.55	CCL24;NLRP12;ADORA2B;KRT1;EGFR
wound healing (GO:0042060)	0.00	-2.16	4.21	PTK7;ERBB2;HMOX1
Notch receptor processing (GO:0007220)	0.00	-2.43	4.73	NOTCH3;ADAM17
regulation of T cell differentiation in thymus (GO:0033081)	0.00	-2.40	4.69	ERBB2;SOD1
cell chemotaxis (GO:0060326)	0.00	-2.15	4.19	CCL24;CXCL2;CCL26;CCL15
regulation of smooth muscle cell proliferation (GO:0048660)	0.01	-2.05	4.00	NOTCH3;HMOX1;EGFR
regulation of cytokine production (GO:0001817)	0.01	-2.39	4.67	NLRP12;ADAM17;ADORA2B;TRAF3IP1;HMOX1;EPX;SOD1
response to metal ion (GO:0010038)	0.01	-2.33	4.48	SYT3;CYBRD1;HMOX1;EGFR;SOD1
positive regulation of protein serine/threonine kinase activity (GO:0071902)	0.01	-2.25	4.30	ADAM17;ADORA2B;ERBB2;EGFR;SOD1
purinergic receptor signaling pathway (GO:0035587)	0.01	-2.60	4.95	ADORA2B;C12ORF76
anterograde axon cargo transport (GO:0008089)	0.01	-2.46	4.69	SNAPIN;SOD1
regulation of leukocyte degranulation (GO:0043300)	0.01	-2.44	4.66	ADORA2B;HMOX1
regulation of myeloid leukocyte mediated immunity (GO:0002886)	0.01	-2.35	4.48	ADORA2B;HMOX1
response to iron ion (GO:0010039)	0.01	-2.17	4.14	HMOX1;CYBRD1
positive regulation of vasodilation (GO:0045909)	0.01	-2.66	4.93	HMOX1;EGFR
regulation of mast cell activation (GO:0033003)	0.01	-2.27	4.12	ADORA2B;HMOX1
regulation of MAP kinase activity (GO:0043405)	0.01	-2.26	4.10	ADORA2B;ERBB2;EGFR;SOD1;DUSP14
response to acid chemical (GO:0001101)	0.01	-2.35	4.27	DGAT2;PTK7;KRT8;EGFR;GIP
acylglycerol homeostasis (GO:0055090)	0.01	-2.26	4.09	DGAT2;GIP
triglyceride homeostasis (GO:0070328)	0.01	-2.25	4.08	DGAT2;GIP
regulation of intracellular transport (GO:0032386)	0.01	-2.42	4.38	NLRP12;SNAPIN;TBC1D20;ADORA2B;HMOX1;EGFR
regulation of angiogenesis (GO:0045765)	0.01	-2.15	3.77	CCL24;ERBB2;KRT1;HMOX1

regulation of regulated secretory pathway (GO:1903305)	0.01	-2.32	4.05	ADORA2B;HMOX1
adipose tissue development (GO:0060612)	0.01	-2.15	3.76	DGAT2;SH3PXD2B
microtubule-based transport (GO:0010970)	0.01	-2.10	3.65	SNAPIN;TRAF3IP1;SOD1
response to hydrogen peroxide (GO:0042542)	0.01	-2.07	3.60	HMOX1;EPX;SOD1
response to oxidative stress (GO:0006979)	0.01	-2.22	3.87	KRT1;HMOX1;EPX;EGFR;SOD1
superoxide metabolic process (GO:0006801)	0.01	-2.12	3.58	SH3PXD2B;SOD1
regulation of protein serine/threonine kinase activity (GO:0071900)	0.01	-2.23	3.77	ADAM17;ADORA2B;ERBB2;EGFR;SOD1;DUSP14
metal ion homeostasis (GO:0055065)	0.01	-2.26	3.70	HMOX1;CYBRD1;HAMP;EGFR;CCL15;SOD1
regulation of ARF GTPase activity (GO:0032312)	0.01	-2.20	3.59	ASAP3;ARAP3
sensory organ development (GO:0007423)	0.01	-2.10	3.42	TRAF3IP1;SH3PXD2B;EGFR
regulation of vasculature development (GO:1901342)	0.01	-2.14	3.48	CCL24;KRT1;ERBB2;HMOX1
positive regulation of MAP kinase activity (GO:0043406)	0.01	-2.10	3.43	ADORA2B;ERBB2;EGFR;SOD1
cytoskeleton-dependent intracellular transport (GO:0030705)	0.01	-2.06	3.35	SNAPIN;TRAF3IP1;SOD1
response to extracellular stimulus (GO:0009991)	0.01	-2.27	3.54	ADORA2B;HMOX1;EGFR;GIP;SOD1
regulation of leukocyte migration (GO:0002685)	0.01	-2.12	3.22	CCL24;ADAM17;HMOX1
positive regulation of reactive oxygen species metabolic process (GO:2000379)	0.01	-2.07	3.14	EGFR;SOD1
regulation of vasodilation (GO:0042312)	0.01	-2.23	3.37	HMOX1;EGFR
retina homeostasis (GO:0001895)	0.01	-2.12	3.20	KRT1;SOD1
response to axon injury (GO:0048678)	0.01	-2.12	3.20	GIP;SOD1
cellular chemical homeostasis (GO:0055082)	0.01	-2.25	3.35	DGAT2;CYBRD1;HMOX1;HAMP;CCL15;SOD1
axon cargo transport (GO:0008088)	0.01	-2.15	3.20	SNAPIN;SOD1
negative regulation of DNA binding (GO:0043392)	0.02	-2.13	3.01	HMOX1;ZFP90
cation homeostasis (GO:0055080)	0.02	-2.21	3.09	HMOX1;CYBRD1;HAMP;EGFR;CCL15;SOD1
negative regulation of defense response (GO:0031348)	0.02	-2.04	2.84	NLRP12;TRAF3IP1;KRT1
lipoprotein metabolic process (GO:0042157)	0.02	-1.99	2.74	APOL5;NPC1L1
connective tissue development (GO:0061448)	0.02	-1.95	2.68	DGAT2;SH3PXD2B
excretion (GO:0007588)	0.02	-2.00	2.69	ADORA2B;HMOX1
regulation of response to wounding (GO:1903034)	0.02	-2.26	3.02	CCL24;NLRP12;ADORA2B;KRT1;EGFR
positive regulation of Rac GTPase activity (GO:0032855)	0.02	-1.91	2.54	CCL24;CCL26
positive regulation of GTPase activity (GO:0043547)	0.02	-2.18	2.89	CCL24;TBC1D20;ERBB2;ASAP3;ARAP3;CCL26
positive regulation of GTP catabolic process (GO:0033126)	0.02	-2.18	2.89	CCL24;TBC1D20;ERBB2;ASAP3;ARAP3;CCL26
exocytosis (GO:0006887)	0.02	-2.06	2.71	SYT3;SNAPIN;SYTL3;SOD1
response to drug (GO:0042493)	0.02	-2.22	2.92	ADAM17;NPC1L1;EGFR;GIP;SOD1
muscle system process (GO:0003012)	0.02	-2.09	2.66	GJC1;ADORA2B;HMOX1;SOD1
cellular metal ion homeostasis (GO:0006875)	0.02	-2.22	2.79	CYBRD1;HMOX1;HAMP;CCL15;SOD1
negative regulation of secretion by cell (GO:1903531)	0.02	-2.01	2.49	PSMD9;NLRP12;HMOX1
regulation of smooth muscle contraction	0.02	-1.87	2.31	ADORA2B;SOD1

(GO:0006940)				
positive regulation of insulin secretion (GO:0032024)	0.02	-1.80	2.23	PSMD9;GIP
positive regulation of secretion by cell (GO:1903532)	0.02	-2.14	2.56	PSMD9;NLRP12;ADORA2B;GIP
response to reactive oxygen species (GO:0000302)	0.02	-2.04	2.36	HMOX1;EPX;SOD1
granulocyte migration (GO:0097530)	0.03	-1.82	2.11	CCL24;EPX
regulation of epithelial cell proliferation (GO:0050678)	0.03	-2.20	2.55	CCL24;ERBB2;EGFR;CCL26
positive regulation of actin filament polymerization (GO:0030838)	0.03	-1.95	2.26	CCL24;CCL26
cellular response to acid chemical (GO:0071229)	0.03	-2.09	2.42	DGAT2;PTK7;EGFR
cellular cation homeostasis (GO:0030003)	0.03	-2.19	2.53	CYBRD1;HMOX1;HAMP;CCL15;SOD1
cellular response to oxidative stress (GO:0034599)	0.03	-2.02	2.35	HMOX1;EPX;SOD1
taxis (GO:0042330)	0.03	-2.13	2.47	CCL24;CXCL2;CCL26;CCL15
chemotaxis (GO:0006935)	0.03	-2.13	2.46	CCL24;CXCL2;CCL26;CCL15
negative regulation of secretion (GO:0051048)	0.03	-2.03	2.36	PSMD9;NLRP12;HMOX1
peptidyl-tyrosine phosphorylation (GO:0018108)	0.03	-1.96	2.27	PTK7;ERBB2;EGFR
peptidyl-tyrosine modification (GO:0018212)	0.03	-1.96	2.27	PTK7;ERBB2;EGFR
cellular ion homeostasis (GO:0006873)	0.03	-2.18	2.52	CYBRD1;HMOX1;HAMP;CCL15;SOD1
positive regulation of defense response (GO:0031349)	0.03	-2.15	2.49	CCL24;NLRP12;ADORA2B;EGFR
positive regulation of secretion (GO:0051047)	0.03	-2.13	2.47	PSMD9;NLRP12;ADORA2B;GIP
positive regulation of endothelial cell proliferation (GO:0001938)	0.03	-1.88	2.18	CCL24;CCL26
positive regulation of cell cycle (GO:0045787)	0.03	-2.22	2.57	PSMD9;ADAM17;UBE2C;EGFR
positive regulation of peptide hormone secretion (GO:0090277)	0.03	-1.79	2.07	PSMD9;GIP
regulation of ERK1 and ERK2 cascade (GO:0070372)	0.03	-2.10	2.43	NLRP12;ERBB2;EGFR
positive regulation of cell migration (GO:0030335)	0.03	-2.18	2.52	CCL24;ADAM17;EGFR;CCL26
positive regulation of translation (GO:0045727)	0.03	-1.91	2.21	ERBB2;WIBG
positive regulation of peptide secretion (GO:0002793)	0.04	-1.78	2.06	PSMD9;GIP
negative regulation of ubiquitin-protein ligase activity involved in mitotic cell cycle (GO:0051436)	0.04	-1.78	2.06	PSMD9;UBE2C
organic hydroxy compound biosynthetic process (GO:1901617)	0.04	-1.98	2.29	CHKA;NPC1L1;HDC
positive regulation of cell motility (GO:2000147)	0.04	-2.16	2.51	CCL24;ADAM17;EGFR;CCL26
positive regulation of protein polymerization (GO:0032273)	0.04	-1.93	2.23	CCL24;CCL26
response to nutrient levels (GO:0031667)	0.04	-2.11	2.44	HMOX1;EGFR;GIP;SOD1
regulation of epidermal growth factor receptor signaling pathway (GO:0042058)	0.04	-1.84	2.13	ADAM17;EGFR
positive regulation of cellular component movement (GO:0051272)	0.04	-2.14	2.48	CCL24;ADAM17;EGFR;CCL26
positive regulation of blood circulation (GO:1903524)	0.04	-1.88	2.17	HMOX1;EGFR
positive regulation of ubiquitin-protein ligase activity involved in mitotic cell cycle	0.04	-1.74	2.01	PSMD9;UBE2C

(GO:0051437)				
microtubule-based process (GO:0007017)	0.04	-2.08	2.41	DNAH3;SNAPIN;TRAF3IP1;ULK4;SOD1
regulation of ERBB signaling pathway (GO:1901184)	0.04	-1.77	2.05	ADAM17;EGFR
negative regulation of ligase activity (GO:0051352)	0.04	-1.70	1.97	PSMD9;UBE2C
negative regulation of ubiquitin-protein transferase activity (GO:0051444)	0.04	-1.70	1.97	PSMD9;UBE2C
positive regulation of locomotion (GO:0040017)	0.04	-2.06	2.38	CCL24;ADAM17;EGFR;CCL26
myeloid leukocyte migration (GO:0097529)	0.04	-1.72	1.99	CCL24;EPX
regulation of vesicle-mediated transport (GO:0060627)	0.05	-1.99	2.30	SYT3;TBC1D20;ADORA2B;HMOX1
regulation of DNA binding (GO:0051101)	0.05	-1.72	1.99	HMOX1;ZFP90
positive regulation of leukocyte migration (GO:0002687)	0.05	-1.71	1.98	CCL24;ADAM17
ovulation cycle process (GO:0022602)	0.05	-1.68	1.94	GPR149;SOD1
regulation of ubiquitin-protein ligase activity involved in mitotic cell cycle (GO:0051439)	0.05	-1.61	1.86	PSMD9;UBE2C
cellular response to external stimulus (GO:0071496)	0.05	-1.87	2.17	ADORA2B;HMOX1;EGFR
eye development (GO:0001654)	0.05	-1.70	1.97	TRAF3IP1;SH3PXD2B
sulfur compound metabolic process (GO:0006790)	0.05	-1.86	2.16	DGAT2;ARSG;EGFR;SOD1
positive regulation of protein kinase activity (GO:0045860)	0.05	-1.97	2.29	ADAM17;ADORA2B;ERBB2;EGFR;SOD1
positive regulation of protein import into nucleus (GO:0042307)	0.05	-1.69	1.95	NLRP12;EGFR
B cell differentiation (GO:0030183)	0.05	-1.62	1.87	CD79A;ADAM17
**GO Cellular process**	**P-value**	**Z-score**	**Combined Score**	**Genes involved**
extracellular space (GO:0005615)	0.00	-2.21	2.37	CCL24;KRT1;CPAMD8;HMOX1;ARSG;CXCL2;PRKAG3;EGFR;GIP;CCL26;SOD1;CCL15
membrane raft (GO:0045121)	0.01	-2.29	2.46	CD79A;ADAM17;HMOX1;EGFR
brush border membrane (GO:0031526)	0.02	-2.19	2.35	NPC1L1;CYBRD1
receptor complex (GO:0043235)	0.03	-2.31	2.48	CD79A;NOTCH3;ERBB2;EGFR
ruffle (GO:0001726)	0.04	-2.17	2.33	ASAP3;ARAP3
immunoglobulin complex (GO:0019814)	0.04	-1.01	1.09	CD79A
endosome membrane (GO:0010008)	0.04	-2.12	2.28	ERBB2;VPS45;EGFR
AMP-activated protein kinase complex (GO:0031588)	0.04	-1.17	1.26	PRKAG3
endoplasmic reticulum membrane (GO:0005789)	0.04	-2.14	2.30	GJC1;NOTCH3;DGAT2;TBC1D20;HMOX1
**GO Molecular process**	**P-value**	**Z-score**	**Combined Score**	**Genes involved**
chemokine activity (GO:0008009)	0.00	-2.44	9.68	CCL24;CXCL2;CCL26;CCL15
chemokine receptor binding (GO:0042379)	0.00	-2.26	8.96	CCL24;CXCL2;CCL15;CCL26
receptor signaling protein tyrosine kinase activity (GO:0004716)	0.00	-2.42	6.24	ERBB2;EGFR
purinergic receptor activity (GO:0035586)	0.01	-2.87	4.67	ADORA2B;C12ORF76
cytokine receptor binding (GO:0005126)	0.00	-2.40	3.99	CCL24;ADAM17;CXCL2;CCL15;CCL26
transmembrane receptor protein tyrosine	0.00	-2.15	3.56	PTK7;ERBB2;EGFR

kinase activity (GO:0004714)				
transmembrane receptor protein kinase activity (GO:0019199)	0.01	-2.18	3.55	PTK7;ERBB2;EGFR
Rab GTPase binding (GO:0017137)	0.01	-2.17	3.53	TBC1D20;NPC1L1;SYTL3
ARF GTPase activator activity (GO:0008060)	0.01	-2.38	3.52	ASAP3;ARAP3
calcium-dependent phospholipid binding (GO:0005544)	0.01	-2.33	3.43	SYT3;SYTL3
Ras GTPase binding (GO:0017016)	0.01	-2.28	3.36	TBC1D20;NPC1L1;SYTL3;SOD1
small GTPase binding (GO:0031267)	0.02	-2.29	3.30	TBC1D20;NPC1L1;SYTL3;SOD1
cytokine activity (GO:0005125)	0.02	-2.27	3.28	CCL24;CXCL2;CCL26;CCL15
aspartic-type endopeptidase activity (GO:0004190)	0.02	-2.24	3.27	NRIP2;PGA4
protein phosphatase binding (GO:0019903)	0.01	-2.17	3.21	ERBB2;EGFR;SOD1
aspartic-type peptidase activity (GO:0070001)	0.02	-2.19	3.19	NRIP2;PGA4
GTPase binding (GO:0051020)	0.02	-2.28	3.16	TBC1D20;NPC1L1;SYTL3;SOD1
G-protein coupled receptor binding (GO:0001664)	0.02	-2.19	2.91	CCL24;CXCL2;CCL26;CCL15
protein tyrosine kinase activity (GO:0004713)	0.03	-2.10	2.61	PTK7;ERBB2;EGFR
phosphatase binding (GO:0019902)	0.03	-2.01	2.50	ERBB2;EGFR;SOD1
phosphatidylinositol bisphosphate binding (GO:1902936)	0.03	-1.99	2.46	SH3PXD2B;ARAP3
SNARE binding (GO:0000149)	0.03	-1.97	2.39	SYT3;SNAPIN
ribonucleoprotein complex binding (GO:0043021)	0.04	-1.94	2.09	ERAL1;WIBG
antioxidant activity (GO:0016209)	0.05	-1.93	2.08	EPX;SOD1
**KEGG pathway**	**P-value**	**Z-score**	**Combined Score**	**Genes involved**
Cytokine-cytokine receptor interaction_Homo sapiens_hsa04060	0.01	-1.88	3.22	CCL24;CXCL2;EGFR;CCL26;CCL15
Dorso-ventral axis formation_Homo sapiens_hsa04320	0.01	-1.56	2.68	NOTCH3;EGFR
Chemokine signaling pathway_Homo sapiens_hsa04062	0.01	-1.88	3.22	CCL24;CXCL2;CCL26;CCL15
HIF-1 signaling pathway_Homo sapiens_hsa04066	0.01	-1.76	3.02	ERBB2;HMOX1;EGFR
Bladder cancer_Homo sapiens_hsa05219	0.01	-1.71	2.94	ERBB2;EGFR
Fat digestion and absorption_Homo sapiens_hsa04975	0.01	-1.60	2.74	DGAT2;NPC1L1
Notch signaling pathway_Homo sapiens_hsa04330	0.02	-1.50	2.57	NOTCH3;ADAM17
Mineral absorption_Homo sapiens_hsa04978	0.02	-1.49	2.56	CYBRD1;HMOX1
Endometrial cancer_Homo sapiens_hsa05213	0.02	-1.66	2.85	ERBB2;EGFR
Endocytosis_Homo sapiens_hsa04144	0.02	-1.74	2.99	ASAP3;ARAP3;VPS45;EGFR
Non-small cell lung cancer_Homo sapiens_hsa05223	0.03	-1.69	2.89	ERBB2;EGFR
Longevity regulating pathway - multiple species_Homo sapiens_hsa04213	0.03	-1.60	2.71	PRKAG3;SOD1
Pancreatic cancer_Homo sapiens_hsa05212	0.03	-1.57	2.65	ERBB2;EGFR
Central carbon metabolism in cancer_Homo sapiens_hsa05230	0.03	-1.44	2.44	ERBB2;EGFR
Epithelial cell signaling in Helicobacter pylori infection_Homo sapiens_hsa05120	0.04	-1.44	2.44	ADAM17;EGFR

MicroRNAs in cancer_Homo sapiens_hsa05206	0.04	-1.55	2.62	NOTCH3;ERBB2;HMOX1;EGFR
Adherens junction_Homo sapiens_hsa04520	0.04	-1.33	2.26	ERBB2;EGFR
Calcium signaling pathway_Homo sapiens_hsa04020	0.04	-1.53	2.59	ADORA2B;ERBB2;EGFR
**Reactome pathway**	**P-value**	**Z-score**	**Combined Score**	**Genes involved**
ERBB2 Activates PTK6 Signaling_Homo sapiens_R-HSA-8847993	0.00	-2.21	3.79	ERBB2;EGFR
Receptor-ligand binding initiates the second proteolytic cleavage of Notch receptor_Homo sapiens_R-HSA-156988	0.00	-1.96	3.37	NOTCH3;ADAM17
ERBB2 Regulates Cell Motility_Homo sapiens_R-HSA-6785631	0.00	-2.19	3.76	ERBB2;EGFR
PI3K events in ERBB2 signaling_Homo sapiens_R-HSA-1963642	0.00	-2.07	3.56	ERBB2;EGFR
GRB2 events in ERBB2 signaling_Homo sapiens_R-HSA-1963640	0.00	-1.94	3.34	ERBB2;EGFR
SHC1 events in ERBB2 signaling_Homo sapiens_R-HSA-1250196	0.00	-2.02	3.42	ERBB2;EGFR
Surfactant metabolism_Homo sapiens_R-HSA-5683826	0.01	-1.96	2.41	ADORA2B;PGA4
Histidine, lysine, phenylalanine, tyrosine, proline and tryptophan catabolism_Homo sapiens_R-HSA-6788656	0.01	-1.82	2.24	HDC;ASRGL1
Iron uptake and transport_Homo sapiens_R-HSA-917937	0.02	-2.00	2.45	CYBRD1;HMOX1
Signaling by ERBB2_Homo sapiens_R-HSA-1227986	0.02	-1.98	2.43	ERBB2;EGFR
Vesicle-mediated transport_Homo sapiens_R-HSA-5653656	0.02	-2.08	2.56	GJC1;CD163;SNAPIN;TBC1D20;VPS45;PRKAG3
Signaling by the B Cell Receptor (BCR)_Homo sapiens_R-HSA-983705	0.02	-2.38	2.93	PSMD9;CD79A;ERBB2;EGFR
Constitutive Signaling by Aberrant PI3K in Cancer_Homo sapiens_R-HSA-2219530	0.03	-1.83	2.25	ERBB2;EGFR
Autodegradation of Cdh1 by Cdh1:APC/C_Homo sapiens_R-HSA-174084	0.03	-2.04	2.51	PSMD9;UBE2C
Hedgehog ligand biogenesis_Homo sapiens_R-HSA-5358346	0.03	-2.20	2.70	PSMD9;ADAM17
Membrane Trafficking_Homo sapiens_R-HSA-199991	0.03	-1.99	2.44	GJC1;SNAPIN;TBC1D20;VPS45;PRKAG3
IRS-mediated signalling_Homo sapiens_R-HSA-112399	0.03	-2.36	2.90	PSMD9;ERBB2;PRKAG3;EGFR
APC/C:Cdc20 mediated degradation of Securin_Homo sapiens_R-HSA-174154	0.03	-2.03	2.49	PSMD9;UBE2C
Insulin receptor signalling cascade_Homo sapiens_R-HSA-74751	0.03	-2.34	2.87	PSMD9;ERBB2;PRKAG3;EGFR
Signaling by Type 1 Insulin-like Growth Factor 1 Receptor (IGF1R)_Homo sapiens_R-HSA-2404192	0.03	-2.30	2.83	PSMD9;ERBB2;PRKAG3;EGFR
IGF1R signaling cascade_Homo sapiens_R-HSA-2428924	0.03	-2.30	2.82	PSMD9;ERBB2;PRKAG3;EGFR
IRS-related events triggered by IGF1R_Homo sapiens_R-HSA-2428928	0.03	-2.29	2.81	PSMD9;ERBB2;PRKAG3;EGFR
Diseases of signal transduction_Homo sapiens_R-HSA-5663202	0.03	-2.15	2.63	PSMD9;ADAM17;ERBB2;EGFR
Signaling by PTK6_Homo sapiens_R-HSA-8848021	0.03	-1.71	2.10	ERBB2;EGFR
Macroautophagy_Homo sapiens_R-HSA-1632852	0.03	-1.59	1.96	WDR45B;PRKAG3
APC/C:Cdh1 mediated degradation of Cdc20 and other APC/C:Cdh1 targeted proteins in late	0.04	-1.91	2.34	PSMD9;UBE2C

mitosis/early G1_Homo sapiens_R-HSA-174178				
Cdc20:Phospho-APC/C mediated degradation of Cyclin A_Homo sapiens_R-HSA-174184	0.04	-1.88	2.31	PSMD9;UBE2C
APC:Cdc20 mediated degradation of cell cycle proteins prior to satisfation of the cell cycle checkpoint_Homo sapiens_R-HSA-179419	0.04	-1.88	2.31	PSMD9;UBE2C
APC/C:Cdc20 mediated degradation of mitotic proteins_Homo sapiens_R-HSA-176409	0.04	-1.88	2.31	PSMD9;UBE2C
Activation of APC/C and APC/C:Cdc20 mediated degradation of mitotic proteins_Homo sapiens_R-HSA-176814	0.04	-1.82	2.23	PSMD9;UBE2C
Signaling by Insulin receptor_Homo sapiens_R-HSA-74752	0.04	-2.15	2.64	PSMD9;ERBB2;PRKAG3;EGFR
Regulation of APC/C activators between G1/S and early anaphase_Homo sapiens_R-HSA-176408	0.05	-1.80	2.21	PSMD9;UBE2C
Downstream signaling events of B Cell Receptor (BCR)_Homo sapiens_R-HSA-1168372	0.05	-2.06	2.53	PSMD9;ERBB2;EGFR
PI5P, PP2A and IER3 Regulate PI3K/AKT Signaling_Homo sapiens_R-HSA-6811558	0.05	-1.63	2.01	ERBB2;EGFR

Downregulated genes in PBMC of GR.A:SAHIO vs. GR.B:SAHNIO

GO Biological process	P-value	Z-score	Combined Score	Genes involved
nuclear-transcribed mRNA poly(A) tail shortening (GO:0000289)	0.00	-2.71	1.54	CNOT4;CNOT6;CNOT11;CNOT2;MLH1
positive regulation of myeloid leukocyte cytokine production involved in immune response (GO:0061081)	0.00	-2.79	0.88	MIF;BCL10;TLR4
negative regulation of interleukin-17 production (GO:0032700)	0.00	-2.44	0.88	TUSC2;TNFSF4;TLR4
chromatin modification (GO:0016568)	0.00	-2.40	0.86	SUZ12;KDM5A;PBRM1;PRKAA1;SMARCB1;CHD9;TAF10;USP3;ATRX;PRKCA;ELK4;HIRA;KANSL1;KAT5;TBL1XR1;BAHD1;FAM175A;RCOR1;CSRP2BP;WHSC1L1
induction of positive chemotaxis (GO:0050930)	0.00	-2.65	0.84	VEGFB;VEGFC;PRKCA
positive regulation of cytokine production involved in immune response (GO:0002720)	0.00	-2.65	0.84	TNFSF4;MIF;BCL10;TLR4
signal transduction by p53 class mediator (GO:0072331)	0.00	-2.31	0.83	RBM38;DYRK2;KAT5;RFWD2;ATRX;MDM4;MIF;PPP2R5C;NBN
isotype switching (GO:0045190)	0.01	-2.62	0.83	MSH6;NBN;MLH1
somatic recombination of immunoglobulin genes involved in immune response (GO:0002204)	0.01	-2.61	0.82	MSH6;NBN;MLH1
DNA damage response, signal transduction by p53 class mediator (GO:0030330)	0.00	-2.28	0.82	RBM38;KAT5;RFWD2;ATRX;MDM4;MIF;NBN;PPP2R5C
B cell activation involved in immune response (GO:0002312)	0.00	-2.61	0.82	MSH6;NBN;MLH1;TLR4
somatic diversification of immunoglobulins involved in immune response (GO:0002208)	0.01	-2.60	0.82	MSH6;NBN;MLH1
signal transduction in response to DNA damage (GO:0042770)	0.00	-2.27	0.82	RBM38;KAT5;RFWD2;ATRX;MDM4;MIF;PPP2R5C;NBN
somatic recombination of immunoglobulin gene segments (GO:0016447)	0.01	-2.54	0.80	MSH6;NBN;MLH1
regulation of interleukin-17 production (GO:0032660)	0.01	-2.53	0.80	TUSC2;TNFSF4;TLR4
vesicle fusion (GO:0006906)	0.01	-2.48	0.78	EEA1;VAMP7;USO1

positive regulation of positive chemotaxis (GO:0050927)	0.02	-2.48	0.78	VEGFB;VEGFC;PRKCA
somatic diversification of immunoglobulins (GO:0016445)	0.02	-2.43	0.77	MSH6;NBN;MLH1
modification by host of symbiont morphology or physiology (GO:0051851)	0.03	-2.42	0.76	SMARCB1;ZNF639;TUSC2
negative regulation of lymphocyte apoptotic process (GO:0070229)	0.02	-2.41	0.76	SLC46A2;MIF;BCL10
regulation of positive chemotaxis (GO:0050926)	0.02	-2.40	0.76	VEGFB;VEGFC;PRKCA
positive regulation of interleukin-10 production (GO:0032733)	0.01	-2.39	0.75	TUSC2;TNFSF4;TLR4
T cell selection (GO:0045058)	0.02	-2.37	0.75	FAS;CD1D;CD3E
mismatch repair (GO:0006298)	0.02	-2.34	0.74	MSH6;MLH1;MUTYH
apoptotic signaling pathway (GO:0097190)	0.01	-2.33	0.74	DYRK2;TRIO;TM2D1;XIAP;PRKCA;PPP2R5C;CD3E;MLH1;MSH6;FAS;TNFRSF25;NBN;ARHGEF6
**GO Cellular process**	**P-value**	**Z-score**	**Combined Score**	**Genes involved**
CCR4-NOT complex (GO:0030014)	0.00	-2.81	8.06	CNOT4;CNOT6;CNOT11;CNOT2
Golgi apparatus (GO:0005794)	0.00	-2.31	6.63	SLC35B2;LEPROT;MCFD2;TMEM167B;SAR1B;USO1;HTT;ZDHHC2;FUT4;ARHGAP21;TRAPPC11;ERC1;RAB6A;TMED7;EVI5;ARSB;CTSC;SLC30A7;SLC35A2;GALNT6;GABARAPL2;TICAM2;B3GAT3;MMGT1;GLCE;AGTRAP;MSH6;NSFL1C;VAMP7;AKAP9;C6ORF89;GOPC
nucleoplasm (GO:0005654)	0.00	-2.24	6.43	FYTTD1;SMARCB1;CHD9;MPG;SP140;FMR1;RORA;NMD3;HNRNPR;COIL;HIRA;KAT5;DGCR8;NBN;SRSF10;RNF111;MUTYH;SUZ12;TAF10;IPMK;GTF3A;MED8;PRKCA;GTF2F2;MED26;NFKB1;PIAS1;RAD52;KANSL1;RFWD2;TBL1XR1;POLR3E;SNRPA1;ERCC5;MCM5;RCOR1;HIST1H2BC
nucleolus (GO:0005730)	0.00	-2.16	5.91	SMARCB1;DYRK2;FMR1;TCF20;HNRNPR;AHR;HNMT;ELK1;ELK4;COIL;KAT5;DGCR8;NBN;RNF111;ZNF641;RBM17;MRPS26;IPMK;DDX56;ATRX;ZBTB33;ILF2;ZHX1;MSH6;TMX1;SUB1;AAGAB;SNRPA1;ZNF436;MCM5;VPS25;FYTTD1;ZNF350;USO1;NOL8;NMD3;MIER1;SHOC2;CCT4;API5;CEP350;MLH1;NFKB1;PBX1;THAP11;NSFL1C;RFWD2;POLR3E;ERCC5;NF2;RCOR1
Golgi membrane (GO:0000139)	0.00	-2.21	5.20	SLC30A7;SLC35A2;GALNT6;SLC35B2;GABARAPL2;B3GAT3;SAR1B;PROS1;PITPNB;USO1;MAN2A1;RFWD2;MGAT5;ARFIP1;CHPT1
integral component of mitochondrial inner membrane (GO:0031305)	0.00	-2.33	3.68	SMDT1;COX18;TIMM23
protein kinase complex (GO:1902911)	0.01	-2.06	2.87	KDM5A;PRKAA1;PHKG2;ERCC5;PHKB;ERC1
serine/threonine protein kinase complex (GO:1902554)	0.01	-1.95	2.72	PRKAA1;PHKG2;ERCC5;PHKB;ERC1
transcriptional repressor complex (GO:0017053)	0.01	-1.95	1.47	ZNF350;TBL1XR1;MIER1;N4BP2L2;RCOR1
platelet alpha granule lumen (GO:0031093)	0.02	-1.91	1.31	CFD;PROS1;VEGFB;VEGFC
**GO Molecular process**	**P-value**	**Z-score**	**Combined Score**	**Genes involved**
vascular endothelial growth factor receptor binding (GO:0005172)	0.00	-2.72	2.32	VEGFB;VEGFC;CD2AP
cadherin binding (GO:0045296)	0.00	-2.73	2.16	FER;APC;CTNND1;CD2AP
mismatch repair complex binding (GO:0032404)	0.00	-2.34	1.99	MSH6;MLH1;MUTYH
chemoattractant activity (GO:0042056)	0.01	-2.80	1.55	VEGFB;VEGFC;MIF

E-box binding (GO:0070888)	0.01	-2.62	1.45	NEUROD2;TCF12;AHR;ASCL2
hydrolase activity, acting on glycosyl bonds (GO:0016798)	0.02	-2.24	1.24	GALC;ABHD10;MAN2A1;MPG;HYAL3;PHKB;MUTYH
carbohydrate derivative transporter activity (GO:1901505)	0.03	-2.22	1.23	SLC35A2;SLC35B2;SLC29A3
beta-catenin binding (GO:0008013)	0.01	-2.22	1.23	SOX17;APC;TBL1XR1;RORA;CD2AP
disulfide oxidoreductase activity (GO:0015036)	0.02	-2.16	1.19	COIL;TMX1;GLRX
nucleobase-containing compound transmembrane transporter activity (GO:0015932)	0.02	-2.15	1.19	SLC35A2;SLC35B2;SLC29A3
helicase activity (GO:0004386)	0.03	-2.02	1.12	CHD9;DDX56;ATRX;HELZ;MCM5;NBN;GTF2F2
Rab GTPase binding (GO:0017137)	0.03	-1.98	1.09	FER;KIAA1432;ERC1;RABGGTB;EVI5
zinc ion binding (GO:0008270)	0.03	-1.98	1.07	KDM5A;PRDM4;MYT1L;SP140;MYCBP2;XIAP;RORA;TCF20;ZDHHC2;EEA1;ZMAT1;RNF139;MAN2A1;ZMYM6;PGGT1B;RNF111;WHSC1L1;ERAP2;MMP1;USP3;ATRX;LNPEP;PRKCA;QTRTD1;RABGGTB;THAP11;PIAS1;CNOT4;AFG3L2;RFWD2;COMMD3-BMI1;MDM4;RBAK;RFPL4AL1
protein kinase A binding (GO:0051018)	0.04	-1.97	1.05	AKAP11;PKIA;C2ORF88
acetyl-CoA:L-lysine N6-acetyltransferase (GO:0090595)	0.03	-1.90	1.05	KANSL1;KAT5;TAF10;CSRP2BP
histone acetyltransferase activity (GO:0004402)	0.03	-1.88	1.04	KANSL1;KAT5;TAF10;CSRP2BP
transcription corepressor binding (GO:0001222)	0.01	-1.83	1.01	CNOT2;RORA
**KEGG pathway**	**P-value**	**Z-score**	**Combined Score**	**Genes involved**
NF-kappa B signaling pathway_Homo sapiens_hsa04064	0.00	-1.77	0.96	CCL13;TICAM2;XIAP;BCL10;ERC1;TLR4;NFKB1
Ras signaling pathway_Homo sapiens_hsa04014	0.01	-1.96	0.65	PAK1;VEGFB;VEGFC;SHOC2;PRKCA;KRAS;ELK1;NFKB1;GNG13;FGF21
Pathways in cancer_Homo sapiens_hsa05200	0.05	-1.90	0.63	MMP1;VEGFB;VEGFC;XIAP;PRKCA;MLH1;NFKB1;GNG13;MSH6;APC;FAS;KRAS;FGF21
Endometrial cancer_Homo sapiens_hsa05213	0.02	-1.83	0.61	APC;KRAS;MLH1;ELK1
Hepatitis B_Homo sapiens_hsa05161	0.03	-1.81	0.60	TICAM2;FAS;PRKCA;KRAS;ELK1;TLR4;NFKB1
AGE-RAGE signaling pathway in diabetic complications_Homo sapiens_hsa04933	0.05	-1.80	0.60	VEGFB;VEGFC;PRKCA;KRAS;NFKB1
**Reactome pathway**	**P-value**	**Z-score**	**Combined Score**	**Genes involved**
Transcriptional Regulation by TP53_Homo sapiens_R-HSA-3700989	0.00	-2.31	2.26	PRKAA1;DYRK2;COX18;TAF10;PPP2R5C;MLH1;RABGGTB;GTF2F2;CNOT4;CNOT6;KAT5;CNOT11;CNOT2;FAS;MDM4;COX14;NBN
Generic Transcription Pathway_Homo sapiens_R-HSA-212436	0.00	-2.23	2.19	ZNF430;PRKAA1;DYRK2;ZNF350;CHD9;COX18;RORA;KAT5;COX14;NBN;ZNF587;ZNF641;RNF111;TAF10;MED8;PPP2R5C;RABGGTB;MLH1;MED26;GTF2F2;CNOT4;CNOT6;CNOT11;TBL1XR1;CNOT2;ZNF558;FAS;ZNF436;MDM4
Gene Expression_Homo sapiens_R-HSA-74160	0.00	-2.15	2.11	TRAM1;DYRK2;PDCD7;CHD9;HNRNPR;RORA;WDR43;KAT5;DGCR8;NBN;ZNF641;RNF111;RPS12;GTF3A;MED8;PRKCA;RPSA;PPP2R5C;RABGGTB;QTRTD1;MED26;GTF2F2;TBL1XR1;ZNF558;ZNF436;SNRPA1;HIST1H2BC;ZNF430;FYTTD1;PRKAA1;ZNF350;COX18;SSR1;COX14;IARS2;ZNF587;SUZ12;DARS;TAF10;MLH1;EIF2S2;KIAA0391;TRIT1;CNOT4;CNOT6;CNOT11;PRKRA;CNOT2;POLR3E;FAS;MDM4
Chromatin organization_Homo sapiens_R-HSA-4839726	0.00	-2.14	2.10	SUZ12;KDM5A;PBRM1;SMARCB1;KAT5;KANSL1;TBL1XR1;TAF10;CSRP2BP;RCOR1;WHSC1L1;HIST1H2BC

Chromatin modifying enzymes_Homo sapiens_R-HSA-3247509	0.00	-2.13	2.09	SUZ12;KDM5A;PBRM1;SMARCB1;KAT5;KANSL1;TBL1XR1;TAF10;CSRP2BP;RCOR1;WHSC1L1;HIST1H2BC
TP53 regulates transcription of additional cell cycle genes whose exact role in the p53 pathway remain uncertain_Homo sapiens_R-HSA-6804115	0.00	-2.00	1.96	CNOT4;CNOT6;CNOT11;CNOT2
Deadenylation of mRNA_Homo sapiens_R-HSA-429947	0.00	-1.68	1.64	CNOT4;CNOT6;CNOT11;CNOT2

Supplementary table-7: Variable important in Projection Score for the Genes Differentially expressed in GR.A:SAHIO as compared to GR.B:SAHNIO

Multivariate analysis PBMC						
**SR.no**	**GENES**	**Component. 1**	**Component. 2**	**Component. 3**	**Component. 4**	**Component. 5**
1	PPEF2	22.174	21.591	18.897	18.114	17.213
2	NOTCH2	9.3426	9.1368	8.0076	7.6737	7.2918
3	GUCY2F	7.0165	6.9355	6.0845	5.8286	5.537
4	LYZ	4.8239	4.3274	4.6174	4.7176	4.5311
5	CCL5	3.9406	3.6033	4.219	4.0976	4.915
6	SH2D1A	3.7362	3.7236	3.2296	3.0934	2.9414
7	CELA3A	3.7011	3.6703	3.1707	3.0363	2.885
8	ORMDL2	3.6535	3.7004	3.2034	3.0688	2.9208
9	FCN1	3.3809	2.9537	3.6669	3.6642	3.5172
10	EMP3	3.1206	2.5333	4.0827	4.1271	3.9206
11	S100A4	3.0393	2.6155	3.2808	3.1794	3.0325
12	OR2M5	3.0369	2.8352	2.4695	2.3651	2.2554
13	CD52	2.9961	2.8152	2.5147	2.4493	2.9454
14	C1orf228	2.9799	2.614	2.2843	2.1889	2.0822
15	GZMH	2.9734	2.7024	2.3866	2.3013	2.187
16	HIST1H2BF	2.9395	2.9354	2.6115	2.5051	2.3847
17	IFI30	2.8831	2.4145	3.1653	3.0964	2.963
18	CD163	2.8767	3.1844	3.3907	3.7383	3.8887
19	CD300E	2.8668	2.4562	3.3245	3.3865	3.227
20	OR9Q1	2.8335	2.7753	2.3947	2.2972	2.1868
21	CD74	2.8306	2.4483	2.8876	2.9287	2.8137
22	ANKRD9	2.8023	3.0426	2.6279	2.5247	2.3987
23	LY6G6F	2.7968	2.8256	2.4652	2.3623	2.2441
24	VCAN	2.7687	2.4716	2.5956	2.5092	2.5377
25	LSM10	2.758	2.8303	2.5168	2.4204	2.2993
26	ZNF157	2.7573	2.6935	2.3321	2.2345	2.1337
27	SRGN	2.7353	2.4623	2.6488	2.5594	2.4336
28	CCR1	2.6119	2.2001	2.8624	2.8238	2.721
29	IL1F10	2.6035	2.3698	2.076	1.9902	1.9033
30	FAM110D	2.5911	2.4445	2.1124	2.0225	1.9229
31	EVI2B	2.5836	2.3392	2.4861	2.3949	2.3008
32	WDR74	2.5183	2.0121	4.2558	4.4665	4.2543
33	METTL24	2.5177	2.4084	2.0922	2.0034	1.9125
34	FFAR1	2.4348	2.6471	2.2784	2.199	2.0904
35	CYBB	2.4051	2.1849	2.2098	2.1199	2.1316
36	LOC100996515	2.4004	2.4861	2.1449	2.0541	1.9512

37	CLEC12A	2.3926	2.0858	2.377	2.3417	2.2619
38	C22orf23	2.3648	2.2232	1.9432	1.8606	1.7674
39	KRTAP10-11	2.3427	2.1054	1.8355	1.7733	1.6871
40	TFF2	2.3308	1.9568	1.7	1.6596	1.6156
41	CARD16	2.3207	1.9437	2.7677	2.7344	2.6213
42	KRTAP10-10	2.2169	2.2214	1.9165	1.839	1.7544
43	C5AR2	2.2071	2.0597	1.7949	1.7199	1.6385
44	TMEM203	2.2029	2.0433	1.7793	1.7051	1.633
45	HOXB8	2.193	2.0639	1.7812	1.7057	1.6204
46	PPBP	2.1726	2.2846	2.5516	2.5618	3.1143
47	TUBB1	2.1717	2.4481	3.0092	2.9534	3.5671
48	LOC728392	2.1558	1.9016	1.7705	1.6958	1.611
49	CD68	2.1523	1.8343	2.3201	2.3038	2.1951
50	GLYATL2	2.1437	2.1746	1.8707	1.803	1.7141
51	XCL2	2.1389	1.9621	1.7074	1.6356	1.5755
52	S100A12	2.1383	2.0365	1.7886	1.7171	1.6374
53	ARMS2	2.1264	1.9571	1.7049	1.6387	1.56
54	TMSB4X	2.1247	2.4515	2.8559	2.7808	3.1621
55	NDNL2	2.1201	2.0924	1.825	1.7476	1.6896
56	IGLL1	2.1038	1.726	1.6041	1.5463	1.5123
57	LCP1	2.0973	1.9728	2.2501	2.1657	2.1961
58	PLBD1	2.0939	1.8726	1.8804	1.8171	1.9728
59	LOC100653515	2.0932	1.7079	1.7969	1.7204	1.6575
60	PPP1R15A	2.0815	2.1997	2.5723	2.4721	2.4623
61	CD300A	2.0452	1.7402	2.3134	2.2213	2.1854
62	MRPS18B	2.0431	2.0218	1.7432	1.6758	1.5919
63	ACTB	2.0416	2.0541	2.681	2.5719	2.4501
64	JUN	2.0399	1.6451	1.931	1.8614	1.7976
65	GIMAP7	2.0353	2.1193	3.2385	3.1647	3.1445
66	STARD3NL	2.0321	2.0174	1.7776	1.7096	1.631
67	SOX2	2.0278	2.2153	1.9064	1.8324	1.741
68	OAZ1	2.0211	1.919	2.403	2.3018	2.2812
69	NGB	1.9996	1.9639	1.6984	1.6263	1.5532
70	CT45A1	1.9832	1.7775	1.5424	1.4924	1.4188
71	OR4N4	1.9826	1.9511	1.6829	1.6137	1.5396
72	GPR149	1.9635	1.5833	1.4034	1.3491	1.2878
73	OXER1	1.9578	1.7366	1.5053	1.4416	1.413
74	CHRNB3	1.9569	1.9667	1.693	1.629	1.5495
75	DUSP6	1.9484	1.7696	1.9737	1.9039	1.9067
76	C6orf25	1.9448	2.1159	2.3604	2.3582	2.8398
77	NRGN	1.9301	1.8669	2.0115	2.0527	2.5402

78	TYROBP	1.9299	1.5697	2.1641	2.3742	2.2555
79	PF4	1.9182	2.8401	3.4142	3.3096	3.5973
80	TLR8	1.8996	1.7012	1.7631	1.693	1.6624
81	GPR78	1.8925	1.7545	1.5248	1.4628	1.3896
82	SLC25A6_1	1.8892	1.6364	1.8542	1.827	1.737
83	CORT	1.8865	2.007	1.7861	1.731	1.6465
84	OR52N2	1.882	1.969	1.6955	1.6278	1.5542
85	C1orf233	1.8818	2.0493	1.7632	1.7074	1.6267
86	PTPRC	1.8768	1.6102	1.826	1.7751	1.7528
87	C10orf35	1.8669	1.5842	1.3724	1.3394	1.2777
88	KLHDC9	1.8631	2.0526	1.7661	1.7216	1.6403
89	NACA2	1.8608	1.6792	1.6584	1.5885	1.5755
90	SH3BGRL3	1.8462	1.6419	1.7979	1.7248	1.6945
91	FAM131C	1.8439	2.0226	1.7541	1.6798	1.6086
92	KRTAP16-1	1.8414	2.642	2.4186	2.3638	2.4729
93	ARHGDIB	1.8309	2.053	2.709	2.5943	2.4644
94	LTA4H	1.8263	1.5418	2.0504	1.9864	1.9129
95	GPR27	1.8191	2.5555	2.3301	2.3648	2.5274
96	LOC101928975	1.8026	1.8355	1.583	1.5171	1.4447
97	NLRP5	1.7999	1.6948	1.4688	1.4063	1.3366
98	PRR15	1.7939	1.4398	1.271	1.234	1.2386
99	CEACAM21	1.7871	1.7422	1.5078	1.4458	1.376
100	OR4D5	1.7849	1.7703	1.5255	1.4616	1.401
101	OCEL1	1.7744	1.6707	1.4439	1.3859	1.3204
102	LENEP	1.7726	1.5533	1.358	1.3009	1.2379
103	KLK13	1.7682	1.7561	1.5376	1.4758	1.4153
104	AKAP17A1	1.7573	1.6209	1.5174	1.4529	1.3809
105	GGTLC2	1.7516	1.7274	1.5019	1.44	1.3698
106	RASL12	1.7474	1.7149	1.4787	1.4274	1.3561
107	KISS1	1.7423	1.7385	1.5021	1.4391	1.3707
108	IKBKG	1.7416	1.7652	1.5759	1.5139	1.4418
109	CSF3R	1.7378	2.5062	2.7996	2.7495	2.6128
110	MS4A7	1.7373	1.4787	1.8394	1.7665	1.7521
111	NRIP2	1.737	1.5551	1.3934	1.3342	1.2679
112	PLAC8	1.7321	1.4783	1.9406	1.9031	1.8355
113	GFI1B	1.7292	1.484	1.3064	1.2655	1.2184
114	VGLL1	1.7282	1.5792	1.3707	1.3163	1.2747
115	C14orf180	1.7255	1.9274	1.658	1.6026	1.5252
116	HIST1H4J	1.7147	2.3189	2.3501	2.2519	2.1715
117	ITGB3	1.7084	1.8566	2.1409	2.1362	2.4476
118	FAM132B	1.7083	1.7842	1.5348	1.4699	1.3965

119	LGALS3	1.7011	1.6869	1.4739	1.4469	1.4299
120	HOXB5	1.6991	1.8494	1.591	1.5421	1.4651
121	VPS9D1	1.678	1.6386	1.4194	1.3596	1.2935
122	RGS2	1.6777	1.3759	2.0273	2.0745	2.0234
123	ITGB2	1.6769	1.7225	2.2554	2.2069	2.1215
124	PRAME	1.6728	1.608	1.3856	1.3301	1.2663
125	TNS4	1.6672	1.7217	1.4844	1.4224	1.3513
126	ATP5G1	1.6665	1.5902	1.3947	1.3358	1.2697
127	FAM89A	1.6644	1.502	1.3281	1.2966	1.2323
128	TGFB1	1.6616	1.4665	1.8156	1.7406	1.7956
129	TAS1R1	1.6574	1.8367	1.581	1.5303	1.4543
130	SH3BGR	1.6521	1.5111	1.3103	1.2551	1.2116
131	GGN	1.6475	1.6781	1.4802	1.4275	1.3561
132	MED7	1.6404	1.5267	1.3274	1.2719	1.232
133	TTLL10	1.6378	1.3404	1.2772	1.2234	1.181
134	RTL1	1.6325	1.3018	1.1563	1.1268	1.1783
135	IHH	1.6275	1.6854	1.4501	1.389	1.3194
136	SELE	1.6275	1.508	1.3009	1.2455	1.1994
137	ZNF784	1.6238	1.4394	1.2696	1.2203	1.161
138	MNDA	1.623	1.3752	1.8565	1.7812	1.829
139	CNIH3	1.617	1.7071	1.4695	1.4069	1.3394
140	AWAT1	1.611	1.6812	1.4488	1.3916	1.3253
141	UCP2	1.6078	1.4113	1.6727	1.6018	1.5356
142	S100A11	1.603	1.3181	1.8048	1.7481	1.7174
143	NFKBID	1.6018	1.3274	1.3109	1.2961	1.2314
144	VIM	1.5981	1.6021	1.5215	1.462	1.5479
145	CLDN19	1.5917	1.5916	1.3778	1.3196	1.256
146	TBC1D10B	1.5917	1.593	1.4457	1.3967	1.3268
147	RFXANK	1.5888	1.7494	1.5193	1.4577	1.3847
148	MMGT1	1.587	1.4841	1.3655	1.3081	1.3038
149	CD44	1.5794	1.3978	1.4202	1.376	1.388
150	KIF3B	1.5765	1.5013	1.3328	1.2762	1.2145
151	ZNF385A	1.5739	1.4318	1.5514	1.509	1.4722
152	IFNGR2	1.5691	1.5797	1.5218	1.4932	1.8478
153	AIF1	1.5612	1.3978	1.9486	1.9714	1.8864
154	BLOC1S3	1.5576	1.5627	1.3638	1.3069	1.2465
155	LRRC30	1.5562	2.2204	2.0289	2.0454	2.2507
156	TMPRSS4	1.5527	1.5194	1.3099	1.2553	1.1936
157	HLA-E	1.5509	1.4513	1.8675	1.7883	1.7851
158	KIAA1211L	1.5506	1.3947	1.2014	1.1555	1.0983
159	H2AFX	1.55	1.4577	1.3231	1.2788	1.222

160	BATF2	1.5489	1.2369	1.286	1.2621	1.254
161	NCCRP1	1.5448	1.2329	1.127	1.0891	1.0755
162	LRRC10B	1.5411	1.2679	1.1053	1.1121	1.0829
163	CD300C	1.537	1.478	1.3384	1.3188	1.2535
164	NUDT8	1.5368	1.6793	1.511	1.448	1.4227
165	MSN	1.5329	1.3904	1.6324	1.5773	1.4986
166	OR1J2	1.5302	1.612	1.3932	1.3543	1.2865
167	GPN3	1.5264	1.3006	1.1741	1.1514	1.1897
168	PIAS1	1.5255	1.4218	1.318	1.2628	1.2
169	RPLP1	1.5232	1.6434	1.4504	1.3886	1.3199
170	SLFNL1	1.5162	1.345	1.1693	1.129	1.0749
171	SRRT	1.5154	1.3599	1.2641	1.2164	1.1591
172	USP43	1.5141	1.2455	1.1042	1.0594	1.0066
173	FKBP1A	1.5121	1.3479	1.5257	1.4802	1.4574
174	S100A9	1.5112	1.6173	1.7561	1.739	2.1095
175	SELL	1.5089	1.2417	1.7298	1.6929	1.7152
176	SAT1	1.5079	1.3016	2.2191	2.1288	2.0921
177	FAM228B	1.5057	1.2106	1.0748	1.0562	1.0698
178	PCBP1	1.5053	1.3601	1.5693	1.5063	1.4659
179	GBGT1	1.5019	1.6567	1.4341	1.3903	1.322
180	DEFA1	1.4979	1.3071	1.1398	1.0996	1.0531
181	SLC25A2	1.497	1.4663	1.276	1.2227	1.1638
182	TNFAIP8L2	1.4954	1.3095	1.4608	1.4004	1.4788
183	TULP3	1.4947	1.4467	1.2471	1.1943	1.1452
184	CSF1R	1.4945	1.2617	1.5987	1.5614	1.5295
185	BTBD18	1.4866	1.613	1.4016	1.3421	1.2802
186	CMTM7	1.4853	1.4049	1.2526	1.2085	1.1793
187	CASP1	1.4845	1.2406	1.6564	1.6509	1.6085
188	ARPC2	1.4756	1.3099	1.4826	1.4266	1.4022
189	FYB	1.4741	1.296	1.8579	1.7928	1.703
190	CAPZA1	1.474	1.2808	1.3959	1.3383	1.2798
191	CPA4	1.4704	1.4233	1.2385	1.1956	1.1522
192	EFNA3	1.47	1.5367	1.322	1.2805	1.2185
193	GPR88	1.4659	1.4158	1.225	1.1729	1.1142
194	BRINP1	1.4653	1.4771	1.2709	1.2173	1.1696
195	B3GNT4	1.4636	1.3006	1.1318	1.0932	1.0856
196	SERF1B	1.4635	1.2632	1.119	1.1051	1.1419
197	EIF1	1.4634	1.3985	1.3288	1.2977	1.2871
198	NSA2	1.4602	1.4336	1.2586	1.205	1.1645
199	SEPHS2	1.4569	1.4213	1.2787	1.2253	1.1868
200	LILRB1	1.45	1.2318	1.5226	1.4891	1.452

201	TSPAN11	1.4414	1.1993	1.0524	1.0622	1.0591
202	HIST1H4E	1.4407	1.5255	2.1809	2.1194	2.0618
203	NUDT16L1	1.4373	1.4555	1.2894	1.2366	1.1761
204	SYT9	1.4361	1.3916	1.1976	1.1472	1.1041
205	CLEC1B	1.4356	1.333	1.4732	1.4508	1.8583
206	HIST1H2AK	1.4355	2.3221	2.3517	2.3902	2.52
207	FAM117A	1.4352	1.3645	1.2073	1.1561	1.0983
208	P2RY13	1.4319	1.1972	1.4077	1.4243	1.4301
209	RPL36A-HNRNPH2	1.4285	1.2956	1.5934	1.5305	1.4544
210	ZNF433	1.4284	1.4308	1.2384	1.1901	1.1349
211	TBC1D27	1.428	1.2433	1.0828	1.0949	1.1471
212	ARMC1	1.4258	1.3631	1.1847	1.1361	1.1094
213	RABAC1	1.4251	1.5146	1.4249	1.3648	1.3023
214	HIST2H2AA3	1.4247	1.2348	1.2849	1.2451	1.3774
215	PRAMEF8	1.4234	1.2121	1.0441	1.0449	1.011
216	VPS45	1.4211	1.2679	1.0985	1.053	1.0113
217	TXNIP	1.4208	1.3714	1.3265	1.3711	1.3497
218	BMP8A	1.4205	1.6031	1.3802	1.3247	1.2588
219	ANKRD32	1.4182	1.3653	1.1753	1.1328	1.079
220	C2orf88	1.4169	1.3185	1.4463	1.4222	1.7603
221	CEBPB	1.4166	1.2132	1.2582	1.2058	1.1655
222	MAPK3	1.4162	1.5877	1.4337	1.3729	1.3094
223	SIRPB2	1.4157	1.1297	1.3386	1.2936	1.2435
224	RCSD1	1.4151	1.2192	2.0355	1.962	1.8894
225	XRCC3	1.4132	1.5195	1.308	1.2582	1.1964
226	FAM216A	1.4129	1.4596	1.2556	1.217	1.1852
227	ZNF556	1.4127	1.3559	1.1673	1.1384	1.0815
228	CABLES1	1.4122	1.1517	0.99786	0.98811	0.97375
229	FAM32A	1.4069	1.2206	1.4345	1.3759	1.3129
230	CSNK1D	1.4066	1.1454	1.1629	1.1171	1.0633
231	SLC25A43	1.406	1.26	1.1169	1.0862	1.1068
232	PRF1	1.4022	1.5407	1.3766	1.3245	1.2667
233	SNCA	1.4005	1.1226	1.5535	1.5981	1.5662
234	C22orf15	1.4004	1.1256	1.0478	1.0502	1.0221
235	HIST1H2AE	1.3988	1.2007	1.6025	1.5496	1.7125
236	TSPAN7	1.3954	1.3909	1.1966	1.1471	1.1063
237	EEF1A1	1.3917	1.2313	1.4409	1.3867	1.3404
238	FAM86B1	1.3914	1.1288	1.0238	0.98058	0.9539
239	GPS2	1.3889	1.3349	1.1929	1.144	1.0873
240	LILRB2	1.3878	1.1542	1.52	1.4778	1.4173
241	ITGA4	1.3875	1.2694	1.1948	1.146	1.1899

242	ZNF224	1.3867	1.3629	1.1861	1.1415	1.0897
243	KLF2	1.3849	1.3236	1.7572	1.695	1.7799
244	FAM49A	1.383	1.2048	1.2751	1.2411	1.2275
245	HIST1H2BD	1.3824	1.5085	1.5271	1.4825	1.4129
246	TAC3	1.381	1.2954	1.1149	1.0745	1.0211
247	C1QTNF2	1.3781	1.1868	1.0507	1.008	0.96866
248	GBP1	1.3776	1.1433	1.5406	1.5034	1.5311
249	CXorf21	1.3754	1.163	1.4664	1.5333	1.5012
250	GDPD2	1.3707	1.2454	1.0752	1.0303	0.98314
251	PPT1	1.3696	1.2458	1.1567	1.1158	1.1709
252	ZNF414	1.3648	1.3305	1.1534	1.1098	1.0846
253	GLP2R	1.3632	1.436	1.2453	1.1972	1.1391
254	KHDC1	1.362	1.142	1.0146	0.98464	0.93534
255	KCNA7	1.3619	1.1487	1.0046	0.96584	0.92388
256	CSRNP1	1.3614	1.1	1.2134	1.1638	1.1619
257	ZNF267	1.3553	1.2357	1.1434	1.122	1.0661
258	CWC15	1.3551	1.5226	1.3209	1.2675	1.2048
259	CPVL	1.3541	1.1453	1.3904	1.4645	1.4166
260	C3orf38	1.3532	1.2877	1.1269	1.079	1.0618
261	ISOC2	1.352	1.333	1.1498	1.1035	1.0483
262	NANOS3	1.3509	1.0875	0.99955	0.95979	1.0373
263	TMEM115	1.3507	1.6874	1.4548	1.4011	1.3326
264	UBA52	1.3507	1.2506	1.1762	1.1272	1.0728
265	CDPF1	1.349	1.0804	0.9312	0.97227	1.0014
266	SLC25A6	1.3479	1.4041	2.0856	1.9975	1.9245
267	KIAA1644	1.3466	1.0738	0.9339	0.90972	0.91627
268	TSPAN12	1.3455	1.3148	1.1317	1.0871	1.033
269	TNNC1	1.345	1.2118	1.0802	1.0361	1.0077
270	ADAM17	1.3419	1.6661	1.6967	1.8614	1.9498
271	UST	1.3392	1.3099	1.1319	1.0838	1.064
272	KCTD21	1.3365	1.2278	1.0626	1.0216	0.97081
273	RAB40C	1.3362	1.4777	1.277	1.2334	1.1717
274	HOXC5	1.3315	1.467	1.2659	1.6469	1.8323
275	CRMP1	1.3271	1.3249	1.1446	1.0975	1.0426
276	TMIE	1.3198	1.4153	1.2176	1.177	1.1483
277	ZNF543	1.317	1.2385	1.0683	1.0231	0.97616
278	GBP5	1.3148	1.1075	1.3555	1.3962	1.3704
279	UGT3A2	1.3135	1.3569	1.1686	1.1211	1.0772
280	TLR4	1.3131	1.1512	1.2266	1.1744	1.216
281	CMTM5	1.3122	1.9956	1.7221	1.7616	1.71
282	ARPC4	1.3114	1.1391	1.5168	1.4524	1.4501

283	TMEM191B	1.3094	1.4859	1.2805	1.2423	1.1967
284	ZNF345	1.3082	1.2023	1.0366	0.99504	0.94751
285	IQCF1	1.3079	1.2328	1.0624	1.0289	1.0164
286	GPR150	1.3053	1.6148	1.417	1.3673	1.3004
287	COX14	1.3052	1.2802	1.1343	1.115	1.0594
288	ZNF576	1.3045	1.2083	1.0475	1.0128	0.96205
289	C22orf26	1.3039	1.9208	1.7918	1.8371	2.0781
290	LOC554223	1.3033	1.2031	1.0376	0.99658	0.98598
291	SEPW1	1.3008	1.1284	1.0142	0.97426	0.92837
292	ARMCX5	1.2993	1.3428	1.1598	1.1104	1.0879
293	VNN2	1.2993	1.0841	1.3743	1.3389	1.3139
294	ETV2	1.2988	1.0439	0.9405	0.90098	0.86815
295	ATG3	1.2978	1.0711	1.4142	1.362	1.3079
296	IST1	1.2977	1.2431	1.1365	1.089	1.0472
297	HNRNPH2	1.2956	1.1614	1.5537	1.5	1.4402
298	C9orf89	1.2945	1.5329	1.4668	1.415	1.3763
299	RPTN	1.2923	1.312	1.1299	1.0879	1.039
300	B3GNT8	1.2922	1.079	1.0151	0.99026	0.9637
301	NUDT18	1.291	1.2897	1.1206	1.0745	1.0225
302	BCL2L15	1.2903	1.0496	0.91815	0.90593	0.94606
303	UQCRHL	1.2859	1.076	1.5027	1.4546	1.409
304	DPEP3	1.2858	1.1827	1.0179	0.98042	0.93222
305	MON1B	1.2858	1.0294	1.0387	1.0133	0.97285
306	CNTNAP1	1.2829	1.189	1.0365	0.9957	0.94652
307	RIT1	1.282	1.1478	1.164	1.1148	1.3085
308	WFIKKN1	1.2802	1.3077	1.1269	1.0895	1.0353
309	GNRH2	1.2789	1.3797	1.1872	1.1368	1.0801
310	RASD2	1.2788	1.0398	0.95315	0.9498	1.0191
311	IGSF6	1.2768	1.0849	1.3062	1.2713	1.2739
312	DXO	1.2767	1.1762	1.0922	1.0618	1.0096
313	MOGAT2	1.2753	1.0326	0.91673	0.87912	0.8376
314	FTH1	1.2748	1.3007	1.4242	1.375	1.3468
315	C1orf94	1.2743	1.3672	1.1767	1.1269	1.0707
316	ANGPTL6	1.2736	1.2119	1.0473	1.0088	0.96995
317	LYPLA2	1.2699	1.1778	1.0321	1.0009	0.9514
318	LYN	1.2674	1.0912	1.2285	1.1971	1.1825
319	ZNF665	1.2665	1.167	1.0041	0.96283	0.95133
320	ADAM10	1.2574	1.1177	1.1466	1.0988	1.0487
321	RNF214	1.2559	1.4115	1.2798	1.2434	1.1822
322	EFEMP2	1.2545	1.2409	1.0697	1.0427	0.99099
323	CCDC135	1.2544	1.1203	0.97037	0.94227	0.9018

324	ATP6V1A	1.2537	1.1162	1.1153	1.0757	1.1
325	HIST1H4F	1.2537	1.0349	1.4301	1.3871	1.4146
326	RSPH10B	1.2532	1.2019	1.1273	1.1033	1.0493
327	BCL6	1.2507	1.8031	2.0147	2.014	1.9166
328	TSSK4	1.2503	1.1148	1.0465	1.0413	0.99439
329	FOXD1	1.2483	1.8176	1.6718	1.6011	1.6063
330	GABARAPL2	1.2479	1.116	1.2275	1.2239	1.1698
331	USHBP1	1.2476	1.0044	0.87284	0.89195	0.89682
332	CKS1B	1.2467	1.2984	1.118	1.0715	1.0375
333	SNAPIN	1.2463	1.0163	0.90663	0.8683	0.83017
334	RAB7A	1.2457	1.4203	1.4288	1.3763	1.3089
335	FLI1	1.2448	1.1167	1.4276	1.3696	1.3095
336	WIPF1	1.2435	1.279	1.5113	1.4771	1.4563
337	ARG1	1.24	1.8495	1.7654	1.6913	1.6458
338	GNG13	1.2399	1.4633	1.3305	1.4253	1.4747
339	IL7R	1.2344	1.1423	1.0552	1.0978	1.1682
340	POU4F1	1.233	1.3869	1.1979	1.1538	1.0961
341	EVI2A	1.2313	1.0293	1.1008	1.0883	1.061
342	GP6	1.2276	1.1657	1.1681	1.1184	1.1317
343	C10orf54	1.2267	1.0208	1.4224	1.4666	1.3973
344	GP9	1.2262	1.152	1.1355	1.3908	1.4866
345	VAMP7	1.2256	1.074	1.24	1.1963	1.1559
346	CRBN	1.2235	1.2402	1.08	1.0343	1.0032
347	TFE3	1.2192	0.97609	0.98943	0.94986	0.91037
348	VAX2	1.2172	1.8176	1.5888	1.5886	1.518
349	HMGN3	1.2166	1.3873	1.1987	1.1507	1.1021
350	FBXL5	1.2111	1.0135	1.2703	1.2169	1.2186
351	CYP1B1	1.2092	1.2243	1.0707	1.1675	1.1104
352	IFI44	1.2091	1.2482	1.1195	1.1146	1.1121
353	LST1	1.2087	0.96761	1.586	1.6223	1.545
354	AP4M1	1.2068	1.171	1.029	0.99161	0.94215
355	ZFP36	1.2068	1.205	2.1117	2.0649	1.9623
356	PRRT1	1.2054	1.467	1.3561	1.8229	1.8065
357	ERAP2	1.2046	1.0477	1.1143	1.1492	1.1165
358	PLEK	1.2032	0.98764	1.3659	1.3313	1.2651
359	NOG	1.203	1.414	1.3708	1.4157	1.3727
360	CCDC109B	1.2023	1.434	1.2472	1.194	1.164
361	USP17L17	1.2021	1.1342	0.97775	0.9363	0.8904
362	NR5A2	1.2011	1.1205	0.96397	0.92369	0.89036
363	TNFSF10	1.1996	1.0079	1.2178	1.2069	1.2165
364	PRCP	1.1995	1.0512	1.1435	1.146	1.1071

365	HKR1	1.1979	0.97003	0.85761	0.82325	0.7821
366	SCGB2B2	1.1974	1.6971	1.5168	1.5245	1.5875
367	L1CAM	1.197	1.3936	1.2008	1.1603	1.1046
368	REM2	1.1948	0.96214	0.95076	0.91136	0.86629
369	USP17L30	1.1945	0.96979	0.84653	0.81049	0.77072
370	SLC34A1	1.1935	1.0036	0.87	0.88907	0.88564
371	FCGR1A	1.1931	0.9751	1.4579	1.4674	1.4048
372	SLC8A1	1.1928	1.0107	1.2608	1.2179	1.1612
373	POLR2J3	1.192	1.0748	1.1151	1.0713	1.0802
374	BTBD19	1.1906	1.0065	1.0341	0.99324	1.0181
375	PRNP	1.1899	1.1489	1.0115	0.98918	0.95099
376	CDH15	1.1898	1.1727	1.0111	0.9698	0.93199
377	TAF10	1.1873	1.1238	1.099	1.113	1.2599
378	FLJ44635	1.1866	0.98868	1.3496	1.3236	1.2611
379	TRIM58	1.1865	1.0716	1.2354	1.2479	1.4766
380	TSR2	1.1858	1.1982	1.064	1.0222	0.97461
381	LSMD1	1.1855	1.2255	1.0718	1.0265	0.98122
382	AAMDC	1.1851	1.076	0.93187	0.92528	1.0126
383	MAT2B	1.185	1.0403	1.2059	1.1584	1.1224
384	DYNC2LI1	1.1844	1.2925	1.1178	1.0767	1.0241
385	RSPH10B2	1.1835	1.2349	1.1289	1.0822	1.0324
386	TLR2	1.181	1.0897	1.0054	0.96264	1.0268
387	DCTN3	1.1799	1.2276	1.0751	1.0325	0.98466
388	ACSBG1	1.1791	1.0822	0.94099	0.90133	0.85875
389	FANCL	1.1779	1.2753	1.102	1.0564	1.0095
390	CDKN2D	1.1772	1.1696	1.7026	1.6562	2.1168
391	TMIGD2	1.1771	1.0996	1.0306	1.0005	0.98023
392	HIST2H2BE	1.1758	1.7151	1.6806	1.628	1.6122
393	TMEM52B	1.173	1.0685	0.92275	0.885	0.86357
394	PRICKLE3	1.1724	1.1133	0.99473	0.95693	0.90904
395	CLCC1	1.1714	1.1422	0.98916	0.94752	0.92174
396	ANXA5	1.1678	1.029	1.0627	1.023	1.1001
397	CAAP1	1.1675	1.1015	0.95686	0.92462	0.89977
398	PABPN1L	1.1666	1.2214	1.0674	1.031	0.98548
399	KRTAP29-1	1.1644	1.047	0.90121	0.91799	0.89148
400	PLA2G4B	1.1637	1.1835	1.032	0.9881	0.95703
401	CDH17	1.1635	1.0683	0.91902	0.88513	0.91744
402	ZNF70	1.1629	1.1502	0.99578	0.9539	0.93703
403	LAT2	1.1626	1.1131	1.1171	1.0711	1.0191
404	SLC25A34	1.1625	1.0847	0.95291	0.92109	0.90846
405	LILRA1	1.1624	0.98196	1.1957	1.1449	1.1156

406	RPL23	1.1609	1.1816	1.0882	1.0429	0.99077
407	ENKD1	1.1602	1.2662	1.0899	1.0447	0.99368
408	PNPLA2	1.1599	0.97598	1.0699	1.0261	0.97807
409	RAB25	1.1598	1.4627	1.2685	1.2993	1.2366
410	HLA-B	1.1597	1.0846	2.0294	2.0249	1.9237
411	FAM110A	1.1596	1.0622	1.0534	1.034	1.1148
412	PSME1	1.1561	1.0771	0.95521	0.93646	1.0705
413	CDC20	1.1552	1.037	0.89959	0.86493	0.82329
414	CYP26A1	1.154	1.1603	0.99815	0.96213	0.91678
415	CCR2	1.1528	0.98534	1.1473	1.1055	1.1496
416	TBC1D25	1.1526	1.1964	1.0938	1.0495	0.9972
417	POM121C	1.1522	1.0528	0.92565	0.88646	0.84207
418	PPP1R7	1.151	1.2465	1.0775	1.0322	0.99164
419	TLN1	1.1478	1.0923	1.4064	1.3546	1.3639
420	SLC16A14	1.1469	1.6535	1.4833	1.4786	1.5459
421	KLHL40	1.1464	1.2873	1.1091	1.0626	1.0095
422	CUTA	1.1458	1.125	0.98487	0.94344	0.90236
423	RBMXL3	1.1453	1.3212	1.1771	1.4218	1.5524
424	C1orf162	1.1452	0.96222	1.1541	1.1246	1.0943
425	NEUROG2	1.1438	1.7428	1.5315	1.6631	1.5826
426	ZIC4	1.1425	1.1289	0.97175	0.93821	0.89123
427	RLIM	1.1421	1.1671	1.0483	1.0039	1.1037
428	CLCNKB	1.1391	1.0543	0.90741	0.86875	0.84876
429	IFI6	1.1388	0.99414	1.1388	1.1336	1.1319
430	FAM187B	1.1379	1.1264	1.0236	1.2388	1.3919
431	CFD	1.1378	1.1121	0.95769	0.92397	0.95355
432	ACAP1	1.1374	0.92116	0.89881	0.86156	0.83127
433	C3orf36	1.1364	1.6936	1.5849	1.5804	1.8395
434	CHID1	1.1331	1.2383	1.0716	1.0342	1.016
435	GCH1	1.1329	0.97122	1.0739	1.0536	1.0442
436	SNN	1.1322	1.0482	1.2676	1.2227	1.3991
437	SOX10	1.1319	1.1002	0.94898	0.91766	0.93957
438	NAMPT	1.1317	0.91493	1.546	1.4931	1.4504
439	HIST1H4I	1.1307	1.8114	1.8271	1.8029	2.0293
440	COA5	1.1289	1.0879	0.95262	0.91232	0.8867
441	ASMT	1.1288	1.8007	1.8426	1.7642	1.7547
442	RPL29	1.1277	0.93364	1.267	1.2425	1.2568
443	USP17L21	1.1275	1.1221	0.96783	0.92757	0.89521
444	SYT3	1.127	0.95994	0.84204	0.81022	0.77019
445	CYP1A1	1.1268	1.0595	0.9143	0.87631	0.83255
446	GAGE12G	1.1267	1.6753	1.5513	1.602	1.7169

447	CD247	1.1265	1.1928	1.1344	1.0867	1.0335
448	RGS18	1.1263	1.8497	2.2155	2.2196	2.6844
449	LTK	1.1237	1.0131	0.88239	0.85613	0.81622
450	PIK3R2	1.1234	0.93667	1.1859	1.1454	1.0926
451	RAP1GAP2	1.1225	1.0083	1.067	1.0297	0.97977
452	DHRS13	1.1211	0.99497	0.87022	0.83458	0.81312
453	RASL10A	1.1208	1.4786	1.2727	1.3275	1.2611
454	FAM43A	1.1205	0.95435	0.87724	0.90509	0.90259
455	LILRB3	1.1201	0.9045	1.4211	1.3708	1.3091
456	APOL5	1.1199	0.89852	0.79736	0.76523	0.74178
457	IL22	1.1198	1.2767	1.1171	1.0974	1.0434
458	SERP1	1.1181	1.0127	1.0152	0.9774	0.93155
459	OCM2	1.1169	1.0299	0.92112	0.88413	0.83986
460	PRKX	1.1148	1.0435	0.91654	0.88151	0.9344
461	CDC42EP3	1.1142	1.033	0.90344	0.8864	0.89992
462	BTG2	1.114	0.90634	1.7476	1.7245	1.6452
463	EN2	1.1131	1.3262	1.1417	1.105	1.0564
464	LSP1	1.1128	0.93235	1.41	1.3548	1.3127
465	DGAT2	1.1127	0.93958	0.84358	0.82032	0.79075
466	MITD1	1.1124	0.93391	1.2442	1.1931	1.1346
467	GMNN	1.1123	1.141	1.0001	0.99174	0.94273
468	PLCD3	1.1112	0.92218	0.82301	0.79363	0.83639
469	CTSZ	1.1109	0.97777	1.1801	1.1374	1.0815
470	PLAUR	1.11	1.0959	1.5554	1.4899	1.4352
471	THAP7	1.1096	1.3503	1.2384	1.2043	1.1628
472	C19orf26	1.109	1.2639	1.1042	1.0727	1.0198
473	RSG1	1.106	0.98588	0.8645	0.82836	0.78688
474	RHOJ	1.1057	0.91325	0.78693	0.78625	0.86851
475	SLC48A1	1.1057	1.0627	0.94635	0.98385	1.0285
476	PQBP1	1.1056	1.0138	1.2572	1.2037	1.1573
477	ELSPBP1	1.1037	1.0884	0.93633	0.89967	0.86173
478	CTSD	1.1026	1.0889	1.1671	1.1205	1.0746
479	HIST1H4B	1.1026	0.9055	1.4679	1.4145	1.8718
480	SLC22A8	1.1016	0.8832	0.83099	0.7993	0.8173
481	ZDHHC20	1.101	0.98594	0.9174	0.92091	0.88242
482	RBMX	1.0996	0.96435	0.94386	0.91706	0.89686
483	UBE2L6	1.0994	1.0156	1.3243	1.2849	1.2221
484	EXOSC2	1.0992	0.93098	0.85403	0.82335	0.81133
485	PLK3	1.099	0.91163	0.98141	0.9418	0.92067
486	CIR1	1.0972	0.91412	1.1991	1.2807	1.2174
487	STRA8	1.0972	1.6106	1.5148	1.4518	1.3827

488	DESI1	1.0959	1.028	0.93563	0.91031	0.86485
489	YWHAE	1.0958	1.1719	1.6006	1.5404	1.5588
490	ALPL	1.0956	0.91463	0.79857	0.76505	0.73086
491	ZNF561	1.0945	1.0524	0.90859	0.87041	0.84105
492	TUBA1A	1.0939	0.89943	1.1381	1.0961	1.0899
493	SLC16A6	1.0931	0.90101	1.0251	0.98994	0.9462
494	C1QTNF9B	1.092	1.5714	1.4484	1.3958	1.3619
495	TMEM107	1.092	0.87421	0.90993	0.89769	0.87188
496	ICAM4	1.0919	1.8346	1.9369	1.8658	1.8128
497	CEACAM18	1.091	0.98422	0.85813	0.82614	0.7889
498	TMED4	1.0903	1.3947	1.2003	1.1642	1.1079
499	COPG2	1.0886	1.0798	0.92939	0.8931	0.85755
500	MCL1	1.0879	0.87386	1.7432	1.6696	1.5902
501	C5orf20	1.0878	1.1888	1.0301	0.99997	0.95615
502	CCDC124	1.0872	0.90846	0.81927	0.7893	0.76522
503	MFSD1	1.0871	1.056	1.1355	1.105	1.0609
504	MACROD1	1.0844	1.325	1.1417	1.1303	1.0745
505	HIST1H3D	1.0835	1.2201	1.0534	1.0111	0.97114
506	LPAR6	1.0834	1.0066	0.87707	0.88608	0.9169
507	GDF15	1.0818	0.90078	0.79097	0.75841	0.72067
508	STX10	1.0796	0.90734	0.85475	0.81835	0.77916
509	THAP11	1.0788	1.125	0.97582	1.0384	1.0023
510	MEGF9	1.0784	0.89433	1.156	1.1094	1.1545
511	LURAP1L	1.0783	1.2423	1.0687	1.0232	0.97307
512	ASCL2	1.0777	1.1047	0.99463	0.97478	0.92598
513	SEBOX	1.077	1.2321	1.0825	1.2977	1.4069
514	LGALS1	1.0765	0.91543	1.0523	1.031	1.0983
515	ARMCX3	1.0755	0.9149	1.2315	1.1829	1.1856
516	RNF219	1.0754	0.96124	0.85847	0.82475	0.78551
517	VHLL	1.0742	0.95452	0.90168	0.87366	0.83342
518	SAMD9	1.0731	0.93707	0.95676	0.97123	1.2195
519	PPM1M	1.0727	0.86518	0.96704	0.93467	0.94922
520	SPATA2	1.0721	1.3529	1.1734	1.127	1.0752
521	DACT3	1.0719	1.068	0.9238	0.88795	0.85083
522	OCA2	1.0718	0.96605	0.83126	0.79685	0.761
523	HIST1H2AC	1.0717	1.6973	2.092	2.051	2.4183
524	EMD	1.0714	1.1268	0.96933	1.0504	1.0247
525	ZFP91	1.0709	1.035	0.89453	0.96799	0.9199
526	SFT2D2	1.0707	1.0277	0.88658	0.87117	0.9036
527	CEBPD	1.0685	0.95542	1.5133	1.4737	1.4409
528	TMED7	1.0684	0.9991	0.87103	0.86637	0.86868

529	TMEM114	1.0673	1.0515	0.90894	0.87153	0.84893
530	GSTM5	1.0651	0.92517	0.85119	0.81613	0.81377
531	VAMP3	1.0645	0.94368	0.9263	0.88947	0.84654
532	DNASE1L1	1.0624	0.95757	0.97055	0.9427	0.93359
533	COL23A1	1.0618	0.98537	0.84954	0.81428	0.77395
534	HMGB1	1.0618	0.94869	1.0978	1.0833	1.1845
535	DCAF15	1.0607	1.2912	1.1232	1.0763	1.0244
536	TNFRSF10C	1.0605	1.767	1.8835	1.8345	1.755
537	S100A6	1.0604	1.0327	1.6973	1.7009	1.6198
538	OR5C1	1.0599	0.84576	0.7872	0.81547	0.87522
539	C9orf72	1.0585	0.90424	0.90539	0.89207	0.84923
540	PPP2R5D	1.056	1.0937	0.95292	0.91665	0.87386
541	MYADM	1.0559	0.84814	1.3267	1.3145	1.2614
542	PPAT	1.0558	1.055	0.90789	0.86921	0.86684
543	CNBD2	1.0541	0.84031	0.72921	0.69815	0.66693
544	FAR1	1.0541	0.92101	0.88403	0.87836	0.91813
545	C1orf54	1.0524	0.95958	0.84152	0.85064	0.87513
546	EPSTI1	1.0522	0.9916	0.85411	0.89328	0.89253
547	GBP4	1.052	0.9122	0.97951	0.94887	0.97463
548	LRFN4	1.0519	1.1173	0.96298	1.2232	1.3335
549	ANKRD30A	1.0498	0.99259	0.86108	0.82439	0.82492
550	LILRA3	1.0498	0.83706	1.2919	1.6512	1.576
551	FKBP11	1.0493	1.2317	1.0596	1.0162	0.96738
552	WDFY1	1.0485	0.93811	0.89445	0.85636	0.86023
553	ZDHHC2	1.0473	1.0619	0.95787	0.91897	0.87499
554	ASNA1	1.0472	0.94696	0.83513	0.80438	0.77292
555	SARS	1.0469	0.85156	0.80952	0.79147	0.75918
556	PLA2G10	1.0468	1.5706	1.4797	1.4232	1.3896
557	RBM38	1.0467	0.96264	0.91931	0.89644	0.95585
558	ENKUR	1.0463	1.259	1.0972	1.0505	1.004
559	HIST1H3J	1.0463	1.6397	1.6655	1.7105	1.626
560	NRBF2	1.0462	0.95019	0.87061	0.92934	0.93384
561	CSNK1A1L	1.0461	1.1353	1.0113	0.9751	1.0123
562	SLC4A3	1.0454	1.0179	0.87933	0.84288	0.84762
563	ZNF813	1.0446	1.123	1.0925	1.054	1.0012
564	TMED7-TICAM2	1.0434	1.0439	0.898	0.86165	0.81928
565	COIL	1.0429	1.0657	0.91732	0.87933	0.91159
566	ADRBK2	1.0421	0.92949	0.89075	0.85918	0.85989
567	KRT84	1.0403	1.0703	0.94105	0.95782	0.91559
568	SIX1	1.0392	1.1313	0.97512	0.93794	0.89357
569	SPATA31A7	1.0381	1.5348	1.3985	1.3449	1.4657

570	EEPD1	1.038	0.84035	0.77257	0.75345	0.72826
571	ACRBP	1.0379	1.2533	1.2056	1.1542	1.1072
572	MIS18BP1	1.0372	0.84324	1.2155	1.1701	1.1115
573	USP17L19	1.0339	0.96978	0.85585	0.8328	0.79932
574	ABHD1	1.0311	1.0997	0.94617	0.91838	0.88357
575	DEFB116	1.0307	1.1942	1.0344	1.0169	0.96633
576	CST7	1.0305	0.8322	0.72121	0.75793	1.0393
577	P2RY8	1.0299	0.84314	1.1809	1.2344	1.174
578	TAS1R2	1.0298	1.0757	0.92672	0.9102	0.87976
579	MTA3	1.0297	0.82046	0.7058	0.69282	0.66507
580	DAND5	1.0283	0.89697	0.78835	0.75564	0.71782
581	NUTF2	1.028	1.0576	0.90984	0.87257	0.83047
582	C7orf73	1.0279	0.93112	1.0529	1.0479	1.2259
583	RELB	1.0267	0.84282	0.82637	0.80002	0.78837
584	SHOC2	1.0261	0.90644	0.91004	0.88088	0.84188
585	ATP6V0D1	1.0244	0.84043	1.1631	1.1135	1.065
586	PTCHD1	1.0217	0.89377	0.78184	0.75143	0.74473
587	LYL1	1.0205	1.0328	0.88846	1.0401	0.9968
588	LCTL	1.0204	0.84916	0.73278	0.71193	0.68493
589	C12orf75	1.0203	1.1577	1.1344	1.0861	1.0348
590	CMIP	1.0201	0.9201	1.0126	0.97531	1.0983
591	ZFY	1.0188	1.0774	1.003	1.0453	0.99861
592	SPAG11B	1.0182	1.1345	0.9767	0.93608	0.88957
593	GPRIN1	1.0179	1.0036	0.88382	0.8463	0.81061
594	ATXN7L3	1.0162	1.0539	0.94438	0.90414	0.8629
595	CMTM3	1.0153	0.82495	1.0223	1.0196	1.0239
596	RPL15	1.0145	0.87484	0.97734	0.93705	0.90741
597	RGL4	1.014	1.549	1.452	1.5337	1.674
598	NBN	1.0136	0.90376	0.86933	0.88675	0.85991
599	APOBEC3A	1.0134	0.85009	1.7457	1.7828	1.6936
600	H1FX	1.0129	0.90995	0.94188	0.95761	0.99125
601	GAPDH	1.0128	1.3907	1.4212	1.3679	1.3018
602	ZNF329	1.0123	0.94421	0.81924	0.80046	0.78752
603	HMX3	1.0122	0.93792	0.91606	1.2079	1.2277
604	TMEM50B	1.0122	0.94099	0.8266	0.81514	0.80474
605	GPR183	1.0117	0.96056	0.82635	0.80652	0.76652
606	PGAP3	1.0115	0.81213	0.74414	0.72752	0.73515
607	SSX3	1.0106	1.4858	1.3333	1.2772	1.2164
608	SLC9A7	1.0103	1.0877	0.94909	0.91693	0.87504
609	PLP2	1.01	1.0522	0.95256	0.91204	0.87131
610	PAK1	1.0086	0.84484	1.0694	1.0349	1.0246

611	UCN2	1.0086	0.94794	0.81601	0.82437	0.85303
612	IL10RB	1.0084	0.80927	1.2608	1.2831	1.2471
613	SIGLEC10	1.0076	0.86756	1.0059	0.97079	0.92746
614	KLF11	1.0073	0.94663	0.81765	0.92667	0.8855
615	ARL4C	1.0053	1.0005	0.86157	0.83286	0.79168
616	RPS12	1.004	0.90474	0.93192	0.90276	0.92729
617	GPR52	1.0037	1.0044	0.86412	0.87375	0.83903
618	TNFSF8	1.0034	0.95799	0.8257	0.79708	0.75759
619	PMM2	1.0017	0.79841	0.74973	0.72677	0.71978
620	ADO	1.0002	1.107	1.0913	1.0448	0.99278

Supplementary table 8: Univariate and Multivariate analysis of the genes differentially regulated in Liver biopsy and PBMC of GR.A:SAHIO as compared to GR.B:SAHNIO

Liver biopsy GR.A:SAHIO vs GR.B:SAHNIO							PBMC GR.A:SAHIO vs GR.B:SAHNIO						
Univariate analysis						Multivariate	Univariate analysis PBMC						Multivariate
GENE	Mean (SD) of GR.B:SAHNIO	Mean (SD) of GR.A:SAHIO	LOG FC GR.A:SAHIO/ GR.B:SAHNIO	p-value	GR.A:SAHIO/ GR.B:SAHNIO	VIP	GENE	Mean (SD) of GR.B:SAHNIO	Mean (SD) of GR.A:SAHIO	Fold Change LOG GR.A:SAHIO/ GR.B:SAHNIO	p-value	GR.A:SAHIO/ GR.B:SAHNIO	VIP
**CD163**	**-1.898 (0.174)**	**3.416 (1.567)**	**5.314**	**0.0016**	**Up**	**1.43**	**CD163**	**-1.765 (0.096)**	**3.176 (1.304)**	**4.941**	**0.00328**	**UP**	**2.8767**
**TMED7**	**-1.080 (0.319)**	**1.945 (4.951)**	**3.025**	**0.04195**	**UP**	**1.82**	**ADAM17**	**-0.823 (0.390)**	**1.482 (0.971)**	**2.305**	**0.00099**	**UP**	**1.3419**
**ADAM17**	**-0.804 (0.226)**	**1.448 (0.794)**	**2.252**	**0.0026**	**Up**	**1.05**	**TMED7**	**0.655 (2.177)**	**-1.180 (0.371)**	**-1.835**	**0.04526**	**Down**	**1.0684**
APOE	-19.759 (2.153)	35.566 (39.034)	55.325	0.001	Up	14.92	GPR149	-1.204 (0.248)	2.168 (5.232)	3.372	0.01120	UP	1.9635
HP	-17.132 (44.209)	30.838 (36.572)	47.97	0.042	Up	12.94	LENEP	-1.087 (0.433)	1.957 (5.830)	3.044	0.02897	UP	1.7726
ALB	-10.261 (32.248)	18.469 (36.673)	28.73	0.019	Up	7.75	NRIP2	-1.065 (0.509)	1.918 (6.125)	2.983	0.02897	UP	1.737
HSPA1B	-3.416 (0.975)	6.149 (19.053)	9.565	0.042	Up	2.58	VPS45	-0.872 (0.595)	1.569 (4.806)	2.441	0.04195	UP	1.4211
HSPA1A	-3.379 (0.382)	6.083 (16.523)	9.462	0.002	Up	2.55	BCL2L15	-0.791 (0.742)	1.425 (3.496)	2.216	0.04314	UP	1.2903
HSPB1	-3.108 (2.345)	5.595 (7.179)	8.703	0.004	Up	2.35	RSPH10B	-0.769 (0.648)	1.384 (1.347)	2.153	0.00699	UP	1.2532
AGRN	-2.875 (1.362)	5.175 (9.628)	8.05	0.029	Up	2.17	SNAPIN	-0.764 (0.770)	1.376 (2.459)	2.14	0.02897	UP	1.2463
CYB5A	-2.588 (1.895)	4.658 (8.886)	7.246	0.042	Up	1.95	HKR1	-0.735 (0.249)	1.323 (3.224)	2.058	0.00699	UP	1.1979
ERBB2	-2.523 (0.091)	4.541 (0.927)	7.064	0.0001	Up	1.91	USP17L30	-0.733 (0.409)	1.319 (3.161)	2.052	0.01592	UP	1.1945
HSPA1L	-2.442 (0.768)	4.396 (12.776)	6.838	0.029	Up	1.84	RSPH10B2	-0.726 (0.723)	1.307 (1.050)	2.033	0.00699	UP	1.1835
MAGED1	-2.378 (0.668)	4.281 (9.081)	6.659	0.0112	Up	1.80	SYT3	-0.691 (0.237)	1.244 (3.530)	1.935	0.02897	UP	1.127
SERPINA5	-2.116 (4.246)	3.809 (10.693)	5.925	0.0446	Up	1.60	APOL5	-0.687 (0.607)	1.236 (2.324)	1.923	0.02897	UP	1.1199
TAGLN	-2.096 (1.181)	3.772 (5.530)	5.868	0.029	Up	1.58	OCM2	-0.685 (0.677)	1.233 (1.123)	1.918	0.00399	UP	1.1169
CP	-2.033 (1.535)	3.660 (1.173)	5.693	0.0001	Up	1.54	DGAT2	-0.683 (0.315)	1.229 (3.460)	1.912	0.03270	UP	1.1127
MAF	-1.750 (1.937)	3.150 (5.128)	4.9	0.019	Up	1.32	OR5C1	-0.650 (0.471)	1.170 (2.678)	1.82	0.02897	UP	1.0599
NFKBIL1	-1.737 (3.962)	3.126 (8.922)	4.863	0.0446	Up	1.31	LCTL	-0.626 (0.708)	1.127 (1.656)	1.753	0.01898	UP	1.0204
C21orf33	-1.712 (1.221)	3.081 (6.429)	4.793	0.0233	Up	1.29	ADO	0.614 (1.185)	-1.104 (0.578)	-1.718	0.01120	Down	1.0002
ZBED1	-1.670 (1.261)	3.007 (6.627)	4.677	0.042	Up	1.26	TNFSF8	0.615 (1.513)	-1.108 (0.708)	-1.723	0.02325	Down	1.0034
TPST2	-1.642 (1.094)	2.955 (5.241)	4.597	0.042	Up	1.24	GPR52	0.616 (1.894)	-1.108 (0.648)	-1.724	0.04455	Down	1.0037
PRKCDBP	-1.627 (1.079)	2.929 (4.161)	4.556	0.0321	Up	1.23	RPS12	0.616 (2.551)	-1.108 (0.352)	-1.724	0.04526	Down	1.004

TSKU	-1.587 (2.414)	2.857 (8.584)	4.444	0.0233	Up	1.20	ARL4C	0.617 (2.285)	-1.110 (0.285)	-1.727	0.04526	Down	1.0053
NUDC	-1.552 (3.125)	2.794 (6.732)	4.346	0.019	Up	1.17	PAK1	0.619 (3.072)	-1.114 (0.303)	-1.733	0.02897	Down	1.0086
CYGB	-1.540 (0.434)	2.771 (4.617)	4.311	0.002	Up	1.16	TMEM50B	0.621 (1.705)	-1.118 (0.533)	-1.739	0.04455	Down	1.0122
IQCJ-SCHIP1	-1.515 (1.290)	2.728 (4.069)	4.243	0.042	Up	1.14	NBN	0.622 (2.173)	-1.119 (0.233)	-1.741	0.03855	Down	1.0136
PRDX4	-1.503 (0.216)	2.705 (7.031)	4.208	0.0321	Up	1.14	ZFY	0.625 (1.323)	-1.125 (0.440)	-1.75	0.01120	Down	1.0188
SLC25A1	-1.489 (1.343)	2.680 (4.249)	4.169	0.0327	Up	1.12	C12orf75	0.626 (2.122)	-1.127 (0.432)	-1.753	0.02280	Down	1.0203
MAGEF1	-1.447 (1.202)	2.605 (2.844)	4.052	0.0049	Up	1.09	SHOC2	0.629 (1.832)	-1.133 (0.595)	-1.762	0.04195	Down	1.0261
ST13	-1.416 (1.596)	2.548 (3.622)	3.964	0.0233	Up	1.07	NUTF2	0.631 (1.386)	-1.135 (0.086)	-1.766	0.00957	Down	1.028
BLVRB	-1.410 (1.157)	2.539 (4.390)	3.949	0.0228	Up	1.07	COIL	0.640 (1.968)	-1.151 (0.258)	-1.791	0.01627	Down	1.0429
EFHD1	-1.391 (0.589)	2.505 (5.490)	3.896	0.029	Up	1.05	TICAM2	0.640 (1.394)	-1.152 (0.263)	-1.792	0.00506	Down	1.0434
PROS1	-1.357 (0.525)	2.443 (5.520)	3.8	0.029	Up	1.03	ZNF813	0.641 (1.306)	-1.153 (0.933)	-1.794	0.01198	Down	1.0446
TRAPPC12	-1.353 (1.197)	2.436 (2.897)	3.789	0.0453	Up	1.02	RBM38	0.642 (2.236)	-1.156 (0.998)	-1.798	0.01627	Down	1.0467
PCBD1	-1.351 (0.381)	2.431 (4.140)	3.782	0.0408	Up	1.02	ZDHHC2	0.642 (1.002)	-1.156 (0.902)	-1.798	0.00399	Down	1.0473
GPX1	-1.343 (0.343)	2.418 (2.413)	3.761	0.0248	Up	1.01	DNASE1L1	0.652 (2.241)	-1.173 (0.594)	-1.825	0.04526	Down	1.0624
ZNF622	-1.333 (2.299)	2.400 (4.940)	3.733	0.0453	Up	1.01	ZFP91	0.657 (2.149)	-1.182 (0.255)	-1.839	0.02897	Down	1.0709
-	-	-	-	-	-	-	ASCL2	0.661 (1.393)	-1.190 (0.897)	-1.851	0.01627	Down	1.0777
-	-	-	-	-	-	-	THAP11	0.662 (2.419)	-1.191 (0.438)	-1.853	0.02897	Down	1.0788
-	-	-	-	-	-	-	SLC48A1	0.678 (2.097)	-1.221 (0.239)	-1.899	0.00759	Down	1.1057
-	-	-	-	-	-	-	PRKX	0.684 (2.551)	-1.231 (0.190)	-1.915	0.00099	Down	1.1148
-	-	-	-	-	-	-	CFD	0.698 (1.700)	-1.256 (0.637)	-1.954	0.02897	Down	1.1378
-	-	-	-	-	-	-	TAF10	0.728 (3.552)	-1.311 (0.235)	-2.039	0.01898	Down	1.1873
-	-	-	-	-	-	-	ERAP2	0.739 (3.211)	-1.330 (0.342)	-2.069	0.04455	Down	1.2046
-	-	-	-	-	-	-	IFI44	0.742 (3.239)	-1.335 (0.280)	-2.077	0.04455	Down	1.2091
-	-	-	-	-	-	-	VAMP7	0.752 (3.079)	-1.353 (0.265)	-2.105	0.04526	Down	1.2256
-	-	-	-	-	-	-	GNG13	0.761 (3.462)	-1.369 (0.184)	-2.13	0.03737	Down	1.2399
-	-	-	-	-	-	-	GABARAPL2	0.765 (2.890)	-1.378 (0.367)	-2.143	0.04526	Down	1.2479
-	-	-	-	-	-	-	ATP6V1A	0.769 (2.804)	-1.384 (0.376)	-2.153	0.01627	Down	1.2537
-	-	-	-	-	-	-	IGSF6	0.783 (3.772)	-1.410 (0.344)	-2.193	0.04526	Down	1.2768
-	-	-	-	-	-	-	COX14	0.801 (2.824)	-1.441 (0.158)	-2.242	0.01627	Down	1.3052
-	-	-	-	-	-	-	TLR4	0.805 (3.504)	-1.450 (0.164)	-2.255	0.04195	Down	1.3131
-	-	-	-	-	-	-	C3orf38	0.830 (2.541)	-1.494 (0.565)	-2.324	0.02325	Down	1.3532
-	-	-	-	-	-	-	ITGA4	0.851 (3.123)	-1.532 (0.327)	-2.383	0.01120	Down	1.3875
-	-	-	-	-	-	-	C2orf88	0.869 (6.050)	-1.564 (0.210)	-2.433	0.04195	Down	1.4169
-	-	-	-	-	-	-	CLEC1B	0.881 (6.553)	-1.585 (0.208)	-2.466	0.03213	Down	1.4356
-	-	-	-	-	-	-	TNFAIP8L2	0.917 (4.235)	-1.651 (0.179)	-2.568	0.01094	Down	1.4954
-	-	-	-	-	-	-	PIAS1	0.936 (2.982)	-1.684 (0.153)	-2.62	0.03270	Down	1.5255

-	-	-	-	-	-	-	CD300C	0.943 (2.529)	-1.697 (0.313)	-2.64	0.02265	Down	1.537
-	-	-	-	-	-	-	MMGT1	0.973 (3.289)	-1.752 (0.233)	-2.725	0.00759	Down	1.587
-	-	-	-	-	-	-	CLEC12A	1.468 (6.261)	-2.642 (0.147)	-4.11	0.01898	Down	2.3926

Supplementary table-9: Spearman Correlation Analysis for sCD163 and TNF-α with survival in SAH

Spearman's rho Correlations												
**Spearman's rho**	**Correlation**	**Survival**	**sCD163**	**He-HP**	**Ferritin**	**Iron**	**TNF-Alpha**	**1 Month Survival**	**15 Days Survival**	**CTP**	**MELD**	**SOFA**
Survival	CC	1.000	-.788**	.471**	-0.037	-.532**	-.688**	.596**	.313**	-.340**	-.250*	-.331**
	P-Val	.	0.000	0.000	0.712	0.000	0.000	0.000	0.001	0.000	0.012	0.001
sCD163	CC	-.788**	1.000	-.351**	0.093	.510**	.583**	-.512**	-.218*	.276**	0.121	.297**
	P-Val	0.000	.	0.000	0.356	0.000	0.000	0.000	0.028	0.005	0.226	0.003
He-HP	CC	.471**	-.351**	1.000	-0.107	-.215*	-.379**	.318**	0.155	-.228*	-0.019	-0.076
	P-Val	0.000	0.000	.	0.286	0.031	0.000	0.001	0.121	0.022	0.853	0.453
Ferritin	CC	-0.037	0.093	-0.107	1.000	0.082	0.057	-0.004	-0.054	-0.022	-0.037	-0.003
	P-Val	0.712	0.356	0.286	.	0.413	0.574	0.966	0.592	0.824	0.712	0.978
Iron	CC	-.532**	.510**	-.215*	0.082	1.000	.445**	-.247*	-0.158	-0.014	0.157	0.077
	P-Val	0.000	0.000	0.031	0.413	.	0.000	0.013	0.115	0.890	0.116	0.442
TNF-Alpha	CC	-.688**	.583**	-.379**	0.057	.445**	1.000	-.237*	-0.175	0.045	-0.015	0.023
	P-Val	0.000	0.000	0.000	0.574	0.000	.	0.017	0.081	0.653	0.879	0.818
1 Month Survival	CC	.596**	-.512**	.318**	-0.004	-.247*	-.237*	1.000	.491**	-.286**	-.307**	-.309**
	P-Val	0.000	0.000	0.001	0.966	0.013	0.017	.	0.000	0.004	0.002	0.002
15 Days Survival	CC	.313**	-.218*	0.155	-0.054	-0.158	-0.175	.491**	1.000	-0.146	-.265**	-.234*
	P-Val	0.001	0.028	0.121	0.592	0.115	0.081	0.000	.	0.145	0.007	0.019
CTP	CC	-.340**	.276**	-.228*	-0.022	-0.014	0.045	-.286**	-0.146	1.000	.369**	.481**
	P-Val	0.000	0.005	0.022	0.824	0.890	0.653	0.004	0.145	.	0.000	0.000
MELD	CC	-.250*	0.321	-0.019	-0.037	0.157	-0.015	-.307**	-.265**	.369**	1.000	.504**
	P-Val	0.012	0.022**	0.853	0.712	0.116	0.879	0.002	0.007	0.000	.	0.000
SOFA	CC	-.331**	.297**	-0.076	-0.003	0.077	0.023	-.309**	-.234*	.481**	.504**	1.000
	P-Val	0.001	0.003	0.453	0.978	0.442	0.818	0.002	0.019	0.000	0.000	.
** Correlation is significant at the 0.01 level												
* Correlation is significant at the 0.05 level												

Supplementary table-10: Pairwise comparison of ROC curves using a Hanley & McNeil, 1983 method

sCD163 ~ TNFa	
Difference between areas	0.0981
Standard Error [a]	0.0327
95% Confidence Interval	-0.00596 to 0.122
z statistic	1.778
Significance level	P <0.05
sCD163 ~ Iron	
Difference between areas	0.149
Standard Error [a]	0.0464
95% Confidence Interval	0.0579 to 0.240
z statistic	3.209
Significance level	P <0.00
sCD163 ~ HE	
Difference between areas	0.292
Standard Error [a]	0.0545
95% Confidence Interval	0.185 to 0.398
z statistic	5.351
Significance level	P < 0.00
sCD163 ~ HE_HP	
Difference between areas	0.191
Standard Error [a]	0.0498
95% Confidence Interval	0.0937 to 0.289
z statistic	3.843
Significance level	P <0.00
